# Identification of an Unusual Pattern of Global Gene Expression in Group B *Streptococcus* Grown in Human Blood

**DOI:** 10.1371/journal.pone.0007145

**Published:** 2009-09-23

**Authors:** Laurent Mereghetti, Izabela Sitkiewicz, Nicole M. Green, James M. Musser

**Affiliations:** 1 Center for Molecular and Translational Human Infectious Diseases Research, The Methodist Hospital Research Institute, Houston, Texas, United States of America; 2 Université François Rabelais de Tours, IFR136, EA3854 “Bactéries et risque materno-foetal”, Tours, France; 3 CHU de Tours, Tours, France; University of Hyderabad, India

## Abstract

Because passage of the bacterium to blood is a crucial step in the pathogenesis of many group B *Streptococcus* (GBS) invasive infections, we recently conducted a whole-genome transcriptome analysis during GBS incubation *ex vivo* with human blood. In the current work, we sought to analyze in detail the difference in GBS gene expression that occurred in one blood sample (donor A) relative to other blood samples. We incubated GBS strain NEM316 with fresh heparinized human blood obtained from healthy volunteers, and analyzed GBS genome expression and cytokine production. Principal component analysis identified extensive clustering of the transcriptome data among all samples at time 0. In striking contrast, the whole bacterial gene expression in the donor A blood sample was significantly different from the gene expression in all other blood samples studied, both after 30 and 90 min of incubation. More genes were up-regulated in donor A blood relative to the other samples, at 30 min and 90 min. Furthermore, there was significant variation in transcript levels between donor A blood and other blood samples. Notably, genes with the highest transcript levels in donor A blood were those involved in carbohydrate metabolism. We also discovered an unusual production of proinflammatory and immunomodulatory cytokines: MIF, tPAI-1 and IL-1β were produced at higher levels in donor A blood relative to the other blood samples, whereas GM-CSF, TNF-α, IFN-γ, IL-7 and IL-10 remained at lower levels in donor A blood. Potential reasons for our observations are that the immune response of donor A significantly influenced the bacterial transcriptome, or both GBS gene expression and immune response were influenced by the metabolic status of donor A.

## Introduction

*Streptococcus agalactiae* (group B *Streptococcus*, GBS) is a common inhabitant of the human gut and genitourinary tract, and also rarely colonizes other anatomic sites [1]. GBS is also the major cause of neonatal infections, with an estimated incidence of ∼34 cases per 100,000 live births in the United States [2]. For several years, phylogenetic analyses have identified high-risk lineages, grouping strains more frequently implicated in neonatal diseases, regardless of the genetic typing method used [3], [4], [5], [6], [7]. More recently, it was also proposed that the major hyperinvasive neonatal clone had emerged from a bovine ancestor [8], suggesting that a greater genetic distance from other human lineages might explain the higher invasiveness of this lineage among neonates [9].

However, the phylogeny does not correlate as well with the epidemiology of GBS non-pregnant adult infections, which have drastically increased in the last two decades [10]. Conversely, GBS predominantly infects elderly and immunocompromised patients, highlighting the idea that underlying medical conditions increase the risk of non-pregnant adults to develop invasive GBS infections. For example, the most common predisposing conditions observed in adult populations are diabetes and malignancy [10]. Indeed, it is well-known that components of the host immune system, especially the innate response and the polymorphonuclear neutrophils, may be detrimentally altered in diabetic patients, however, the exact mechanisms responsible for this are less well characterized [11]. Similarly, immune response is decreased in patients affected by cancer, either because of malignancy, antineoplastic therapy, or both.

Using the serotype III strain NEM316 isolated from a patient with fatal septicaemia [12], we recently analyzed transcriptome changes that occur during incubation of GBS in human blood [13], a crucial step in many invasive infections. We found that GBS gene expression was similar during incubation in blood obtained from seven distinct donors [13]. However, during ongoing studies of the interaction of GBS with human blood, we generated data suggesting that the transcriptome of strain NEM316 was significantly different when incubated with the blood of an eighth donor (arbitrarily referred to as donor A) (Mereghetti unpublished data), suggesting inter-individual variability in GBS gene expression. In the present study, we sought to analyze the differences of GBS gene expression that occurred in this donor's blood. We also explored human blood cytokine response that may contribute to GBS transcriptome differences. We found that fewer bacterial genes were down-regulated in donor A blood. In addition, the transcript levels were also different in donor A blood relative to the other samples. Furthermore, cytokine production was different in donor A blood, either before any contact with GBS or after 90 min of incubation with this pathogen.

## Results

### GBS transcriptome is different in donor A blood sample

**Figure 1:**
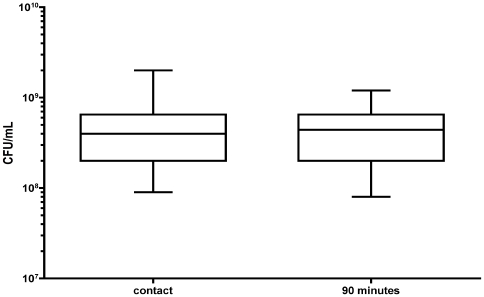
Number of bacterial cells during incubation with human blood. The number of CFU/ml in the donor A blood sample (marked by a cross) is similar to the number of CFU/ml in the seven other blood samples at time 0 and after 90 min of incubation at 37°C.

Blood obtained from each of the eight donors was processed separately. We found by CFU counting that there was no significant difference in the number of input GBS inoculated into the donor A blood sample and the seven other blood samples (arbitrarily named donor B to H) (Fig. 1). We also documented that there was no significant difference between the donor A blood sample and the other blood samples in the number of viable bacteria after 90 min of incubation in the blood (Fig. 1).

**Figure 2:**
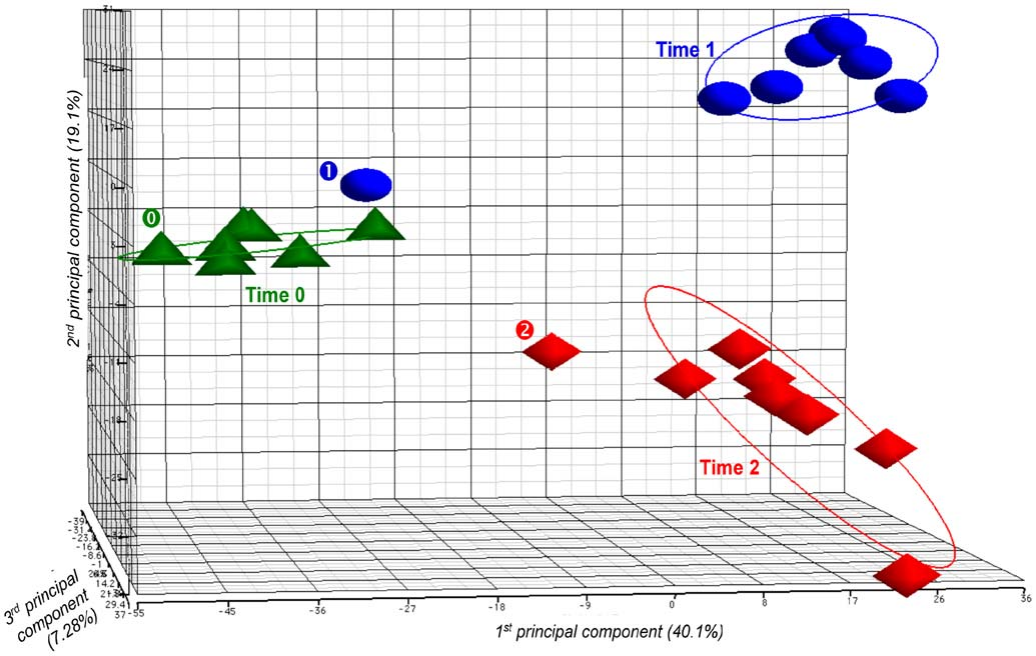
Principal component analysis (PCA) plot showing transcriptome differences between expression microarray data of GBS NEM316 strain incubated in human blood at 37°C. Samples were analyzed at time 0, and after 30 min and 90 min of incubation. The PCA plot captures the variance in a dataset in terms of principal components and displays the most significant of these on the x, y, and z axes. The percentages of the total variation that are accounted for by the 1^st^, 2^nd^, and 3^rd^ principal components are shown on the x-, y- and z-axes labels. Plots are colored and shaped by time point. Plots for donor A blood sample are specifically marked (“
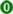
” for time 0, “
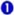
” for time 1, and “
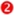
” for time 2).

Principal component analysis (PCA) identified extensive clustering of the transcriptome data among all samples at time 0, including the donor A blood sample (Fig. 2). Conversely, the whole bacterial gene expression in the donor A blood sample was different from the gene expression in the seven other blood samples, both after 30 and 90 min of incubation (Fig. 2). The unexpected outlier position of GBS gene transcript data from donor A blood was confirmed by a second set of microarray data (data not shown).

### Peculiar whole GBS transcriptional signature during incubation with donor A blood

We previously showed that microarray data obtained from seven donors were highly similar to one another [13]. Using the microarray data obtained from these seven samples, we calculated the average transcript level for each gene at each time point (i.e., immediately after mixing bacteria with donor human blood, and after 30 and 90 min of incubation) for the 1,995 ORFs present on the chip [13]. Differences in gene transcripts were determined by comparing the average value from one time point to another. An ORF was considered to be differentially expressed if there was a “present” signal at a minimum of one time point, if there was a significant (*P*<0.05, T-test) change in expression greater than 2-fold or lower than 0.5 at one time point relative to another, and if these criteria were met for at least six of the seven donor samples [13]. Similarly, we compared GBS gene expression in the donor A blood sample after 30 and 90 min of incubation to gene expression immediately after mixing the bacteria with the human blood. Our analysis was based on the comparison of GBS gene expression in the donor A blood sample and in the seven other donor blood samples.

The expression data revealed that extensive remodelling of the GBS transcript profile occurred during incubation of the bacterial strain in each blood sample. Importantly, we identified a large difference between the donor A blood sample data and data for the other seven blood samples [13]. For example, after 30 min incubation in donor A blood, transcripts of only 11.1% of the ORFs present on the chip changed significantly, whereas transcripts of 39.6% of the ORFs in the other donors' blood were significantly altered: 160 transcripts were up-regulated and 62 were down-regulated in donor A blood, whereas 134 transcripts were up-regulated and 658 were down-regulated in the other blood samples (Table 1). After 90 min incubation, 32% of the transcripts changed in donor A blood whereas 31.7% of the transcripts were modified in the other donors' blood: 270 transcripts were up-regulated and 369 were down-regulated in donor A blood, whereas 115 transcripts were up-regulated and 518 were down-regulated in the other blood samples (Table 1).

**Table 1:** Number (and percentage) of GBS transcripts significantly up- and down-regulated in donor A and the seven other donor blood samples according to the duration of the incubation at 37°C.

	Donor	A	Average	7 donors
	After 30 minutes	After 90 minutes	After 30 minutes	After 90 minutes
	(time 1)	(time 2)	(time 1)	(time 2)
Up-regulation a	160 (72.0%)	270 (42.2%)	134 (16.9%)	115 (18.2%)
Down-regulation a	62 (28.0%)	369 (57.8%)	658 (83.1%)	518 (81.8%)
Total b	222 (11.1%)	639 (32.0%)	792 (39.6%)	633 (31.7%)

An ORF is considered differentially expressed if there is a significant (*P*<0.05, T-test) change in expression greater than 2-fold at one time point relative to another, and if the signal is detected. Up- and down-regulation are expressed relative to time 0.

a Percentage is expressed relative to the number of genes whose expression is modified.

b Percentage is expressed relative to the total number of genes present on the chip.

In summary, far fewer transcripts changed significantly after 30 min incubation in donor A blood relative to other blood samples, and the changes mainly involved gene up-regulation. Gene expression changed after 90 min incubation relative to time 0 for a similar number of genes in donor blood A and the other blood samples, but more genes were up-regulated in donor A blood.

**Figure 3:**
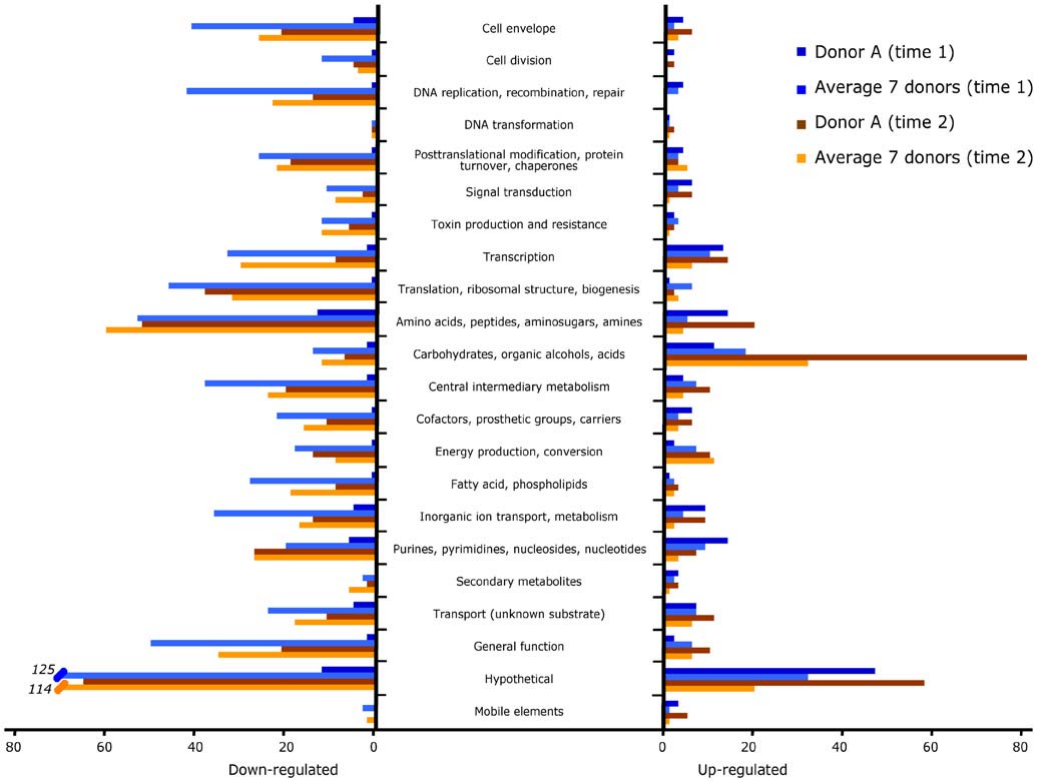
Differential regulation of transcript expression in GBS strain NEM316 after incubation with human blood. Genes were classified into 22 main functional categories. Bars indicate the numbers of genes whose expression was modified in the donor A blood sample and the average of the seven other donor blood samples at time 1 and time 2 relative to time 0. On the left, genes that were down-regulated relative to time 0. After 30 min of incubation, 62 transcripts were down-regulated in the donor A blood sample, while 658 transcripts were down-regulated in the other donor blood samples. After 90 min of incubation, 369 transcripts were down-regulated in the donor A blood sample, while 518 transcripts were down-regulated in the other donor blood samples. On the right, genes that were up-regulated relative to time 0. After 30 min of incubation, 160 transcripts were up-regulated in the donor A blood sample, while 134 transcripts were up-regulated in the other donor blood samples. After 90 min of incubation, 270 transcripts were up-regulated in the donor A blood sample, while 115 transcripts were up-regulated in the other donor blood samples.

Of note, the difference in gene up-regulation between donor A and other blood samples included many genes involved in amino acid, purine-pyrimidine, and central intermediary metabolism, and the most notable difference concerned genes contributing to carbohydrate metabolism (Fig. 3, and see below).

### Differential levels of GBS gene expression in donor A blood sample

**Figure 4:**
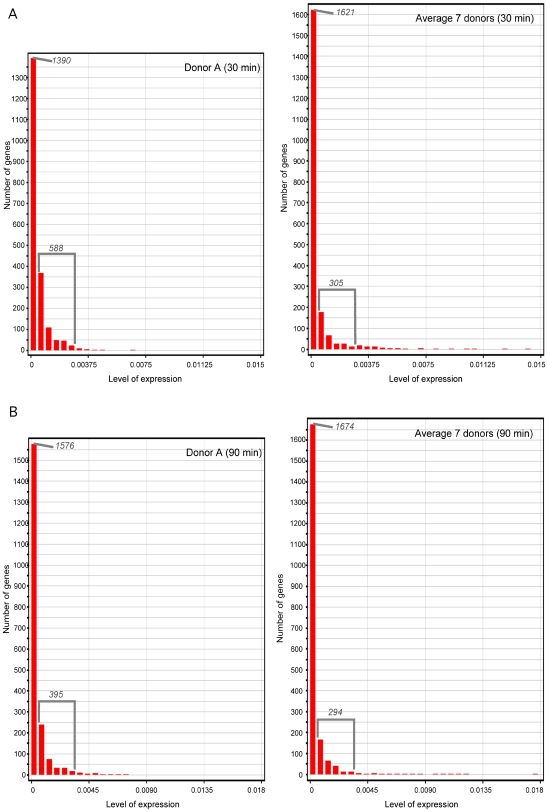
Level of GBS strain NEM316 gene expression in donor A blood and other donor blood samples. Levels of expression were categorized arbitrarily as: (i) expressed at very low level or not expressed, (ii) “normally” expressed, and (iii) highly expressed (form left to right, first column following five columns, and last 15 columns, respectively). After 30 min of incubation, 1,390, 588 and no ORFs were categorized as mentioned above in donor A blood, while there were 1,621, 305 and 6 ORFs in the other blood samples. After 90 min of incubation, 1,576, 395 and no ORFs were categorized as mentioned above in donor A blood, while there were 1,674, 294 and 6 ORFs in the other blood samples.

To further dissect the difference of the GBS transcriptome in the donor A blood sample relative to the other donor blood samples, we categorized the 1,995 genes according to their level of expression. We observed unusual features of expression of genes after 30 min of incubation: (i) fewer genes were expressed at a very low level (or not expressed) in the donor A blood sample than in the other blood samples (i.e., 1,390 and 1,621 ORFs, respectively), (ii) more genes were expressed at a higher level in the donor A blood sample than in the other blood samples (i.e. 588 and 305 ORFs, respectively), and (iii) no genes were expressed at a very high level in the donor A blood sample to the difference of other blood samples (Fig. 4A).

Although of smaller magnitude, we observed a similar difference of levels of gene expression between the donor A blood sample and other donor blood samples after 90 min of incubation (Fig. 4B).

### Difference in transcripts of genes involved in carbohydrate metabolism

**Figure 5:**
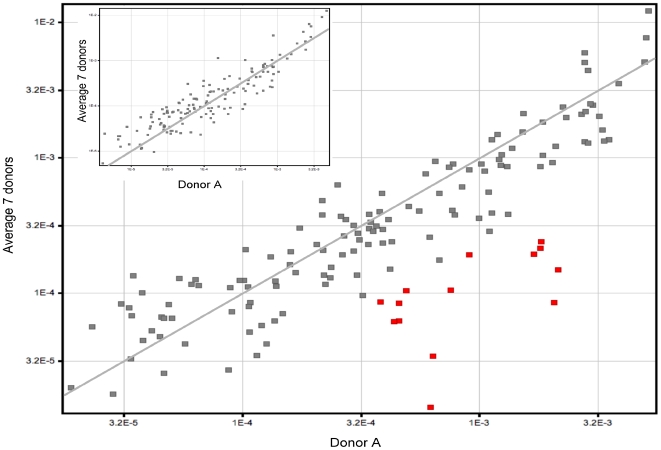
Differential expression of genes involved in carbohydrate metabolism in GBS strain NEM316. All of the 14 red plots mark genes involved in carbohydrate metabolism expressed at higher levels in donor A blood (axis) relative to the other blood samples (ordinate) after 90 min incubation. For comparison, small figure on the top left represents gene expression after 30 min incubation.

Within all functional categories of genes involved in bacterial metabolism, the most notable difference between the data from donor A blood and other donor blood samples was expression of genes involved in carbohydrate metabolism. Indeed, after 30 min of incubation, 11 and 18 ORFs related to carbohydrate metabolism were up-regulated in donor A and the other seven donor blood samples, respectively; after 90 min incubation, 81 and 32 ORFs were up-regulated relative to time 0 in donor A blood and other donor blood samples, respectively. Genes encoding a galactose PTS (gbs1920–gbs1922) and a galactose-phosphate-isomerase (gbs1917–gbs1918) were among the highest transcripts in donor A blood relative to the other donors' blood (Fig. 5). Similarly, other genes encoding proteins involved in carbohydrate binding, transport, and metabolism were (gbs0032, gbs0033–gbs0035, gbs1416–gbs1417, gbs1914, gbs1916) also expressed at a higher level in donor A blood (Fig. 5 and table S1). These results highlight a specific difference in regulation of genes involved in carbohydrate metabolism in donor blood A relative to the other donors, suggesting different metabolic conditions in this specific sample and/or a putative different innate or acquired immune response of the host.

### Later expression of genes involved in stress response in donor A blood

After 30 min of incubation, the transcripts of gbs1721 (encoding a general stress protein), gbs1202 and gbs1204 (encoding proteins belonging to the Gls24 stress family), and gbs0808 (encoding a superoxide dismutase) were 5.8-, 2.0- and 2.6-fold higher in the seven blood samples than in the donor A blood sample, respectively (Table S1). A similar observation was made after 90 min of incubation. Three of these genes were also within the most expressed ORFs in donor A blood after 90 min, whereas there were no stress genes among the 20 most expressed ORFs after 30 min. Taken together, these results show a lower and later GBS stress response in donor A blood than in other blood samples.

### Complex kinetics of transcripts of proven and putative virulence genes

After 30 min of incubation in blood, transcripts of three genes encoding proteins with an LPXTG motif were present at higher levels in the seven blood samples than in the donor A blood sample: transcripts of gbs1087 (encoding the FbsA fibrinogen binding protein), gbs2018 (encoding the BibA protein), and gbs1420 (encoding another LPXTG motif protein) were 1.5-, 4.2- and 1.45-fold higher in the seven blood samples, respectively. Streptococcal proteins with an LPXTG motif generally are secreted, covalently tethered to the bacterial cell-surface, and involved in virulence. However, after 90 min of incubation, transcripts of gbs1087, gbs2018, and gbs1420 were higher in donor A blood than in the other blood samples (Table S1). Another gene putatively involved in virulence, gbs1195 (encoding a streptokinase-like protein), was also expressed at a higher level in the 7 blood donors after 30 min and at a higher level in donor A blood after 90 min of incubation. Interestingly, gbs1087, gbs2018 and gbs1195 were also within the 12 highest expressed genes in donor A blood after 90 min of incubation.

Among other proven or putative GBS virulence genes, we observed other distinct features of the transcript kinetics. For example, most of the genes encoding enzymes involved in synthesis of the sialylated capsular polysaccharide (gbs1233–gbs1247), one of the most important GBS virulence factors, and the group B antigen (gbs1480–gbs1493), another major cell wall polysaccharide [12], were expressed at higher levels in donor A blood relative to the other blood samples after 30 min of incubation. Similar kinetics were observed for gbs0456, encoding a protein with an LPXTS motif, which was 22- and 6.6-fold more expressed in donor A blood than in the other blood samples at 30 min and 90 min, respectively.

We also observed that after 30 min and 90 min of incubation, gbs1053-gbs1076 were expressed from 2.2- to 18.5-fold higher in donor A blood than in the seven other donors, and also after 90 min of incubation. Interestingly, this chromosomal region corresponds to part of pathogenicity island IX. Although this region of the chromosome contains mostly genes encoding proteins of unknown function [14], it was recently shown that genes of pathogenicity island IX were up-regulated at temperatures approximating the human body compared to a lower temperature more presentative of environmental conditions, suggesting a role in virulence [15].

### Unexpected characteristics of cytokine production in donor A blood relative to the other blood samples

To begin to understand the significant difference between the GBS transcriptome in donor A blood and the other donor blood samples, we measured cytokine levels in blood immediately before contact with GBS and after 90 min of incubation with the bacteria. Despite inter-individual variability among blood samples, at time 0 as well as at time 2 (90 min), there was no specific difference in the level of cytokines (for most of the cytokines tested) in donor A blood relative to the seven other blood samples (Table S2).

**Figure 6:**
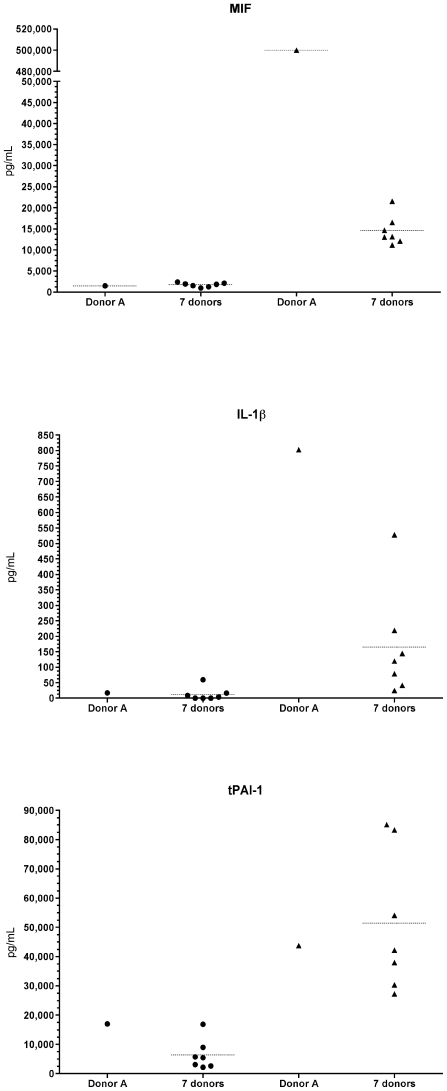
Cytokine production in the eight blood donor samples. Cytokine concentrations were measured using the xMAP technology combining a sandwich immunoassay with fluorescent bead-based technology. Cytokines were measured immediately before contact with GBS (left of each figure) and after 90 min of incubation with the bacteria (right of each figure). tPAI-1 was higher in donor A blood relative to the other blood samples at time 0, while MIF and IL-1β were higher in donor A blood than in the other blood samples after 90 min. Cytokine concentrations are expressed in pg/ml.

However, after 90 min, IL-1β was higher in donor A blood and, notably, MIF was 23- to 44-fold higher in donor A blood than in the other samples, whereas there was no difference between donor A blood and the other samples at time 0 (Fig. 6). Even though the difference was of smaller magnitude, we also observed that at time 0 tPAI-1 in donor A blood was at the highest level (together with donor E) relative to the other blood samples (Fig. 6). Conversely, GM-CSF, IFN-γ, IL-7, IL-10, IL-12p40, MIP-1α and MIP-1β were present at a lower level in the donor A blood sample relative to the other blood samples after 90 min of incubation, whereas there was no difference between donor A blood and the other samples at time 0 (Fig. 6). Taken together, these results show an unexpected difference in the level of proinflammatory and immunomodulatory cytokines in donor A blood relative to the other blood samples. Certain cytokines were elevated predominantly after incubation with GBS, but before contact with GBS, suggesting a physiologic state distinct to this donor relative to the seven other subjects.

## Discussion

*Ex vivo* studies provide important advantages in mirroring conditions existing within infected humans [16]. A key finding from our study is that GBS grown in the blood of donor A had an unusual transcript pattern relative to the seven other blood donors, both after 30 min and 90 min of incubation. The most notable transcript differences were from genes involved in carbohydrate metabolism. Importantly, regulation of carbohydrate metabolism is crucial during in vitro growth and host colonization of group A *Streptococcus* [17], [18], [19], and was recently found to be a major feature of GBS growing in vitro in laboratory media [15]. Unexpectedly, the stress response of GBS grown in donor A blood occurred at a later time and was of decreased magnitude. This lead us to speculate that GBS encountered more favorable environmental conditions in donor A blood relative to in the other blood samples, allowing the bacterium to diversely regulate its transcriptome according to the metabolic status or the immune response of the host.

This latter hypothesis is based on epidemiological data showing that GBS infections occuring in non-pregnant adults mainly affect patients with a certain degree of immunosuppression and/or metabolic derangements such as diabetes [2]. Thus, in addition to the inability to accurately neutralize the infectious process, the host immune response can conduct to variation in the expression of virulence factors by the bacterium [20]. Indeed, some bacterial pathogens are able to substantially modify their surface components, as for example *Borrelia burgdorferi*, which changes its antigenic expression under the influence of immune response [21]. In GBS, hemolysin can be differentially expressed during infection in a single patient [22], [23], another phenotypic change occuring in response to the immune response of the host, consistent with differences in resistance to host phagocytic killing [22]. Thus, if the immune system can exert a selective pressure on the expression of virulence factors, it could as well influence the entire transcriptome itself, and consequently explain the difference between GBS genome expression in donor A blood and the other samples. It has to be noted that there is an inter-individual variability of cytokine production that we observed within our various samples, but such variability has already been observed both in *in vitro* conditions and during human infections [24], [25]. However, our results show an unusual immune response of the various cytokine types tested. Indeed, production of both pro-inflammatory (such as TNF-α, IFN-γ, IL-12, and MIP) and anti-inflammatory (such as IL-10) cytokines was significantly lower in donor A blood, whereas conversely, another pro-inflammatory cytokine, IL-1β, was produced at a higher level in donor A blood relative to the other blood samples.

Another hypothesis to explain the differences in bacterial transcriptomes between donor A blood and the other blood samples is linked to a peculiar metabolic status of the host. This is supported by more elevated levels of tPAI-1 and MIF in donor A blood. A high level of tPAI-1, which is the predominant inhibitor of the fibrinolytic system, leads to hypofibrinolysis with a lack of dissolution of microvascular fibrin depositions as a consequence [26]. Plasma concentration of tPAI-1 is influenced by genetic determinants, and among metabolic determinants, by insulin resistance and diabetes [27]. Furthermore, it has been shown that populations who are subject to develop diabetes after several years had higher levels of tPAI-1 than population who did not [28]. MIF is another pro-inflammatory cytokine constitutively expressed by various cells and tissues, and can be involved in multiple inflammatory reactions [29]. Similarly to tPAI-1, there is strong link between MIF production and type 2 diabetes [30], although it is not clear yet which disorder originates the other. Thus, high levels of both tPAI-1 and MIF might be consistent with a putative pre-diabetic status of donor A. Interestingly, and consistent with our results, MIF can be rapidly released from preformed pools in response to microbial stimulation [31], and high concentrations of MIF have been detected in the bloodstream of patients with severe sepsis [32]. Elevated levels of tPAI-1 are also positively related to poor outcomes and increased severity in patients suffering from meningococcal sepsis [33].

In summary, when analyzing the GBS expression during contact with human blood, we identified an unexpected bacterial expression in one blood sample relative to the other samples, and investigated the immune response of the hosts to clarify this point. Our results show a link between GBS gene expression and donor A immune response, either because donor A immune response directly influences the bacterial transcriptome, or alternatively, because both GBS gene expression and immune response in blood are influenced by a putative pre-diabetic status of donor A. Further studies are necessary to confirm either hypothesis in focusing analyses on donors with strictly defined underlying diseases such as diabetes.

## Materials and Methods

### Bacterial strain, human blood and growth conditions

The serotype III GBS strain NEM316 used for this study has been well characterized and its genome has been sequenced [12]. The organism was grown in Todd Hewitt broth supplemented with 0.2% yeast extract (THY) in 5% CO_2_ at 37°C until the OD_600_ reached 0.75. The bacteria were harvested by centrifugation for 8 min at 4000×g at 37°C, and the cell pellet was suspended in phosphate-buffered saline (PBS).

Fresh heparinized human blood was obtained from eight volunteers (4 males and 4 females, arbitrarily named A to H) in accordance with a protocol approved by the Institutional Review Board of The Methodist Hospital Research Institute (IRB Protocol # IRB0108-0005). All donors provided written informed consent for the collection of samples and subsequent analysis. The donors were not taking medications that would influence the transcriptome analysis, such as antimicrobial agents. Bacteria were mixed with 80 ml of whole blood from each donor (final GBS concentration of ∼5×10^8^ CFU/ml of blood) and incubated at 37°C with slight rotation to avoid sedimentation of blood and bacterial cells.

Samples for transcriptome analysis were removed immediately after mixing the bacteria with human blood, and after 30 and 90 min of incubation. Samples for cytokine analysis were removed before adding the bacteria to the blood and after 90 min of incubation.

### RNA isolation

Bacterial RNA was isolated from each sample as previously described [13]. The RNA concentration was evaluated by measuring absorbance at 260 and 280 nm, and RNA quality was evaluated by electrophoretic analysis with an Agilent 2100 Bioanalyzer (Agilent Technologies Inc., Palo Alto, CA).

### cDNA synthesis, fragmentation, and labelling

The methods used for cDNA synthesis, fragmentation, and labelling have been described extensively elsewhere [15].

### Expression microarray analysis

Expression microarray analysis was performed with a custom-made Affymetrix chip formulated based on the genome sequence of strain NEM316 [12]. The chip contains 1,995 probe sets corresponding to the annotated open reading frames (ORFs) in this genome. Briefly, end-labelled cDNA was hybridized overnight at 40°C using the Affymetrix hybridization and staining modules, according to the manufacturer's instructions. Chip hybridization data were acquired and normalized using Affymetrix GeneChip Operating Software (GCOS). Hybridization intensity values were normalized to the mean intensity of all GBS genes present on the chip using GCOS version 1.0 to permit comparison of data obtained from multiple experimental conditions. We compared GBS transcript levels in the donor A blood sample to the average of transcript levels in the seven other blood samples (donors B to H) [13].

The microarray data for this study have been deposited in the Gene Expression Omnibus database (GSE12201 for donor A blood sample and GSE11705 for donors B to H blood samples).

### Cytokine production

The concentration of various cytokines was measured using the xMAP technology (Luminex Corp., Austin, TX) which combines the principle of a sandwich immunoassay with fluorescent bead-based technology. The xMAP serum assay was performed according to the manufacturer's instructions for eotaxin, granulocyte colony-stimulating factor (G-CSF), granulocyte macrophage colony-stimulating factor (GM-CSF), interferon-α (IFN-α), interferon-γ (IFN-γ), interleukin-1α (IL-1α), interleukin-1β (IL-1β), interleukin-2 (IL-2), interleukin-3 (IL-3), interleukin-4 (IL-4), interleukin-5 (IL-5), interleukin-6 (IL-6), interleukin-7 (IL-7), interleukin-8 (IL-8), interleukin-10 (IL-10), interleukin-12p40 (IL-12p40), interleukin-12p70 (IL-12p70), interleukin-13 (IL-13), interleukin-15 (IL-15), interleukin-17 (IL-17), interferon-gamma inducible protein-10 (IP-10), monocyte chemoattractant protein-1 (MCP-1), macrophage inflammatory protein-1α (MIP-1α), macrophage inflammatory protein-1β (MIP-1β), tumor necrosis factor-α (TNF-α), tumor necrosis factor-β (TNF-β), macrophage inhibitor factor (MIF), and plasminogen activator inhibitor type-1 (tPAI-1). Experiments were performed in duplicate and samples were diluted appropriately when necessary.

### Bioinformatic analyses

Analyses, statistics, and graphics were performed with Partek Pro Genomics Suite 6.0 (Partek, St. Louis, MO), ArrayAssist software v5.5.1 (Stratagene, La Jolla, CA), and GraphPad Prism v4 (GraphPad Software Inc., San Diego, CA).

## Supporting Information

Table S1 Concentration of the cytokines before adding the bacteria to the blood samples and after 90 min of incubation at 37°C for the eight donors.
(0.04 MB PDF)

Table S2 Microarray expression data from GBS strain NEM316 during incubation with human blood at 37°C. GBS transcript levels in the donor A blood sample were compared to the average of transcript levels in the seven other blood samples (donors B to H) after 30 min (Time 1) and 90 min (Time 2) of incubation. Only ratios greater than 2 and less than 0.5 were considered for analysis, according to the absent (A), present (P) or marginal (M) level of transcription in each sample.
(0.59 MB PDF)
