# Supplementary material for: Identification of an Unusual Pattern of Global Gene Expression in Group B Streptococcus Grown in Human Blood
Source: PLoS One. 2009 Sep 23;4(9):e7145. doi: 10.1371/journal.pone.0007145 (PMC2745576; doi:10.1371/journal.pone.0007145)
Supplement: Table S1 — Concentration of the cytokines before adding the bacteria to the blood samples and after 90 min of incubation at 37°C for the eight donors. (0.04 MB PDF) [file pone.0007145.s001.pdf]

Sample	**MIF**	**tPAI-1**	**Eotaxin**	**G-CSF**	**GM-CSF**	**IFN-α**	**IFN-γ**	**IL-1α**	**IL-1β**	**IL-2**	**IL-3**	**IL-4**	**IL-5**	**IL-6**	**IL-7**
Donor A (T0)	1 495	16 999	265	17	0	0	0	32	17	51	0	0	0	12	0
Donor B (T0)	2 407	3 097	392	28	1 458	2 923	8	249	16	4	20	452	26	20	340
Donor C (T0)	1 856	5 478	446	20	352	653	32	232	4	33	8	43	0	57	20
Donor D (T0)	1 299	2 159	333	0	54	212	0	93	0	0	0	0	0	5	0
Donor E (T0)	1 991	16 856	171	13	168	1 501	62	1 152	60	41	22	0	41	179	153
Donor F (T0)	2 139	5 725	572	30	94	977	9	243	9	0	22	0	9	28	54
Donor G (T0)	1 045	2 622	39	61	241	452	7	75	0	0	0	84	0	0	121
Donor H (T0)	1 570	9 058	98	21	0	0	22	1 025	0	0	0	0	0	0	0
Donor A (T2)	500 000	43 791	262	9	0	0	0	82	803	0	0	0	0	310	0
Donor B (T2)	13 092	27 242	524	32	546	655	15	182	528	0	6	38	0	2 296	70
Donor C (T2)	13 200	42 284	517	41	344	449	5	203	120	0	6	22	0	862	54
Donor D (T2)	21 536	37 966	125	15	147	423	27	878	25	0	10	0	8	482	68
Donor E (T2)	12 162	83 229	431	45	1 296	3 232	7	297	219	4	22	545	28	1 297	330
Donor F (T2)	11 199	30 335	671	31	232	1 041	3	275	145	0	25	0	9	324	102
Donor G (T2)	14 682	85 093	98	26	399	518	4	93	79	4	0	100	0	105	83
Donor H (T2)	16 528	54 127	142	31	356	0	18	624	42	0	0	0	0	116	18

IL-8	IL-10	IL-12p40	IL-12p70	IL-13	IL-15	IL-17	IP-10	MCP-1	MIP-1α	MIP-1β	TNF-α	TNF-β
5	0	0	0	0	12	0	339	295	30	179	5	0
58	165	419	0	38	13	10	372	484	96	92	8	19
18	19	105	13	19	5	33	358	372	246	371	4	4
3	4	0	0	0	0	0	336	306	0	43	0	0
31	115	2 035	31	238	68	50	2 056	312	210	239	5	134
16	26	554	6	53	13	12	223	238	103	90	4	20
8	10	40	0	0	3	10	240	156	51	61	0	0
4	0	51	59	0	0	20	69	225	55	278	4	0
737	0	0	0	0	0	0	237	530	888	0	294	0
2 411	19	120	5	6	5	9	373	512	5 406	3 864	1 969	4
2 296	12	217	5	14	5	3	363	552	4 802	2 234	1 281	5
401	40	508	0	66	11	13	3 085	369	1 183	296	390	33
2 704	185	481	0	43	14	6	415	790	2 399	1 259	2 067	21
2 657	30	530	0	46	13	0	238	458	4 913	1 546	1 740	25
2 631	13	46	0	0	4	3	259	171	3 931	1 091	897	0
2 744	4	60	23	0	3	16	53	425	3 468	278	1 765	0
