# Supplementary material for: Identification of an Unusual Pattern of Global Gene Expression in Group B Streptococcus Grown in Human Blood
Source: PLoS One. 2009 Sep 23;4(9):e7145. doi: 10.1371/journal.pone.0007145 (PMC2745576; doi:10.1371/journal.pone.0007145)
Supplement: Table S2 — Microarray expression data from GBS strain NEM316 during incubation with human blood at 37°C. GBS transcript levels in the donor A blood sample were compared to the average of transcript levels in the seven other blood samples (donors B to H) after 30 min (Time 1) and 90 min (Time 2) of incubation. Only ratios greater than 2 and less than 0.5 were considered for analysis, according to the absent (A), present (P) or marginal (M) level of transcription in each sample. (0.59 MB PDF) [file pone.0007145.s002.pdf]

NCBI ID	COG(s)	Locus	Gene	Functional category	Subcategory	Putative function	A vs 7d (T1)	A	B	C	D	E	F	G	H	A vs 7d (T2)	A	B	C	D	E	F	G	H
1029804	COG0593L	gbs0001	dnaA	Cellular processes	DNA replication, recombination and repair	Chromosomal replication initiator protein dnaA	2,315	P	P	P	P	P	P	P	P	1,780	P	P	P	P	P	P	P	P
1029808	COG0592L	gbs0002	dnaN	Cellular processes	DNA replication, recombination and repair	DNA polymerase III, beta chain (EC 2.7.7.7)	1,806	P	P	P	P	P	P	P	P	1,579	P	P	P	P	P	P	P	P
1029799	COG1597IR	gbs0003		Hypothetical		Hypothetical protein	2,367	P	A	P	M	P	P	M	A	1,517	P	A	A	M	A	A	A	A
1029797	-	gbs0004		Hypothetical		Hypothetical cytosolic protein	2,072	P	A	A	A	A	A	A	A	2,088	P	A	A	A	A	A	A	A
1029795	-	gbs0005		Hypothetical		Hypothetical protein	2,043	P	P	P	P	P	P	P	P	1,930	P	A	P	P	A	P	A	P
1030675	COG0012J	gbs0006	ychF	Cellular processes	Translation, ribosomal structure and biogenesis	GTP-binding protein, probable translation factor	1,744	P	P	P	P	P	P	P	P	0,759	P	P	P	P	P	P	P	P
1030987	-	gbs0007	pth	Cellular processes	Translation, ribosomal structure and biogenesis	Peptidyl-tRNA hydrolase (EC 3.1.1.29)	7,592	M	A	A	A	A	A	A	A	0,205	A	A	A	A	A	A	A	A
1029794	COG1197LK	gbs0008	trcF	Cellular processes	DNA replication, recombination and repair	Transcription-repair coupling factor	5,373	A	A	A	A	A	A	A	A	0,675	A	A	A	A	A	A	A	A
1029790	COG1188J	gbs0009	HSP15	Cellular processes	Translation, ribosomal structure and biogenesis	Heat shock protein 15	1,616	P	P	P	P	P	P	P	P	1,256	P	A	M	A	A	A	A	A
1029787	COG2919D	gbs0010	divIC	Cellular processes	Cell division	Cell division protein DIVIC	1,876	P	P	P	A	P	P	P	P	0,885	P	A	P	P	P	P	A	P
1030424	-	gbs0011		Hypothetical		Hypothetical protein	3,462	P	P	P	P	P	P	P	P	1,608	P	A	P	A	P	P	A	A
1029782	COG2367V	gbs0012		Cellular processes	Toxin production and resistance	Beta-lactamase (EC 3.5.2.6)	2,410	P	P	P	M	P	P	P	P	0,890	P	A	A	A	A	P	A	A
1029802	COG0037D	gbs0013	tilS	Cellular processes	Translation, ribosomal structure and biogenesis	tRNA(Ile)-lysidine synthetase TilS	2,234	P	A	P	A	P	P	A	A	0,662	P	A	P	A	A	P	M	A
1030991	COG0634F	gbs0014	hpt	Metabolism and transport	Purines, pyrimidines, nucleosides, and nucleotides	Hypoxanthine-guanine phosphoribosyltransferase (EC 2.4.2.8)	1,717	P	P	P	P	P	P	P	P	1,275	P	P	P	P	P	P	P	P
1030977	COG0465O	gbs0015	ftsH	Cellular processes	Posttranslational modification, protein turnover, chaperones	Cell division protein ftsH (EC 3.4.24.-)	1,549	P	P	P	P	P	P	P	P	1,524	P	A	P	A	P	P	P	P
1031481	3883S, COG3	gbs0016	pcsB	Cell Envelope	Other	Glucan-binding protein B	1,454	P	P	P	P	P	P	P	P	0,773	P	A	P	P	P	P	P	P
1031120	COG0462FE	gbs0017	prsA.2	Metabolism and transport	Purines, pyrimidines, nucleosides, and nucleotides	Ribose-phosphate pyrophosphokinase (EC 2.7.6.1)	2,160	P	P	P	P	P	P	P	P	1,270	P	P	P	P	P	P	P	P
1031047	COG0436E	gbs0018	aspB	Metabolism and transport	Amino acids, peptides, aminosugars and amines	Aromatic amino acid aminotransferase (EC 2.6.1.57) Acetyldiaminopimelate amin	1,417	P	P	P	P	P	P	P	P	1,684	P	A	P	M	P	P	P	P
1030999	COG1381L	gbs0019	recO	Cellular processes	DNA replication, recombination and repair	DNA repair protein recO	2,436	P	P	P	P	P	P	P	P	1,477	P	A	P	M	M	P	P	P
1031000	-	gbs0020		Cellular processes	Posttranslational modification, protein turnover, chaperones	CAAX amino terminal protease family	0,248	A	A	A	A	A	A	A	P	0,145	A	P	A	A	A	A	A	A
1031476	COG0416I	gbs0021	plsX	Metabolism and transport	Fatty acid and phospholipids	Fatty acid phospholipid synthesis protein plsX	2,831	P	P	P	P	P	P	P	P	1,619	P	A	P	P	P	P	A	P
1031477	COG0236IQ	gbs0022	acpP.2	Metabolism and transport	Fatty acid and phospholipids	Acyl carrier protein	2,238	P	P	P	P	P	P	P	P	1,257	P	A	P	A	A	P	P	A
1031217	-	gbs0023	purC	Metabolism and transport	Purines, pyrimidines, nucleosides, and nucleotides	Phosphoribosylaminoimidazole-succinocarboxamide synthase (EC 6.3.2.6)	1,620	P	P	P	P	P	P	P	P	0,476	A	A	A	A	A	P	A	A
1030849	30046F, COG0	gbs0024		Metabolism and transport	Purines, pyrimidines, nucleosides, and nucleotides	Phosphoribosylformylglycinamidine synthase (EC 6.3.5.3)	0,995	P	P	P	P	P	P	P	P	0,619	P	P	P	P	P	P	M	P
1031315	COG0034F	gbs0025	purF	Metabolism and transport	Purines, pyrimidines, nucleosides, and nucleotides	Amidophosphoribosyltransferase (EC 2.4.2.14)	1,502	P	P	P	P	P	P	P	P	1,845	P	A	A	A	A	P	A	A
1029830	COG0150F	gbs0026	purM	Metabolism and transport	Purines, pyrimidines, nucleosides, and nucleotides	Phosphoribosylformylglycinamidine cyclo-ligase (EC 6.3.3.1)	0,989	P	P	P	P	P	P	P	P	0,845	P	M	P	P	P	P	P	P
1030771	-	gbs0027	purN	Metabolism and transport	Purines, pyrimidines, nucleosides, and nucleotides	Phosphoribosylglycinamide formyltransferase (EC 2.1.2.2)	1,741	P	M	P	P	P	P	P	P	2,252	P	A	A	A	A	A	A	A
1031482	-	gbs0028		Hypothetical		Zwittermicin A resistance protein zmaR	1,920	P	P	P	P	P	P	P	P	3,580	P	P	M	A	A	P	A	A
1031413	-	gbs0029	purH	Metabolism and transport	Purines, pyrimidines, nucleosides, and nucleotides	Phosphoribosylaminoimidazolecarboxamide formyltransferase (EC 2.1.2.3) IMP c	1,145	P	P	P	P	P	P	P	P	1,761	P	A	P	A	P	P	P	P
1031034	COG0739M	gbs0030		Cellular processes	Posttranslational modification, protein turnover, chaperones	Peptidoglycan-specific endopeptidase, M23 family	1,373	P	A	A	A	A	A	A	A	0,083	A	A	A	A	A	A	A	A
1031483	-	gbs0031	sip	Hypothetical		Surface antigen	1,099	P	P	P	P	P	P	P	P	0,853	P	P	P	P	P	P	P	P
1029831	COG3010G	gbs0032		Metabolism and transport	Carbohydrates, organic alcohols, and acids	N-acetylmannosamine-6-phosphate 2-epimerase (EC 5.1.3.9)	0,612	P	A	P	A	A	A	A	A	8,317	P	P	P	P	P	P	P	P
1030981	COG1653G	gbs0033		Metabolism and transport	Carbohydrates, organic alcohols, and acids	N-acetylneuraminate-binding protein	0,803	A	A	A	A	A	A	A	A	7,517	P	A	P	A	P	M	A	A
1029979	COG1175G	gbs0034		Metabolism and transport	Carbohydrates, organic alcohols, and acids	N-acetylneuraminate transport system permease protein	0,658	A	A	A	A	A	A	A	A	7,252	P	A	M	A	A	A	A	A
1031470	COG0395G	gbs0035		Metabolism and transport	Carbohydrates, organic alcohols, and acids	N-acetylneuraminate transport system permease protein	0,176	A	A	A	A	A	A	A	A	4,372	P	A	P	A	A	A	A	A
1031485	-	gbs0036		Hypothetical		Hypothetical cytosolic protein	0,420	A	A	M	A	A	A	A	A	4,450	P	A	P	A	P	P	A	A
1031486	-	gbs0037		Hypothetical		Hypothetical membrane spanning protein	2,058	A	A	A	A	A	A	A	A	2,842	P	A	P	A	A	P	A	A
1030355	COG0329EM	gbs0038	nanH	Cell Envelope	Biosynthesis and degradation of surface polysaccharides and lipopo	N-acetylneuraminate lyase (EC 4.1.3.3)	0,243	A	A	A	A	A	A	A	A	3,891	P	A	A	A	A	A	A	A
1030984	COG1940KG	gbs0039		Cellular processes	Signal transduction	N-acetylmannosamine kinase (EC 2.7.1.60) Transcriptional regulator	0,727	A	A	A	A	A	A	A	A	1,828	P	A	A	A	A	A	A	A
1031495	COG3458Q	gbs0040		Metabolism and transport	Secondary metabolites	Cephalosporin-C deacetylase (EC 3.1.1.41)	0,658	A	A	A	A	A	A	A	A	1,542	P	A	M	A	A	A	A	A
1030478	COG1737K	gbs0041		Cellular processes	Transcription	Transcriptional regulator, RpiR family	2,221	P	P	P	A	P	P	P	P	1,641	P	P	P	P	P	P	P	P
1031720	COG0151F	gbs0042	purD	Metabolism and transport	Purines, pyrimidines, nucleosides, and nucleotides	Phosphoribosylamine--glycine ligase (EC 6.3.4.13)	0,535	P	P	P	P	P	P	P	P	0,302	A	A	M	A	A	M	P	A
1030779	COG0041F	gbs0043	purE	Metabolism and transport	Purines, pyrimidines, nucleosides, and nucleotides	Phosphoribosylaminoimidazole carboxylase carboxyltransferase subunit (EC 4.1.1	0,670	P	P	P	P	P	P	P	P	0,495	P	A	A	A	A	P	M	M
1030983	COG0026F	gbs0044	purK	Metabolism and transport	Purines, pyrimidines, nucleosides, and nucleotides	Phosphoribosylaminoimidazole carboxylase NCAIR mutase subunit (EC 4.1.1.21)	0,611	P	P	P	P	P	P	P	P	0,561	P	A	M	A	A	P	A	M
1030998	-	gbs0045		Hypothetical		Hypothetical protein	0,847	P	P	P	P	P	P	P	P	1,526	P	P	P	P	P	P	P	P
1030996	-	gbs0046		Hypothetical		Hypothetical protein	0,869	P	P	P	P	P	P	P	P	1,559	P	P	P	P	P	P	P	P
1030994	COG0015F	gbs0047	purB	Metabolism and transport	Purines, pyrimidines, nucleosides, and nucleotides	Adenylosuccinate lyase (EC 4.3.2.2)	0,787	P	P	P	P	P	P	P	P	0,945	P	P	P	P	P	P	P	P
1030979	-	gbs0048		Cellular processes	Transcription	Transcriptional regulator	1,625	P	P	P	P	P	P	P	P	1,978	P	A	P	P	P	P	A	M
1030989	COG2255L	gbs0049	ruvB	Cellular processes	DNA replication, recombination and repair	Holliday junction DNA helicase ruvB	1,175	P	A	A	A	A	A	A	A	0,847	P	A	A	A	A	A	A	A
1030982	COG0394T	gbs0050		Cellular processes	Signal transduction	Protein tyrosine phosphatase (EC 3.1.3.48)	0,940	P	P	P	P	P	P	P	P	1,256	P	P	P	P	P	P	P	M
1030978	COG4642S	gbs0051		Hypothetical		Hypothetical membrane associated protein	1,199	P	P	P	P	P	P	P	P	2,411	P	A	P	A	A	M	A	A
1030985	COG1835I	gbs0052		Metabolism and transport	Fatty acid and phospholipids	Acyltransferase family	1,648	P	P	P	P	P	P	P	P	1,596	P	P	P	P	P	P	P	P
1030824	1012C, COG1	gbs0053	adh2	Metabolism and transport	Energy production and conversion	Alcohol dehydrogenase (EC 1.1.1.1) Acetaldehyde dehydrogenase [acetylating] (	0,660	P	A	A	P	P	P	P	A	0,346	P	P	P	A	P	P	A	A
1030992	COG1064R	gbs0054	adhA	Metabolism and transport	Energy production and conversion	Alcohol dehydrogenase (EC 1.1.1.1)	0,360	P	A	P	M	P	P	P	P	0,530	P	P	P	P	P	P	P	P
1031381	COG0498E	gbs0055	thrC	Metabolism and transport	Amino acids, peptides, aminosugars and amines	Threonine synthase (EC 4.2.3.1)	2,542	P	A	P	A	A	M	A	M	1,673	P	A	A	A	A	A	A	A
1030757	COG0534V	gbs0056		Cellular processes	Toxin production and resistance	Na+ driven multidrug efflux pump	1,777	P	M	P	P	P	P	P	P	0,628	P	P	P	P	P	P	P	P
1030755	COG0051J	gbs0057	rpsJ	Cellular processes	Translation, ribosomal structure and biogenesis	SSU ribosomal protein S10P	1,241	P	P	P	P	P	P	P	P	0,682	P	P	P	A	P	P	P	P
1030743	-	gbs0058	rplC	Cellular processes	Translation, ribosomal structure and biogenesis	LSU ribosomal protein L3P	1,038	P	P	P	P	P	P	P	P	0,840	P	P	P	P	P	P	P	P
1031368	-	gbs0059	rplD	Cellular processes	Translation, ribosomal structure and biogenesis	LSU ribosomal protein L1E (= L4P)	0,892	P	P	P	P	P	P	P	P	0,911	P	P	P	P	P	P	P	P
1030756	COG0089J	gbs0060	rplW	Cellular processes	Translation, ribosomal structure and biogenesis	LSU ribosomal protein L23P	0,837	P	P	P	P	P	P	P	P	1,063	P	P	P	P	P	P	P	P
1030731	COG0090J	gbs0061	rplB	Cellular processes	Translation, ribosomal structure and biogenesis	LSU ribosomal protein L2P	0,925	P	P	P	P	P	P	P	P	1,105	P	P	P	P	P	P	P	P
1030738	COG0185J	gbs0062	rpsS	Cellular processes	Translation, ribosomal structure and biogenesis	SSU ribosomal protein S19P	0,752	P	P	P	P	P	P	P	P	1,040	P	P	P	P	P	P	P	P
1030594	-	gbs0063	rplV	Cellular processes	Translation, ribosomal structure and biogenesis	LSU ribosomal protein L22P	0,750	P	P	P	P	P	P	P	P	1,046	P	P	P	P	P	P	P	P
1031362	COG0092J	gbs0064	rpsC	Cellular processes	Translation, ribosomal structure and biogenesis	SSU ribosomal protein S3P	0,629	P	P	P	P	P	P	P	P	1,139	P	P	P	P	P	P	P	P
1031364	COG0197J	gbs0065	rplP	Cellular processes	Translation, ribosomal structure and biogenesis	LSU ribosomal protein L16P	0,690	P	P	P	P	P	P	P	P	1,215	P	P	P	P	P	P	P	P
1031365	-	gbs0066	rpmC	Cellular processes	Translation, ribosomal structure and biogenesis	LSU ribosomal protein L29P	0,673	P	P	P	P	P	P	P	P	1,253	P	P	P	P	P	P	P	P
1031359	COG0186J	gbs0067	rpsQ	Cellular processes	Translation, ribosomal structure and biogenesis	SSU ribosomal protein S17P	0,827	P	P	P	P	P	P	P	P	1,572	P	P	P	P	P	P	P	P
1031447	-	gbs0068	rplN	Cellular processes	Translation, ribosomal structure and biogenesis	LSU ribosomal protein L14P	0,629	P	P	P	P	P	P	P	P	1,513	P	P	P	P	P	P	P	P
1030496	COG0198J	gbs0069	rplX	Cellular processes	Translation, ribosomal structure and biogenesis	LSU ribosomal protein L24P	0,598	P	P	P	P	P	P	P	P	1,304	P	P	P	P	P	P	P	P
1031361	COG0094J	gbs0070	rplE	Cellular processes	Translation, ribosomal structure and biogenesis	LSU ribosomal protein L5P	0,652	P	P	P	P	P	P	P	P	1,490	P	P	P	P	P	P	P	P
1031357	COG0199J	gbs0071	rpsN	Cellular processes	Translation, ribosomal structure and biogenesis	SSU ribosomal protein S14P	0,650	P	P	P	P	P	P	P	P	1,553	P	P	P	P	P	P	P	P
1030668	-	gbs0072	rpsH	Cellular processes	Translation, ribosomal structure and biogenesis	SSU ribosomal protein S8P	0,674	P	P	P	P	P	P	P	P	1,670	P	P	P	P	P	P	P	P
1031356	COG0097J	gbs0073	rplF	Cellular processes	Translation, ribosomal structure and biogenesis	LSU ribosomal protein L6P	0,623	P	P	P	P	P	P	P	P	1,446	P	P	P	P	P	P	P	P
1030664	COG0256J	gbs0074	rplR	Cellular processes	Translation, ribosomal structure and biogenesis	LSU ribosomal protein L18P	0,607	P	P	P	P	P	P	P	P	1,299	P	P	P	M	P	P	P	P
1030663	COG0098J	gbs0075	rpsE	Cellular processes	Translation, ribosomal structure and biogenesis	SSU ribosomal protein S5P	0,601	P	P	P	P	P	P	P	P	1,454	P	P	P	P	P	P	P	P
1031353	COG1841J	gbs0076	rpmD	Cellular processes	Translation, ribosomal structure and biogenesis	LSU ribosomal protein L30P	0,645	P	P	P	P	P	P	P	P	1,874	P	P	P	P	P	P	P	P
1031349	COG0200J	gbs0077	rplO	Cellular processes	Translation, ribosomal structure and biogenesis	LSU ribosomal protein L15P	0,695	P	P	P	P	P	P	P	P	1,929	P	P	P	P	P	P	P	P
1030657	COG0201U	gbs0078	secY	Cellular processes	Posttranslational modification, protein turnover, chaperones	Protein translocase subunit secY	0,875	P	P	P	P	P	P	P	P	2,204	P	P	P	P	P	P	P	P
1030590	COG0563F	gbs0079	adk	Metabolism and transport	Purines, pyrimidines, nucleosides, and nucleotides	Adenylate kinase (EC 2.7.4.3) Nucleoside-diphosphate kinase (EC 2.7.4.6)	1,383	P	P	P	P	P	P	P	P	1,286	P	P	P	P	P	P	P	P
1031340	COG0361J	gbs0080	infA	Cellular processes	Translation, ribosomal structure and biogenesis	Bacterial Protein Translation Initiation Factor 1 (IF-1)	0,553	P	P	P	P	P	P	P	P	0,840	P	P	P	P	P	P	P	P
1031343	COG0257J	gbs0081	rpmJ	Cellular processes	Translation, ribosomal structure and biogenesis	LSU ribosomal protein L36P	0,498	P	P	P	P	P	P	P	P	0,770	P	P	P	P	P	P	P	P
1030659	COG0099J	gbs0082	rpsM	Cellular processes	Translation, ribosomal structure and biogenesis	SSU ribosomal protein S13P	0,526	P	P	P	P	P	P	P	P	0,903	P	P	P	P	P	P	P	P
1030596	COG0100J	gbs0083	rpsK	Cellular processes	Translation, ribosomal structure and biogenesis	SSU ribosomal protein S11P	0,662	P	P	P	P	P	P	P	P	0,871	P	P	P	P	P	P	P	P
1031335	COG0202K	gbs0084	rpoA	Cellular processes	Transcription	DNA-directed RNA polymerase alpha chain (EC 2.7.7.6)	0,736	P	P	P	P	P	P	P	P	1,049	P	P	P	P	P	P	P	P
1030588	COG0203J	gbs0085	rplQ	Cellular processes	Translation, ribosomal structure and biogenesis	LSU ribosomal protein L17P	1,042	P	P	P	P	P	P	P	P	1,144	P	P	P	P	P	P	P	P
1030554	-	gbs0086		Hypothetical		Hypothetical protein	1,748	A	A	A	A	A	A	A	A	0,152	A	A	A	A	A	A	A	A
1031450	-	gbs0087		Hypothetical		Hypothetical protein	1,838	M	A	A	A	A	A	A	A	0,076	A	A	A	A	A	A	A	A
1031323	-	gbs0088		Hypothetical		Hypothetical protein	0,889	P	A	A	P	P	A	A	A	2,045	P	A	A	A	A	A	A	A
1030498	-	gbs0089		Hypothetical		Hypothetical protein	0,631	P	P	P	P	P	P	P	P	2,427	P	P	P	P	P	P	P	P
1031446	-	gbs0090	comX1.1	Cellular processes	DNA transformation	Competence-specific sigma factor ComX	0,402	A	A	A	A	A	A	A	A	0,038	A	A	A	A	A	A	A	A
1031317	COG0406G	gbs0091		Metabolism and transport	Carbohydrates, organic alcohols, and acids	Phosphoglycerate mutase family protein	0,664	P	P	P	P	P	P	P	P	1,138	P	P	P	P	P	P	M	P
1030544	-	gbs0092		Cell Envelope	Biosynthesis and degradation of murein sacculus and peptidoglycan	D-alanyl-D-alanine metallocarboxypeptidase (EC 3.4.17.14)	1,248	P	P	P	P	P	P	P	P	1,830	P	A	P	A	M	P	P	A
1031431	COG1705NU	gbs0093		Cell Envelope	Biosynthesis and degradation of murein sacculus and peptidoglycan	N-acetylmuramoyl-L-alanine amidase (EC 3.5.1.28)	1,439	P	P	P	P	P	P	P	P	1,720	P	P	P	P	P	P	P	P
1030538	COG1420K	gbs0094	hrcA	Cellular processes	Transcription	Heat-inducible transcription repressor hrcA	3,689	P	P	P	P	P	P	P	P	1,207	P	P	P	P	P	P	P	P

1031176	COG0576O	gbs0095	grpE	Cellular processess	Posttranslational modification, protein turnover, chaperones	GrpE protein	2,288	P	A	A	P	P	P	P	P	1,072	P	A	P	A	A	P	A	P
1030530	COG0443O	gbs0096	dnaK	Cellular processess	Posttranslational modification, protein turnover, chaperones	Chaperone protein dnaK	1,067	P	P	P	P	P	P	P	P	1,155	P	P	P	P	P	P	P	P
1031312	COG0484O	gbs0097	dnaJ	Cellular processess	Posttranslational modification, protein turnover, chaperones	Chaperone protein dnaJ	1,775	P	P	P	P	P	P	P	P	2,023	P	A	P	P	P	P	P	P
1031314	COG1167KE	gbs0098		Cellular processess	Transcription	Transcriptional regulator, GntR family AMINOTRANSFERASE CLASS-I (EC 2.6.1	1,350	P	A	A	A	A	A	A	A	1,469	P	A	A	A	A	A	A	A
1031307	COG0101J	gbs0099	truA	Cellular processess	Translation, ribosomal structure and biogenesis	tRNA pseudouridine synthase A (EC 4.2.1.70)	4,285	P	P	P	P	P	P	P	P	1,806	P	P	P	P	P	P	P	P
1030485	COG0351H	gbs0100	thiD	Metabolism and transport	Cofactors, prosthetic groups, and carriers	Phosphomethylpyrimidine kinase (EC 2.7.4.7) Hydroxymethylpyrimidine kinase (E	5,498	P	P	P	P	P	P	P	P	2,847	P	P	P	P	P	P	P	P
1030521	COG4720S	gbs0101		Hypothetical		Hypothetical membrane spanning protein	6,003	P	P	P	P	P	P	A	P	3,238	P	P	P	P	P	P	A	P
1031311	-	gbs0102		Hypothetical		Hypothetical protein	8,244	P	A	P	A	P	P	A	A	2,485	P	P	P	P	P	P	P	P
1031306	COG0668M	gbs0103		General function predicted only		Mechanosensitive ion channel	2,060	P	A	A	A	A	A	A	A	1,540	A	A	A	A	A	A	A	A
1031436	COG0544O	gbs0104	tig	Cellular processess	Posttranslational modification, protein turnover, chaperones	Trigger factor, ppiase (EC 5.2.1.8)	2,568	P	P	P	P	P	P	P	P	1,150	P	P	P	P	P	P	P	P
1031441	COG3343K	gbs0105	rpoE	Cellular processess	Transcription	DNA-directed RNA polymerase delta chain (EC 2.7.7.6)	2,822	P	P	P	P	P	P	P	P	0,855	P	P	P	P	P	P	P	P
1031303	COG0504F	gbs0106	pyrG	Metabolism and transport	Purines, pyrimidines, nucleosides, and nucleotides	CTP synthase (EC 6.3.4.2)	1,949	P	P	P	P	P	P	P	P	1,240	P	A	P	A	P	P	P	P
1031300	COG1073R	gbs0107		General function predicted only		Alpha beta hydrolase	3,915	P	A	A	A	A	A	A	P	0,678	P	A	M	A	A	A	A	A
1030724	-	gbs0108		Metabolism and transport	Purines, pyrimidines, nucleosides, and nucleotides	Deoxyuridine 5 -triphosphate nucleotidohydrolase (EC 3.6.1.23)	1,832	P	A	A	A	A	A	A	A	0,762	P	A	A	A	A	A	A	A
1031294	COG1066O	gbs0109	radA	Cellular processess	DNA replication, recombination and repair	DNA repair protein RadA	2,711	P	A	P	A	A	A	A	A	2,632	P	A	A	A	A	A	A	A
1031293	-	gbs0110		Metabolism and transport	Central intermediary metabolism	Carbonic anhydrase (EC 4.2.1.1)	0,731	P	P	P	P	P	P	P	P	0,305	P	P	P	A	P	P	P	P
1030491	COG1249C	gbs0111		Metabolism and transport	Energy production and conversion	Pyridine nucleotide-disulphide oxidoreductase family protein (EC 1.-.-.-)	0,664	P	P	P	P	P	P	P	P	0,538	P	P	P	P	P	P	P	P
1031263	COG0008J	gbs0112	gltX	Cellular processess	Translation, ribosomal structure and biogenesis	Glutamyl-tRNA synthetase (EC 6.1.1.17)	1,663	P	P	P	P	P	P	P	P	0,763	P	P	P	P	P	P	P	P
1031265	COG1879G	gbs0113	rbsB	Metabolism and transport	Carbohydrates, organic alcohols, and acids	D-ribose-binding protein	1,068	P	P	P	A	P	P	P	P	2,056	P	A	P	P	P	P	P	P
1030717	COG1172G	gbs0114		Metabolism and transport	Carbohydrates, organic alcohols, and acids	Ribose transport system permease protein rbsC	3,788	P	A	A	A	A	A	A	A	5,328	P	A	A	A	A	A	A	A
1030709	COG1129G	gbs0115		Metabolism and transport	Carbohydrates, organic alcohols, and acids	Ribose transport ATP-binding protein rbsA	0,649	P	P	P	P	P	P	M	P	1,301	P	A	P	P	M	P	P	A
1030707	COG1869G	gbs0116	rbsD	Metabolism and transport	Carbohydrates, organic alcohols, and acids	D-ribose mutarotase (EC 5.1.3.-)	0,818	P	A	P	A	P	P	A	P	1,165	P	A	P	M	M	M	A	M
1031255	COG0524G	gbs0117	rbsK	Metabolism and transport	Carbohydrates, organic alcohols, and acids	Ribokinase (EC 2.7.1.15)	0,920	P	P	P	A	P	A	A	A	1,612	P	A	P	M	A	A	A	A
1031258	COG1609K	gbs0118	rbsR	Cellular processess	Transcription	Ribose operon repressor	0,583	P	P	P	A	P	P	P	P	1,035	P	A	A	A	A	A	A	A
1030699	COG0577V	gbs0119		Transport and binding proteins	Unknown substrate	ABC transporter permease protein	0,871	A	A	A	A	A	A	A	A	0,120	A	A	A	A	A	A	A	A
1031253	COG1136V	gbs0120		Transport and binding proteins	Unknown substrate	ABC transporter ATP-binding protein	0,574	A	A	A	A	A	A	A	A	0,184	A	A	A	A	A	A	A	A
1031250	COG0745TK	gbs0121		Cellular processess	Signal transduction	Two-component response regulator	0,941	P	P	P	A	P	P	P	P	0,948	P	A	P	A	A	A	A	A
1031246	COG0642T	gbs0122		Cellular processess	Signal transduction	Phosphate regulon sensor protein phoR (EC 2.7.3.-)	1,896	P	A	P	A	A	A	A	A	2,338	M	A	A	A	A	A	A	A
1031244	COG0137E	gbs0123	argG	Metabolism and transport	Amino acids, peptides, aminosugars and amines	Argininosuccinate synthase (EC 6.3.4.5)	0,378	P	P	P	A	P	P	P	P	0,370	P	A	P	A	A	A	P	A
1030698	COG0165E	gbs0124	argH	Metabolism and transport	Amino acids, peptides, aminosugars and amines	Argininosuccinate lyase (EC 4.3.2.1)	0,536	P	P	P	P	P	P	P	P	1,150	P	A	P	A	P	P	P	A
1030788	COG0191G	gbs0125	fba	Metabolism and transport	Carbohydrates, organic alcohols, and acids	Fructose-bisphosphate aldolase (EC 4.1.2.13)	0,423	P	P	P	P	P	P	P	P	0,545	P	P	P	P	P	P	P	P
1030068	COG0039C	gbs0126		Metabolism and transport	Energy production and conversion	L-2-hydroxyisocaproate dehydrogenase (EC 1.1.1.-)	0,817	P	P	P	P	P	P	P	P	1,012	P	M	A	A	A	P	A	P
1031241	COG0227J	gbs0127	rpmV	Cellular processess	Translation, ribosomal structure and biogenesis	LSU ribosomal protein L28P	0,720	P	P	P	P	P	P	P	P	0,610	P	P	P	P	P	P	P	P
1030008	-	gbs0128	asp	Cellular processess	Posttranslational modification, protein turnover, chaperones	General stress protein, Gls24 family	0,695	P	P	P	P	P	P	P	P	1,240	P	P	P	P	P	P	P	P
1030784	COG1461R	gbs0129		General function predicted only		Dihydroxyacetone kinase family protein	1,331	P	P	P	P	P	P	P	P	1,545	P	P	P	P	P	P	P	P
1031234	COG0330O	gbs0130		Cellular processess	Posttranslational modification, protein turnover, chaperones	Membrane protease protein family	0,830	P	P	P	P	P	P	P	P	1,650	P	P	P	P	P	P	P	P
1031237	COG1126E	gbs0131		Metabolism and transport	Amino acids, peptides, aminosugars and amines	Amino acid transport ATP-binding protein	1,638	P	P	P	P	P	P	P	P	1,195	P	P	P	P	P	P	P	P
1031235	0765E, COG08	gbs0132		Metabolism and transport	Amino acids, peptides, aminosugars and amines	ABC transporter amino acid-binding protein Amino acid ABC transporter permeas	1,496	P	P	P	P	P	P	P	P	0,956	P	P	P	P	P	P	P	P
1031233	COG4907S	gbs0133		Hypothetical		Hypothetical membrane spanning protein	1,314	P	P	P	P	P	P	P	P	1,222	P	A	P	A	P	P	A	A
1030804	-	gbs0134	uppP	Cellular processess	Toxin production and resistance	Bacitracin resistance protein (Putative undecaprenol kinase) (EC 2.7.1.66)	1,123	P	P	P	P	P	P	P	P	1,788	P	P	P	P	P	P	P	P
1029996	-	gbs0135	mecA	Cellular processess	Transcription	Negative regulator of genetic competence mecA	3,233	P	A	M	A	P	P	P	A	1,990	P	A	A	A	A	P	A	A
1031218	COG0472M	gbs0136		Metabolism and transport	Central intermediary metabolism	Undecaprenyl-phosphate alpha-N-acetylglucosaminephosphotransferase (EC 2.7.8	2,234	P	P	P	P	P	P	P	P	1,410	P	A	P	A	A	P	P	A
1031016	COG0396O	gbs0137	sufC	Cellular processess	Posttranslational modification, protein turnover, chaperones	ATP-dependent transporter sufC	0,865	P	P	P	P	P	P	P	P	0,595	P	A	P	A	A	A	A	A
1031214	-	gbs0138	sufD	Metabolism and transport	Cofactors, prosthetic groups, and carriers	SufD protein	1,167	P	P	P	A	P	P	P	P	0,991	P	A	A	A	A	P	A	A
1031215	COG0520E	gbs0139	nifS3	Metabolism and transport	Amino acids, peptides, aminosugars and amines	Cysteine desulfurase (EC 2.8.1.7) Selenocysteine lyase (EC 4.4.1.16)	1,526	P	P	P	A	P	P	P	P	1,344	P	A	P	A	A	P	P	A
1031211	COG0822C	gbs0140	nifU	Metabolism and transport	Energy production and conversion	IscU protein	1,510	P	P	P	P	P	P	P	P	0,949	P	M	P	P	P	P	P	P
1030191	-	gbs0141	sufB	Transport and binding proteins	Unknown substrate	ABC transporter-associated protein sufB	1,342	P	P	P	P	P	P	P	P	1,047	P	A	P	P	P	P	P	P
1031213	COG1686M	gbs0142		Cell Envelope	Biosynthesis and degradation of murein sacculus and peptidoglycan	D-alanyl-D-alanine serine-type carboxypeptidase (EC 3.4.16.4)	1,021	P	A	P	M	P	M	A	A	1,474	P	A	A	A	A	A	A	A
1030695	COG1686M	gbs0143	dacA2	Cell Envelope	Biosynthesis and degradation of murein sacculus and peptidoglycan	D-alanyl-D-alanine serine-type carboxypeptidase (EC 3.4.16.4)	1,738	P	A	P	A	P	P	P	P	1,529	P	A	A	A	A	A	A	A
1030112	COG4166E	gbs0144	oppA	Metabolism and transport	Amino acids, peptides, aminosugars and amines	Oligopeptide-binding protein oppA	0,625	P	P	P	P	P	P	P	P	1,117	P	P	P	P	P	P	P	P
1030110	COG0601EP	gbs0145		Metabolism and transport	Amino acids, peptides, aminosugars and amines	Oligopeptide transport system permease protein oppB	1,091	P	P	P	P	P	P	P	P	1,721	P	A	P	A	P	P	P	P
1031444	COG1173EP	gbs0146	oppC	Metabolism and transport	Amino acids, peptides, aminosugars and amines	Oligopeptide transport system permease protein oppC	1,535	P	P	P	P	P	P	P	P	1,935	P	P	P	P	P	P	P	P
1031206	COG0444EP	gbs0147	oppD	Metabolism and transport	Amino acids, peptides, aminosugars and amines	Oligopeptide transport ATP-binding protein oppD	1,489	P	P	P	P	P	P	P	P	2,146	P	P	P	P	P	P	P	P
1031432	COG4608E	gbs0148	oppF	Metabolism and transport	Amino acids, peptides, aminosugars and amines	Oligopeptide transport ATP-binding protein oppF	1,169	P	P	P	P	P	P	P	P	2,010	P	P	P	P	P	P	P	P
1030799	COG1947I	gbs0149	ispE	Metabolism and transport	Fatty acid and phospholipids	4-diphosphocytidyl-2-C-methyl-D-erythritol kinase (EC 2.7.1.148)	3,078	P	P	P	P	P	P	P	P	1,019	P	P	P	P	P	P	P	P
1030844	COG1846K	gbs0150	adcR	Cellular processess	Transcription	Transcriptional regulator, MarR family	2,825	P	P	P	P	P	P	P	P	1,427	P	P	P	P	P	P	P	P
1031199	COG1121P	gbs0151	adcC	Metabolism and transport	Inorganic ion transport and metabolism	High-affinity zinc uptake system ATP-binding protein znuC	2,276	P	P	P	P	P	P	P	P	1,172	P	P	P	M	P	P	P	P
1031194	COG1108P	gbs0152	adcB	Metabolism and transport	Inorganic ion transport and metabolism	High-affinity zinc uptake system membrane protein znuB	4,737	P	A	P	A	A	A	A	A	1,886	P	A	A	A	A	P	A	A
1031196	-	gbs0153		General function predicted only		Streptodornase (EC 3.1.21.1)	0,417	P	P	P	A	P	A	A	A	2,070	P	A	A	A	A	A	A	A
1031384	COG0162J	gbs0154	tyrS	Cellular processess	Translation, ribosomal structure and biogenesis	Tyrosyl-tRNA synthetase (EC 6.1.1.1)	1,608	P	A	P	A	P	P	M	A	0,905	P	A	A	A	A	A	A	A
1031193	COG0744M	gbs0155	pbp1b	Cell Envelope	Biosynthesis and degradation of murein sacculus and peptidoglycan	Multimodular transpeptidase-transglycosylase PBP 1B (EC 2.4.1.129)	1,275	P	A	A	A	P	A	A	A	0,867	P	A	P	A	A	P	A	A
1031385	COG0085K	gbs0156	rpoB	Cellular processess	Transcription	DNA-directed RNA polymerase beta chain (EC 2.7.7.6)	1,496	P	P	P	P	P	P	P	P	0,643	P	P	P	M	P	P	P	P
1031192	COG0086K	gbs0157	rpoC	Cellular processess	Transcription	DNA-directed RNA polymerase beta chain (EC 2.7.7.6)	1,428	P	P	P	P	P	P	P	P	1,039	P	P	P	P	P	P	P	P
1031191	-	gbs0158		General function predicted only		Putative DNA binding protein	1,553	P	P	P	P	P	P	P	P	0,918	P	P	P	P	P	P	P	P
1030692	COG2804NU	gbs0159	comYA	Cellular processess	DNA transformation	ComG operon protein 1	0,138	A	A	A	A	A	A	A	A	0,117	A	A	A	A	A	A	A	A
1030286	COG1459NU	gbs0160	comYB	Cellular processess	DNA transformation	ComG operon protein 2	0,247	A	A	A	A	A	A	A	P	0,216	P	A	A	A	A	A	P	P
1031190	COG4537U	gbs0161	comYC	Cellular processess	DNA transformation	ComG operon protein 3	0,771	A	A	A	A	A	A	A	A	0,881	A	A	A	A	A	A	A	A
1030569	COG2165NU	gbs0162		Cellular processess	DNA transformation	ComG operon protein 4	1,164	A	A	A	A	A	A	A	A	0,279	A	A	A	A	A	A	A	A
1031678	COG2165NU	gbs0163		Cellular processess	DNA transformation	ComG operon protein 5	0,235	A	A	A	A	A	A	A	A	0,142	A	A	A	A	A	A	A	A
1031456	COG4940U	gbs0164	comYD	Cellular processess	DNA transformation	ComG operon protein 6	0,918	A	A	A	A	A	A	A	A	0,569	A	A	A	A	A	A	A	A
1031182	-	gbs0165		Cellular processess	DNA transformation	ComG operon protein 6	0,723	A	A	A	A	A	A	A	A	0,106	A	A	A	A	A	A	A	A
1030503	COG0827L	gbs0166		Cellular processess	DNA replication, recombination and repair	Adenine-specific methyltransferase (EC 2.1.1.72)	3,338	P	A	A	A	M	A	A	A	2,802	A	A	A	A	A	A	A	A
1031180	COG0282C	gbs0167	ackA	Metabolism and transport	Energy production and conversion	Acetate kinase (EC 2.7.2.1)	1,444	P	P	P	P	P	P	P	P	0,926	P	P	P	P	P	P	P	P
1030243	COG1476K	gbs0168		Cellular processess	Transcription	Transcriptional regulator, Cro CI family	1,048	P	P	P	P	P	P	P	P	0,973	P	A	P	M	P	P	M	P
1031383	-	gbs0169		Hypothetical		Hypothetical protein	1,259	P	P	P	M	P	M	P	P	0,793	P	A	A	A	M	A	A	A
1031177	-	gbs0170		Cellular processess	Posttranslational modification, protein turnover, chaperones	CAAX amino terminal protease family	1,153	P	P	P	P	P	P	P	P	1,190	P	A	P	P	P	P	P	P
1030209	COG0345E	gbs0171	proC	Metabolism and transport	Amino acids, peptides, aminosugars and amines	Pyrroline-5-carboxylate reductase (EC 1.5.1.2)	1,869	P	P	P	P	P	P	P	P	1,971	P	A	P	A	P	P	A	P
1030208	COG1363G	gbs0172	pepA	Metabolism and transport	Carbohydrates, organic alcohols, and acids	Glutamyl aminopeptidase (EC 3.4.11.7)	1,290	P	M	P	A	A	A	A	A	1,243	P	A	A	A	A	A	A	A
1031171	-	gbs0173		Hypothetical		Hypothetical protein	3,289	P	A	P	P	P	P	P	P	1,362	P	A	P	A	M	P	P	A
1031679	-	gbs0174		Hypothetical		Hypothetical membrane associated protein	3,202	P	P	P	A	P	P	P	A	1,195	P	A	P	A	A	P	A	A
1030857	COG0526OC	gbs0175	trxA	Metabolism and transport	Energy production and conversion	Thioredoxin	3,036	P	P	P	A	P	P	P	P	1,327	P	A	P	A	A	A	P	M
1030174	COG0073R	gbs0176		General function predicted only		tRNA binding domain protein	1,595	P	P	P	P	P	P	P	P	0,777	P	A	P	A	A	P	P	M
1031169	COG0500QR	gbs0177		Metabolism and transport	Secondary metabolites	Methyltransferase (EC 2.1.1.-)	2,193	P	A	P	A	P	P	A	P	1,078	P	A	A	A	A	P	A	A
1030805	COG0629L	gbs0178		Cellular processess	DNA replication, recombination and repair	Single-strand DNA binding protein	0,819	A	A	A	A	A	A	A	A	0,151	A	A	A	A	A	A	A	A
1030840	COG0637R	gbs0179		Metabolism and transport	Carbohydrates, organic alcohols, and acids	Beta-phosphoglucomutase (EC 5.4.2.6) Glucose-1-phosphate phosphodismutase	0,997	P	M	P	A	P	M	A	A	1,247	P	A	A	A	A	A	A	A
1031387	COG3275T	gbs0180		Cellular processess	Signal transduction	Autolysin sensor kinase (EC 2.7.3.-)	1,802	P	A	A	A	A	A	A	A	0,922	P	A	A	A	A	A	A	A
1029880	COG3279KT	gbs0181		Cellular processess	Transcription	Autolysin response regulator	0,827	P	P	P	P	P	P	P	A	0,849	P	A	P	A	M	P	A	P
1031167	COG1380R	gbs0182		Cell Envelope	Biosynthesis and degradation of murein sacculus and peptidoglycan	Murein hydrolase exporter	0,784	A	A	A	A	A	A	A	A	0,120	P	P	P	M	P	P	P	P
1030827	-	gbs0183		Cell Envelope	Biosynthesis and degradation of murein sacculus and peptidoglycan	Murein hydrolase export regulator	1,020	P	A	A	A	A	A	A	A	0,130	P	P	P	M	P	P	P	P
1031166	COG0747E	gbs0184	dppA	Metabolism and transport	Amino acids, peptides, aminosugars and amines	Dipeptide-binding protein	1,420	P	P	A	A	P	P	A	A	1,229	A	A	A	A	A	A	A	A
1030990	COG0601EP	gbs0185	dppB	Metabolism and transport	Amino acids, peptides, aminosugars and amines	Dipeptide transport system permease protein dppB	1,297	A	A	A	A	A	A	A	A	1,091	A	A	A	A	A	A	A	A
1030966	COG1173EP	gbs0186	dppC	Metabolism and transport	Amino acids, peptides, aminosugars and amines	Dipeptide transport system permease protein dppC	2,002	P	A	A	A	A	A	A	A	0,275	A	A	A	A	A	A	A	A
1031164	COG0444EP	gbs0187	dppD	Metabolism and transport	Amino acids, peptides, aminosugars and amines	Dipeptide transport ATP-binding protein dppD	1,728	A	A	A	A	A	A	A	A	1,434	A	A	A	A	A	A	A	A
1030832	COG1124EP	gbs0188	dppE	Metabolism and transport	Amino acids, peptides, aminosugars and amines	Dipeptide transport ATP-binding protein dppF	2,544	P	A	A	A	A	A	A	A	0,270	A	A	A	A	A	A	A	A
1031157	COG1263G	gbs0189		Metabolism and transport	Carbohydrates, organic alcohols, and acids	PTS system, trehalose-specific IIBC component (EC 2.7.1.69) PTS system, treha	1,000	P	A	A	A	P	A	A	P	0,882	P	A	A	A	P	A	P	A

1031158	-	gbs0190	dexS	Metabolism and transport	Carbohydrates, organic alcohols, and acids	Trehalose-6-phosphate hydrolase (EC 3.2.1.93)	1,278	P	A	A	A	P	P	A	A	1,163	P	P	P	P	P	P	P	P
1031156	1762GT, COG3	gbs0191		Cellular processess	Transcription	Transcription antiterminator, BglG family PTS system, mannitol (Cryptic)-specific	0,075	A	A	A	A	A	A	A	A	1,916	P	A	M	P	A	A	A	M
1030573	COG3414G	gbs0192		Metabolism and transport	Carbohydrates, organic alcohols, and acids	PTS SYSTEM, IIB COMPONENT (EC 2.7.1.69)	0,380	P	P	P	P	P	P	P	P	1,018	P	P	P	P	A	P	P	P
1029929	-	gbs0193	ulaA	Transport and binding proteins	Unknown substrate	Putative transport protein sgaT	0,416	M	A	A	P	A	A	A	A	1,214	P	P	P	P	A	P	P	P
1030740	COG3959G	gbs0194		Metabolism and transport	Carbohydrates, organic alcohols, and acids	Transketolase (EC 2.2.1.1)	0,515	A	A	A	A	A	A	A	A	1,357	P	A	P	P	M	A	A	A
1031454	COG3958G	gbs0195		Metabolism and transport	Carbohydrates, organic alcohols, and acids	Transketolase (EC 2.2.1.1)	1,228	A	A	A	A	A	A	A	A	1,000	P	A	P	P	A	A	A	A
1031153	COG4097P	gbs0196		Metabolism and transport	Inorganic ion transport and metabolism	Vanillate O-demethylase oxidoreductase (EC 1.14.13.-)	1,993	P	A	A	A	P	A	A	M	1,355	P	A	A	A	A	A	A	A
1031150	-	gbs0197	rpsO	Cellular processess	Translation, ribosomal structure and biogenesis	SSU ribosomal protein S15P	1,388	P	P	P	P	P	P	P	P	0,674	P	P	P	P	P	P	P	P
1031145	COG1185J	gbs0198	pnpA	Cellular processess	Translation, ribosomal structure and biogenesis	Polyribonucleotide nucleotidyltransferase (EC 2.7.7.8) Polynucleotide adenylyltra	2,727	P	P	P	A	P	P	A	M	0,831	P	A	P	A	A	P	P	A
1030002	-	gbs0199		Hypothetical		Hypothetical protein	3,082	P	P	P	P	P	P	P	P	1,440	P	P	P	P	P	P	P	P
1031138	COG1045E	gbs0200	cysE	Metabolism and transport	Amino acids, peptides, aminosugars and amines	Serine acetyltransferase (EC 2.3.1.30)	4,115	P	P	P	A	P	P	P	P	1,732	P	P	P	A	M	P	P	P
1031141	-	gbs0201		Hypothetical		Hypothetical protein	3,597	P	P	P	P	P	P	P	P	1,418	P	P	P	P	P	P	P	P
1031132	COG0215J	gbs0202	cysS	Cellular processess	Translation, ribosomal structure and biogenesis	Cysteinyl-tRNA synthetase (EC 6.1.1.16)	3,189	P	P	P	P	P	P	P	P	1,686	P	P	P	P	P	P	P	P
1031126	COG1939S	gbs0203		Hypothetical		Hypothetical protein	2,802	P	P	P	P	P	P	P	P	1,646	P	A	P	M	P	P	P	P
1031127	COG0566J	gbs0204		Cellular processess	Translation, ribosomal structure and biogenesis	23S rRNA Gm2251 methyltransferase (EC 2.1.1.-)	0,903	P	P	P	P	P	P	P	P	1,093	P	A	P	P	M	P	P	P
1031004	-	gbs0205		Hypothetical		Hypothetical cytosolic protein	0,954	P	P	P	P	P	P	P	P	1,106	P	A	P	P	P	P	P	P
1030748	COG1307S	gbs0206	DegV	Metabolism and transport	Fatty acid and phospholipids	Fatty acid-binding protein, DegV family	1,711	P	P	P	P	P	P	P	P	1,037	P	P	P	P	P	P	P	P
1030907	COG1476K	gbs0207		Cellular processess	Transcription	Transcriptional regulator, Cro CI family	1,331	P	A	A	A	A	A	A	A	0,293	A	A	A	A	A	A	A	A
1030906	COG2826L	gbs0208		Mobile and extrachromosomal elements		Transposase	0,420	P	A	P	A	P	M	A	A	1,778	P	A	A	A	A	M	A	A
1030745	-	gbs0209	rplM	Cellular processess	Translation, ribosomal structure and biogenesis	LSU ribosomal protein L13P	1,336	P	P	P	P	P	P	P	P	0,967	P	P	P	P	P	P	P	P
1031664	-	gbs0210	rpsI	Cellular processess	Translation, ribosomal structure and biogenesis	SSU ribosomal protein S9P	1,200	P	P	P	P	P	P	P	P	0,806	P	P	P	M	P	P	P	P
1031123	COG0582L	gbs0211	int.1	Mobile and extrachromosomal elements		DNA integration recombination inversion protein	0,772	P	A	P	P	M	A	M	A	1,071	P	A	A	A	A	A	A	A
1031018	-	gbs0212		Hypothetical		Hypothetical protein	1,022	P	P	P	P	P	P	P	P	1,283	P	A	P	P	A	P	P	A
1030061	-	gbs0213		Hypothetical		Hypothetical protein	0,466	A	A	A	A	A	A	A	A	0,200	A	A	A	A	A	A	A	A
1030798	-	gbs0214		Hypothetical		Hypothetical protein	0,484	A	A	A	A	A	A	A	A	0,445	P	A	A	A	A	A	A	A
1031124	-	gbs0215		Hypothetical		Hypothetical protein	0,584	A	A	A	A	A	A	A	A	0,266	A	A	A	A	A	A	A	A
1030509	COG1393P	gbs0216		Hypothetical		Hypothetical protein	0,499	A	A	A	A	A	A	A	A	0,240	A	A	A	A	A	A	A	A
1031448	-	gbs0217	repE	General function predicted only		Replication initiation protein, RepA family	0,533	A	A	A	A	A	A	A	A	0,570	A	A	A	A	A	A	A	A
1030104	-	gbs0218		Hypothetical		Hypothetical protein	0,468	A	A	A	A	A	A	A	A	0,187	A	A	A	A	A	A	A	A
1031125	COG1196D	gbs0219	preA	Mobile and extrachromosomal elements		Plasmid recombination protein, Mob family	0,368	P	A	P	P	P	A	M	P	0,453	P	A	A	A	A	A	A	M
1029946	-	gbs0220		Hypothetical		Hypothetical protein	0,254	A	A	A	A	A	A	A	A	1,119	A	A	A	A	A	A	A	A
1030479	-	gbs0221		Hypothetical		Hypothetical cytosolic protein	0,557	P	P	P	P	P	P	P	P	0,816	P	P	P	P	P	P	P	P
1030793	-	gbs0222		Cellular processess	DNA replication, recombination and repair	DNA-damage-inducible protein J	0,490	P	P	P	P	P	P	P	P	1,051	P	P	P	P	P	P	P	P
1030797	COG4842S	gbs0223		Hypothetical		Hypothetical protein	0,909	P	P	P	P	P	P	P	P	1,739	P	A	A	A	A	A	A	A
1030450	-	gbs0224		Hypothetical		Hypothetical protein	0,495	P	P	P	P	P	P	P	P	0,830	P	A	P	P	P	P	A	P
1031146	-	gbs0225		Cellular processess	DNA replication, recombination and repair	Hypothetical protein	0,643	P	P	P	P	P	P	P	P	1,963	P	A	P	P	P	P	A	M
1031149	-	gbs0226		Hypothetical		Hypothetical protein	0,867	P	P	P	P	P	P	P	P	0,869	P	A	P	P	P	P	A	P
1030786	-	gbs0228		Hypothetical		Hypothetical protein	0,973	P	A	P	P	P	P	A	P	2,753	P	A	A	A	A	A	A	A
1031152	-	gbs0229		Hypothetical		Hypothetical protein	0,900	P	P	P	P	P	P	P	P	1,439	P	A	P	P	A	P	A	A
1029978	-	gbs0230	mutR	Cellular processess	Transcription	Transcriptional regulator	0,728	P	P	P	P	P	P	M	P	2,011	P	A	A	A	A	A	A	A
1030927	-	gbs0231	mefE	Cellular processess	Toxin production and resistance	Macrolide-efflux protein	1,770	P	P	P	A	P	P	P	M	1,316	P	A	A	A	A	A	A	A
1031154	COG1174E	gbs0232		Metabolism and transport	Amino acids, peptides, aminosugars and amines	Glycine betaine carnitine choline transport system permease protein opuCD	1,531	P	P	P	M	P	P	P	P	0,908	P	P	P	A	A	P	P	P
1031424	COG1732M	gbs0233		Metabolism and transport	Amino acids, peptides, aminosugars and amines	Glycine betaine carnitine choline-binding protein	1,254	P	P	P	P	P	M	P	P	1,316	P	A	A	A	A	P	M	M
1031155	COG1174E	gbs0234		Metabolism and transport	Amino acids, peptides, aminosugars and amines	Glycine betaine carnitine choline transport system permease protein opuCB	1,488	P	P	P	M	P	P	P	P	1,794	P	A	P	A	A	P	A	A
1030037	COG1125E	gbs0235		Metabolism and transport	Amino acids, peptides, aminosugars and amines	Glycine betaine carnitine choline transport ATP-binding protein opuCA	1,218	P	P	P	P	P	P	P	P	1,284	P	A	P	P	M	P	A	A
1030836	-	gbs0236		Hypothetical		Hypothetical protein	0,663	P	P	P	P	P	P	P	P	0,920	P	P	P	P	P	P	P	P
1031440	COG0582L	gbs0237		Mobile and extrachromosomal elements		DNA integration recombination inversion protein	0,878	P	P	P	A	A	A	P	M	1,078	P	A	A	A	A	P	A	P
1031168	-	gbs0238		Hypothetical		Hypothetical protein	0,945	P	A	P	P	P	P	P	A	1,069	P	A	P	A	A	A	A	A
1031172	-	gbs0239		General function predicted only		Rep protein	0,713	A	A	A	A	A	A	A	A	0,467	A	A	A	A	A	A	A	A
1031173	-	gbs0240		Hypothetical		Hypothetical protein	0,411	P	P	P	P	P	P	P	P	1,220	P	A	P	A	M	P	A	A
1031453	COG1674D	gbs0241		Cellular processess	Cell division	FtsK SpoIIIE family	0,394	P	P	P	P	P	P	P	P	1,160	P	A	A	A	A	P	A	A
1031174	-	gbs0242		Hypothetical		Hypothetical protein	0,459	P	P	P	P	P	P	P	P	1,556	P	A	P	A	A	P	A	A
1030826	-	gbs0243		Hypothetical		Hypothetical protein	0,355	P	P	P	P	P	P	P	P	0,510	P	P	M	A	P	P	P	A
1030232	-	gbs0244		Hypothetical		Hypothetical protein	1,359	P	P	P	P	P	P	M	P	1,210	P	A	P	A	A	P	A	P
1031189	COG1670J	gbs0245		Metabolism and transport	Central intermediary metabolism	Acetyltransferase (EC 2.3.1.-)	0,796	P	P	P	P	P	P	P	P	1,298	P	P	P	P	P	P	P	P
1031175	COG1670J	gbs0246		Cellular processess	Translation, ribosomal structure and biogenesis	Ribosomal-protein-alanine acetyltransferase (EC 2.3.1.128)	0,940	P	P	P	P	P	P	P	P	1,657	P	P	P	P	P	P	P	P
1030934	COG1670J	gbs0247		Cellular processess	Translation, ribosomal structure and biogenesis	Ribosomal-protein-serine acetyltransferase (EC 2.3.1.-)	1,283	P	P	P	P	P	P	P	P	1,625	P	M	P	P	P	P	P	P
1031386	-	gbs0248		general function predicted only		ECF-type sigma factor negative effector	0,973	P	A	P	A	A	P	P	M	1,190	P	A	A	A	A	A	A	A
1030245	COG1595K	gbs0249		Cellular processess	Transcription	RNA polymerase ECF-type sigma factor	0,872	A	A	A	A	A	A	A	A	3,027	A	A	A	A	A	A	A	A
1031184	COG1309K	gbs0250		Cellular processess	Transcription	Transcriptional regulator, TetR family	5,244	P	A	A	A	A	A	A	A	0,221	A	A	A	A	A	A	A	A
1031185	COG0842V	gbs0251	sagI	Transport and binding proteins	Unknown substrate	ABC transporter permease protein	0,891	A	A	A	A	A	A	A	A	0,089	A	A	A	A	A	A	A	A
1031420	COG1131V	gbs0252	sagG	Cellular processess	Toxin production and resistance	Daunorubicin resistance ATP-binding protein drrA	0,422	A	A	A	A	A	A	A	A	0,076	A	A	A	A	A	A	A	A
1031187	-	gbs0253		Hypothetical		Hypothetical protein	1,041	P	A	P	A	P	P	P	A	1,071	P	A	A	A	A	M	A	P
1031382	COG1695K	gbs0254		Cellular processess	Transcription	Transcriptional regulator, PadR family	1,700	P	A	P	A	M	P	A	P	2,084	P	A	A	A	A	M	A	A
1030359	-	gbs0255		Hypothetical		Hypothetical protein	0,625	P	P	P	P	P	P	P	P	1,151	P	P	P	P	P	P	P	P
1031426	COG1820G	gbs0256	nagA	Metabolism and transport	Carbohydrates, organic alcohols, and acids	N-acetylglucosamine-6-phosphate deacetylase (EC 3.5.1.25)	0,765	P	P	P	P	P	P	P	P	0,766	P	A	A	A	A	P	A	A
1030358	-	gbs0257		Hypothetical		Hypothetical membrane spanning protein	3,247	P	P	P	P	P	P	P	P	3,954	P	A	P	P	P	P	P	P
1030838	-	gbs0258	glyQ	Cellular processess	Translation, ribosomal structure and biogenesis	Glycyl-tRNA synthetase alpha chain (EC 6.1.1.14)	1,468	P	P	P	P	P	P	P	P	1,003	P	A	P	P	P	P	P	P
1031429	COG1182I	gbs0259		Metabolism and transport	Fatty acid and phospholipids	FMN-dependent NADH-azoreductase (EC 1.6.99.2)	1,782	P	P	P	P	P	P	P	P	1,325	P	A	P	A	A	P	A	A
1031195	COG0751J	gbs0260	glyS	Cellular processess	Translation, ribosomal structure and biogenesis	Glycyl-tRNA synthetase beta chain (EC 6.1.1.14)	1,390	P	P	P	P	P	P	P	P	1,322	P	P	P	P	P	P	P	P
1030775	-	gbs0261		Hypothetical		Hypothetical cytosolic protein	1,186	P	P	P	P	P	P	P	P	1,756	P	P	P	P	P	P	P	P
1030809	-	gbs0262		Hypothetical		Hypothetical membrane spanning protein	1,086	P	A	P	A	P	P	M	A	1,125	P	A	A	A	A	P	A	A
1030693	COG0554C	gbs0263	glpK	Metabolism and transport	Energy production and conversion	Glycerol kinase (EC 2.7.1.30)	0,323	P	P	P	P	P	P	P	P	1,152	P	P	P	P	P	P	P	P
1031442	COG0578C	gbs0264	glpD	Metabolism and transport	Energy production and conversion	Alpha-glycerophosphate oxidase (EC 1.1.3.21)	0,423	P	A	P	A	P	A	A	P	1,407	P	P	P	P	P	P	P	P
1030430	-	gbs0265	glpF	Metabolism and transport	Carbohydrates, organic alcohols, and acids	Glycerol uptake facilitator protein	0,578	P	A	M	A	A	P	M	P	1,283	P	P	P	P	P	P	P	P
1031204	COG0446R	gbs0266		Metabolism and transport	Central intermediary metabolism	NADH peroxidase (EC 1.11.1.1)	2,532	P	A	P	A	A	P	A	A	1,839	P	P	P	P	P	P	P	P
1030416	-	gbs0267	mga ?	Hypothetical		trans-acting positive regulator	2,819	P	A	A	A	A	A	A	A	1,678	P	P	P	P	P	P	P	P
1030442	COG0021G	gbs0268	tkt	Metabolism and transport	Carbohydrates, organic alcohols, and acids	Transketolase (EC 2.2.1.1)	0,963	P	A	P	A	P	P	P	M	1,230	P	A	P	A	M	P	P	A
1030385	-	gbs0269		Hypothetical		Hypothetical protein	2,135	P	P	P	P	P	P	P	P	2,082	P	P	P	P	P	P	P	P
1031207	COG1131V	gbs0270		Transport and binding proteins	Unknown substrate	ABC transporter ATP-binding protein	1,274	P	P	P	P	P	P	P	P	1,606	P	M	P	P	P	P	P	P
1030079	-	gbs0271	proB	Transport and binding proteins	Unknown substrate	ABC transporter permease protein	2,096	P	P	P	P	P	P	P	P	2,175	P	A	P	P	P	P	P	P
1031209	COG1263G	gbs0272		Metabolism and transport	Carbohydrates, organic alcohols, and acids	PTS system, beta-glucoside-specific IIABC component (EC 2.7.1.69)	0,668	P	A	A	A	A	A	A	A	0,252	A	A	A	A	A	A	A	A
1031210	COG0263E	gbs0273	proB	Metabolism and transport	Amino acids, peptides, aminosugars and amines	Glutamate 5-kinase (EC 2.7.2.11)	1,719	P	M	P	P	P	P	P	A	1,606	P	A	M	A	A	P	A	A
1031437	COG0014E	gbs0274	proA	Metabolism and transport	Amino acids, peptides, aminosugars and amines	Gamma-glutamyl phosphate reductase (EC 1.2.1.41)	1,380	P	P	P	A	P	M	P	A	0,985	P	A	A	A	A	A	A	A
1031208	-	gbs0275	mraW	General function predicted only		S-adenosyl-methyltransferase mraW (EC 2.1.1.-)	4,549	P	A	A	A	A	A	A	A	1,541	P	A	A	A	A	A	A	A
1030691	-	gbs0276	ftsL	Cellular processess	Cell division	Cell division protein ftsL	4,861	P	P	P	A	P	P	M	P	1,368	P	M	P	A	P	P	A	A
1031219	COG0768M	gbs0277		Cell Envelope	Biosynthesis and degradation of murein sacculus and peptidoglycan	Division specific D,D-transpeptidase Cell division protein ftsI	1,461	P	A	A	M	A	A	P	A	0,550	P	A	A	A	A	P	P	A
1030464	COG0472M	gbs0278	mraY	Cell Envelope	Biosynthesis and degradation of murein sacculus and peptidoglycan	Phospho-N-acetylmuramoyl-pentapeptide-transferase (EC 2.7.8.13)	1,645	P	A	A	A	P	P	A	A	0,821	P	M	P	A	A	P	A	A
1031649	COG0513LKJ	gbs0279	deaD	Cellular processess	DNA replication, recombination and repair	ATP-dependent RNA helicase	0,567	P	P	P	P	P	P	P	P	0,881	P	A	P	P	P	P	A	A
1031220	COG0834ET	gbs0280		Transport and binding proteins	Unknown substrate	ABC transporter substrate-binding protein	0,573	P	P	P	P	P	P	P	P	0,254	P	A	A	A	P	P	A	A
1030039	COG0765E	gbs0281		Metabolism and transport	Amino acids, peptides, aminosugars and amines	Amino acid ABC transporter permease protein	1,018	P	A	P	M	M	P	P	P	0,980	M	A	A	A	A	A	A	A
1030231	COG1126E	gbs0282		Metabolism and transport	Amino acids, peptides, aminosugars and amines	Amino acid transport ATP-binding protein	1,360	P	P	P	A	M	A	A	M	0,681	M	A	A	A	A	A	A	A
1030089	-	gbs0283	aapA	Hypothetical		Hypothetical membrane associated protein	1,673	P	P	P	A	P	P	P	P	0,527	P	A	P	A	P	P	A	A
1030075	COG0492O	gbs0284	trxB	Metabolism and transport	Energy production and conversion	Thioredoxin reductase (EC 1.8.1.9)	1,027	P	P	P	P	P	P	P	P	0,652	P	P	P	P	P	P	P	P
1030696	COG1488H	gbs0285	nadE	Metabolism and transport	Cofactors, prosthetic groups, and carriers	Nicotinate phosphoribosyltransferase (EC 2.4.2.11)	1,659	P	P	P	P	P	P	P	P	1,472	P	A	P	A	P	P	P	M

1031238	COG0171H	gbs0286	nadE	Metabolism and transport	Cofactors, prosthetic groups, and carriers	NH(3)-dependent NAD(+) synthetase (EC 6.3.5.1)	1,103	P	P	P	A	P	P	M	P	0,952	P	A	A	A	A	A	A	A
1030350	COG3579E	gbs0287	pepC	Metabolism and transport	Amino acids, peptides, aminosugars and amines	Aminopeptidase C (EC 3.4.22.40)	1,003	P	P	P	P	P	P	P	P	1,586	P	P	P	P	P	P	P	P
1031240	COG0744M	gbs0288	ponA	Cell Envelope	Biosynthesis and degradation of murein sacculus and peptidoglycan	Multimodular transpeptidase-transglycosylase PBP 1A	1,637	P	P	P	P	P	P	P	A	1,433	P	P	P	P	P	P	P	P
1031247	COG3331R	gbs0289	recU	Cellular processess	DNA replication, recombination, and repair	Recombination protein recU	3,435	P	P	P	P	P	P	P	P	1,589	P	P	P	P	P	P	P	P
1031242	-	gbs0290		Hypothetical		Hypothetical cytosolic protein	0,717	P	A	A	A	A	A	A	A	0,415	P	A	A	A	A	A	A	A
1030697	COG3599D	gbs0291		Cellular processess	Cell division	Cell division initiation protein DivIVA	2,054	P	P	P	P	P	P	P	P	1,033	P	P	P	P	P	P	P	P
1031245	COG0116L	gbs0292		Metabolism and transport	Central intermediary metabolism	Methyltransferase (EC 2.1.1.-)	1,681	P	A	P	A	A	P	A	A	0,634	P	A	M	A	A	M	A	A
1031248	-	gbs0293		Hypothetical		Hypothetical protein	1,533	P	P	P	P	P	P	P	P	0,905	P	M	P	P	P	P	P	P
1030700	-	gbs0294	luxS	Cellular processess	Signal transduction	Autoinducer-2 production protein luxS (EC 3.13.1.-)	1,546	P	P	P	P	P	P	P	P	1,986	P	A	P	A	M	P	A	A
1031251	COG1418R	gbs0295		Metabolism and transport	Central intermediary metabolism	Hydrolase (HAD superfamily)	2,209	P	P	P	A	P	P	P	P	0,888	P	P	P	A	P	P	P	P
1031256	COG1131V	gbs0296		Transport and binding proteins	Unknown substrate	ABC transporter ATP-binding protein	1,658	P	A	M	A	P	A	A	A	1,098	A	A	A	A	A	A	A	A
1031254	COG0842V	gbs0297		Transport and binding proteins	Unknown substrate	ABC transporter permease protein	0,820	M	A	A	A	A	A	A	A	0,925	A	A	A	A	A	A	A	A
1031261	COG4585T	gbs0298		Cellular processess	Signal transduction	Sensory Transduction Histidine Kinase (EC 2.7.3.-)	1,526	P	A	P	P	P	P	A	A	0,618	P	A	P	A	M	P	A	A
1030718	COG2197TK	gbs0299		Cellular processess	Signal transduction	Two-component response regulator	1,662	P	A	P	A	P	P	A	A	0,832	P	A	A	A	A	A	A	A
1030702	-	gbs0300		Hypothetical		Hypothetical membrane spanning protein	1,181	P	A	P	P	P	P	A	P	0,475	P	A	P	M	P	A	A	A
1031266	COG0194F	gbs0301	gmk	Metabolism and transport	Purines, pyrimidines, nucleosides, and nucleotides	Guanylate kinase (EC 2.7.4.8)	3,205	P	P	P	P	P	P	P	P	1,719	P	P	P	P	P	P	P	P
1031267	COG1758K	gbs0302		Cellular processess	Transcription	DNA-directed RNA polymerase omega chain (EC 2.7.7.6)	1,401	P	P	P	A	P	P	P	P	1,198	P	A	P	A	P	P	P	P
1031268	COG1198L	gbs0303	priA	Cellular processess	DNA replication, recombination and repair	Primosomal protein N	2,221	P	A	P	A	A	A	A	A	1,957	A	A	A	A	A	A	A	A
1031270	COG0223J	gbs0304	fmt	Cellular processess	Translation, ribosomal structure and biogenesis	Methionyl-tRNA formyltransferase (EC 2.1.2.9)	0,881	P	P	P	P	P	P	P	P	0,798	P	A	P	M	P	P	A	A
1030722	COG0144J	gbs0305	sunL	Cellular processess	Translation, ribosomal structure and biogenesis	16S rRNA m(5)C 967 methyltransferase (EC 2.1.1.-)	1,546	P	P	P	M	P	P	P	P	1,268	P	M	P	A	M	P	A	A
1031295	COG0631T	gbs0306	stp1/pppl	Cellular processess	Signal transduction	Protein phosphatase 2C (EC 3.1.3.16)	1,962	P	P	P	P	P	P	P	P	1,268	P	P	P	P	P	P	P	P
1030725	515RTKL, COG	gbs0307	stk1	Cellular processess	Signal transduction	Serine threonine protein kinase (EC 2.7.1.37)	1,495	P	P	P	P	P	P	P	P	1,330	P	P	P	P	P	P	P	P
1030188	COG4758S	gbs0308	yvqF	Transport and binding proteins	Unknown substrate	Transporter yvqF	1,943	P	A	P	A	P	P	A	A	1,062	P	A	P	M	P	A	A	P
1031297	COG4585T	gbs0309	yvqE	Cellular processess	Signal transduction	Two-component sensor protein yvqE (EC 2.7.3.-)	3,980	P	A	A	A	A	A	A	A	1,893	P	A	A	A	A	A	A	A
1031299	COG2197TK	gbs0310	yvqC	Cellular processess	Signal transduction	Two-component response regulator yvqC	1,016	P	A	P	M	P	P	P	P	0,866	P	A	P	A	A	P	P	A
1031439	0561R, COG0	gbs0311		Metabolism and transport	Central intermediary metabolism	Peptidyl-prolyl cis-trans isomerase (EC 5.2.1.8)	1,665	P	P	P	P	P	P	P	P	0,792	P	P	P	P	P	P	P	P
1030520	COG1098J	gbs0312		Cellular processess	Translation, ribosomal structure and biogenesis	S1-type RNA-binding domain	1,689	P	P	P	P	P	P	P	P	0,827	P	A	P	A	P	P	A	A
1030470	COG1180O	gbs0313		Metabolism and transport	Energy production and conversion	Pyruvate formate-lyase activating enzyme (EC 1.97.1.4)	0,051	P	P	P	P	P	P	P	P	0,572	P	P	P	P	P	P	P	P
1031443	COG1349KG	gbs0314		Cellular processess	Transcription	Transcriptional regulator, DeoR family	0,723	P	P	P	P	P	P	P	P	1,389	P	A	P	A	P	P	P	P
1031449	COG2390K	gbs0315		Cellular processess	Transcription	Transcriptional regulatory protein	1,235	P	P	P	P	P	P	P	P	1,706	P	P	P	A	M	P	A	P
1031313	-	gbs0316		Metabolism and transport	Carbohydrates, organic alcohols, and acids	PTS system, cellobiose-specific IIA component (EC 2.7.1.69)	0,429	P	P	P	P	P	P	P	P	0,922	P	A	P	A	A	M	A	P
1031316	COG1440G	gbs0317		Metabolism and transport	Carbohydrates, organic alcohols, and acids	PTS system, cellobiose-specific IIB component (EC 2.7.1.69)	0,315	P	M	P	P	P	P	P	A	0,759	P	A	A	A	A	A	A	A
1030527	COG1455G	gbs0318		Metabolism and transport	Carbohydrates, organic alcohols, and acids	PTS system, cellobiose-specific IIC component (EC 2.7.1.69)	0,240	A	A	A	A	A	A	A	A	0,361	A	A	A	A	A	A	A	A
1030546	COG1882C	gbs0319	pflD	Metabolism and transport	Energy production and conversion	Formate acetyltransferase (EC 2.3.1.54)	0,102	P	P	P	P	P	P	P	P	0,245	P	P	P	P	P	P	P	P
1031318	-	gbs0320	mipB	Metabolism and transport	Energy production and conversion	Transaldolase (EC 2.2.1.2)	0,039	A	A	P	P	P	P	A	P	0,219	P	P	P	P	P	P	P	P
1030547	COG0371C	gbs0321	gldA	Metabolism and transport	Energy production and conversion	Glycerol dehydrogenase (EC 1.1.1.6)	0,172	A	P	P	P	P	P	A	P	0,237	P	P	P	P	P	P	P	P
1031319	COG0031E	gbs0322	cysK	Metabolism and transport	Amino acids, peptides, aminosugars and amines	Cysteine synthase (EC 2.5.1.47)	1,487	P	P	P	M	P	P	P	A	0,923	P	A	A	A	P	M	P	A
1030550	COG1739S	gbs0323		Hypothetical		Hypothetical protein	5,515	P	A	A	A	A	A	A	A	0,750	A	A	A	A	A	A	A	A
1031416	COG4098L	gbs0324	comFA	Cellular processess	DNA transformation	COMF operon protein 1	0,100	A	A	A	A	A	A	A	A	0,035	A	A	A	A	A	A	A	A
1031320	COG1040R	gbs0325	comFC	General function predicted only		COMF operon protein 3	0,445	A	A	A	A	A	M	A	A	0,387	P	A	A	A	A	A	A	A
1030561	COG1544J	gbs0326		Cellular processess	Translation, ribosomal structure and biogenesis	Ribosome-associated factor Y	0,241	P	P	P	P	P	P	P	P	0,281	P	P	P	P	P	P	P	P
1031329	COG0527E	gbs0327	lysC	Metabolism and transport	Amino acids, peptides, aminosugars and amines	Aspartokinase (EC 2.7.2.4)	2,125	P	A	A	A	A	A	A	A	0,625	M	A	A	A	A	A	A	A
1031799	COG0637R	gbs0328		Metabolism and transport	Carbohydrates, organic alcohols, and acids	Beta-phosphoglucomutase (EC 5.4.2.6) Glucose-1-phosphate phosphodismutase	3,166	P	P	P	P	P	A	A	P	1,223	P	A	A	P	A	A	A	A
1031330	COG1024I	gbs0329	phaB	Metabolism and transport	Fatty acid and phospholipids	Enoyl-CoA hydratase (EC 4.2.1.17)	1,164	P	P	P	P	P	P	P	P	0,454	P	A	P	A	A	P	P	P
1031333	COG1846K	gbs0330		Cellular processess	Transcription	Transcriptional regulator, MarR family	1,606	P	P	P	P	P	P	P	P	1,056	P	P	P	P	P	P	P	P
1030728	COG0332I	gbs0331	fabH	Metabolism and transport	Fatty acid and phospholipids	3-oxoacyl-[acyl-carrier-protein] synthase III (EC 2.3.1.41)	1,472	P	P	P	P	P	P	P	P	1,011	P	P	P	P	P	P	P	P
1031336	COG0236IQ	gbs0332	acpP	Metabolism and transport	Fatty acid and phospholipids	Acyl carrier protein	1,155	P	P	P	P	P	P	P	P	1,172	P	P	P	P	P	P	P	P
1031832	COG2070R	gbs0333	fabK	Metabolism and transport	Fatty acid and phospholipids	Enoyl-[acyl-carrier protein] reductase (NADH) (EC 1.3.1.9)	1,698	P	P	P	P	P	P	P	P	1,188	P	A	P	P	A	P	P	P
1031797	COG0331I	gbs0334	fabD	Metabolism and transport	Fatty acid and phospholipids	Malonyl-CoA-[acyl-carrier-protein] transacylase (EC 2.3.1.39)	1,715	P	P	P	P	P	M	P	P	1,157	P	A	P	A	A	M	P	P
1030587	COG1028IQR	gbs0335	fabG	Metabolism and transport	Fatty acid and phospholipids	3-oxoacyl-[acyl-carrier protein] reductase (EC 1.1.1.100)	1,878	P	A	P	P	P	P	P	P	0,893	P	A	P	A	P	P	P	P
1031339	COG0304IQ	gbs0336	fabF	Metabolism and transport	Fatty acid and phospholipids	3-oxoacyl-[acyl-carrier-protein] synthase (EC 2.3.1.41)	1,599	P	P	P	P	P	P	P	P	0,702	P	P	P	P	P	P	P	P
1030727	COG0511I	gbs0337	accB	Metabolism and transport	Fatty acid and phospholipids	Biotin carboxyl carrier protein of acetyl-CoA carboxylase (EC 6.4.1.2)	1,520	P	P	P	P	P	P	P	P	0,824	P	P	P	P	P	P	P	P
1031344	COG0764I	gbs0338	abZ/clpA	Metabolism and transport	Fatty acid and phospholipids	(3R)-hydroxyacyl-[acyl carrier protein] dehydratase (EC 4.2.1.60)	2,662	P	P	P	P	P	P	P	P	0,883	P	P	P	P	P	P	P	P
1030589	COG0439I	gbs0339	accC	Metabolism and transport	Fatty acid and phospholipids	Biotin carboxylase (EC 6.3.4.14)	1,746	P	A	P	A	A	A	P	A	0,791	P	A	P	A	M	P	P	A
1031347	COG0777I	gbs0340	accA	Metabolism and transport	Fatty acid and phospholipids	Acetyl-coenzyme A carboxylase carboxyl transferase subunit beta (EC 6.4.1.2)	1,331	P	P	P	P	P	P	P	P	0,806	P	P	P	P	P	P	P	P
1031345	COG0825I	gbs0341	accD	Metabolism and transport	Fatty acid and phospholipids	Acetyl-coenzyme A carboxylase carboxyl transferase subunit alpha (EC 6.4.1.2)	1,456	P	P	P	P	P	P	P	P	0,865	P	P	P	P	P	P	P	P
1030828	-	gbs0342		Hypothetical		Hypothetical protein	0,658	P	P	P	P	P	P	P	P	0,872	P	P	P	P	P	P	P	P
1031352	COG0172J	gbs0343	serS	Cellular processess	Translation, ribosomal structure and biogenesis	Seryl-tRNA synthetase (EC 6.1.1.11)	0,778	P	P	P	P	P	P	P	P	1,000	P	M	P	A	P	P	P	M
1030671	COG3274S	gbs0344		Hypothetical		Integral membrane protein	2,467	P	P	P	P	P	P	P	P	1,002	P	A	P	P	P	P	A	P
1030667	-	gbs0345	manO	Hypothetical		Hypothetical cytosolic protein	1,773	P	P	P	P	P	P	P	P	2,578	P	P	P	P	P	P	P	P
1030670	-	gbs0346	manN	Metabolism and transport	Carbohydrates, organic alcohols, and acids	PTS system, mannose-specific IID component (EC 2.7.1.69)	1,109	P	P	P	P	P	P	P	P	2,048	P	P	P	P	P	P	P	P
1030669	-	gbs0347	manM	Metabolism and transport	Carbohydrates, organic alcohols, and acids	PTS system, mannose-specific IIC component (EC 2.7.1.69)	1,211	P	P	P	P	P	P	P	P	2,091	P	P	P	P	P	P	P	P
1030489	COG2893G	gbs0348	manL	Metabolism and transport	Carbohydrates, organic alcohols, and acids	PTS system, mannose-specific IIAB component (EC 2.7.1.69)	0,794	P	P	P	P	P	P	P	P	2,068	P	P	P	P	P	P	P	P
1030672	COG0561R	gbs0349		Metabolism and transport	Central intermediary metabolism	Hydrolase (HAD superfamily)	3,961	P	A	A	A	P	A	A	A	1,129	P	A	P	A	M	A	A	A
1031417	-	gbs0350		Hypothetical		Hypothetical protein	1,692	P	P	P	A	P	P	P	P	1,243	P	A	P	A	P	P	A	P
1031366	-	gbs0351		General function predicted only		Permease	2,774	P	P	P	M	P	P	P	P	2,199	P	A	P	A	A	P	A	A
1030742	COG2252R	gbs0352		Metabolism and transport	Purines, pyrimidines, nucleosides, and nucleotides	Guanine-hypoxanthine permease	1,447	P	P	P	P	P	P	P	P	0,711	P	P	P	P	P	P	P	P
1030741	COG0802R	gbs0353		General function predicted only		ATP GTP hydrolase	4,110	P	P	P	P	P	P	P	P	1,397	P	A	P	A	P	P	M	A
1030754	-	gbs0354		Metabolism and transport	Central intermediary metabolism	Acetyltransferase, GNAT family	4,068	P	A	P	A	A	P	A	A	1,271	P	A	P	A	A	A	A	A
1031369	COG1316K	gbs0355	lytR	Cellular processess	Transcription	Transcriptional regulator, LytR family	2,717	P	P	P	P	P	P	P	P	2,061	P	A	P	P	P	P	M	A
1031370	-	gbs0356		Hypothetical		Hypothetical protein	0,990	P	P	P	P	P	P	P	P	1,217	P	P	P	P	P	P	P	P
1031380	COG0537FGR	gbs0357	hit	Metabolism and transport	Purines, pyrimidines, nucleosides, and nucleotides	Adenosine 5 -monophosphoramidase	0,646	P	P	P	P	P	P	P	P	1,025	P	M	M	A	A	P	P	A
1031374	-	gbs0358		Hypothetical		Hypothetical protein	1,004	P	P	P	P	P	P	P	P	3,913	P	P	P	P	P	P	P	P
1030766	COG1131V	gbs0359		Transport and binding proteins	Unknown substrate	ABC-type transporter ATP-binding protein ecsA	0,677	P	P	P	P	P	P	P	P	1,276	P	A	P	P	P	M	P	A
1031402	-	gbs0360		Transport and binding proteins	Unknown substrate	Protein ecsB	1,135	P	P	P	P	P	P	P	P	1,922	P	A	P	A	P	P	A	M
1031674	-	gbs0364		Hypothetical		Hypothetical protein	0,642	A	A	A	A	A	A	A	A	1,369	A	A	A	A	A	A	A	A
1031644	-	gbs0393		Hypothetical		LPXTG Hypothetical protein	0,323	P	P	P	P	P	P	P	P	2,079	P	A	A	A	A	A	A	A
1031640	-	gbs0402		Hypothetical		TRSE PROTEIN	0,316	P	A	P	A	A	A	M	P	0,397	M	A	A	A	A	A	A	A
1031639	-	gbs0403		Hypothetical		Hypothetical protein	0,588	P	A	P	M	M	M	A	A	0,388	M	A	A	A	A	A	A	A
1031636	COG1705NU	gbs0404		Hypothetical		Hypothetical protein	0,493	M	A	A	A	A	A	A	A	0,363	A	A	A	A	A	A	A	A
1031632	-	gbs0405		Hypothetical		Hypothetical protein	0,336	P	M	P	A	P	P	P	A	0,372	P	A	M	A	A	A	A	A
1031633	COG1192D	gbs0406	parA	Cellular processess	Cell division	Hypothetical protein	0,974	P	P	P	M	P	P	P	P	0,644	P	A	A	A	A	P	A	A
1031631	-	gbs0407		Hypothetical		Hypothetical protein	1,366	P	P	P	P	P	P	P	P	0,980	P	A	M	M	A	P	A	A
1031629	-	gbs0408		Hypothetical		Hypothetical protein	1,445	P	P	P	P	P	P	P	P	1,282	P	A	A	A	A	P	P	A
1031627	-	gbs0409		Hypothetical		Hypothetical protein	1,028	P	P	P	P	P	P	P	P	1,221	P	A	P	A	P	P	P	A
1031628	-	gbs0410		Hypothetical		Hypothetical protein	2,264	A	A	A	A	A	A	A	A	0,545	A	A	A	A	A	A	A	A
1031625	COG0510M	gbs0411		General function predicted only		Phosphotransferase enzyme family	3,110	P	P	P	P	P	P	P	P	1,093	P	P	P	P	P	P	P	P
1029806	COG0220R	gbs0412	trmB	Cellular processess	Translation, ribosomal structure and biogenesis	tRNA (m(7)G46) methyltransferase (EC 2.1.1.33)	3,115	P	P	P	P	P	P	P	P	0,976	P	P	P	P	P	P	P	P
1031630	COG0779S	gbs0413		Hypothetical		Hypothetical cytosolic protein	1,755	P	P	P	P	P	P	P	P	1,104	P	A	P	P	P	P	P	P
1029803	COG0195K	gbs0414	nusA	Cellular processess	Transcription	N utilization substance protein A	1,831	P	P	P	P	P	P	P	P	1,180	P	M	P	P	P	P	P	P
1031624	COG2740K	gbs0415		Hypothetical		Hypothetical cytosolic protein	1,932	P	P	P	A	P	P	P	P	2,223	P	A	P	A	A	A	A	P
1030800	COG1358J	gbs0416		Cellular processess	Translation, ribosomal structure and biogenesis	LSU ribosomal protein L7AE	1,603	P	P	P	P	P	P	P	P	1,246	P	A	A	M	A	P	P	P
1031626	COG0532J	gbs0417	infB	Cellular processess	Translation, ribosomal structure and biogenesis	Bacterial Protein Translation Initiation Factor 2 (IF-2)	1,312	P	P	P	P	P	P	P	P	0,933	P	P	P	A	P	P	P	M
1029800	COG0858J	gbs0418	rbfA	Cellular processess	Translation, ribosomal structure and biogenesis	Ribosome-binding factor A	2,062	P	P	P	P	P	P	P	P	2,142	P	A	P	P	P	P	A	P
1031622	COG0657I	gbs0419		Metabolism and transport	Fatty acid and phospholipids	Esterase (EC 3.1.1.-)	3,706	P	P	P	P	P	P	P	P	3,899	P	P	P	P	P	P	P	P

1031825	COG3682K	gbs0420	copY	Cellular processess	Transcription	CopAB ATPases metal-fist type repressor	1,124	P	P	P	P	P	P	P	P	1,187	P	A	M	A	P	P	A	A
1029796	COG2217P	gbs0421	copA	Metabolism and transport	Inorganic ion transport and metabolism	Copper-exporting ATPase (EC 3.6.3.4)	1,063	P	P	P	P	P	M	P	M	0,638	P	A	P	P	A	A	P	M
1029801	COG2608P	gbs0422	copZ	Metabolism and transport	Inorganic ion transport and metabolism	Copper chaperone copZ	0,920	P	P	P	P	P	P	P	P	0,838	P	P	P	M	P	P	P	P
1030027	COG2860S	gbs0423		Hypothetical		Hypothetical membrane spanning protein	1,687	P	P	P	P	P	P	P	P	1,532	P	A	P	A	A	P	P	M
1031623	COG0561R	gbs0424		Metabolism and transport	Central intermediary metabolism	Hydrolase (HAD superfamily)	1,332	P	P	P	P	P	P	P	P	0,973	P	A	A	A	A	A	A	A
1030017	30258L, COG0	gbs0425	polA	Cellular processess	DNA replication, recombination and repair	DNA polymerase I (EC 2.7.7.7)	1,880	P	P	P	A	P	P	P	P	1,942	P	A	P	A	M	P	P	A
1031621	COG1832R	gbs0426		General function predicted only		CoA binding protein	2,001	P	P	P	A	P	P	P	P	2,137	P	A	P	M	P	P	P	M
1031826	COG0735P	gbs0427	perR	Metabolism and transport	Inorganic ion transport and metabolism	Oxidative stress response regulator BosR	2,950	P	P	P	P	P	P	P	P	1,570	P	P	P	P	P	P	P	P
1029793	-	gbs0428		Transport and binding proteins	Unknown substrate	LPXTG Transporter	0,903	P	P	P	P	P	P	P	P	1,580	P	P	P	P	P	P	P	P
1029791	COG0745TK	gbs0429	resD	Cellular processess	Signal transduction	Two-component response regulator SaeR	2,861	P	P	P	P	P	P	P	P	3,299	P	A	P	A	P	P	A	P
1031620	COG0642T	gbs0430		Cellular processess	Signal transduction	Sensory transduction protein kinase SaeS (EC 2.7.3.-)	2,200	P	P	P	P	P	P	P	P	3,215	P	M	P	P	P	P	P	P
1029798	COG5523S	gbs0431		Hypothetical		Integral membrane protein	3,164	P	P	P	A	P	P	P	A	1,252	P	A	P	A	A	P	A	A
1031618	COG0343J	gbs0432	tgt	Cellular processess	Translation, ribosomal structure and biogenesis	Queuine tRNA-ribosyltransferase (EC 2.4.2.29)	2,371	P	A	A	A	A	A	A	A	2,693	A	A	A	A	A	A	A	A
1030136	COG4357S	gbs0433		Hypothetical		Zinc finger protein	2,161	P	A	A	A	A	A	A	A	3,781	P	A	A	A	A	A	A	A
1031619	COG1268R	gbs0434	bioY	Metabolism and transport	Cofactors, prosthetic groups, and carriers	BioY protein	0,540	P	A	A	P	A	A	P	A	0,982	P	A	A	A	A	A	A	A
1031614	COG1234R	gbs0435		Cellular processess	Toxin production and resistance	Metal-dependent hydrolase (EC 3.-.-.-)	0,559	P	P	P	P	P	P	P	P	1,269	P	P	P	P	P	P	P	P
1031613	COG0590FJ	gbs0436		Cellular processess	Translation, ribosomal structure and biogenesis	tRNA-specific adenosine deaminase (EC 3.5.4.-)	0,675	P	P	P	P	P	P	P	P	1,372	P	A	P	P	P	P	M	P
1031617	COG0166G	gbs0437	pgi	Metabolism and transport	Carbohydrates, organic alcohols, and acids	Glucose-6-phosphate isomerase (EC 5.3.1.9)	0,810	P	P	P	P	P	P	P	P	0,852	P	P	P	P	P	P	P	P
1031610	-	gbs0438		Metabolism and transport	Amino acids, peptides, aminosugars and amines	5-formyltetrahydrofolate cyclo-ligase (EC 6.3.3.2)	1,150	P	P	P	P	P	P	P	P	0,938	P	A	P	P	P	P	P	P
1031616	COG0705R	gbs0439		General function predicted only		Integral membrane protein (Rhomboid family)	1,076	P	P	P	P	P	P	P	P	1,112	P	P	P	P	P	P	P	P
1031615	COG1744R	gbs0440		Metabolism and transport	Purines, pyrimidines, nucleosides, and nucleotides	Nucleoside-binding protein	1,116	P	P	P	P	P	P	P	P	0,737	P	P	P	P	P	P	P	P
1031606	COG1210M	gbs0441	hasC	Cell Envelope	Biosynthesis and degradation of surface polysaccharides and lipopo	UTP--glucose-1-phosphate uridylyltransferase (EC 2.7.7.9)	1,628	P	P	P	P	P	P	P	P	1,459	P	A	P	P	P	P	P	P
1031612	COG0240C	gbs0442	gpsA	Metabolism and transport	Energy production and conversion	Glycerol-3-phosphate dehydrogenase [NAD(P)+] (EC 1.1.1.94)	1,671	P	P	P	P	P	P	P	P	0,985	P	A	P	P	P	P	P	P
1031609	COG0594J	gbs0443	rnpA	Cellular processess	Transcription	Ribonuclease P protein component (EC 3.1.26.5)	3,868	P	P	P	P	P	P	P	P	2,768	P	A	P	P	P	P	P	P
1031611	COG0706U	gbs0444		Cell Envelope	Other	60 kDa inner membrane protein YIDC	2,461	P	P	P	P	P	P	P	P	3,142	P	A	P	P	P	P	A	A
1031607	COG1847R	gbs0445	jag	General function predicted only		Jag protein	1,558	P	P	P	M	P	P	A	P	1,840	P	A	P	M	A	P	P	A
1031602	COG3557J	gbs0446		Cellular processess	Translation, ribosomal structure and biogenesis	Hypothetical cytosolic protein	3,053	P	P	P	P	P	P	P	P	0,931	P	P	P	P	P	P	P	P
1031599	COG2137R	gbs0447	recX	Cellular processess	DNA replication, recombination, and repair	Regulatory protein recX	2,711	P	A	P	P	P	P	P	P	0,963	P	A	P	A	A	P	P	A
1031600	COG2265J	gbs0448		Cellular processess	Translation, ribosomal structure and biogenesis	tRNA (Uracil-5-) -methyltransferase (EC 2.1.1.35)	1,134	P	P	P	P	P	P	P	P	1,200	P	A	P	P	P	P	A	P
1029792	COG2820F	gbs0449		Metabolism and transport	Purines, pyrimidines, nucleosides, and nucleotides	Phosphorylase, Pnp Udp family	2,778	P	P	P	P	P	P	P	P	1,380	P	A	P	P	A	P	A	A
1031595	COG2153R	gbs0450		Metabolism and transport	Central intermediary metabolism	Acetyltransferase, GNAT family	2,271	P	P	P	A	P	P	A	P	2,964	P	A	P	A	A	M	A	A
1031603	COG1404O	gbs0451	cspA ?	Cellular processess	Posttranslational modification, protein turnover, chaperones	LPXTS C5A peptidase precursor (EC 3.4.21.-)	2,224	P	P	P	A	P	P	A	A	0,856	P	A	A	A	A	A	A	A
1031598	COG0463M	gbs0452		Cell Envelope	Biosynthesis and degradation of murein sacculus and peptidoglycan	alpha-L-Rha alpha-1,3-L-rhamnosyltransferase (EC 2.4.1.-)	3,358	P	A	A	A	A	M	A	A	1,728	P	A	A	A	A	A	A	A
1030121	COG0208F	gbs0453	nrdF.1	Metabolism and transport	Purines, pyrimidines, nucleosides, and nucleotides	Ribonucleoside-diphosphate reductase beta chain (EC 1.17.4.1)	7,630	P	M	P	A	P	P	A	P	2,583	P	A	P	P	P	P	P	P
1031597	COG1780F	gbs0454	nrdI	Metabolism and transport	Purines, pyrimidines, nucleosides, and nucleotides	NrdI protein	8,693	P	A	P	A	P	P	A	P	1,965	P	A	P	A	P	P	A	P
1031593	COG0209F	gbs0455	nrdE.1	Metabolism and transport	Purines, pyrimidines, nucleosides, and nucleotides	Ribonucleoside-diphosphate reductase alpha chain (EC 1.17.4.1)	9,182	P	A	P	A	A	A	A	A	2,540	P	A	P	A	A	A	P	A
1031596	-	gbs0456	lrrG	Hypothetical		LPXTS Cell surface protein	22,109	P	A	M	A	A	A	A	A	6,603	P	A	A	A	A	A	A	A
1031594	COG3576R	gbs0457		Hypothetical		Hypothetical cytosolic protein	7,942	P	A	P	A	A	A	A	A	3,691	P	A	P	A	A	P	A	P
1031588	COG3708S	gbs0458		Cellular processess	Transcription	Transcriptional regulator, AraC family	0,974	P	P	P	P	P	P	P	P	2,737	P	A	A	A	A	A	A	A
1031592	COG4367S	gbs0459		Hypothetical		Hypothetical protein	1,220	P	A	P	P	A	A	A	A	3,728	P	A	A	A	A	A	A	A
1031590	COG0599S	gbs0460		Metabolism and transport	Central intermediary metabolism	4-carboxymuconolactone decarboxylase (EC 4.1.1.44)	0,859	P	P	P	P	P	P	P	P	1,588	P	A	P	A	P	A	A	A
1031583	COG1917S	gbs0461		Hypothetical		Hypothetical cytosolic protein	0,887	P	A	P	P	A	P	P	A	1,007	P	A	A	A	A	A	A	A
1031591	COG0789K	gbs0462		Cellular processess	Transcription	Transcriptional regulator, MerR family	1,641	P	A	P	P	A	A	A	A	2,027	P	A	A	A	A	A	A	A
1031582	COG1063ER	gbs0464		Metabolism and transport	Carbohydrates, organic alcohols, and acids	Sorbitol dehydrogenase (EC 1.1.1.14)	0,657	P	A	M	P	A	A	M	M	0,792	P	A	A	A	A	A	A	A
1031584	COG0656R	gbs0466		Metabolism and transport	Central intermediary metabolism	Aldose reductase (EC 1.1.1.21)	0,774	P	P	P	P	P	P	P	P	1,201	P	A	P	P	P	P	A	P
1031586	COG1230P	gbs0467	czcD	Metabolism and transport	Inorganic ion transport and metabolism	Cobalt-zinc-cadmium resistance protein czcD	0,721	P	P	P	P	P	P	P	P	1,319	P	P	P	P	P	P	P	P
1031579	COG1309K	gbs0468		Cellular processess	Transcription	Transcriptional regulator, TetR family	3,328	P	A	A	A	A	A	A	A	1,177	P	A	A	A	A	A	A	A
1031581	COG4753T	gbs0469		Cellular processess	Transcription	Transcriptional regulator, AraC family	1,907	P	A	A	A	A	A	A	A	1,021	A	A	A	A	A	A	A	A
1031585	-	gbs0470	alp2	Hypothetical		LPXTG Cell surface protein	0,342	P	P	P	P	P	P	P	P	0,440	P	A	P	A	A	P	A	A
1031575	COG3077L	gbs0471		Cellular processess	DNA replication, recombination and repair	DNA-damage-inducible protein J	1,146	P	P	P	P	P	P	P	P	0,960	P	P	P	P	P	P	P	P
1031578	-	gbs0472		Hypothetical		Hypothetical cytosolic protein	1,314	P	P	P	P	P	P	P	P	1,114	P	P	P	P	P	P	P	P
1031577	-	gbs0473		Hypothetical		Hypothetical exported protein	0,941	P	P	P	P	P	P	P	P	0,869	P	P	P	P	P	P	P	P
1031567	-	gbs0474		Hypothetical		Hypothetical cytosolic protein	0,768	A	A	A	A	A	A	A	A	0,196	A	A	A	A	A	A	A	A
1031573	COG1396K	gbs0475		Cellular processess	Transcription	Transcriptional regulator, Cro CI family	0,706	P	A	P	A	A	A	A	A	0,299	A	A	A	A	A	A	A	A
1031572	-	gbs0476		Hypothetical		Membrane protein	0,692	A	A	A	A	A	P	A	A	0,888	P	A	A	A	A	A	A	A
1031571	-	gbs0477		Hypothetical		Hypothetical exported protein	0,734	A	A	A	A	A	A	A	A	0,532	A	A	A	A	A	A	A	A
1031580	-	gbs0478		Hypothetical		Hypothetical exported protein	0,566	A	A	A	A	A	A	A	A	0,086	A	A	A	A	A	A	A	A
1031568	-	gbs0479		Hypothetical		LPXTG Cell surface protein	0,183	A	A	A	A	A	A	A	A	0,060	A	A	A	A	A	A	A	A
1031566	-	gbs0480		Hypothetical		Hypothetical membrane spanning protein	0,896	P	P	P	P	P	P	P	P	1,000	P	A	P	A	P	P	P	A
1031576	-	gbs0481		Hypothetical		Hypothetical protein	0,303	M	A	A	P	A	A	A	A	0,260	A	A	A	A	A	A	A	A
1031561	-	gbs0482		Mobile and extrachromosomal elements		DNA integration recombination inversion protein	0,906	P	P	P	P	P	P	P	P	1,190	P	A	A	A	A	A	A	A
1031558	COG1917S	gbs0484		General function predicted only		Putative acetate kinase	1,062	P	P	P	P	P	P	P	P	2,202	P	P	P	P	P	P	P	P
1031569	COG1917S	gbs0485		Hypothetical		Hypothetical cytosolic protein	0,656	P	P	P	P	P	P	P	P	1,671	P	A	P	P	P	P	P	P
1031565	COG0500QR	gbs0486		Metabolism and transport	Secondary metabolites	SAM-dependent methyltransferase	0,598	P	P	P	P	P	P	P	P	1,255	P	P	P	P	P	P	P	P
1031557	-	gbs0487		Hypothetical		Hypothetical protein	0,723	P	P	P	P	P	P	P	P	1,383	P	A	P	P	P	P	P	P
1031560	-	gbs0488		Hypothetical		Hypothetical cytosolic protein	2,008	P	A	A	A	A	A	A	A	2,113	A	A	A	A	A	A	A	A
1031551	COG1670J	gbs0489		Cellular processess	Translation, ribosomal structure and biogenesis	Ribosomal-protein-serine acetyltransferase (EC 2.3.1.-)	1,742	P	A	A	A	A	A	A	A	1,173	A	A	A	A	A	A	A	A
1031559	COG1670J	gbs0490		Cellular processess	Translation, ribosomal structure and biogenesis	Ribosomal-protein-serine acetyltransferase (EC 2.3.1.-)	1,596	P	P	P	A	P	P	A	P	0,795	P	A	M	A	A	P	A	A
1031554	COG0703E	gbs0491		Hypothetical		Hypothetical protein	1,991	P	A	P	A	P	P	A	P	1,472	P	A	A	A	A	P	A	A
1031555	COG0525J	gbs0492	valS	Cellular processess	Translation, ribosomal structure and biogenesis	Valyl-tRNA synthetase (EC 6.1.1.9)	1,112	P	P	P	P	P	P	P	P	0,795	P	A	P	P	P	P	P	P
1031552	-	gbs0493		Hypothetical		Hypothetical protein	0,725	P	P	P	P	P	P	P	P	2,338	P	M	P	P	P	P	P	P
1031550	COG0673R	gbs0494		Metabolism and transport	Central intermediary metabolism	NAD-dependent oxidoreductase	0,998	P	M	P	A	M	P	P	P	0,601	P	P	A	A	A	A	A	A
1031556	COG0598P	gbs0495		Metabolism and transport	Inorganic ion transport and metabolism	Magnesium and cobalt transport protein corA	2,355	P	A	P	M	P	P	A	P	0,706	P	A	P	A	A	M	A	A
1031548	COG2315S	gbs0496		Hypothetical		Hypothetical cytosolic protein	6,937	P	A	A	A	A	A	A	A	1,835	A	A	A	A	A	A	A	A
1031547	COG2502E	gbs0497	asnA	Metabolism and transport	Amino acids, peptides, aminosugars and amines	Aspartate--ammonia ligase (EC 6.3.1.1)	3,034	P	A	A	A	A	A	A	A	3,489	P	A	A	A	A	A	A	A
1031540	-	gbs0498		Metabolism and transport	Energy production and conversion	Thioredoxin	0,681	P	P	P	P	P	P	A	A	0,450	M	A	A	A	A	A	A	A
1031553	COG0742L	gbs0499		Cellular processess	DNA replication, recombination and repair	Methyltransferase (EC 2.1.1.-)	3,066	P	P	P	P	P	P	P	P	2,231	P	A	A	A	A	P	A	A
1031539	-	gbs0500		Hypothetical		Hypothetical protein	3,067	P	P	P	P	P	P	P	P	1,354	P	A	P	M	M	P	P	M
1031546	COG0669H	gbs0501	coaD	Metabolism and transport	Cofactors, prosthetic groups, and carriers	Phosphopantetheine adenylyltransferase (EC 2.7.7.3)	3,034	P	P	P	P	P	P	P	P	1,925	P	A	P	A	P	P	P	A
1031535	COG3480T	gbs0502		Cellular processess	Signal transduction	ATP-dependent endopeptidase Lon (EC 3.4.21.53)	3,291	P	P	P	P	P	P	P	P	1,716	P	A	P	A	P	P	P	P
1031532	COG0737F	gbs0503		Metabolism and transport	Purines, pyrimidines, nucleosides, and nucleotides	5 -nucleotidase (EC 3.1.3.5)	1,052	M	A	A	A	A	A	A	A	0,290	A	A	A	A	A	A	A	A
1031529	-	gbs0504		Hypothetical		Hypothetical cytosolic protein	2,171	P	P	P	P	P	P	P	P	1,626	P	A	P	A	A	A	A	M
1031542	COG0820R	gbs0505		General function predicted only		Radical SAM family enzyme	2,247	P	P	P	A	P	P	P	P	1,047	P	A	P	A	M	M	A	A
1031537	COG4767V	gbs0506		Hypothetical		VanZ family protein	3,849	P	A	A	A	P	M	A	M	1,113	P	A	M	A	A	P	A	A
1031534	COG1132V	gbs0507	atp	Cellular processess	Toxin production and resistance	Multidrug resistance ABC transporter ATP-binding and permease protein	0,794	P	A	A	A	A	A	A	A	0,649	P	A	A	A	A	A	A	A
1031526	COG1132V	gbs0508		Cellular processess	Toxin production and resistance	Multidrug protein lipid ABC transporter family, ATP-binding and permease protein	1,397	A	A	A	A	A	A	A	A	0,651	A	A	A	A	A	A	A	A
1031538	COG0512EH	gbs0509	trpG	Metabolism and transport	Cofactors, prosthetic groups, and carriers	Anthranilate synthase component II (EC 4.1.3.27) Para-aminobenzoate synthase	1,484	P	A	P	P	P	P	P	A	0,989	P	A	P	A	A	A	A	A
1031533	COG1268R	gbs0510		Metabolism and transport	Cofactors, prosthetic groups, and carriers	BioY protein	1,200	A	A	A	A	A	A	A	A	1,110	A	A	A	A	A	A	A	A
1031523	COG0502H	gbs0511	bioB	Metabolism and transport	Cofactors, prosthetic groups, and carriers	Biotin synthase (EC 2.8.1.6)	0,431	A	A	A	A	A	A	M	A	0,498	A	A	A	A	A	A	A	A
1031531	-	gbs0512		Hypothetical		Hypothetical protein	0,439	A	A	A	A	A	A	A	A	0,311	A	A	A	A	A	A	A	A
1031527	COG0318IQ	gbs0514		Metabolism and transport	Fatty acid and phospholipids	Long-chain-fatty-acid--CoA ligase (EC 6.2.1.3)	0,370	A	A	A	A	A	A	A	A	0,425	A	P	A	A	A	A	A	A
1031525	COG0177L	gbs0515	nth	Cellular processess	DNA replication, recombination and repair	Endonuclease III (EC 4.2.99.18)	0,761	P	P	P	P	P	P	P	P	0,758	P	P	P	P	A	P	P	A
1031520	-	gbs0516		General function predicted only		Type 4 prepilin-like proteins leader peptide processing enzyme	0,289	A	A	A	A	A	A	A	A	0,017	A	A	A	A	A	A	A	A
1031528	-	gbs0517		Hypothetical		Hypothetical cytosolic protein	2,181	P	P	P	P	P	P	P	P	2,187	P	P	P	P	P	P	P	P
1031524	COG1940KG	gbs0518	glcK	Metabolism and transport	Carbohydrates, organic alcohols, and acids	Glucokinase (EC 2.7.1.2)	1,191	P	P	P	P	P	P	P	P	1,501	P	M	P	P	P	P	P	P

1031517	COG0607P	gbs0519		Metabolism and transport	Inorganic ion transport and metabolism	Rhodanese-related sulfurtransferases	1,214	P	P	P	P	P	P	P	P	1,471	P	P	P	P	P	P	P	P
1031521	COG1217T	gbs0520	typA	Cellular processess	Signal transduction	GTP-binding protein TypA BipA	2,771	P	P	P	A	P	P	P	P	0,639	P	A	P	A	P	P	P	P
1031519	-	gbs0521		Hypothetical		Hypothetical membrane spanning protein	2,188	P	P	P	P	P	P	P	P	0,623	P	A	P	P	P	P	P	P
1031514	COG0771M	gbs0522	murD	Cell Envelope	Biosynthesis and degradation of murein sacculus and peptidoglycan	UDP-N-acetylmuramoylalanine--D-glutamate ligase (EC 6.3.2.9)	1,316	P	P	P	P	P	M	P	P	1,554	P	A	P	A	A	A	A	A
1031516	COG0707M	gbs0523	murG	Cell Envelope	Biosynthesis and degradation of murein sacculus and peptidoglycan	UDP-N-acetylglucosamine--N-acetylmuramyl-(pentapeptide) pyrophosphoryl-unde	1,505	P	M	A	A	A	A	A	A	0,897	P	M	M	A	A	A	A	A
1031512	COG1589M	gbs0524	divIB	Cellular processess	Cell division	Cell-division initiation protein DivIB	1,633	P	P	P	P	P	P	P	P	1,049	P	A	P	P	P	P	P	P
1031518	COG0849D	gbs0525	ftsA	Cellular processess	Cell division	Cell division protein ftsA	2,966	P	P	P	P	P	P	P	P	1,480	P	P	P	P	P	P	P	P
1031509	COG0206D	gbs0526	ftsZ	Cellular processess	Cell division	Cell division protein ftsZ	1,679	P	P	P	P	P	P	P	P	1,154	P	P	P	P	P	P	P	P
1031508	COG0325R	gbs0527		General function predicted only		Pyridoxal-5 -phosphate family protein	3,842	P	P	P	P	P	P	P	P	1,736	P	P	P	P	P	P	P	P
1031513	COG1799S	gbs0528	ylmF	Hypothetical		Hypothetical cytosolic protein	2,985	P	M	P	A	P	P	P	P	1,938	P	P	P	A	P	P	P	P
1031506	-	gbs0529		Hypothetical		Integral membrane protein	3,181	P	P	P	P	P	P	P	P	1,582	P	P	P	P	P	P	P	P
1031510	COG2302S	gbs0530		General function predicted only		RNA binding protein	4,035	P	P	P	P	P	P	P	P	1,415	P	P	P	P	P	P	P	P
1031511	COG3599D	gbs0531	divIVAS	Cellular processess	Cell division	Cell division initiation protein DivIVA	2,743	P	P	P	P	P	P	P	P	1,268	P	P	P	P	P	P	P	P
1031507	COG0060J	gbs0532	ileS	Cellular processess	Translation, ribosomal structure and biogenesis	Isoleucyl-tRNA synthetase (EC 6.1.1.5)	1,527	P	P	P	P	P	P	P	P	1,356	P	P	P	P	P	P	P	P
1031505	-	gbs0533		Hypothetical		Hypothetical cytosolic protein	0,256	P	P	P	P	P	P	P	P	0,719	P	P	P	P	P	P	P	P
1031515	COG0494LR	gbs0534	mutT	General function predicted only		Mutator mutT protein (7,8-dihydro-8-oxoguanine-triphosphatase) (EC 3.6.1.-)	0,734	P	P	P	P	P	P	P	P	1,579	P	A	A	A	A	A	A	A
1031503	COG0542O	gbs0535	clpE	Cellular processess	Posttranslational modification, protein turnover, chaperones	ATP-dependent endopeptidase clp ATP-binding subunit clpE	0,952	P	P	P	P	P	P	P	P	0,722	P	A	P	P	P	P	P	P
1031504	-	gbs0536		Hypothetical		Hypothetical cytosolic protein	2,276	P	M	P	M	P	P	P	P	0,783	P	A	A	A	A	P	P	A
1031502	COG0765E	gbs0537	artQ	Metabolism and transport	Amino acids, peptides, aminosugars and amines	Arginine transport system permease protein artQ	1,233	P	P	P	A	P	P	P	P	0,509	P	A	P	A	A	P	M	A
1031497	COG1126E	gbs0538	artP	Metabolism and transport	Amino acids, peptides, aminosugars and amines	Arginine transport ATP-binding protein artP	0,843	P	P	P	P	P	P	P	P	0,679	P	A	P	A	P	P	M	P
1031498	COG1109G	gbs0539		Metabolism and transport	Carbohydrates, organic alcohols, and acids	Phosphoglucomutase (EC 5.4.2.2) Phosphomannomutase (EC 5.4.2.8)	2,312	P	P	P	P	P	P	P	P	1,934	P	M	P	P	P	P	P	P
1031499	COG0190H	gbs0540	folD	Metabolism and transport	Cofactors, prosthetic groups, and carriers	Methylenetetrahydrofolate dehydrogenase (NADP+) (EC 1.5.1.5) Methenyltetrahy	0,880	P	P	P	P	P	P	P	P	0,859	P	A	P	P	P	P	P	P
1031492	COG0063G	gbs0541	yjeF	Metabolism and transport	Carbohydrates, organic alcohols, and acids	Sugar kinase	1,597	P	A	A	A	A	A	A	A	2,037	M	A	A	A	A	A	A	A
1031490	COG1570L	gbs0542	xseA	Cellular processess	DNA replication, recombination and repair	Exodeoxyribonuclease VII large subunit (EC 3.1.11.6)	1,551	P	M	P	P	M	M	M	P	1,226	P	A	P	P	P	P	M	A
1031496	COG1722L	gbs0543	xseB	Cellular processess	DNA replication, recombination and repair	Exodeoxyribonuclease VII small subunit (EC 3.1.11.6)	1,375	P	A	P	P	P	P	A	A	0,828	P	A	P	P	P	P	P	A
1031500	COG0142H	gbs0544	fps	Metabolism and transport	Cofactors, prosthetic groups, and carriers	Dimethylallyltransferase (EC 2.5.1.1) Geranyltranstransferase (EC 2.5.1.10)	1,833	P	A	A	A	A	A	A	A	1,714	P	A	A	A	A	A	A	A
1031493	COG1189J	gbs0545	hemA	Cellular processess	Translation, ribosomal structure and biogenesis	Hemolysin	1,763	P	P	P	P	M	P	P	P	1,297	P	P	P	P	P	P	P	M
1031491	COG1438K	gbs0546	argR1	Cellular processess	Transcription	Arginine repressor, argR	2,129	P	P	P	P	P	P	P	P	1,414	P	A	P	P	P	P	P	P
1031484	COG0497L	gbs0547	recN	Cellular processess	DNA replication, recombination and repair	DNA repair protein recN	1,759	P	P	P	P	P	P	P	P	0,868	P	P	P	P	P	P	P	P
1031489	COG1307S	gbs0548	degV	Metabolism and transport	Fatty acid and phospholipids	Fatty acid-binding protein, DegV family	1,897	P	P	P	P	P	P	P	P	1,191	P	P	P	P	P	P	P	P
1031488	COG2755E	gbs0549		Metabolism and transport	Fatty acid and phospholipids	Lipase Acylhydrolase with GDSL-like motif	2,339	P	A	A	A	A	A	A	A	0,941	P	A	A	A	A	A	A	A
1031479	-	gbs0550	yfaA	Hypothetical		Hypothetical membrane associated protein	2,584	P	A	A	A	A	A	A	A	1,368	P	A	P	A	M	P	A	A
1031763	-	gbs0551		Cellular processess	DNA replication, recombination and repair	DNA-binding protein HU	0,414	P	P	P	P	P	P	P	P	0,584	P	P	P	P	P	P	P	P
1031782	-	gbs0552		Hypothetical		Hypothetical protein	1,201	P	P	P	P	P	P	P	P	1,746	P	P	P	A	P	P	P	P
1031236	COG0167F	gbs0553	pyrD	Metabolism and transport	Purines, pyrimidines, nucleosides, and nucleotides	Dihydroorotate dehydrogenase (EC 1.3.3.1)	1,991	P	P	P	P	P	P	P	P	0,789	P	P	P	A	P	P	P	P
1031697	COG2348V	gbs0554		Cellular processess	Toxin production and resistance	Factor essential for expression of methicillin resistance	1,442	P	A	A	A	P	A	P	A	1,247	P	A	P	A	A	A	A	A
1031713	COG2348V	gbs0555	murM	Cell Envelope	Biosynthesis and degradation of murein sacculus and peptidoglycan	UDP-N-acetylmuramoylpentapeptide-lysine N(6)-alanyltransferase (EC 2.3.2.10)	2,070	P	P	M	A	P	P	P	P	1,199	P	A	A	A	A	A	M	A
1030787	COG2348V	gbs0556		Cell Envelope	Biosynthesis and degradation of murein sacculus and peptidoglycan	UDP-N-acetylmuramoylpentapeptide-lysine N(6)-alanyltransferase (EC 2.3.2.10)	1,518	P	A	P	A	P	A	A	A	0,937	P	A	A	M	A	A	A	A
1031795	COG0561R	gbs0557		Metabolism and transport	Central intermediary metabolism	Hydrolase (HAD superfamily)	2,831	P	A	P	A	P	M	A	A	1,354	P	A	P	A	A	A	P	A
1030920	COG1078R	gbs0558		General function predicted only		dGTP triphosphohydrolase	1,629	P	A	A	A	A	A	A	A	1,653	P	A	A	A	A	A	A	A
1031474	-	gbs0559		Hypothetical		Hypothetical cytosolic protein	1,540	P	M	P	P	P	P	P	P	1,580	P	A	P	A	M	P	A	A
1030837	COG0474P	gbs0560	pacL	Metabolism and transport	Inorganic ion transport and metabolism	Calcium-transporting ATPase (EC 3.6.3.8)	1,066	P	P	P	P	P	P	P	P	0,612	P	P	P	P	P	P	P	P
1031766	COG1409R	gbs0561		Metabolism and transport	Central intermediary metabolism	Phosphoesterase	2,816	P	P	A	P	A	M	A	A	1,230	P	A	A	A	A	A	A	A
1031434	-	gbs0562		Metabolism and transport	Carbohydrates, organic alcohols, and acids	Fructose-1,6-bisphosphatase (EC 3.1.3.11)	1,594	P	A	A	A	A	A	A	A	0,698	P	A	A	A	A	A	A	A
1031105	COG1600C	gbs0563		Metabolism and transport	Energy production and conversion	iron-sulfur cluster-binding protein	2,889	P	A	A	A	A	A	A	A	2,021	P	A	A	A	A	A	A	A
1031794	COG1186J	gbs0564	prfB	Cellular processess	Translation, ribosomal structure and biogenesis	Bacterial Peptide Chain Release Factor 2 (RF-2)	4,454	P	P	P	A	P	P	A	P	1,666	P	P	P	P	A	P	A	A
1030257	COG2884D	gbs0565	ftsE	Cellular processess	Cell division	Cell division ATP-binding protein ftsE	2,820	P	P	P	P	P	P	P	P	1,529	P	P	P	P	P	P	P	P
1031425	COG2177D	gbs0566	ftsX	Cellular processess	Cell division	Cell division protein ftsX	2,131	P	P	P	M	P	P	P	P	1,982	P	A	P	M	P	P	M	P
1031796	COG0596R	gbs0567		General function predicted only		CARBOXYMETHYLENEBUTENOLIDASE-RELATED PROTEIN	2,172	P	A	A	A	A	A	A	A	1,634	P	A	A	A	A	A	A	A
1031200	COG0491R	gbs0568		General function predicted only		Hydroxyacylglutathione hydrolase (EC 3.1.2.6)	1,571	P	A	P	A	P	P	A	M	0,876	P	A	P	M	A	P	A	A
1031652	COG1028IQR	gbs0569		Metabolism and transport	Fatty acid and phospholipids	(R,R)-butanediol dehydrogenase (EC 1.1.1.4) Acetoin dehydrogenase (EC 1.1.1.5	0,327	P	P	P	P	P	P	P	P	0,512	P	P	P	P	P	P	P	P
1031729	0847L, COG11	gbs0570	dinG	Cellular processess	DNA replication, recombination and repair	ATP-dependent helicase, DinG family	2,088	P	A	P	A	P	P	M	A	1,628	P	A	P	A	M	P	A	A
1031689	COG0436E	gbs0571	aspC	Metabolism and transport	Amino acids, peptides, aminosugars and amines	Aspartate aminotransferase (EC 2.6.1.1)	2,843	P	P	P	A	P	P	P	P	1,041	P	M	P	A	P	P	P	A
1031758	COG0017J	gbs0572	asnS	Cellular processess	Translation, ribosomal structure and biogenesis	Asparaginyl-tRNA synthetase (EC 6.1.1.22)	3,014	P	P	P	A	P	P	A	A	1,237	P	A	P	A	P	P	P	A
1031767	-	gbs0573		Hypothetical		Integral membrane protein	3,366	P	P	P	A	M	P	A	A	2,396	P	A	A	A	A	A	A	A
1031428	COG1957F	gbs0574		Metabolism and transport	Purines, pyrimidines, nucleosides, and nucleotides	Inosine-uridine preferring nucleoside hydrolase (EC 3.2.2.1)	2,256	P	P	P	P	P	P	P	P	1,624	P	A	P	P	A	P	A	P
1031406	COG1764O	gbs0575		Cellular processess	Posttranslational modification, protein turnover, chaperones	Osmotically inducible protein C	1,532	P	P	P	P	P	P	P	P	1,871	P	A	P	A	A	M	A	A
1031160	COG1660R	gbs0576		General function predicted only		ATP-binding protein (contains P-loop)	0,988	P	P	P	P	P	P	P	P	1,171	P	P	P	P	P	P	P	P
1031710	-	gbs0577		Hypothetical		Hypothetical membrane associated protein	0,901	P	P	P	P	P	P	P	P	0,974	P	A	P	P	P	P	P	P
1031411	COG1481S	gbs0578		Hypothetical		Hypothetical cytosolic protein	1,965	P	P	P	P	P	P	P	P	1,869	P	A	P	M	P	P	A	P
1031472	-	gbs0579	pepD	Cellular processess	Posttranslational modification, protein turnover, chaperones	Dipeptidase A (EC 3.4.13.-)	1,431	P	M	P	A	P	P	M	P	1,829	P	A	A	A	A	P	A	A
1031163	i0803P, COG3	gbs0580		Metabolism and transport	Inorganic ion transport and metabolism	High-affinity zinc uptake system protein znuA precursor Hypothetical exported pro	1,890	P	P	P	A	P	P	P	A	0,531	P	A	P	A	P	P	P	A
1031350	COG0254J	gbs0581	rpmE	Cellular processess	Translation, ribosomal structure and biogenesis	LSU ribosomal protein L31P	0,831	P	P	P	P	P	P	P	P	0,618	P	P	P	P	P	P	P	P
1031269	COG0618R	gbs0582		Metabolism and transport	Central intermediary metabolism	Phosphoesterase, DHH family protein	0,978	P	P	P	P	P	P	P	P	0,615	P	A	A	A	A	A	A	A
1031736	COG1816F	gbs0583	add	Metabolism and transport	Purines, pyrimidines, nucleosides, and nucleotides	Adenosine deaminase (EC 3.5.4.4)	3,384	P	P	P	A	P	P	P	A	0,920	P	A	P	A	A	A	A	A
1030782	COG0716C	gbs0584		Metabolism and transport	Energy production and conversion	Flavodoxin	2,811	P	P	P	P	P	P	P	P	1,438	P	P	P	P	P	P	P	P
1030889	-	gbs0585		Metabolism and transport	Amino acids, peptides, aminosugars and amines	Chorismate mutase (EC 5.4.99.5)	4,924	P	A	M	A	A	P	A	A	2,472	P	A	P	A	P	P	A	A
1031186	COG0038P	gbs0586		Metabolism and transport	Inorganic ion transport and metabolism	Chloride channel protein	2,295	P	A	P	A	A	P	A	M	1,400	P	A	P	A	A	A	A	A
1031049	-	gbs0587	rplS	Cellular processess	Translation, ribosomal structure and biogenesis	LSU ribosomal protein L19P	1,607	P	P	P	P	P	P	P	P	0,714	P	P	P	P	P	P	P	P
1030772	COG2963L	gbs0589		Mobile and extrachromosomal elements		Transposase	3,729	P	A	A	A	A	A	A	A	0,753	P	A	A	A	A	A	M	A
1031302	-	gbs0593		Hypothetical		Hypothetical protein	1,205	A	A	A	A	A	A	A	A	0,185	A	A	A	A	A	A	A	A
1031026	-	gbs0594		Transport and binding proteins	Unknown substrate	ABC transporter permease protein	3,745	P	P	P	P	P	P	P	P	2,257	P	A	P	A	P	P	A	A
1031257	COG1136V	gbs0595		Transport and binding proteins	Unknown substrate	ABC transporter ATP-binding protein	2,722	P	P	P	P	P	P	P	P	1,313	P	P	P	P	P	P	P	P
1031334	COG0577V	gbs0596		Transport and binding proteins	unknown substrate	ABC transporter permease protein	3,059	P	P	P	P	P	P	P	P	1,802	P	A	P	P	P	P	M	P
1031326	COG0745TK	gbs0597	vcnS	hypothetical		Hypothetical protein	4,179	P	P	P	A	P	P	A	P	0,763	P	A	A	A	A	P	A	A
1031040	COG0642T	gbs0598	cnR /ihk	Cellular processess	Signal transduction	Phosphate regulon sensor protein phoR (EC 2.7.3.-)	5,325	P	A	P	A	P	P	A	A	2,412	P	A	A	A	A	A	A	A
1031031	-	gbs0600		Hypothetical		Hypothetical protein	0,358	P	P	P	P	P	P	P	P	0,717	P	P	P	P	P	P	P	P
1031223	-	gbs0601	mreB	Cell Envelope	Biosynthesis and degradation of murein sacculus and peptidoglycan	Rod shape-determining protein rodA	2,413	P	P	A	A	P	A	A	A	0,750	P	A	A	A	A	A	A	P
1031348	COG0546R	gbs0602	pgp	Metabolism and transport	Central intermediary metabolism	Phosphatase	4,343	P	P	P	P	P	P	P	P	1,895	P	P	P	P	P	P	P	P
1031332	COG0187L	gbs0603	gyrB	Cellular processess	DNA replication, recombination and repair	DNA gyrase subunit B (EC 5.99.1.3)	1,943	P	P	P	P	P	P	P	P	1,571	P	P	P	P	P	P	P	P
1031401	COG4477D	gbs0604		Cellular processess	Cell division	Septation ring formation regulator	2,364	P	P	P	P	P	P	P	P	1,598	P	P	P	P	P	P	P	P
1031358	COG0560E	gbs0605		Metabolism and transport	Amino acids, peptides, aminosugars and amines	Phosphoserine phosphatase (EC 3.1.3.3)	1,413	P	P	P	A	P	P	P	P	1,560	P	A	A	A	A	A	A	A
1031351	COG0494LR	gbs0606		Cellular processess	DNA replication, recombination and repair	Phosphohydrolase (MutT nudix family protein)	1,113	P	P	P	P	P	P	P	P	1,775	P	A	P	A	A	A	A	A
1031360	COG5506S	gbs0607		Hypothetical		Hypothetical cytosolic protein	1,148	P	P	P	P	P	P	P	P	1,902	P	A	P	A	P	P	A	P
1031354	COG0148G	gbs0608	eno	Metabolism and transport	Carbohydrates, organic alcohols, and acids	Enolase (EC 4.2.1.11)	0,500	P	P	P	P	P	P	P	P	0,463	P	P	P	P	P	P	P	P
1031375	-	gbs0609		General function predicted only		Streptodornase (EC 3.1.21.1)	1,192	A	A	A	A	A	A	A	A	0,298	A	A	A	A	A	A	A	A
1031372	COG0128E	gbs0610	aroA	Metabolism and transport	Amino acids, peptides, aminosugars and amines	3-phosphoshikimate 1-carboxyvinyltransferase (EC 2.5.1.19)	1,394	P	P	P	P	P	P	P	P	1,642	P	A	P	P	P	P	A	P
1031039	COG0703E	gbs0611	aroK	Metabolism and transport	Amino acids, peptides, aminosugars and amines	Shikimate kinase (EC 2.7.1.71)	2,122	P	P	P	P	P	P	P	P	1,260	P	A	P	P	P	P	P	P
1030877	COG1316K	gbs0612		Cellular processess	Transcription	Transcriptional regulator, LytR family	1,292	P	A	P	P	P	P	P	P	0,962	P	P	P	A	A	A	P	A
1030885	COG2265J	gbs0613		Cellular processess	Translation, ribosomal structure and biogenesis	23S rRNA m(5)U 1939 methyltransferase (EC 2.1.1.-)	4,143	P	A	A	A	A	A	A	A	2,092	P	A	A	A	A	A	A	A
1030908	-	gbs0614		Hypothetical		Hypothetical protein	3,099	P	A	P	A	P	A	A	A	1,796	P	A	P	A	A	A	P	A
1031260	COG3700R	gbs0615		General function predicted only		Class B acid phosphatase (EC 3.1.3.2)	0,314	P	P	P	P	P	P	P	P	1,089	P	P	P	P	P	P	P	P
1030879	COG4283S	gbs0616		Hypothetical		Hypothetical cytosolic protein	0,547	P	P	P	P	P	P	P	P	1,670	P	P	P	P	P	P	P	P
1031170	-	gbs0617		Hypothetical		Hypothetical protein	7,212	A	A	A	A	A	A	A	A	1,826	A	A	A	A	A	A	A	A
1031249	COG1309K	gbs0618		Cellular processess	Transcription	Transcriptional regulator, TetR family	2,161	P	A	A	A	A	A	A	A	0,309	P	A	A	A	M	A	A	A

1030880	-	gbs0619		Hypothetical		Hypothetical protein	1,055	A	A	A	A	A	A	A	A	0,621	P	A	A	A	A	A	A	A
1031243	COG2963L	gbs0621		Mobile and extrachromosomal elements		Transposase	3,062	P	A	P	P	P	P	P	P	1,418	P	P	P	P	P	P	P	P
1031262	-	gbs0622		Hypothetical		Hypothetical protein	1,078	P	A	A	A	A	A	A	A	0,880	A	A	A	A	A	A	A	A
1031121	-	gbs0625		Cellular processess	Posttranslational modification, protein turnover, chaperones	33 kDa chaperonin	2,020	P	P	P	P	P	P	P	P	1,018	P	P	P	P	P	P	P	P
1030901	-	gbs0627	araC	Cellular processess	Transcription	Transcriptional regulator, AraC family	1,036	P	P	P	P	P	P	P	P	1,023	P	P	P	P	P	P	P	P
1031310	COG4932M	gbs0628		Hypothetical		IPXTG Hypothetical protein	0,343	P	A	M	M	M	P	M	P	0,230	P	A	P	M	A	P	A	A
1031068	COG4932M	gbs0629		Cellular processess	Posttranslational modification, protein turnover, chaperones	IPXTG Hypothetical protein	1,063	A	A	A	A	A	A	A	A	0,141	A	A	A	A	A	A	A	A
1031473	COG3764M	gbs0630		Cellular processess	Posttranslational modification, protein turnover, chaperones	Sortase	0,307	A	A	A	A	A	A	A	A	0,079	A	A	A	A	A	A	A	A
1031341	COG3764M	gbs0631		Cellular processess	Posttranslational modification, protein turnover, chaperones	Sortase	0,485	A	A	A	P	A	A	A	A	0,242	M	A	A	A	A	A	A	A
1031148	COG4932M	gbs0632		Cell Envelope	Other	FPKTG Collagen adhesion protein	0,097	A	A	A	A	A	A	A	A	0,559	A	A	A	A	A	A	A	A
1030835	COG4932M	gbs0636		Metabolism and transport	Energy production and conversion	Collagen adhesion protein	0,542	A	A	A	A	A	A	A	A	0,127	A	A	A	A	A	A	A	A
1031305	-	gbs0638		Hypothetical		Hypothetical protein	4,509	P	A	P	P	P	P	P	P	2,005	P	A	P	A	A	A	A	P
1031469	-	gbs0639		Hypothetical		Hypothetical protein	1,635	A	A	A	A	A	A	A	A	0,064	A	A	A	A	A	A	A	A
1030886	COG1680V	gbs0640		Cellular processess	Toxin production and resistance	Beta-lactamase family protein	2,371	P	P	A	A	P	P	A	M	0,747	P	A	P	A	A	P	A	A
1030753	COG4586R	gbs0641	drrA	General function predicted only		Daunorubicin resistance ATP-binding protein drrA	3,239	P	A	A	A	A	A	A	A	1,677	P	M	P	P	P	P	P	P
1031475	COG4587R	gbs0642		General function predicted only		Daunorubicin resistance transmembrane protein	5,732	P	A	A	A	A	A	A	A	1,768	P	A	P	A	A	P	A	P
1031396	COG3694R	gbs0643		Transport and binding proteins	Unknown substrate	ABC transporter permease protein	2,608	P	A	A	A	A	A	A	A	1,748	P	A	M	A	A	P	A	A
1031464	-	gbs0644	cylX	Hypothetical		Hypothetical protein	0,938	P	P	P	P	P	P	P	P	2,654	P	P	P	P	P	P	P	P
1031688	COG0331I	gbs0645	cylD	Metabolism and transport	Fatty acid and phospholipids	Malonyl-CoA-[acyl-carrier-protein] transacylase (EC 2.3.1.39)	0,653	P	P	P	P	P	P	P	P	1,817	P	P	P	P	P	P	P	P
1031686	COG1028IQR	gbs0646	cylG	Metabolism and transport	Fatty acid and phospholipids	3-oxoacyl-[acyl-carrier protein] reductase (EC 1.1.1.100)	0,635	P	P	P	P	P	P	P	P	1,926	P	P	P	P	P	P	P	P
1031687	COG0236IQ	gbs0647	acpC	Metabolism and transport	Fatty acid and phospholipids	Acyl carrier protein	0,829	P	P	P	P	P	P	P	P	2,304	P	M	P	P	P	P	P	P
1031692	COG0764I	gbs0648	cylZ	Metabolism and transport	Fatty acid and phospholipids	(3R)-hydroxymyristoyl-[acyl carrier protein] dehydratase (EC 4.2.1.-)	0,844	P	P	P	P	P	P	P	P	1,700	P	P	P	M	P	P	A	P
1031693	COG1131V	gbs0649	cylA	Transport and binding proteins	Unknown substrate	Hemolysin export ABC transporter ATP-binding protein	1,274	P	P	P	P	P	P	P	P	1,984	P	P	P	P	P	P	P	P
1031691	-	gbs0650	cylB	Transport and binding proteins	Unknown substrate	Hypothetical ABC transporter permease protein	1,782	P	P	P	P	P	P	P	P	2,377	P	A	P	A	P	P	A	M
1031430	-	gbs0651	cylE	Hypothetical		Hypothetical protein	1,877	P	P	P	P	P	P	P	P	2,034	P	M	P	A	P	P	P	P
1031690	COG0404E	gbs0652	cylF	Metabolism and transport	Amino acids, peptides, aminosugars and amines	Aminomethyltransferase (EC 2.1.2.10)	1,537	P	P	P	P	P	P	P	P	2,556	P	P	P	P	P	P	P	P
1031699	COG0304IQ	gbs0653	cylI	Metabolism and transport	Fatty acid and phospholipids	3-oxoacyl-[acyl-carrier-protein] synthase (EC 2.3.1.41) Chain length factor	1,957	P	P	P	P	P	P	P	P	1,959	P	P	P	P	P	P	P	P
1031696	COG1819GC	gbs0654	cylJ	Metabolism and transport	Carbohydrates, organic alcohols, and acids	UDP glycosyltransferase (EC 2.4.1.-)	2,798	P	P	P	P	P	P	P	P	2,845	P	P	P	A	P	P	P	P
1031698	-	gbs0655	cylK	Hypothetical		Hypothetical protein	3,902	P	P	P	P	P	P	P	A	3,087	P	P	P	P	P	P	P	P
1031700	COG0577V	gbs0657		Transport and binding proteins	Unknown substrate	ABC transporter permease protein	1,690	A	A	A	A	A	A	A	A	0,405	A	A	A	A	A	A	A	A
1031695	-	gbs0658		Hypothetical		Hypothetical protein	0,396	A	A	A	A	A	A	A	A	0,805	A	A	A	A	A	A	A	A
1031701	COG1136V	gbs0659		Transport and binding proteins	Unknown substrate	ABC transporter ATP-binding protein	0,456	A	A	A	A	A	A	A	A	0,079	A	A	A	A	A	A	A	A
1031115	COG4529S	gbs0660		Hypothetical		Hypothetical exported protein	1,329	P	A	P	A	M	A	A	A	0,919	P	A	A	A	A	A	A	A
1031706	-	gbs0661		Cellular processess	DNA transformation	DNA-entry nuclease (EC 3.1.30.-)	0,636	P	P	P	P	P	P	P	P	2,443	P	P	P	P	P	P	P	P
1031702	COG0586S	gbs0662		General function predicted only		DedA family protein	1,269	P	M	A	A	A	P	A	P	1,291	A	A	A	A	A	A	A	A
1031703	COG4619R	gbs0663		Transport and binding proteins	Unknown substrate	ABC transporter ATP-binding protein	0,606	P	A	P	A	P	M	P	P	0,617	A	A	A	A	A	A	A	A
1031704	COG0390R	gbs0664		Transport and binding proteins	Unknown substrate	ABC transporter permease protein	0,674	P	A	M	A	A	A	A	A	0,804	A	A	A	A	A	A	A	A
1031705	-	gbs0665		Hypothetical		Hypothetical protein	0,780	P	A	P	P	P	P	P	P	0,557	P	A	A	A	A	A	A	A
1031707	COG0583K	gbs0666		Cellular processess	Transcription	Transcriptional regulators, LysR family	0,962	P	A	P	P	P	P	P	P	1,125	P	A	A	A	A	A	A	A
1031052	COG3641R	gbs0667		Hypothetical		Hypothetical membrane spanning protein	1,105	P	P	P	P	P	P	P	P	0,316	P	A	P	P	P	P	P	P
1031708	COG1052CHR	gbs0668	ddh	Metabolism and transport	Central intermediary metabolism	D-2-hydroxyacid dehydrogenase (EC 1.1.1.-)	1,304	P	P	P	P	P	P	P	P	0,315	P	A	P	P	P	P	P	M
1031711	COG2211G	gbs0669		Metabolism and transport	Carbohydrates, organic alcohols, and acids	SUGAR SODIUM SYMPORTER	0,394	A	A	A	A	A	A	A	A	0,372	A	A	A	A	A	A	A	A
1031712	COG0524G	gbs0670		Metabolism and transport	Carbohydrates, organic alcohols, and acids	2-dehydro-3-deoxygluconokinase (EC 2.7.1.45)	0,324	A	A	A	A	A	A	A	A	0,056	A	A	A	A	A	A	A	A
1031721	COG3250G	gbs0671	lacZ	Metabolism and transport	Carbohydrates, organic alcohols, and acids	Beta-glucuronidase (EC 3.2.1.31)	1,882	P	A	A	A	A	A	A	A	0,833	A	A	A	A	A	A	A	A
1031715	COG2186K	gbs0672		Cellular processess	Transcription	Transcriptional regulator, GntR family	0,720	A	A	A	A	A	A	A	A	1,003	A	A	A	A	A	A	A	A
1031709	COG0800G	gbs0673		Metabolism and transport	Carbohydrates, organic alcohols, and acids	4-Hydroxy-2-oxoglutarate aldolase (EC 4.1.3.16) 2-dehydro-3-deoxyphosphogluc	0,394	P	P	P	M	P	A	P	A	0,802	P	A	M	M	A	A	A	A
1030888	COG1904G	gbs0674		Metabolism and transport	Carbohydrates, organic alcohols, and acids	Uronate isomerase (EC 5.3.1.12)	0,935	P	A	A	A	A	A	A	A	1,329	P	A	A	A	A	A	A	A
1031717	COG1312G	gbs0675		Metabolism and transport	Carbohydrates, organic alcohols, and acids	Mannonate dehydratase (EC 4.2.1.8)	0,749	A	A	A	A	A	A	A	A	0,496	A	A	A	A	A	A	A	A
1031719	COG1028IQR	gbs0676		Metabolism and transport	Carbohydrates, organic alcohols, and acids	Fructuronate reductase (EC 1.1.1.57)	0,529	P	M	P	A	P	P	A	A	0,566	P	A	P	P	P	P	A	A
1031716	COG0546R	gbs0677		Metabolism and transport	Central intermediary metabolism	Phosphoglycolate phosphatase (EC 3.1.3.18)	1,005	P	A	A	A	A	A	A	A	0,777	P	A	A	P	A	A	A	A
1031371	-	gbs0678		Metabolism and transport	Carbohydrates, organic alcohols, and acids	Beta-N-acetylhexosaminidase (EC 3.2.1.52)	0,567	A	A	A	A	A	A	A	A	3,168	A	A	A	A	A	A	A	A
1031724	COG0006E	gbs0679	pepQ	Metabolism and transport	Amino acids, peptides, aminosugars and amines	Xaa-Pro aminopeptidase (EC 3.4.11.9)	0,760	P	P	P	P	P	P	P	P	1,690	P	A	P	P	P	P	P	P
1031718	COG1609K	gbs0680	ccpA	Cellular processess	Transcription	Catabolite control protein A	1,397	P	P	P	P	P	P	P	P	0,778	P	P	P	P	P	P	P	P
1031212	-	gbs0681		Metabolism and transport	Carbohydrates, organic alcohols, and acids	Alpha-amylase (EC 3.2.1.1)	1,295	P	P	P	A	P	P	P	P	1,135	P	A	P	P	P	P	P	P
1031321	COG0438M	gbs0682		Metabolism and transport	Central intermediary metabolism	Glycosyltransferase (EC 2.4.1.-)	2,410	P	P	P	P	P	P	P	P	1,723	P	A	P	A	A	P	P	A
1031726	COG0438M	gbs0683		Metabolism and transport	Central intermediary metabolism	1,2-diacylglycerol 3-glucosyltransferase (EC 2.4.1.157)	2,146	P	P	P	P	P	P	P	P	1,399	P	A	P	A	P	P	P	P
1031722	COG0441J	gbs0684	thrS	Cellular processess	Translation, ribosomal structure and biogenesis	Threonyl-tRNA synthetase (EC 6.1.1.3)	1,103	P	P	P	P	P	P	P	P	0,631	P	P	P	A	P	P	P	M
1031725	COG0745TK	gbs0685		Cellular processess	Transcription	Transcriptional regulatory protein	4,461	P	A	A	A	A	A	A	A	5,149	P	A	A	A	A	A	A	A
1031730	-	gbs0686		Hypothetical		Hypothetical cytosolic protein	1,518	P	P	P	P	P	P	P	P	0,853	P	A	P	P	A	P	P	P
1031723	-	gbs0687		Hypothetical		Hypothetical membrane associated protein	0,154	P	P	P	P	P	P	P	P	0,142	P	P	P	P	P	P	P	P
1031728	COG0765E	gbs0688		Metabolism and transport	Amino acids, peptides, aminosugars and amines	Glutamine transport system permease protein glnP	8,312	P	A	P	P	P	M	P	A	4,488	P	A	A	A	A	P	A	A
1031732	COG0765E	gbs0689		Metabolism and transport	Amino acids, peptides, aminosugars and amines	Glutamine transport system permease protein glnP	15,956	P	A	A	A	A	A	A	A	5,152	P	A	A	A	A	A	A	A
1031714	COG0834ET	gbs0690		Metabolism and transport	Amino acids, peptides, aminosugars and amines	Glutamine-binding protein	10,446	P	A	A	A	A	A	A	A	5,471	P	A	A	A	A	A	A	A
1031301	COG1126E	gbs0691		Metabolism and transport	Amino acids, peptides, aminosugars and amines	Glutamine transport ATP-binding protein glnQ	10,191	P	A	A	A	A	A	A	A	1,968	A	A	A	A	A	A	A	A
1031731	-	gbs0692		Hypothetical		Hypothetical protein	0,271	A	A	A	A	A	A	A	A	0,307	A	A	A	A	A	A	A	A
1031733	-	gbs0693		Hypothetical		Hypothetical protein	0,713	P	A	A	A	A	A	M	A	0,361	P	A	A	A	A	A	A	A
1031727	-	gbs0694		Hypothetical		Hypothetical protein	0,213	A	A	P	P	P	A	A	A	0,228	P	A	P	P	A	P	A	A
1031740	-	gbs0695		Hypothetical		Hypothetical protein	0,305	A	A	A	A	A	A	A	A	0,323	A	A	A	A	A	A	A	A
1031737	-	gbs0696		Hypothetical		Hypothetical protein	1,416	A	A	A	A	A	A	A	A	0,450	A	A	A	A	A	A	A	A
1031741	-	gbs0697		Hypothetical		Hypothetical protein	0,928	M	A	A	A	A	A	A	A	0,354	A	A	A	A	A	A	A	A
1031735	-	gbs0698		General function predicted only		RepR protein	0,646	A	A	P	A	A	A	A	A	0,084	A	M	A	A	A	A	A	A
1031745	-	gbs0699		Hypothetical		Hypothetical protein	0,100	A	A	A	A	A	A	A	A	0,297	A	A	A	A	A	A	A	A
1031734	-	gbs0700		Hypothetical		Hypothetical protein	0,316	M	P	P	P	P	A	A	M	0,324	M	A	M	A	A	A	A	A
1031749	-	gbs0701		Hypothetical		Hypothetical protein	1,014	A	A	A	A	A	A	A	A	0,288	A	A	A	A	A	A	A	A
1031738	-	gbs0702		Hypothetical		Hypothetical protein	0,941	A	A	A	A	A	A	A	A	0,356	A	A	A	A	A	A	A	A
1031743	-	gbs0703		Hypothetical		Hypothetical protein	0,368	A	A	A	A	A	A	A	A	0,226	A	A	A	A	A	A	A	A
1031753	-	gbs0708		Hypothetical		Hypothetical protein	0,436	P	A	P	A	P	M	A	A	0,163	P	A	A	A	A	A	A	A
1031750	COG0542O	gbs0718	clpA ?	Hypothetical		Hypothetical protein	0,394	M	A	A	A	A	A	A	A	0,476	A	A	A	A	A	A	A	A
1031769	-	gbs0727		Hypothetical		Hypothetical protein	0,300	P	P	P	P	P	P	P	P	0,737	P	P	P	P	P	P	P	P
1030882	-	gbs0728		Hypothetical		Hypothetical protein	0,956	P	P	P	P	P	A	P	A	0,963	P	A	A	A	A	A	A	A
1031772	COG0272L	gbs0729		Hypothetical		Hypothetical protein	1,169	P	P	P	P	P	A	P	A	1,158	P	A	A	A	A	A	A	A
1031768	-	gbs0730		Hypothetical		Hypothetical protein	0,264	P	M	P	P	P	P	P	A	0,548	A	A	A	A	A	A	A	A
1031771	-	gbs0731		Hypothetical		Hypothetical protein	0,336	P	P	P	P	P	P	P	P	0,530	P	A	P	A	A	M	A	P
1030878	COG0745TK	gbs0741	vicR	Cellular processess	Signal transduction	Two-component response regulator VicR	1,369	P	P	P	P	P	P	P	P	1,011	P	P	P	P	P	P	P	P
1031774	COG5002T	gbs0742	vicK	Cellular processess	Signal transduction	Two-component sensor histidine kinase VicK (EC 2.7.3.-)	1,643	P	P	P	P	P	P	P	P	0,840	P	P	P	P	P	P	P	P
1031197	COG1235R	gbs0743	vicX	General function predicted only		Zn-dependent hydrolase (beta-lactamase superfamily)	4,337	P	P	P	P	P	P	P	P	1,050	P	P	P	P	P	P	P	P
1030918	COG2832S	gbs0744		Hypothetical		Hypothetical membrane spanning protein	3,547	P	M	P	M	P	P	P	P	0,946	P	P	P	P	P	P	P	P
1031165	COG0571K	gbs0745	rncS	Cellular processess	Transcription	Ribonuclease III (EC 3.1.26.3)	3,025	P	P	P	A	P	P	P	M	1,030	P	A	P	P	P	P	P	A
1031776	COG1196D	gbs0746	smc	Cellular processess	Cell division	Chromosome partition protein smc	1,862	P	A	A	A	A	A	A	A	1,101	P	A	A	A	A	A	A	A
1030834	COG0561R	gbs0747		Metabolism and transport	Central intermediary metabolism	Hydrolase (HAD superfamily)	1,973	P	A	M	A	A	A	A	A	1,867	P	A	P	M	A	A	A	A
1031298	COG0561R	gbs0748		Metabolism and transport	Central intermediary metabolism	Hydrolase (HAD superfamily)	1,562	P	P	P	A	P	P	P	P	1,873	P	A	P	P	P	P	P	P
1031780	COG0552U	gbs0749	ftsY	Cellular processess	Cell division	Cell division protein ftsY	1,332	P	P	P	P	P	P	P	P	1,443	P	P	P	P	P	P	A	P
1031777	COG3689S	gbs0750		Hypothetical		Hypothetical membrane spanning protein	1,928	P	P	P	P	P	P	P	P	1,313	P	A	P	A	M	P	A	M
1031216	COG0701R	gbs0751		Hypothetical		Hypothetical membrane spanning protein	2,456	P	P	P	P	P	P	P	P	0,976	P	A	P	P	P	P	P	P
1031779	-	gbs0752		Hypothetical		Hypothetical protein	2,441	P	P	P	P	P	P	P	P	0,749	P	A	P	M	P	P	A	A

1031151	COG2141C	gbs0753		Metabolism and transport	Energy production and conversion	Luciferase-like monooxygenase (EC 1.14.-.-)	0,448	P	A	A	A	A	A	A	A	0,483	A	A	A	A	A	A	A	A
1031205	COG2183K	gbs0754		Cellular processess	Transcription	TRANSCRIPTION ACCESSORY PROTEIN (S1 RNA binding domain)	3,709	P	A	A	A	A	A	A	A	1,549	P	A	A	A	A	A	A	A
1031050	-	gbs0755		Cellular processess	Toxin production and resistance	Metallopeptidase, SprT family (EC 3.4.24.-)	1,749	P	P	P	P	P	P	P	P	1,126	P	A	P	A	P	P	P	P
1031462	COG1983KT	gbs0756		Cellular processess	Transcription	Stress-responsive transcriptional regulator PspC	0,932	P	P	P	P	P	P	P	P	0,396	P	P	P	P	P	P	P	P
1031783	COG1493T	gbs0757	ptsK	Cellular processess	Signal transduction	HPR(SER) KINASE (EC 2.7.1.-) PHOSPHATASE (EC 3.1.3.-)	0,550	P	P	P	P	P	P	P	P	0,720	P	P	P	P	P	P	P	P
1031041	-	gbs0758	lgt	Cellular processess	Posttranslational modification, protein turnover, chaperones	Prolipoprotein diacylglyceryl transferase (EC 2.4.99.-)	0,741	P	P	P	P	P	P	P	P	0,801	P	P	P	P	P	P	P	P
1031264	COG4768R	gbs0759		Hypothetical		Hypothetical protein	0,559	P	P	P	P	P	P	P	P	0,626	P	P	P	P	P	P	P	P
1031781	-	gbs0760		Hypothetical		Hypothetical exported protein	0,574	P	P	P	P	P	P	P	P	0,665	P	P	P	P	P	P	P	P
1031786	-	gbs0761		Hypothetical		Hypothetical protein	2,905	P	P	P	P	P	P	P	P	0,769	P	P	P	P	P	P	P	P
1031183	-	gbs0762		Cellular processess	Posttranslational modification, protein turnover, chaperones	Peptidase family U32	5,227	P	A	P	P	P	P	A	P	0,946	P	A	P	P	P	P	P	P
1031342	-	gbs0763		Cellular processess	Posttranslational modification, protein turnover, chaperones	Peptidase family U32	6,688	P	A	A	A	A	A	A	A	1,041	A	A	A	A	A	A	A	A
1031363	COG4443S	gbs0764		Hypothetical		Hypothetical cytosolic protein	0,619	P	A	P	A	P	P	P	P	0,793	A	A	A	A	A	A	A	A
1031785	-	gbs0765		Hypothetical		Hypothetical protein	0,694	P	P	P	P	P	P	P	P	0,503	P	P	P	P	P	P	P	P
1031379	COG1914P	gbs0766		Metabolism and transport	Inorganic ion transport and metabolism	Manganese transport protein mntH	2,452	P	P	P	P	P	P	P	P	1,237	P	A	P	A	P	P	A	A
1031104	0117H, COG1	gbs0767		Metabolism and transport	Cofactors, prosthetic groups, and carriers	Diaminohydroxyphosphoribosylaminopyrimidine deaminase (EC 3.5.4.26) 5-amin	2,048	P	P	P	P	P	P	P	P	2,390	P	A	P	A	A	P	A	P
1031789	COG0307H	gbs0768		Metabolism and transport	Cofactors, prosthetic groups, and carriers	Riboflavin synthase alpha chain (EC 2.5.1.9)	1,119	P	P	P	P	P	P	P	P	2,414	P	A	P	P	P	P	A	P
1031790	COG0807H	gbs0769		Metabolism and transport	Cofactors, prosthetic groups, and carriers	GTP cyclohydrolase II (EC 3.5.4.25) 3,4-dihydroxy-2-butanone-4-phosphate syntl	1,037	P	P	P	P	P	P	P	P	2,786	P	A	P	P	P	P	A	P
1031373	-	gbs0770		Metabolism and transport	Cofactors, prosthetic groups, and carriers	6,7-dimethyl-8-ribityllumazine synthase (EC 2.5.1.9)	0,841	P	P	P	P	P	P	P	P	3,188	P	P	P	P	P	P	P	P
1031784	COG1190J	gbs0771	lysS	Cellular processess	Translation, ribosomal structure and biogenesis	Lysyl-tRNA synthetase (EC 6.1.1.6)	2,529	P	P	P	P	P	P	P	P	1,137	P	P	P	P	P	P	P	P
1031788	COG1011R	gbs0772		Metabolism and transport	Central intermediary metabolism	Haloacid dehalogenase-like hydrolase	2,286	P	A	A	A	A	A	A	A	1,343	P	A	A	A	A	A	A	A
1031445	COG0406G	gbs0773		Metabolism and transport	Carbohydrates, organic alcohols, and acids	Phosphoglycerate mutase family protein	0,350	P	P	P	P	P	P	P	P	0,683	P	P	P	P	P	P	P	P
1031793	COG2606S	gbs0774		Cellular processess	Transcription	Transcriptional regulator	0,526	P	P	P	P	P	P	P	P	1,044	P	P	P	P	P	P	P	P
1031787	451MG, COG0	gbs0775		General function predicted only		NADH-ubiquinone oxidoreductase 39-40 kDa subunit homolog	1,111	P	A	P	A	A	A	A	A	1,966	P	A	A	A	A	A	A	A
1031042	COG3757M	gbs0776		Cellular processess	Posttranslational modification, protein turnover, chaperones	N-acetylmuramoyl-L-alanine amidase (EC 3.5.1.28)	2,058	P	P	P	P	P	P	P	P	1,262	P	P	P	P	P	P	P	P
1031791	-	gbs0777		Hypothetical		Integral membrane protein	1,029	P	P	P	P	P	P	P	P	0,760	P	A	A	A	A	A	A	A
1030905	-	gbs0778		Hypothetical		Hypothetical exported protein	0,653	P	P	P	P	P	P	P	P	0,558	P	P	P	P	P	P	P	P
1031466	COG1164E	gbs0779	pepF	Metabolism and transport	Amino acids, peptides, aminosugars and amines	Oligoendopeptidase F (EC 3.4.24.-)	1,105	P	P	P	P	P	P	P	P	0,725	P	P	P	P	P	P	P	P
1031917	COG2352C	gbs0780	ppc	Metabolism and transport	Energy production and conversion	Phosphoenolpyruvate carboxylase (EC 4.1.1.31)	1,865	P	P	P	P	P	P	P	P	1,272	P	A	P	A	A	P	P	A
1031792	-	gbs0781	ftsW	Cellular processess	Cell division	Cell division protein ftsW	3,193	P	A	P	A	A	M	A	A	0,624	P	A	A	A	A	A	A	A
1031913	COG0050J	gbs0782	tuf	Cellular processess	Translation, ribosomal structure and biogenesis	Protein Translation Elongation Factor Tu (EF-TU)	0,467	P	P	P	P	P	P	P	P	0,529	P	P	P	P	P	P	P	P
1031916	COG0149G	gbs0783	tpiA	Metabolism and transport	Carbohydrates, organic alcohols, and acids	Triosephosphate isomerase (EC 5.3.1.1)	0,763	P	P	P	P	P	P	P	P	0,717	P	P	P	P	P	P	P	P
1031918	COG0588G	gbs0784	gpmA	Metabolism and transport	Carbohydrates, organic alcohols, and acids	Phosphoglycerate mutase (EC 5.4.2.1)	0,907	P	P	P	P	P	P	P	P	0,945	P	P	P	P	P	P	P	P
1031914	COG0768M	gbs0785		Cell Envelope	Biosynthesis and degradation of murein sacculus and peptidoglycan	Penicillin-binding protein	3,027	P	P	P	P	P	P	P	P	1,077	P	A	P	A	A	P	P	A
1031920	COG0353L	gbs0786	recR	Cellular processess	DNA replication, recombination and repair	Recombination protein recR	2,711	P	P	P	P	P	P	P	P	0,752	P	P	P	A	P	P	P	A
1031911	COG1181M	gbs0787	ddl	Cell Envelope	Biosynthesis and degradation of murein sacculus and peptidoglycan	D-alanine--D-alanine ligase (EC 6.3.2.4)	3,030	P	A	A	A	M	A	A	A	1,058	P	A	A	A	A	A	A	A
1031919	COG0770M	gbs0788	murF	Cell Envelope	Biosynthesis and degradation of murein sacculus and peptidoglycan	UDP-N-acetylmuramoyl-tripeptide--D-alanyl-D-alanine ligase (EC 6.3.2.10)	1,915	P	P	P	M	P	P	P	P	1,076	P	P	P	P	P	P	P	P
1031915	-	gbs0789		Transport and binding proteins	Unknown substrate	Transporter, MFS superfamily	0,723	P	P	P	P	P	P	P	P	0,608	P	A	A	A	A	P	A	A
1031924	COG5522S	gbs0790		Hypothetical		Hypothetical membrane spanning protein	3,466	P	P	P	A	P	P	P	P	1,917	P	A	M	A	P	P	A	A
1031910	-	gbs0791		Hypothetical		LPXTG Surface anchor protein	1,396	P	M	P	P	P	P	P	P	0,872	P	A	P	M	P	P	A	P
1031905	COG4108J	gbs0792	prfC	Cellular processess	Translation, ribosomal structure and biogenesis	Bacterial Peptide Chain Release Factor 3 (RF-3)	1,718	P	P	P	M	P	P	P	P	0,685	P	A	P	P	P	P	M	P
1031912	-	gbs0793		Hypothetical		Hypothetical cytosolic protein	1,491	P	P	P	P	P	P	P	P	1,963	P	A	P	A	P	P	A	A
1031922	COG1135P	gbs0794		Metabolism and transport	Inorganic ion transport and metabolism	Probable D-methionine transport ATP-binding protein metN	1,758	P	P	P	A	P	P	P	P	0,932	P	A	A	A	A	P	P	M
1031921	COG2011P	gbs0795		Transport and binding proteins	Unknown substrate	ABC transporter permease protein	3,548	P	P	P	A	P	P	A	P	1,189	P	A	P	A	A	P	A	A
1031923	COG1464P	gbs0796		Transport and binding proteins	Unknown substrate	ABC transporter substrate-binding protein	1,561	P	P	P	A	P	P	P	P	1,138	P	P	P	A	P	P	P	P
1031907	COG0513LKJ	gbs0797	deaD	Cellular processess	DNA replication, recombination and repair	ATP-dependent RNA helicase	2,957	P	P	A	A	P	P	P	M	0,773	A	A	A	A	A	A	P	A
1031909	COG2827L	gbs0798		Hypothetical		Hypothetical protein with endo excinuclease domain	1,337	P	A	P	M	P	P	A	P	1,313	P	A	P	A	A	M	A	A
1031906	COG4123R	gbs0799		Metabolism and transport	Central intermediary metabolism	Methyltransferase (EC 2.1.1.-)	1,850	P	A	A	A	A	A	A	A	1,518	P	A	A	A	A	A	A	A
1031926	COG0204I	gbs0800		Metabolism and transport	Fatty acid and phospholipids	1-acyl-sn-glycerol-3-phosphate acyltransferase (EC 2.3.1.51)	1,292	P	P	P	P	P	P	P	P	0,662	P	P	P	P	P	P	P	P
1031925	COG1555L	gbs0801		Cellular processess	DNA transformation	COME operon protein 1	0,194	A	A	A	A	A	A	A	A	0,083	A	A	A	A	A	A	A	A
1031903	0658R, COG2	gbs0802	comEC	General function predicted only		COME operon protein 3	1,091	P	A	A	A	A	A	A	A	0,240	A	A	A	A	A	A	A	A
1031902	COG0561R	gbs0803		Metabolism and transport	Central intermediary metabolism	Hydrolase (HAD superfamily)	1,185	P	A	P	P	A	P	A	M	0,924	P	A	A	A	A	A	A	A
1031904	COG1609K	gbs0804		Cellular processess	Transcription	Catabolite control protein B	1,374	P	P	P	P	P	P	P	P	0,641	P	A	P	P	M	P	A	P
1031908	COG0657I	gbs0805		Metabolism and transport	Fatty acid and phospholipids	Esterase (EC 3.1.1.-)	4,525	A	A	A	A	A	A	A	A	0,639	A	A	A	A	A	A	A	A
1031888	COG0500QR	gbs0806		Metabolism and transport	Secondary metabolites	Biotin synthesis protein bioC	0,438	P	P	P	P	P	P	P	P	0,606	P	P	P	P	A	P	A	A
1031901	COG1466L	gbs0807		Cellular processess	DNA replication, recombination and repair	DNA polymerase III, delta subunit (EC 2.7.7.7)	1,566	P	P	P	P	P	P	P	P	0,887	P	M	P	P	P	P	P	P
1031927	COG0605P	gbs0808	sodA	Metabolism and transport	Inorganic ion transport and metabolism	Superoxide dismutase (EC 1.15.1.1)	0,387	P	P	P	P	P	P	P	P	0,403	P	P	P	P	P	P	P	P
1031900	COG3711K	gbs0809	licT	Cellular processess	Transcription	Transcription antiterminator, BglG family	0,506	P	A	P	A	A	A	A	A	1,792	P	P	P	A	A	A	A	P
1031929	COG1263G	gbs0810		Metabolism and transport	Carbohydrates, organic alcohols, and acids	PTS system, beta-glucoside-specific IIABC component (EC 2.7.1.69)	0,307	A	A	A	A	A	A	A	A	1,047	P	A	A	A	A	A	A	P
1031897	-	gbs0811	bglA	Metabolism and transport	Carbohydrates, organic alcohols, and acids	6-phospho-beta-glucosidase (EC 3.2.1.86)	1,520	P	A	A	A	A	A	A	A	1,900	P	A	P	A	A	A	A	P
1031899	COG3835KT	gbs0812		Cellular processess	Transcription	Transcriptional regulatory protein	1,109	P	A	M	A	A	A	M	P	1,796	P	A	A	A	A	A	A	A
1031896	-	gbs0813		Metabolism and transport	Energy production and conversion	Glycerate kinase (EC 2.7.1.31)	0,728	A	A	A	A	A	A	A	A	0,262	A	A	A	A	A	A	A	A
1031898	COG2610GE	gbs0814		Metabolism and transport	Carbohydrates, organic alcohols, and acids	Gluconate permease	0,498	P	A	A	A	A	A	A	A	0,514	P	A	P	A	P	P	M	A
1031893	1686M, COG2	gbs0815		Cellular processess	Posttranslational modification, protein turnover, chaperones	Peptidase family S11	3,068	P	A	A	A	A	A	A	A	1,253	P	A	A	A	A	A	A	A
1031928	COG1846K	gbs0816		Cellular processess	Transcription	Transcriptional regulator, MarR family	1,311	P	P	P	P	P	P	P	P	1,685	P	A	P	A	P	P	A	P
1031930	-	gbs0817	queA	Cellular processess	Translation, ribosomal structure and biogenesis	S-adenosylmethionine:tRNA ribosyltransferase-isomerase (EC 5.-.-.-)	1,134	P	P	P	P	P	P	P	P	0,559	P	A	P	P	M	P	P	M
1031894	COG1814S	gbs0818		Hypothetical		Hypothetical membrane associated protein	0,463	P	A	P	M	P	P	A	A	0,189	A	A	A	A	A	A	A	A
1031895	COG0363G	gbs0819	nagB	Metabolism and transport	Carbohydrates, organic alcohols, and acids	Glucosamine-6-phosphate isomerase (EC 3.5.99.6)	0,760	P	P	P	P	P	P	P	P	0,487	P	P	P	M	P	P	P	P
1031890	COG0435O	gbs0820		Metabolism and transport	Central intermediary metabolism	Glutathione S-transferase family protein	0,800	P	P	P	P	P	P	A	P	0,711	P	A	P	P	A	P	P	A
1031932	COG1187J	gbs0821		Cellular processess	Translation, ribosomal structure and biogenesis	Ribosomal small subunit pseudouridine synthase A (EC 4.2.1.70)	3,375	P	A	A	A	A	A	A	A	3,545	P	A	A	A	A	A	A	A
1031934	-	gbs0822		Transport and binding proteins	Unknown substrate	Transporter, MFS superfamily	1,635	P	A	A	A	M	A	A	A	0,667	P	A	M	A	A	P	P	A
1031931	COG4469R	gbs0823		Cellular processess	DNA transformation	Putative competence protein transcription factor	0,965	P	A	A	A	A	A	A	A	0,704	A	A	A	A	A	A	A	A
1029783	COG1164E	gbs0824	pepB	Metabolism and transport	Amino acids, peptides, aminosugars and amines	Oligoendopeptidase F (EC 3.4.24.-)	1,132	P	P	P	P	P	P	P	P	1,249	P	P	P	A	P	P	P	P
1029784	COG0546R	gbs0825		Metabolism and transport	Central intermediary metabolism	Phosphoglycolate phosphatase (EC 3.1.3.18)	2,577	P	A	P	A	P	P	A	A	0,765	P	A	A	M	A	A	A	A
1031933	COG4122R	gbs0826		Cellular processess	Posttranslational modification, protein turnover, chaperones	O-methyltransferase (EC 2.1.1.-)	2,128	P	A	P	A	A	A	M	A	1,077	P	A	A	A	A	A	A	A
1031892	COG0760O	gbs0827	prsA	Cellular processess	Posttranslational modification, protein turnover, chaperones	Peptidyl-prolyl cis-trans isomerase (EC 5.2.1.8)	1,739	P	P	P	P	P	P	P	P	0,926	P	P	P	P	P	P	P	P
1029786	COG4894S	gbs0828		Hypothetical		Hypothetical protein	1,692	P	P	P	P	P	P	P	P	0,971	P	M	P	A	A	P	P	P
1029789	COG0013J	gbs0829	alaS	Cellular processess	Translation, ribosomal structure and biogenesis	Alanyl-tRNA synthetase (EC 6.1.1.7)	1,322	P	P	P	A	P	P	M	A	1,043	P	A	A	A	A	A	A	A
1029810	COG1811R	gbs0830		Hypothetical		Hypothetical membrane spanning protein	2,042	P	P	P	P	P	P	A	P	1,518	P	A	A	A	A	P	A	A
1031935	COG1442M	gbs0831		Metabolism and transport	Central intermediary metabolism	Lipopolysaccharide 1,2-glucosyltransferase (EC 2.4.1.58)	1,583	P	P	P	P	P	P	P	P	0,700	P	A	A	A	A	P	P	M
1029812	-	gbs0832		Hypothetical		Hypothetical protein	0,448	P	P	P	P	P	P	P	P	0,484	P	M	P	P	P	P	P	P
1029805	COG1476K	gbs0833		Cellular processess	Transcription	Transcriptional regulator, Cro CI family	0,435	P	P	P	P	P	P	P	P	0,484	P	P	P	P	P	P	P	P
1029809	-	gbs0834		Hypothetical		Hypothetical membrane spanning protein	0,664	P	P	P	P	P	P	P	P	0,841	P	A	P	A	P	P	A	P
1029785	COG3859S	gbs0835		Transport and binding proteins	Unknown substrate	Thiamine transporter	1,105	P	A	A	A	P	A	A	P	1,147	P	A	A	A	A	P	A	A
1029811	COG0208F	gbs0836	nrdF.2	Metabolism and transport	Purines, pyrimidines, nucleosides, and nucleotides	Ribonucleoside-diphosphate reductase beta chain (EC 1.17.4.1)	2,614	P	P	P	P	P	P	P	P	0,775	P	A	P	P	P	P	P	P
1029807	COG0209F	gbs0837	nrdE.2	Metabolism and transport	Purines, pyrimidines, nucleosides, and nucleotides	Ribonucleoside-diphosphate reductase alpha chain (EC 1.17.4.1)	1,607	P	A	P	A	P	P	A	M	0,379	P	A	P	A	M	P	M	A
1029814	COG0695O	gbs0838	nrdH	Cellular processess	Posttranslational modification, protein turnover, chaperones	Glutaredoxin	1,718	P	P	P	M	P	P	P	P	0,515	P	A	P	A	A	A	A	A
1029813	COG1925G	gbs0839	ptsH	Metabolism and transport	Carbohydrates, organic alcohols, and acids	Phosphocarrier protein HPr	0,441	P	P	P	P	P	P	P	P	0,648	P	P	P	P	P	P	P	P
1029818	COG1080G	gbs0840	ptsI	Metabolism and transport	Carbohydrates, organic alcohols, and acids	Phosphoenolpyruvate-protein phosphotransferase (EC 2.7.3.9)	0,846	P	P	P	P	P	P	P	P	1,081	P	P	P	P	P	P	P	P
1029815	COG1012C	gbs0841	gapN	Metabolism and transport	Energy production and conversion	NADP-dependent glyceraldehyde-3-phosphate dehydrogenase (EC 1.2.1.9)	0,959	P	P	P	P	P	P	P	P	0,929	P	P	P	P	P	P	P	P
1029817	-	gbs0842		Cell Envelope	Biosynthesis and degradation of surface polysaccharides and lipopo	Peptidoglycan N-acetylglucosamine deacetylase (EC 3.5.1.-)	1,323	P	P	P	P	P	P	P	P	2,034	P	A	P	P	P	P	P	P
1029819	COG0513LKJ	gbs0843	deaD2	Cellular processess	DNA replication, recombination and repair	ATP-dependent RNA helicase	0,953	P	P	P	P	P	P	P	P	0,936	P	A	P	P	P	P	P	P
1029822	COG0572F	gbs0844	udk	Metabolism and transport	Purines, pyrimidines, nucleosides, and nucleotides	Uridine kinase (EC 2.7.1.48)	3,023	P	P	P	P	P	P	P	P	1,274	P	A	P	A	A	P	A	A
1029825	COG1956T	gbs0845		Cellular processess	Signal transduction	GAF domain-containing proteins	2,750	P	P	P	P	P	P	P	P	1,142	P	P	P	A	P	P	P	P
1029829	COG2812L	gbs0846		Cellular processess	DNA replication, recombination and repair	DNA polymerase III subunit gamma tau (EC 2.7.7.7)	2,035	P	P	P	P	P	P	P	P	1,452	P	A	P	P	P	P	P	P
1029820	-	gbs0847		Hypothetical		Hypothetical protein	2,264	P	A	A	A	A	A	A	A	0,808	P	A	A	A	A	A	A	A

1029826	0340H, COG1	gbs0848	birA	Cellular processess	Transcription	Biotin operon repressor Biotin--[acetyl-CoA-carboxylase] synthetase (EC 6.3.4.15	1,398	P	A	A	A	A	A	A	A	0,801	P	A	A	A	A	A	A	A
1029821	COG0192H	gbs0849	metK2	Metabolism and transport	Cofactors, prosthetic groups, and carriers	S-adenosylmethionine synthetase (EC 2.5.1.6)	2,007	P	P	M	A	P	P	P	M	1,485	P	A	P	A	P	P	M	A
1029828	-	gbs0850	fbsB	Hypothetical		Hypothetical protein	1,196	P	A	A	A	A	A	A	A	1,316	A	A	A	A	A	A	A	A
1029823	-	gbs0851		Hypothetical		Hypothetical protein	1,152	P	A	A	A	A	A	A	A	0,828	A	A	A	A	A	A	A	A
1029834	-	gbs0852		Hypothetical		Hypothetical protein	1,144	P	A	A	A	A	A	A	A	0,350	A	A	A	A	A	A	A	A
1029824	COG4732S	gbs0853		Transport and binding proteins	Unknown substrate	Hydroxyethylthiazole permease	0,186	A	A	A	A	A	A	A	A	0,273	A	A	A	A	A	A	A	A
1029833	COG4721S	gbs0854		Transport and binding proteins	Unknown substrate	Hydroxymethylpyrimidine transport system permease protein	1,630	A	A	A	A	A	A	A	A	0,570	A	A	A	A	A	A	A	A
1029836	COG1122P	gbs0855		Metabolism and transport	Inorganic ion transport and metabolism	Transporter	2,649	A	A	A	A	A	A	A	A	0,848	A	A	A	A	A	A	A	A
1029832	COG0619P	gbs0856		Metabolism and transport	Inorganic ion transport and metabolism	Hydroxymethylpyrimidine transport system permease protein	1,472	A	A	A	A	A	A	A	A	0,404	A	A	A	A	A	A	A	A
1029838	COG0819K	gbs0857		Cellular processess	Transcription	Transcriptional activator tenA	0,511	A	A	A	A	A	A	A	A	0,477	P	A	A	A	A	A	A	A
1029827	COG0351H	gbs0858		Metabolism and transport	Cofactors, prosthetic groups, and carriers	Phosphomethylpyrimidine kinase (EC 2.7.4.7) Hydroxymethylpyrimidine kinase (E	0,934	A	A	A	A	A	A	A	A	0,745	A	A	A	A	A	A	A	A
1029837	COG2145H	gbs0859		Metabolism and transport	Cofactors, prosthetic groups, and carriers	Hydroxyethylthiazole kinase (EC 2.7.1.50)	0,549	P	A	A	A	M	A	A	A	0,381	P	A	A	A	M	A	A	P
1029839	COG0352H	gbs0860		Metabolism and transport	Cofactors, prosthetic groups, and carriers	Thiamin-phosphate pyrophosphorylase (EC 2.5.1.3)	0,958	M	A	A	A	A	A	A	A	0,745	P	A	A	A	A	A	A	A
1029841	COG0766M	gbs0861	murZ	Cell Envelope	Biosynthesis and degradation of murein sacculus and peptidoglycan	UDP-N-acetylglucosamine 1-carboxyvinyltransferase (EC 2.5.1.7)	3,465	P	A	A	A	A	P	A	A	1,190	P	A	A	A	A	A	A	A
1029840	COG1670J	gbs0862		Cellular processess	Translation, ribosomal structure and biogenesis	Ribosomal-protein-S5-alanine acetyltransferase (EC 2.3.1.128)	2,247	P	A	P	P	P	P	P	M	1,446	P	A	P	A	P	P	P	P
1029848	COG4109K	gbs0863		Cellular processess	Transcription	Cytosolic protein containing multiple CBS domains	1,995	P	A	M	A	P	A	A	A	1,182	P	A	M	A	A	P	M	A
1029845	COG0024J	gbs0864	map	Cellular processess	Translation, ribosomal structure and biogenesis	Methionine aminopeptidase (EC 3.4.11.18)	1,971	P	P	P	P	P	P	P	P	1,307	P	P	P	P	P	P	P	P
1029846	COG1295S	gbs0865		Cellular processess	Transcription	Ribonuclease BN (EC 3.1.-.-)	2,949	P	P	P	P	P	P	P	P	1,199	P	M	P	P	P	P	P	P
1029847	COG2246S	gbs0866		General function predicted only		Bactoprenol-linked monosaccharide translocase (flippase type)	1,014	P	A	A	A	A	A	M	A	0,594	P	A	A	A	A	P	A	A
1029842	COG4708S	gbs0867		Hypothetical		Hypothetical membrane spanning protein	1,015	P	P	P	P	P	P	P	P	0,508	P	A	P	A	P	P	A	A
1029844	COG0272L	gbs0868	lig	Cellular processess	DNA replication, recombination and repair	NAD-dependent DNA ligase (EC 6.5.1.2)	1,078	P	A	A	A	A	A	A	A	1,358	P	A	A	A	A	A	A	A
1029843	COG1597IR	gbs0869		Metabolism and transport	Fatty acid and phospholipids	Diacylglycerol kinase family protein	1,305	P	A	P	P	P	P	P	P	1,433	P	P	P	P	A	P	P	A
1029853	COG1523G	gbs0870		Metabolism and transport	Carbohydrates, organic alcohols, and acids	Pullulanase (EC 3.2.1.41)	1,337	P	A	P	M	M	A	A	A	1,650	P	A	P	A	M	M	A	A
1029852	COG0296G	gbs0871		Metabolism and transport	Carbohydrates, organic alcohols, and acids	1,4-alpha-glucan branching enzyme (EC 2.4.1.18)	1,056	P	P	P	P	P	P	P	P	1,269	P	P	P	P	P	P	P	P
1029849	COG0448G	gbs0872	glgC	Metabolism and transport	Carbohydrates, organic alcohols, and acids	Glucose-1-phosphate adenylyltransferase catalytic subunit (EC 2.7.7.27)	0,800	P	P	P	P	P	P	P	P	1,219	P	P	P	P	P	P	P	P
1029851	COG0448G	gbs0873		Metabolism and transport	Carbohydrates, organic alcohols, and acids	Glucose-1-phosphate adenylyltransferase regulatory subunit (EC 2.7.7.27)	0,805	P	P	P	P	P	P	P	P	1,154	P	P	P	P	P	P	P	P
1029850	COG0297G	gbs0874		Metabolism and transport	Carbohydrates, organic alcohols, and acids	Glycogen synthase (EC 2.4.1.21)	1,294	P	P	P	P	P	P	P	P	1,159	P	P	P	P	P	P	P	P
1029854	-	gbs0875	atpE	Metabolism and transport	Energy production and conversion	ATP synthase C chain (EC 3.6.3.14)	1,114	P	P	P	P	P	P	P	P	0,884	P	M	P	P	P	P	P	P
1029856	-	gbs0876	atpB	Metabolism and transport	Energy production and conversion	ATP synthase A chain (EC 3.6.3.14)	1,806	P	P	P	P	P	P	P	P	1,295	P	P	P	P	P	P	P	P
1029857	COG0711C	gbs0877	atpF	Metabolism and transport	Energy production and conversion	ATP synthase B chain (EC 3.6.3.14)	1,859	P	P	P	P	P	P	P	P	1,220	P	P	P	P	P	P	P	P
1029859	COG0712C	gbs0878	atpH	Metabolism and transport	Energy production and conversion	ATP synthase delta chain (EC 3.6.3.14)	2,636	P	P	P	P	P	P	P	P	1,406	P	P	P	P	P	P	P	P
1029855	COG0056C	gbs0879	atpA	Metabolism and transport	Energy production and conversion	ATP synthase alpha chain (EC 3.6.3.14)	1,646	P	P	P	P	P	P	P	P	1,389	P	P	P	P	P	P	P	P
1029865	COG0224C	gbs0880	atpG	Metabolism and transport	Energy production and conversion	ATP synthase gamma chain (EC 3.6.3.14)	1,841	P	P	P	P	P	P	P	P	1,358	P	P	P	P	P	P	P	P
1029858	COG0055C	gbs0881	atpD	Metabolism and transport	Energy production and conversion	ATP synthase beta chain (EC 3.6.3.14)	1,499	P	P	P	P	P	P	P	P	1,273	P	P	P	P	P	P	P	P
1029863	-	gbs0882	atpC	Metabolism and transport	Energy production and conversion	ATP synthase epsilon chain (EC 3.6.3.14)	1,093	P	P	P	P	P	P	P	P	0,962	P	P	P	P	P	P	P	P
1029866	COG0766M	gbs0883	murA	Cell Envelope	Biosynthesis and degradation of murein sacculus and peptidoglycan	UDP-N-acetylglucosamine 1-carboxyvinyltransferase (EC 2.5.1.7)	1,551	P	P	P	A	P	P	P	P	0,965	P	A	P	M	P	P	P	M
1029867	-	gbs0884	epuA	General function predicted only		EpuA protein	0,868	P	P	P	A	P	P	P	P	0,591	P	A	P	A	P	P	A	A
1029861	-	gbs0885	endA	Cellular processess	DNA transformation	DNA-entry nuclease (EC 3.1.30.-)	3,358	P	A	A	A	M	A	A	A	1,672	P	A	A	A	A	A	A	A
1029860	COG0016J	gbs0886	pheS	Cellular processess	Translation, ribosomal structure and biogenesis	Phenylalanyl-tRNA synthetase alpha chain (EC 6.1.1.20)	1,710	P	P	P	P	P	P	P	P	1,272	P	A	P	A	P	P	P	A
1029864	-	gbs0887	pheT	Metabolism and transport	Central intermediary metabolism	Acetyltransferase, GNAT family	1,665	P	P	P	P	P	P	P	A	1,328	P	A	A	A	A	P	M	A
1029872	0072J, COG0	gbs0888	pheT	Cellular processess	Translation, ribosomal structure and biogenesis	Phenylalanyl-tRNA synthetase beta chain (EC 6.1.1.20)	1,421	P	P	P	P	P	P	P	P	0,882	P	P	P	P	P	P	P	P
1029871	COG3857L	gbs0890	rexB	Cellular processess	DNA replication, recombination and repair	ATP-dependent nuclease subunit B	1,218	P	A	A	A	P	A	A	A	1,794	P	A	P	A	A	A	A	A
1029875	COG1074L	gbs0891	rexA	Cellular processess	DNA replication, recombination and repair	ATP-dependent nuclease subunit A	1,059	M	A	A	A	A	A	A	A	0,846	M	A	A	A	A	A	A	A
1029873	COG0598P	gbs0892		Metabolism and transport	Inorganic ion transport and metabolism	Magnesium and cobalt transport protein corA	4,120	A	A	A	A	A	A	A	A	3,541	P	A	A	A	A	A	A	A
1029874	COG0486R	gbs0893	thdF	Cellular processess	Translation, ribosomal structure and biogenesis	tRNA (5-carboxymethylaminomethyl-2-thiouridylate) synthase	1,441	P	M	P	P	P	P	P	P	0,583	P	P	P	A	A	P	P	A
1029870	COG0488R	gbs0894		Transport and binding proteins	Unknown substrate	ABC transporter ATP-binding protein	3,672	P	A	A	A	A	A	A	A	2,327	P	A	P	A	A	A	A	A
1029869	COG1071C	gbs0895	acoA	Metabolism and transport	Energy production and conversion	Pyruvate dehydrogenase E1 component alpha subunit (EC 1.2.4.1)	0,713	P	P	P	P	P	P	P	P	0,695	P	P	P	P	P	P	P	P
1029878	COG0022C	gbs0896	acoB	Metabolism and transport	Energy production and conversion	Pyruvate dehydrogenase E1 component beta subunit (EC 1.2.4.1)	0,963	P	P	P	P	P	P	P	P	0,724	P	P	P	P	P	P	P	P
1029876	COG0508C	gbs0897	acoC	Metabolism and transport	Energy production and conversion	Dihydrolipoamide acetyltransferase component of pyruvate dehydrogenase comple	1,321	P	P	P	P	P	P	P	P	0,909	P	P	P	P	P	P	P	P
1029879	COG1249C	gbs0898	acoL	Metabolism and transport	Energy production and conversion	Dihydrolipoamide dehydrogenase (EC 1.8.1.4)	1,112	P	P	P	P	P	P	P	P	0,905	P	P	P	P	P	P	P	P
1029885	COG0095H	gbs0899	lplB	Metabolism and transport	Cofactors, prosthetic groups, and carriers	Lipoate-protein ligase A (EC 6.3.2.-)	1,702	P	P	P	A	P	P	P	P	1,290	P	P	P	P	P	P	P	P
1029877	COG3442R	gbs0900		General function predicted only		CobB CobQ-like glutamine amidotransferase domain	1,382	P	P	P	P	P	P	P	P	1,117	P	A	P	A	P	P	P	P
1029887	COG0769M	gbs0901		Cell Envelope	Biosynthesis and degradation of murein sacculus and peptidoglycan	UDP-N-acetylmuramoylalanyl-D-glutamate--2,6-diaminopimelate ligase (EC 6.3.2.1	2,137	P	M	A	A	P	M	P	A	0,719	P	A	A	A	A	A	A	A
1029881	-	gbs0902		Hypothetical		Hypothetical membrane spanning protein	0,666	P	P	P	P	P	P	P	P	1,068	P	P	P	P	P	P	P	P
1029884	COG4856S	gbs0903		Hypothetical		Hypothetical membrane associated protein	0,646	P	P	P	P	P	P	P	P	1,063	P	P	P	P	P	P	P	P
1029882	COG1109G	gbs0904	femD	Metabolism and transport	Carbohydrates, organic alcohols, and acids	Phosphoglucosamine mutase (EC 5.4.2.10)	1,067	P	P	P	P	P	P	P	P	0,703	P	A	P	M	P	P	P	P
1029890	COG3272S	gbs0905		Hypothetical		Hypothetical protein	0,869	P	A	P	P	A	P	A	A	1,083	P	A	P	A	A	A	A	A
1029886	-	gbs0906		Hypothetical		Hypothetical protein	0,863	P	P	P	P	P	P	P	A	0,910	P	A	P	A	M	P	M	A
1029883	COG0635H	gbs0907	hemN	Metabolism and transport	Cofactors, prosthetic groups, and carriers	Oxygen-independent coproporphyrinogen-III oxidase (EC 1.3.99.22)	1,684	P	A	P	P	P	P	A	P	0,715	P	A	P	A	P	P	P	M
1029889	COG3884I	gbs0908		Metabolism and transport	Fatty acid and phospholipids	Acyl-[acyl-carrier-protein] hydrolase (EC 3.1.2.14)	2,343	P	A	A	A	A	A	A	A	0,838	P	A	A	A	A	A	A	A
1029888	COG0647G	gbs0909		Metabolism and transport	Carbohydrates, organic alcohols, and acids	Haloacid dehalogenase-like hydrolase	0,878	P	P	P	P	P	P	P	P	0,692	P	P	P	P	P	P	P	P
1029895	-	gbs0910		Hypothetical		Hypothetical membrane spanning protein	1,947	P	A	M	A	P	P	A	A	0,750	P	A	A	A	A	P	A	A
1029893	COG3513S	gbs0911		Hypothetical		Hypothetical cytosolic protein	1,148	P	P	P	M	P	P	A	P	0,960	P	P	P	P	P	P	P	P
1029891	COG1518L	gbs0912		Hypothetical		Hypothetical cytosolic protein	2,077	P	A	A	A	A	A	A	A	1,282	P	P	P	P	P	P	P	P
1029900	COG3512S	gbs0913		Hypothetical		Hypothetical cytosolic protein	1,500	P	P	P	P	P	P	A	P	1,460	P	P	P	P	P	P	P	P
1029894	-	gbs0914		Hypothetical		Hypothetical protein	1,317	P	P	P	P	P	P	P	P	1,331	P	P	P	P	P	P	P	P
1029892	-	gbs0915		Hypothetical		Hypothetical protein	0,890	P	A	P	M	P	M	A	P	0,663	P	P	P	A	A	P	A	M
1029897	-	gbs0916	ndk	Metabolism and transport	Purines, pyrimidines, nucleosides, and nucleotides	Nucleoside diphosphate kinase (EC 2.7.4.6)	0,438	P	P	P	P	P	P	P	P	0,425	P	P	P	P	P	P	P	P
1029896	COG0481M	gbs0917	lepA	General function predicted only		GTP-binding protein lepA	1,430	P	P	P	A	P	A	A	A	0,741	P	A	A	A	A	A	A	A
1029899	COG4886S	gbs0918	inlA/blr	Cellular processess	Toxin production and resistance	Internalin protein	2,157	P	A	A	A	A	A	A	A	0,578	P	A	A	A	A	A	A	A
1029902	COG1418R	gbs0919		Cellular processess	Toxin production and resistance	Metal dependent hydrolase	4,571	P	A	P	A	P	A	P	A	1,135	P	A	A	A	A	A	A	A
1029898	-	gbs0920		Metabolism and transport	Central intermediary metabolism	Acetyltransferase (EC 2.3.1.-)	2,280	P	P	P	P	P	P	P	P	0,898	P	A	P	A	A	P	A	A
1029905	-	gbs0921	msrB	Cellular processess	Posttranslational modification, protein turnover, chaperones	Peptide methionine sulfoxide reductase msrB (EC 1.8.4.6)	1,000	P	P	P	P	P	P	P	P	0,924	P	P	P	P	P	P	P	P
1029904	COG0474P	gbs0922		Metabolism and transport	Inorganic ion transport and metabolism	Calcium-transporting ATPase (EC 3.6.3.8)	1,020	P	A	M	A	P	P	A	A	0,966	P	A	A	A	A	P	A	A
1029903	-	gbs0923		Metabolism and transport	Fatty acid and phospholipids	Lipase	2,034	P	A	P	A	A	M	M	A	1,478	P	A	P	A	A	P	A	P
1029901	COG0110R	gbs0924		General function predicted only		O-acetyltransferase (EC 2.3.1.-)	4,499	P	A	A	A	A	A	A	A	1,097	P	A	A	A	A	A	A	A
1029906	COG0398S	gbs0925		General function predicted only		Putative membrane-associated alkaline phosphatase	2,144	P	P	P	P	P	P	P	P	1,245	P	A	P	P	P	P	P	M
1029908	-	gbs0926		Transport and binding proteins	Unknown substrate	ABC transporter permease protein	0,629	P	P	P	P	P	P	P	P	0,846	P	P	P	P	P	P	P	P
1029912	COG1131V	gbs0927		Transport and binding proteins	Unknown substrate	ABC transporter ATP-binding protein	0,542	P	P	P	P	P	P	P	P	0,890	P	P	P	A	P	P	P	P
1029907	COG1725K	gbs0928		Cellular processess	Transcription	Transcriptional regulator, GntR family	0,372	P	P	P	P	P	P	P	P	0,738	P	P	P	P	P	P	P	P
1029916	COG0587L	gbs0929	dnaE	Cellular processess	DNA replication, recombination and repair	DNA polymerase III alpha subunit (EC 2.7.7.7)	4,098	P	A	A	A	A	A	A	A	4,086	P	A	A	A	A	A	A	A
1029911	COG0205G	gbs0930	pfkA	Metabolism and transport	Carbohydrates, organic alcohols, and acids	6-phosphofructokinase (EC 2.7.1.11)	1,399	P	P	P	P	P	P	P	P	0,816	P	P	P	P	P	P	P	P
1029910	-	gbs0931	pyk	Metabolism and transport	Energy production and conversion	Pyruvate kinase (EC 2.7.1.40)	1,038	P	P	P	P	P	P	P	P	0,771	P	P	P	P	P	P	P	P
1029917	-	gbs0932	sipC	Cellular processess	Posttranslational modification, protein turnover, chaperones	Signal peptidase I (EC 3.4.21.89)	0,474	P	P	P	P	P	P	P	P	0,922	P	P	P	P	P	P	P	P
1029918	COG0449M	gbs0933	glmS	Metabolism and transport	Amino acids, peptides, aminosugars and amines	Glucosamine--fructose-6-phosphate aminotransferase [isomerizing] (EC 2.6.1.16)	1,732	P	P	P	P	P	P	P	A	1,998	P	A	P	P	P	P	P	A
1029915	COG2824P	gbs0934		Metabolism and transport	Inorganic ion transport and metabolism	PhnA protein	2,192	P	A	A	A	A	A	A	A	0,437	A	A	A	A	A	A	A	A
1029909	COG0765E	gbs0935		Metabolism and transport	Amino acids, peptides, aminosugars and amines	Histidine transport system permease protein hisM	4,455	P	P	P	A	P	P	A	A	0,833	P	A	P	A	M	P	P	M
1029913	COG1126E	gbs0936		Metabolism and transport	Amino acids, peptides, aminosugars and amines	Histidine transport ATP-binding protein hisP	4,753	P	A	M	A	A	A	A	A	0,975	P	A	P	A	A	A	A	A
1029921	COG0834ET	gbs0937		Metabolism and transport	Amino acids, peptides, aminosugars and amines	Histidine-binding protein	3,303	P	P	P	A	P	P	P	P	0,610	P	A	P	P	A	P	P	P
1029920	COG0268J	gbs0938	rpsT	Cellular processess	Translation, ribosomal structure and biogenesis	SSU ribosomal protein S20P	0,659	P	P	P	P	P	P	P	P	0,700	P	P	P	P	P	P	P	P
1029914	COG1072H	gbs0939	coaA	Metabolism and transport	Cofactors, prosthetic groups, and carriers	Pantothenate kinase (EC 2.7.1.33)	2,740	P	A	P	A	P	P	M	P	1,627	P	A	P	A	M	P	P	P
1029926	COG2813J	gbs0940		Cellular processess	Translation, ribosomal structure and biogenesis	16S rRNA m(2)G 1207 methyltransferase (EC 2.1.1.52)	4,718	P	A	A	A	A	A	A	A	1,997	P	A	P	A	M	M	A	A
1029919	COG0295F	gbs0941	cdd	Metabolism and transport	Purines, pyrimidines, nucleosides, and nucleotides	Cytidine deaminase (EC 3.5.4.5)	2,620	P	P	P	M	P	P	P	P	1,109	P	A	P	P	P	P	P	P
1029925	COG1744R	gbs0942		Metabolism and transport	Purines, pyrimidines, nucleosides, and nucleotides	Nucleoside-binding protein	0,444	P	P	P	P	P	P	P	P	0,671	P	P	P	P	P	P	P	P
1029922	COG3845R	gbs0943		Metabolism and transport	Purines, pyrimidines, nucleosides, and nucleotides	Nucleoside transport ATP-binding protein	2,169	P	P	P	A	P	P	P	P	1,030	P	A	P	M	P	P	P	P

1029930	COG4603R	gbs0944		Metabolism and transport	Purines, pyrimidines, nucleosides, and nucleotides	Nucleoside transport system permease protein	2,884	P	P	P	P	P	P	P	P	1,399	P	P	P	P	P	P	P	P
1029927	COG1079R	gbs0945		Metabolism and transport	Purines, pyrimidines, nucleosides, and nucleotides	Nucleoside transport system permease protein	2,292	P	P	P	P	P	P	P	P	1,271	P	P	P	P	P	P	P	P
1029923	COG0446R	gbs0946	nox	Metabolism and transport	Energy production and conversion	NADH oxidase H2O-forming (EC 1.6.-.-)	1,231	P	P	P	P	P	P	P	P	0,617	P	P	P	P	P	P	P	P
1029928	COG0039C	gbs0947	ldh	Metabolism and transport	Energy production and conversion	L-lactate dehydrogenase (EC 1.1.1.27)	0,713	P	P	P	P	P	P	P	P	0,727	P	P	P	P	P	P	P	P
1029931	COG0188L	gbs0948	gyrA	Cellular processes	DNA replication, recombination and repair	DNA gyrase subunit A (EC 5.99.1.3)	2,581	P	P	P	P	P	P	P	P	1,706	P	P	P	P	P	P	P	P
1029933	COG3764M	gbs0949	sortA	Cellular processes	Posttranslational modification, protein turnover, chaperones	Sortase	2,584	P	M	P	A	P	P	P	P	1,849	P	A	P	P	P	P	P	P
1029924	COG0346E	gbs0950		Metabolism and transport	Amino acids, peptides, aminosugars and amines	Glyoxalase family protein	2,614	P	A	A	A	A	A	A	A	1,187	P	A	P	A	A	P	A	A
1029939	COG4086S	gbs0951		Hypothetical		Hypothetical exported protein	2,015	P	A	A	A	P	M	A	A	2,680	P	A	A	A	A	A	A	A
1029932	COG0475P	gbs0952		Metabolism and transport	Inorganic ion transport and metabolism	Na+ H+ antiporter napA	1,579	P	P	P	P	P	P	P	A	0,503	P	P	P	M	P	P	P	P
1029936	G0518F, COG0	gbs0953	guaA	Metabolism and transport	Purines, pyrimidines, nucleosides, and nucleotides	GMP synthase [glutamine-hydrolyzing] (EC 6.3.5.2)	1,827	P	P	P	A	P	P	P	P	1,154	P	A	P	A	A	P	A	A
1029934	COG2188K	gbs0954		Cellular processes	Transcription	Transcriptional regulator, GntR family	1,823	P	P	P	M	P	P	P	P	0,965	P	A	P	A	M	M	A	A
1029944	COG1206J	gbs0955	gid	Cellular processes	Translation, ribosomal structure and biogenesis	Glucose inhibited division protein A	1,278	P	P	P	P	P	P	P	P	1,235	P	A	P	P	P	P	P	P
1029935	-	gbs0956		Metabolism and transport	Central intermediary metabolism	Acetyltransferase (EC 2.3.1.-)	1,873	P	A	P	A	A	P	A	A	1,314	P	A	A	A	A	P	A	A
1029942	COG1464P	gbs0957		Transport and binding proteins	Unknown substrate	ABC transporter substrate-binding protein	1,939	P	P	P	P	P	P	P	P	2,055	P	P	P	A	P	P	P	P
1029943	-	gbs0958		Hypothetical		Hypothetical protein	1,815	P	P	P	P	P	P	P	P	0,482	P	P	P	P	P	P	P	P
1029945	COG0793M	gbs0959		Cellular processes	Toxin production and resistance	Nisin-resistance protein	0,463	P	P	P	P	P	P	P	P	0,603	P	A	A	A	A	A	A	A
1029937	COG1136V	gbs0961		Transport and binding proteins	Unknown substrate	ABC transporter ATP-binding protein	1,053	P	P	P	P	P	P	P	P	1,022	P	A	M	A	A	P	A	A
1029948	-	gbs0962	salY	Transport and binding proteins	Unknown substrate	ABC transporter permease protein	2,207	P	A	A	A	A	A	A	A	3,159	P	A	A	A	A	A	A	A
1029949	COG0745TK	gbs0963		Cellular processes	Signal transduction	Two-component response regulator	0,434	P	P	P	P	P	P	P	P	1,046	P	P	P	P	P	P	P	P
1029955	COG0642T	gbs0964		Cellular processes	Signal transduction	Two-component sensor histidine kinase (EC 2.7.3.-)	0,748	P	A	P	P	P	P	A	P	1,175	P	A	P	A	A	A	A	A
1029941	COG0582L	gbs0965	xerD	Cellular processes	DNA replication, recombination and repair	Integrase recombinase xerD	6,383	P	A	A	A	A	A	A	A	2,641	A	A	A	A	A	A	A	A
1029954	COG4166E	gbs0966		Metabolism and transport	Amino acids, peptides, aminosugars and amines	Oligopeptide-binding protein oppA	0,843	P	A	P	A	M	M	P	P	1,226	P	A	A	A	A	A	A	A
1029950	-	gbs0967	satE	Hypothetical		Hypothetical protein	1,892	P	P	P	P	P	P	P	P	1,000	P	A	P	A	A	P	A	P
1029951	-	gbs0968	satD	Hypothetical		Hypothetical protein	1,206	P	A	P	P	P	A	P	P	0,665	P	A	A	A	A	A	M	A
1029969	COG3505U	gbs0983		General function predicted only		ATPASE VIRD4 HOMOLOG	0,953	A	A	A	A	A	A	A	A	0,287	A	A	A	A	A	A	A	A
1029974	-	gbs0984		Hypothetical		Hypothetical protein	0,754	P	A	P	A	P	A	A	A	0,501	A	A	A	A	M	A	A	A
1029971	COG0629L	gbs0985		Hypothetical		Hypothetical protein	0,288	P	P	P	P	P	P	P	P	0,672	P	A	P	A	A	P	P	P
1029973	-	gbs0987		Hypothetical		Hypothetical protein	0,396	P	P	P	P	P	P	P	P	1,021	P	A	A	A	A	A	A	A
1029975	-	gbs0988		Cell Envelope	Other	M protein	0,341	P	P	P	P	P	P	P	P	0,746	P	A	P	A	A	P	A	A
1029982	-	gbs0989		Hypothetical		Hypothetical protein	0,388	P	P	P	P	P	A	P	A	1,797	P	A	A	A	A	A	A	A
1029968	-	gbs0990		Hypothetical		Hypothetical protein	0,232	P	P	P	P	P	P	P	P	0,786	P	A	A	A	A	M	A	A
1029976	COG0542O	gbs0991	clpA ?	Cellular processes	DNA replication, recombination and repair	DNA topoisomerase III (EC 5.99.1.2)	0,315	A	A	A	A	A	A	A	A	0,661	A	A	A	A	A	A	A	A
1029977	COG0550L	gbs0992		General function predicted only		LtrC-like protein	0,129	A	A	A	A	A	A	A	A	0,467	A	A	A	A	A	A	A	A
1029981	-	gbs0993		Hypothetical		Hypothetical protein	0,312	P	P	P	M	P	P	P	P	0,655	P	A	P	A	A	P	A	P
1029972	-	gbs0994		Hypothetical		Hypothetical cytosolic protein	0,622	A	A	A	A	A	A	A	A	0,197	A	A	A	A	A	A	A	A
1029986	-	gbs0995		Hypothetical		Hypothetical protein	0,512	P	A	M	A	A	A	A	A	0,761	A	A	A	A	A	A	A	A
1029980	-	gbs0996		Mobile and extrachromosomal elements		DNA-entry nuclease (EC 3.1.30.-)	0,528	P	A	P	A	A	A	A	A	0,435	A	A	A	A	A	M	A	A
1029985	-	gbs0997		Hypothetical		Hypothetical protein	0,582	P	P	P	A	A	P	A	P	0,667	P	A	A	A	A	A	A	A
1029984	-	gbs0998		Hypothetical		Hypothetical protein	0,606	P	P	P	A	P	P	P	A	1,016	P	A	P	A	A	A	A	A
1029991	-	gbs0999		Hypothetical		Hypothetical protein	0,762	P	A	P	A	P	P	A	A	2,300	P	A	A	A	A	A	A	A
1029989	-	gbs1002		Hypothetical		Hypothetical protein	0,843	P	A	P	A	A	M	A	A	0,641	M	A	A	A	A	A	A	A
1029997	-	gbs1003		Hypothetical		Hypothetical protein	0,432	P	P	P	P	P	P	P	P	0,719	P	P	P	P	P	P	P	P
1029987	-	gbs1004		Hypothetical		Hypothetical protein	0,915	P	A	A	A	A	M	A	A	0,336	P	A	P	P	A	P	A	A
1029990	-	gbs1005		Hypothetical		Hypothetical protein	0,352	A	A	A	A	A	A	A	A	0,165	A	A	A	A	A	A	A	A
1030009	COG0541U	gbs1017	ffh	Cellular processes	Posttranslational modification, protein turnover, chaperones	SIGNAL RECOGNITION PARTICLE, SUBUNIT FFH SRP54	2,017	P	P	P	M	P	P	P	P	0,897	P	A	P	A	P	P	A	A
1030013	COG2739S	gbs1018	ylxM	General function predicted only		Signal recognition particle associated protein	3,159	P	A	A	A	A	A	A	A	1,015	P	A	A	A	A	A	A	A
1030010	COG0642T	gbs1019	ciaH	Cellular processes	Signal transduction	Sensor protein ciaH (EC 2.7.3.-)	2,310	P	P	P	P	P	P	P	P	1,563	P	A	P	P	P	P	A	P
1030007	COG0745TK	gbs1020	ciaR	Cellular processes	Transcription	Transcriptional regulatory protein ciaR	1,608	P	P	P	P	P	P	P	P	1,524	P	P	P	P	P	P	P	P
1030012	COG0308E	gbs1021	pepN	Metabolism and transport	Amino acids, peptides, aminosugars and amines	Aminopeptidase N (EC 3.4.11.15)	1,466	P	P	P	P	P	P	P	P	0,707	P	P	P	P	P	P	P	P
1030011	COG0704P	gbs1022	phoU	Metabolism and transport	Inorganic ion transport and metabolism	Phosphate transport system protein phoU	1,943	P	P	P	P	P	P	P	P	1,262	P	P	P	A	P	P	P	P
1030014	COG1117P	gbs1023	pstB	Metabolism and transport	Inorganic ion transport and metabolism	Phosphate transport ATP-binding protein pstB	1,988	P	P	P	P	P	P	P	P	0,878	P	P	P	A	P	P	P	P
1030019	COG1117P	gbs1024	pstB2	Metabolism and transport	Inorganic ion transport and metabolism	Phosphate transport ATP-binding protein pstB	1,482	P	M	P	A	P	P	P	P	0,733	P	A	P	A	A	P	P	P
1030016	COG0581P	gbs1025	pstA	Metabolism and transport	Inorganic ion transport and metabolism	Phosphate transport system permease protein pstA	1,676	P	P	P	P	P	P	P	P	1,006	P	A	P	A	P	P	P	P
1030015	COG0573P	gbs1026	pstC	Metabolism and transport	Inorganic ion transport and metabolism	Phosphate transport system permease protein pstC	1,781	P	A	P	A	M	M	A	A	0,692	P	A	P	A	A	P	A	A
1030018	COG0226P	gbs1027	pstS	Metabolism and transport	Inorganic ion transport and metabolism	Phosphate-binding protein	1,605	P	P	P	P	P	P	P	P	0,614	P	M	P	P	P	P	P	P
1030020	G0144J, COG3	gbs1028		Cellular processes	Translation, ribosomal structure and biogenesis	Putative 23S rRNA m(5)C methyltransferase (EC 2.1.1.-)	2,576	P	A	A	A	A	A	A	A	1,877	P	A	A	A	A	A	A	A
1030022	COG0483G	gbs1029		Metabolism and transport	Carbohydrates, organic alcohols, and acids	Myo-inositol-1(or 4)-monophosphatase (EC 3.1.3.25)	2,911	P	P	P	P	P	P	P	P	1,163	P	P	P	P	P	P	P	P
1030031	-	gbs1030		Hypothetical		Hypothetical cytosolic protein	3,207	P	P	P	P	P	P	P	P	1,499	P	P	P	P	P	P	P	P
1030028	COG1393P	gbs1031	spxA	Metabolism and transport	Inorganic ion transport and metabolism	Arsenate reductase family protein	3,197	P	P	P	P	P	P	P	P	1,496	P	A	P	P	P	P	P	P
1030029	COG0196H	gbs1032	mreA	Metabolism and transport	Cofactors, prosthetic groups, and carriers	Riboflavin kinase (EC 2.7.1.26) FMN adenylyltransferase (EC 2.7.7.2)	2,529	P	A	A	A	A	A	M	A	1,178	P	A	M	A	A	P	A	A
1030025	COG0130J	gbs1033	truB	Cellular processes	Translation, ribosomal structure and biogenesis	tRNA pseudouridine synthase B (EC 4.2.1.70)	2,655	P	A	A	A	A	A	A	A	0,921	P	A	A	A	A	A	A	A
1030026	-	gbs1034		Metabolism and transport	Central intermediary metabolism	Acetyltransferase (EC 2.3.1.-)	0,884	P	P	P	P	P	P	P	P	1,292	P	P	P	P	P	P	P	P
1030024	COG4487S	gbs1035		Hypothetical		Hypothetical protein	1,067	P	P	P	P	P	P	P	P	1,728	P	A	P	M	P	P	P	M
1030034	COG0732V	gbs1036		Mobile and extrachromosomal elements		Type I restriction-modification system specificity subunit	0,805	P	P	P	P	P	P	P	P	1,421	P	A	P	P	A	P	P	A
1030030	-	gbs1037		Cellular processes	Posttranslational modification, protein turnover, chaperones	CAAX amino terminal protease family	2,459	P	P	P	P	P	P	P	P	2,776	P	A	P	P	P	P	P	P
1030036	COG0577V	gbs1038		Transport and binding proteins	Unknown substrate	ABC transporter permease protein	1,115	P	P	M	P	P	P	A	A	1,183	P	A	P	P	P	P	P	P
1030033	COG1136V	gbs1039		Transport and binding proteins	Unknown substrate	ABC transporter ATP-binding protein	1,012	P	P	P	P	P	P	P	P	0,903	P	A	P	P	P	P	P	P
1030032	G0550L, COG0	gbs1040		Cellular processes	DNA replication, recombination and repair	DNA topoisomerase I (EC 5.99.1.2)	2,494	P	P	P	P	P	P	P	P	0,452	P	A	P	A	A	P	A	M
1030038	COG0758LU	gbs1041	smf	Cellular processes	DNA replication, recombination and repair	DNA processing protein	3,667	P	A	A	A	A	A	A	A	0,282	A	A	A	A	A	A	A	A
1030041	COG4607P	gbs1042		Metabolism and transport	Inorganic ion transport and metabolism	Ferric anguibactin-binding protein	1,924	P	A	P	A	A	A	A	A	0,933	P	A	A	A	A	A	A	A
1030035	COG4604P	gbs1043		Metabolism and transport	Inorganic ion transport and metabolism	Ferric anguibactin transport ATP-binding protein	5,032	P	P	P	A	P	P	P	P	1,277	P	P	P	P	P	P	P	P
1030043	COG4605P	gbs1044		Metabolism and transport	Inorganic ion transport and metabolism	Ferric anguibactin transport system permease protein fatC	5,041	P	P	P	P	P	P	P	P	1,393	P	M	P	P	P	P	P	P
1030042	COG4606P	gbs1045		Metabolism and transport	Inorganic ion transport and metabolism	Ferric anguibactin transport system permease protein fatD	3,098	P	P	P	P	P	P	P	P	0,872	P	A	P	P	P	P	P	P
1030040	COG0110R	gbs1046		Metabolism and transport	Fatty acid and phospholipids	O-acetyltransferase (cell wall biosynthesis) (EC 2.3.1.-)	2,700	P	A	P	A	A	P	A	P	1,360	P	A	P	A	P	P	A	P
1030046	COG0164L	gbs1047	rnhB	Cellular processes	Transcription	Ribonuclease HII (EC 3.1.26.4)	2,572	P	A	P	P	P	P	P	P	1,391	P	A	P	P	P	P	A	P
1030047	COG1161R	gbs1048		General function predicted only		GTP-binding protein	2,190	P	A	A	A	P	M	P	P	2,087	P	A	A	A	A	P	A	A
1030048	COG1376S	gbs1049		Hypothetical		Hypothetical exported protein	0,782	P	A	P	A	A	P	A	A	0,384	P	A	P	A	A	P	A	A
1030045	COG1966T	gbs1050		Cellular processes	Signal transduction	Carbon starvation protein A	0,423	P	P	P	P	P	P	P	P	0,628	P	P	P	P	P	P	P	P
1030051	COG3279KT	gbs1051	lysS	Cellular processes	Transcription	Autolysin response regulator	1,614	P	A	A	A	A	A	A	A	1,176	P	A	A	A	A	A	A	A
1030052	COG3275T	gbs1052	lysR	Cellular processes	Signal transduction	Autolysin sensor kinase (EC 2.7.3.-)	1,035	P	P	P	P	P	P	P	P	0,925	P	A	P	P	M	P	A	P
1030044	-	gbs1053		Hypothetical		Hypothetical protein	2,212	P	A	A	A	A	P	A	P	2,158	P	A	A	A	A	A	A	A
1030050	-	gbs1054		Hypothetical		Hypothetical protein	4,618	P	A	A	A	A	P	A	A	1,533	P	A	A	A	A	P	A	A
1030053	-	gbs1055		Hypothetical		Hypothetical protein	2,995	M	A	A	A	A	A	A	A	1,370	A	A	A	A	A	A	A	A
1030057	-	gbs1056		Hypothetical		Hypothetical protein	2,416	P	P	P	P	P	P	M	P	1,120	P	A	P	A	A	P	A	A
1030054	-	gbs1057		Hypothetical		Hypothetical protein	4,938	P	A	M	A	A	A	A	A	3,496	P	A	A	A	A	A	A	A
1030067	-	gbs1061		Hypothetical		Hypothetical protein	5,401	P	M	P	P	P	A	P	A	2,280	P	A	M	M	A	A	A	A
1030056	COG4495S	gbs1062		Hypothetical		Hypothetical cytosolic protein	11,463	P	A	P	P	P	P	A	P	6,687	P	A	A	A	A	M	A	A
1030055	-	gbs1063		Hypothetical		Hypothetical protein	18,533	P	A	P	A	P	P	A	A	6,924	P	A	A	A	A	A	A	A
1030062	-	gbs1064		Hypothetical		Hypothetical protein	6,323	P	A	P	A	A	A	A	A	2,809	P	A	A	A	A	A	A	A
1030059	-	gbs1065		Hypothetical		Hypothetical protein	6,750	P	M	P	A	M	A	M	A	4,320	P	A	A	A	A	A	A	A
1030069	-	gbs1066		Hypothetical		Hypothetical protein	16,100	P	A	P	M	A	P	A	P	22,350	P	A	A	A	A	A	A	A
1030060	-	gbs1067		Hypothetical		Hypothetical protein	8,921	P	A	P	A	A	A	A	A	9,090	P	A	A	A	A	A	A	A
1030065	COG1674D	gbs1068		Cellular processes	Cell division	DNA segregation ATPase and related proteins (FtsK SpoIIIE family)	7,230	P	A	P	A	A	A	A	A	4,619	P	A	A	A	A	A	A	A
1030066	-	gbs1069		Hypothetical		Hypothetical protein	10,983	P	P	P	A	P	P	P	P	5,772	P	A	A	A	A	M	A	A
1030070	COG4499S	gbs1070		Hypothetical		Hypothetical membrane associated protein	12,144	P	A	P	A	A	A	A	A	13,660	P	A	A	A	A	A	A	A

1030073	-	gbs1071		Hypothetical		Hypothetical protein	12,675	P	A	P	A	P	P	A	A	17,038	P	A	A	A	A	A	A	A
1030071	-	gbs1072		Hypothetical		Hypothetical protein	7,790	P	P	P	P	P	P	P	P	6,331	P	A	P	A	A	M	A	A
1030077	COG1511S	gbs1073		Mobile and extrachromosomal elements		Phage infection protein	3,680	P	P	P	P	P	P	P	P	3,647	P	A	A	A	A	A	A	A
1030076	COG4842S	gbs1074		Hypothetical		Hypothetical protein	2,494	P	P	P	P	P	P	P	P	6,520	P	A	P	A	A	P	A	A
1030074	COG5153UI	gbs1075		Hypothetical		Hypothetical cytosolic protein	5,007	P	P	P	P	P	P	P	P	9,794	P	A	P	A	P	P	A	M
1030082	-	gbs1076		Hypothetical		Hypothetical secreted protein	3,703	P	P	P	P	P	P	P	P	8,187	P	A	P	A	M	P	A	P
1030084	COG0458EF	gbs1077	carB	Metabolism and transport	Purines, pyrimidines, nucleosides, and nucleotides	Carbamoyl-phosphate synthase large chain (EC 6.3.5.5)	2,112	P	P	P	P	P	P	P	P	2,226	P	P	P	A	P	P	P	P
1030081	COG0505EF	gbs1078	carA	Metabolism and transport	Purines, pyrimidines, nucleosides, and nucleotides	Carbamoyl-phosphate synthase small chain (EC 6.3.5.5)	1,940	P	P	P	P	P	P	P	P	2,707	P	A	P	A	P	P	A	M
1030083	COG0540F	gbs1079	pyrB	Metabolism and transport	Purines, pyrimidines, nucleosides, and nucleotides	Aspartate carbamoyltransferase (EC 2.1.3.2)	1,982	P	P	P	P	P	P	P	P	1,421	P	A	P	P	P	P	P	P
1030072	COG0044F	gbs1080	pyrC	Metabolism and transport	Purines, pyrimidines, nucleosides, and nucleotides	Dihydroorotase (EC 3.5.2.3)	1,200	P	P	P	P	P	P	P	P	1,285	P	A	P	M	P	P	P	P
1030087	COG0461F	gbs1081	pyrE	Metabolism and transport	Purines, pyrimidines, nucleosides, and nucleotides	Orotate phosphoribosyltransferase (EC 2.4.2.10)	1,549	P	P	P	P	P	P	P	P	0,885	P	P	P	A	P	P	P	P
1030080	COG0284F	gbs1082	pyrF	Metabolism and transport	Purines, pyrimidines, nucleosides, and nucleotides	Orotidine 5 -phosphate decarboxylase (EC 4.1.1.23)	1,662	P	P	P	P	P	P	P	P	1,056	P	P	P	P	P	P	P	P
1030085	2966S, COG3	gbs1083		Transport and binding proteins	Unknown substrate	Threonine Serine Exporter	1,729	P	P	P	A	P	P	P	A	1,026	P	A	P	A	A	A	M	A
1030093	COG0488R	gbs1084		Transport and binding proteins	Unknown substrate	ABC transporter ATP-binding protein	3,350	P	A	A	A	A	A	A	A	1,778	P	A	A	A	A	A	A	A
1030086	COG1328F	gbs1085	rnhB	Metabolism and transport	Purines, pyrimidines, nucleosides, and nucleotides	Anaerobic ribonucleoside-triphosphate reductase (EC 1.17.4.2)	0,463	P	P	P	P	P	P	P	P	0,627	P	P	P	P	P	P	P	P
1030088	COG0136E	gbs1086		Metabolism and transport	Amino acids, peptides, aminosugars and amines	Aspartate-semialdehyde dehydrogenase (EC 1.2.1.11)	2,738	P	P	P	P	P	P	P	P	4,794	P	A	P	A	A	P	A	P
1030097	-	gbs1087	fbsA	Hypothetical		LPXTG Hypothetical protein	0,654	P	P	P	P	P	P	P	P	2,824	P	P	P	P	P	P	P	P
1030090	COG1502I	gbs1088		Metabolism and transport	Fatty acid and phospholipids	Cardiolipin synthetase (EC 2.7.8.-)	1,452	P	P	P	P	P	P	P	P	1,390	P	A	P	A	M	P	M	A
1030094	COG2759F	gbs1089	fhs.1	Metabolism and transport	Purines, pyrimidines, nucleosides, and nucleotides	Formate--tetrahydrofolate ligase (EC 6.3.4.3)	0,885	P	P	P	P	P	P	P	P	0,737	P	P	P	P	P	P	P	P
1030091	COG0095H	gbs1090	lplA	Metabolism and transport	Cofactors, prosthetic groups, and carriers	Lipoate-protein ligase A (EC 6.3.2.-)	2,747	P	A	P	A	P	A	A	A	0,959	P	A	M	A	A	M	A	A
1030092	COG0846K	gbs1091		Cellular processess	Transcription	SIR2 family protein	4,364	P	A	P	A	P	P	P	A	1,059	P	A	P	A	A	P	P	P
1030095	-	gbs1092		Cellular processess	Posttranslational modification, protein turnover, chaperones	ATPase associated with chromosome architecture replication	2,398	P	M	M	A	P	P	P	A	0,837	P	A	P	A	P	P	A	A
1030102	COG0509E	gbs1093		Metabolism and transport	Amino acids, peptides, aminosugars and amines	Glycine cleavage system H protein	2,531	P	P	P	A	P	P	P	P	0,983	P	M	P	A	P	P	A	P
1030100	COG2141C	gbs1094		Metabolism and transport	Energy production and conversion	Luciferase-like monooxygenase (EC 1.14.-.-)	3,564	P	A	A	A	A	A	A	A	0,667	P	A	P	A	A	A	P	A
1030096	COG1902C	gbs1095		Metabolism and transport	Energy production and conversion	Probable NADH-dependent flavin oxidoreductase yqiG (EC 1.-.-.-)	2,311	P	A	A	A	A	A	A	A	0,734	P	A	A	A	A	A	A	A
1030101	COG0095H	gbs1096		Metabolism and transport	Cofactors, prosthetic groups, and carriers	Lipoate-protein ligase A (EC 6.3.2.-)	3,355	P	A	P	A	P	P	A	P	0,914	P	A	P	A	P	P	M	A
1030107	COG0452H	gbs1097	dpfB	Metabolism and transport	Cofactors, prosthetic groups, and carriers	Phosphopantothenate--cysteine ligase (EC 6.3.2.5)	2,984	P	A	A	A	A	A	A	A	1,439	P	A	A	A	A	A	P	A
1030103	COG0452H	gbs1098	dfp	Metabolism and transport	Cofactors, prosthetic groups, and carriers	Phosphopantothenoylcysteine decarboxylase (EC 4.1.1.36)	1,478	P	A	M	A	P	P	A	M	0,719	P	A	A	A	A	A	A	A
1030098	COG4684S	gbs1099		Hypothetical		Integral membrane protein	1,861	P	P	P	P	P	P	P	P	1,050	P	A	P	P	P	P	P	P
1030099	COG1109G	gbs1100	pgmA	Metabolism and transport	Carbohydrates, organic alcohols, and acids	Phosphoglucomutase (EC 5.4.2.2) Phosphomannomutase (EC 5.4.2.8)	0,781	P	P	P	P	P	P	P	P	1,166	P	M	P	A	P	P	P	P
1030108	-	gbs1101		Hypothetical		Hypothetical protein	2,816	P	P	P	A	P	P	P	M	1,378	P	A	P	A	P	P	A	P
1030115	COG1132V	gbs1102		Cellular processess	Toxin production and resistance	Multidrug protein lipid ABC transporter family, ATP-binding and permease protein	2,821	P	A	A	A	P	A	A	A	1,748	P	A	A	A	A	A	A	A
1030106	COG1132V	gbs1103		Cellular processess	Toxin production and resistance	Multidrug resistance ABC transporter ATP-binding and permease protein	2,541	P	A	A	A	A	A	A	A	1,613	P	A	A	A	A	A	A	A
1030105	-	gbs1104		Hypothetical		Antigen	2,708	P	A	P	A	P	P	A	P	1,697	P	A	P	A	P	P	A	A
1030119	-	gbs1105		Hypothetical		Hypothetical protein	3,144	P	P	P	A	P	P	P	A	3,545	P	A	A	A	A	P	A	A
1030114	COG0112E	gbs1106	glyA	Metabolism and transport	Amino acids, peptides, aminosugars and amines	Serine hydroxymethyltransferase (EC 2.1.2.1)	2,088	P	P	P	P	P	P	P	P	1,616	P	A	P	A	P	P	P	P
1030116	COG0009J	gbs1107		Cellular processess	Translation, ribosomal structure and biogenesis	Sua5 YciO YrdC YwlC family protein	2,104	P	P	P	P	P	P	P	P	1,318	P	A	P	A	P	P	P	A
1030111	COG2890J	gbs1108	hemK	Cellular processess	Translation, ribosomal structure and biogenesis	Peptide release factor-glutamine N5-methyltransferase (EC 2.1.1.-)	2,652	P	P	P	P	P	P	P	P	1,883	P	A	P	P	P	P	P	P
1030109	COG0216J	gbs1109	prfA	Cellular processess	Translation, ribosomal structure and biogenesis	Bacterial Peptide Chain Release Factor 1 (RF-1)	2,230	P	P	P	M	P	P	P	P	2,418	P	A	P	A	A	A	A	A
1030113	COG1435F	gbs1110	tdk2	Metabolism and transport	Purines, pyrimidines, nucleosides, and nucleotides	Thymidine kinase (EC 2.7.1.21)	1,952	P	P	P	P	P	P	P	P	1,323	P	A	P	P	P	P	P	P
1030117	COG1942R	gbs1111		Metabolism and transport	Energy production and conversion	4-oxalocrotonate tautomerase (EC 5.3.2.-)	0,373	P	P	P	P	P	P	P	P	0,240	P	P	P	A	A	P	M	A
1030118	-	gbs1112	apbE	Metabolism and transport	Cofactors, prosthetic groups, and carriers	Iron-sulfur cluster assembly repair protein ApbE	1,213	P	P	P	P	P	P	P	P	2,348	P	A	P	P	P	P	P	P
1030120	COG0431R	gbs1113		Metabolism and transport	Central intermediary metabolism	NADPH-dependent FMN reductase family protein	0,689	P	P	P	P	P	P	P	P	1,790	P	P	P	P	P	P	P	P
1030123	0431R, COG2	gbs1114		Metabolism and transport	Central intermediary metabolism	Oxidoreductase (EC 1.1.1.-)	0,751	P	M	P	P	P	P	A	P	2,624	P	A	P	P	P	P	P	P
1030125	COG2116P	gbs1115		Metabolism and transport	Inorganic ion transport and metabolism	Formate nitrite transporter family protein	1,177	P	P	P	P	P	P	M	P	1,447	P	P	P	P	P	P	P	P
1030122	COG2233F	gbs1116		Metabolism and transport	Purines, pyrimidines, nucleosides, and nucleotides	Xanthine permease	1,328	P	P	P	P	P	P	P	P	0,899	P	A	P	P	P	P	M	P
1030124	COG0503F	gbs1117	xpt	Metabolism and transport	Purines, pyrimidines, nucleosides, and nucleotides	Xanthine phosphoribosyltransferase (EC 2.4.2.-)	1,167	P	P	P	A	P	P	M	P	0,680	P	A	M	A	A	P	A	A
1030130	-	gbs1118		Hypothetical		Hypothetical protein	1,510	A	A	A	A	A	A	A	A	0,291	A	A	A	A	A	A	A	A
1030126	-	gbs1119		Hypothetical		hypothetical phage protein	0,951	P	A	A	A	A	A	A	A	0,782	P	A	A	A	A	A	A	A
1030127	0863L, COG1	gbs1120		Cellular processess	DNA replication, recombination and repair	Chromosome partitioning protein parB Adenine-specific methyltransferase (EC 2.	0,455	A	A	A	A	A	A	A	A	0,265	A	A	A	A	A	A	A	A
1030132	COG3843U	gbs1121		General function predicted only		Relaxase	0,153	A	A	A	A	A	A	A	A	0,099	A	A	A	A	A	A	A	A
1030128	-	gbs1122		Mobile and extrachromosomal elements		Mobilisation protein	0,239	A	A	A	A	A	A	A	A	0,054	A	A	A	A	A	A	A	A
1030133	-	gbs1123		Hypothetical		Hypothetical protein	0,369	A	A	A	A	A	A	A	A	0,070	A	A	A	A	A	A	A	A
1030129	-	gbs1124		Hypothetical		Hypothetical protein	0,476	A	A	A	A	A	A	A	A	0,410	A	A	A	A	A	A	A	A
1030135	-	gbs1125		Hypothetical		Hypothetical protein	0,148	A	A	A	A	A	A	A	A	0,091	A	A	A	A	A	A	A	A
1030134	-	gbs1126		General function predicted only		LtrC-like protein	0,318	A	A	A	A	A	A	A	A	0,347	A	A	A	A	A	A	A	A
1030137	-	gbs1127		Hypothetical		Hypothetical protein	0,910	A	A	A	A	A	A	A	A	0,288	A	A	A	A	A	A	A	A
1030131	COG3505U	gbs1128		Mobile and extrachromosomal elements		TraG TraD family	0,197	A	A	A	A	A	A	A	A	0,126	A	A	A	A	A	A	A	A
1030141	-	gbs1129		Hypothetical		Hypothetical protein	0,246	A	A	A	A	A	A	A	A	0,463	A	A	A	A	A	A	A	A
1030138	-	gbs1130		Hypothetical		Hypothetical protein	0,604	A	A	A	A	A	A	A	A	0,634	A	A	A	A	A	A	A	A
1030143	-	gbs1131		Hypothetical		Hypothetical protein	0,335	A	A	A	A	M	A	A	A	0,698	A	A	A	A	A	A	A	A
1030142	-	gbs1132		Hypothetical		Hypothetical protein	0,304	A	A	A	A	A	A	A	A	0,103	A	A	A	A	A	A	A	A
1030145	COG3942R	gbs1133		Hypothetical		Hypothetical protein	0,497	A	A	A	A	A	A	A	A	0,325	A	A	A	A	A	A	A	A
1030140	-	gbs1134		Hypothetical		Hypothetical protein	1,183	P	A	A	A	A	A	A	A	0,852	A	A	A	A	A	A	A	A
1030146	COG3451U	gbs1135		Hypothetical		TRSE PROTEIN	0,484	A	A	A	A	A	A	A	A	0,127	A	A	A	A	A	A	A	A
1030150	-	gbs1136		Hypothetical		Hypothetical protein	0,352	P	A	P	P	P	P	M	A	0,312	M	A	A	A	A	P	A	A
1030147	-	gbs1137		Hypothetical		Hypothetical protein	0,678	A	A	M	A	A	A	A	A	0,327	A	A	A	A	A	A	A	A
1030139	-	gbs1138		Hypothetical		Hypothetical protein	0,493	A	A	A	A	A	A	A	A	1,047	A	A	A	A	A	A	A	A
1030151	-	gbs1139		Hypothetical		Hypothetical protein	0,428	P	A	P	P	M	A	A	P	0,242	P	A	M	P	P	A	P	M
1030152	-	gbs1140		Hypothetical		Hypothetical protein	0,361	A	A	A	P	A	A	A	A	0,385	A	A	A	A	A	A	A	A
1030149	-	gbs1141		Hypothetical		Hypothetical protein	0,264	P	P	P	P	P	P	P	P	0,480	P	P	P	P	P	P	P	P
1030144	-	gbs1142		Hypothetical		Hypothetical protein	0,417	A	A	P	A	M	A	A	A	0,251	A	A	A	A	A	A	A	A
1030157	-	gbs1143	epf	Hypothetical		LPXTG Cell surface protein	0,893	A	A	A	A	A	A	A	A	2,247	A	A	A	A	A	A	A	A
1030153	-	gbs1144		Hypothetical		LPXTG Cell surface protein	0,465	A	A	P	A	A	A	A	A	0,052	A	A	A	A	A	A	A	A
1030159	-	gbs1145		Cell Envelope	Other	LPXTG M protein	0,434	A	A	A	A	A	A	A	A	0,111	A	A	A	A	A	A	A	A
1030148	-	gbs1146		Hypothetical		Hypothetical protein	0,397	A	A	A	A	P	A	P	A	0,483	A	A	A	A	A	A	A	A
1030163	COG1396K	gbs1147		Cellular processess	Transcription	Transcriptional regulator, Cro CI family	0,672	A	A	A	A	A	A	A	A	1,073	A	A	A	A	A	A	A	A
1030155	-	gbs1148		Hypothetical		Hypothetical protein	1,879	A	A	A	A	A	A	A	A	0,113	A	A	A	A	A	A	A	A
1030154	-	gbs1149		General function predicted only		Replication initiator protein	0,473	A	A	A	A	A	A	A	A	0,167	A	A	A	A	A	A	A	A
1030156	-	gbs1150		Hypothetical		Hypothetical protein	1,249	A	A	A	A	A	A	A	A	0,151	A	A	A	A	A	A	A	A
1030166	-	gbs1151		Hypothetical		Hypothetical protein	1,541	A	A	A	A	A	A	A	A	0,096	A	A	A	A	A	A	A	A
1030158	-	gbs1152		Hypothetical		Hypothetical protein	0,145	A	A	A	A	A	A	A	A	0,056	A	A	A	A	A	A	A	A
1030160	-	gbs1153		Hypothetical		Hypothetical protein	0,146	A	A	A	A	A	A	A	A	0,420	A	A	A	A	A	A	A	A
1030167	COG0516F	gbs1154	guaC	Metabolism and transport	Purines, pyrimidines, nucleosides, and nucleotides	GMP reductase (EC 1.7.1.7)	0,688	P	P	P	P	P	P	P	P	0,408	P	A	P	A	P	P	P	P
1030161	-	gbs1155		Cellular processess	Toxin production and resistance	Multidrug resistance protein B	2,515	P	A	A	A	A	A	A	A	0,519	M	A	A	A	A	M	A	A
1030165	-	gbs1156		Cellular processess	Toxin production and resistance	Na+ driven multidrug efflux pump	2,124	P	A	M	A	M	A	A	A	0,682	P	A	A	A	A	A	A	A
1030162	COG3158P	gbs1157		Metabolism and transport	Inorganic ion transport and metabolism	Kup system potassium uptake protein	2,657	P	A	A	A	P	A	A	M	0,600	P	A	A	A	M	P	A	P
1030169	COG4221R	gbs1158		Metabolism and transport	Central intermediary metabolism	Short chain dehydrogenase	0,989	P	P	P	P	P	P	P	P	1,454	P	A	P	P	P	P	P	P
1030164	COG0280C	gbs1159	pta	Metabolism and transport	Energy production and conversion	Phosphate acetyltransferase (EC 2.3.1.8)	1,548	P	P	P	P	P	P	P	P	1,136	P	P	P	P	P	P	P	P
1030171	COG0564J	gbs1160		Cellular processess	Translation, ribosomal structure and biogenesis	Ribosomal large subunit pseudouridine synthase D (EC 4.2.1.70)	3,236	P	A	A	A	A	A	A	A	1,604	P	A	A	A	A	A	A	A
1030172	COG0061G	gbs1161	ppnK	Metabolism and transport	Carbohydrates, organic alcohols, and acids	ATP-NAD kinase (EC 2.7.1.23)	3,877	P	P	A	A	P	A	A	A	1,776	P	A	P	A	A	A	A	A
1030177	COG2357S	gbs1162		Metabolism and transport	Purines, pyrimidines, nucleosides, and nucleotides	GTP pyrophosphokinase homolog	2,987	P	P	P	A	P	P	P	P	1,051	P	A	P	A	P	P	P	A
1030173	COG4116S	gbs1163		General function predicted only		Organic phosphate binding CYTH family protein	0,839	P	P	P	P	P	P	P	P	0,998	P	A	P	A	P	P	A	M
1030168	COG0462FE	gbs1164		Metabolism and transport	Purines, pyrimidines, nucleosides, and nucleotides	Ribose-phosphate pyrophosphokinase (EC 2.7.6.1)	1,192	P	P	P	P	P	P	P	P	0,809	P	P	P	P	P	P	P	P
1030175	COG1104E	gbs1165	nifS2	Metabolism and transport	Amino acids, peptides, aminosugars and amines	Cysteine desulfurase (EC 2.8.1.7) Selenocysteine lyase (EC 4.4.1.16)	1,766	P	P	P	P	P	P	P	P	1,244	P	M	P	P	P	P	P	P

1030178	-	gbs1166		Hypothetical		Hypothetical cytosolic protein	1,807	P	P	P	P	P	P	P	P	1,388	P	P	P	P	P	P	P	P
1030176	COG2344R	gbs1167		General function predicted only		AT-rich DNA-binding protein	1,478	P	P	P	P	P	P	P	P	0,404	P	P	P	P	P	P	P	P
1030180	COG2003L	gbs1168	radC	Cellular processes	DNA replication, recombination and repair	DNA repair protein radC	0,715	A	A	A	A	A	A	A	A	0,834	A	A	A	A	A	A	A	A
1030179	COG0628R	gbs1169		Hypothetical		Hypothetical membrane spanning protein	1,165	P	P	P	P	P	P	P	P	1,150	P	A	P	P	M	P	P	P
1030181	-	gbs1170		Metabolism and transport	Carbohydrates, organic alcohols, and acids	6-phospho-beta-glucosidase (EC 3.2.1.86)	0,819	P	A	A	A	A	A	A	A	0,415	P	A	P	A	A	A	A	A
1030185	COG2755E	gbs1171		Metabolism and transport	Fatty acid and phospholipids	Lipase Acylhydrolase family protein	1,585	P	P	P	P	P	P	P	P	0,769	P	P	P	P	P	P	P	P
1030182	COG0561R	gbs1172		Metabolism and transport	Central intermediary metabolism	Hydrolase (HAD superfamily)	1,336	P	P	P	A	P	P	P	P	0,732	P	A	P	A	P	P	P	P
1030192	-	gbs1173		Cellular processes	Transcription	Transcriptional regulator, AraC family	7,249	P	A	A	A	A	A	A	A	3,357	P	A	A	A	A	A	A	A
1030186	COG0038P	gbs1174		Metabolism and transport	Inorganic ion transport and metabolism	Chloride channel protein	1,465	P	P	P	P	P	P	P	P	0,781	P	M	P	P	P	P	P	P
1030184	COG0687E	gbs1175		Metabolism and transport	Amino acids, peptides, aminosugars and amines	Spermidine putrescine-binding protein	2,270	P	P	P	M	M	P	M	P	0,673	P	A	P	A	P	P	P	P
1030190	COG1177E	gbs1176	potC	Metabolism and transport	Amino acids, peptides, aminosugars and amines	Spermidine putrescine transport system permease protein potC	3,529	P	P	P	A	P	P	P	P	0,814	P	A	P	A	P	P	P	P
1030194	COG1176E	gbs1177	potB	Metabolism and transport	Amino acids, peptides, aminosugars and amines	Spermidine putrescine transport system permease protein potB	4,001	P	P	P	P	P	P	P	P	0,904	P	P	P	P	P	P	P	P
1030187	COG3842E	gbs1178	potA	Metabolism and transport	Amino acids, peptides, aminosugars and amines	Spermidine putrescine transport ATP-binding protein potA	2,691	P	A	A	A	A	A	A	A	0,777	P	A	A	A	A	A	A	A
1030189	COG0812M	gbs1179	murB	Cell Envelope	Biosynthesis and degradation of murein sacculus and peptidoglycan	UDP-N-acetylenolpyruvoylglucosamine reductase (EC 1.1.1.158)	2,452	P	A	M	A	P	A	A	A	0,566	P	A	P	A	A	P	A	A
1030195	COG0801H	gbs1180	folK	Metabolism and transport	Cofactors, prosthetic groups, and carriers	2-amino-4-hydroxy-6-hydroxymethyldihydropteridine pyrophosphokinase (EC 2.7.6	1,580	P	M	P	P	P	P	P	P	1,292	P	A	P	M	A	P	A	A
1030197	COG1539H	gbs1181	folQ	Metabolism and transport	Cofactors, prosthetic groups, and carriers	Dihydroneopterin aldolase (EC 4.1.2.25)	3,101	P	P	P	A	P	P	A	A	1,698	P	A	P	A	A	P	A	M
1030193	COG0294H	gbs1182	folP	Metabolism and transport	Cofactors, prosthetic groups, and carriers	Dihydropteroate synthase (EC 2.5.1.15)	2,635	P	M	P	A	P	P	A	M	3,596	P	A	P	A	A	A	A	A
1030198	COG0302H	gbs1183	folE	Metabolism and transport	Cofactors, prosthetic groups, and carriers	GTP cyclohydrolase I (EC 3.5.4.16)	1,834	P	A	M	A	P	P	A	A	1,038	P	A	P	A	A	P	A	A
1030196	COG0285H	gbs1184	folC.1	Metabolism and transport	Cofactors, prosthetic groups, and carriers	Folylpolyglutamate synthase (EC 6.3.2.17) Dihydrofolate synthase (EC 6.3.2.12)	2,238	P	P	P	P	P	P	P	P	1,213	P	A	P	P	P	P	A	A
1030203	COG2962R	gbs1185		General function predicted only		Chloramphenicol-sensitive protein rarD	3,738	P	A	P	A	P	P	A	A	1,182	P	A	P	A	M	P	A	A
1030201	COG0083E	gbs1186		Metabolism and transport	Amino acids, peptides, aminosugars and amines	Homoserine kinase (EC 2.7.1.39)	2,014	P	P	P	A	P	P	P	P	0,809	P	A	P	A	A	P	A	A
1030200	COG0460E	gbs1187		Metabolism and transport	Amino acids, peptides, aminosugars and amines	Homoserine dehydrogenase (EC 1.1.1.3)	1,791	P	A	P	P	P	P	P	P	0,665	P	A	P	A	P	P	P	A
1030199	-	gbs1188		Cell Envelope	Biosynthesis and degradation of surface polysaccharides and lipopo	Polysaccharide deacetylase	1,198	P	P	P	A	P	P	P	M	0,901	P	A	P	A	P	A	A	A
1030205	COG1292M	gbs1191		Metabolism and transport	Amino acids, peptides, aminosugars and amines	Glycine betaine transporter	1,192	P	P	P	P	P	P	P	P	1,161	P	P	P	P	P	P	P	P
1030211	COG1012C	gbs1192	gabD	Metabolism and transport	Energy production and conversion	Succinate-semialdehyde dehydrogenase [NADP+] (EC 1.2.1.16)	0,897	P	P	P	P	P	P	P	P	0,878	P	P	P	P	P	P	P	P
1030206	-	gbs1193		Hypothetical		Hypothetical membrane spanning protein	1,260	P	P	P	A	A	A	P	A	1,231	P	A	A	A	A	A	A	A
1030213	-	gbs1194		Hypothetical		Hypothetical protein	0,515	P	P	P	P	P	P	P	P	1,037	P	P	P	P	P	P	P	P
1030214	-	gbs1195	ska	General function predicted only		streptokinase	0,254	P	P	P	P	P	P	P	P	2,779	P	P	P	P	P	P	P	P
1030207	COG1476K	gbs1196		Cellular processes	Transcription	Transcriptional regulator, Cro CI family	2,521	P	A	A	A	A	A	A	A	1,813	A	A	A	A	A	A	A	A
1030212	-	gbs1197		Hypothetical		Hypothetical protein	1,248	P	A	P	A	A	A	P	A	0,986	P	A	A	A	A	P	A	A
1030210	COG2077O	gbs1198	tpx	Metabolism and transport	Energy production and conversion	Thioredoxin peroxidase (EC 1.11.1.15)	0,577	P	P	P	P	P	P	P	P	0,651	P	P	P	P	P	P	P	P
1030217	COG4912L	gbs1199		Hypothetical		Hypothetical protein	1,564	P	A	A	A	A	P	A	P	1,450	P	A	A	A	A	A	A	A
1030218	COG0561R	gbs1200		Metabolism and transport	Central intermediary metabolism	Hydrolase (HAD superfamily)	1,662	P	P	P	P	P	P	P	P	0,864	P	P	P	P	P	P	P	P
1030216	ò0490P, COG2	gbs1201		Metabolism and transport	Inorganic ion transport and metabolism	NAD-dependent K+ or Na+ uptake system component Transcriptional regulator, (	1,546	P	P	P	P	P	P	P	P	1,146	P	P	P	P	P	P	P	P
1030219	-	gbs1202		Cellular processes	Posttranslational modification, protein turnover, chaperones	General stress protein, Gls24 family	0,534	P	P	P	P	P	P	P	P	0,503	P	P	P	P	P	P	P	P
1030221	-	gbs1203		Hypothetical		Hypothetical protein	0,427	P	P	P	P	P	P	P	P	0,416	P	P	P	P	P	P	P	P
1030215	-	gbs1204		Cellular processes	Posttranslational modification, protein turnover, chaperones	General stress protein, Gls24 family	0,493	P	P	P	P	P	P	P	P	0,507	P	P	P	P	P	P	P	P
1030223	-	gbs1205		General function predicted only		Small integral membrane protein	0,553	P	P	P	P	P	P	P	P	0,556	P	P	P	P	P	P	P	P
1030222	-	gbs1206		Hypothetical		Hypothetical protein	0,572	P	P	P	P	P	P	P	P	0,595	P	P	P	P	P	P	P	P
1030224	COG2261S	gbs1207		Hypothetical		Integral membrane protein	0,489	P	P	P	P	P	P	P	P	0,566	P	P	P	P	P	P	P	P
1030220	COG2261S	gbs1208		Hypothetical		Integral membrane protein	0,410	P	P	P	P	P	P	P	P	0,425	P	P	P	P	P	P	P	P
1030227	COG0210L	gbs1209	pcrA	Cellular processes	DNA replication, recombination and repair	DNA helicase II (EC 3.6.1.-)	1,147	P	A	P	A	P	A	P	A	0,345	M	A	P	A	P	P	A	A
1030226	COG2050Q	gbs1210		Metabolism and transport	Secondary metabolites	Thioesterase superfamily protein	2,497	P	P	P	P	M	P	P	P	0,986	P	A	P	P	P	P	A	A
1030225	COG2233F	gbs1211	pyrP	Metabolism and transport	Purines, pyrimidines, nucleosides, and nucleotides	Uracil permease	2,895	P	P	P	P	P	P	P	P	1,222	P	P	P	A	P	P	P	P
1030230	COG1115E	gbs1212		Metabolism and transport	Amino acids, peptides, aminosugars and amines	Na(+)-linked D-alanine glycine permease	1,773	P	A	A	A	A	A	A	A	0,519	A	A	A	A	A	A	A	A
1030229	COG0053P	gbs1213		Metabolism and transport	Inorganic ion transport and metabolism	Cobalt-zinc-cadmium resistance protein czcD	2,061	P	P	P	P	P	P	M	P	0,895	P	A	M	A	A	A	A	A
1030228	COG1380R	gbs1214		Cell Envelope	Biosynthesis and degradation of murein sacculus and peptidoglycan	Murein hydrolase exporter	2,536	P	A	A	A	M	A	A	A	0,331	A	A	A	A	A	A	A	A
1030236	-	gbs1215		Cell Envelope	Biosynthesis and degradation of murein sacculus and peptidoglycan	Murein hydrolase export regulator	1,890	P	P	P	A	A	P	A	A	0,425	P	A	A	A	A	A	A	A
1030234	-	gbs1216		Mobile and extrachromosomal elements		Phage protein	0,804	P	P	P	P	P	P	P	P	1,355	P	A	P	P	P	P	P	P
1030237	-	gbs1217		Hypothetical		Hypothetical protein	0,582	A	A	A	A	A	A	A	A	0,074	A	A	A	A	A	A	A	A
1030233	-	gbs1218		Hypothetical		Hypothetical protein	1,903	P	M	P	P	P	P	P	A	1,172	P	A	P	A	M	P	A	A
1030242	-	gbs1219		Mobile and extrachromosomal elements		Phage protein	0,127	A	A	A	A	A	A	A	A	0,035	A	A	A	A	A	A	A	A
1030241	-	gbs1220		Hypothetical		Hypothetical protein	2,419	P	M	P	P	P	P	P	A	0,896	P	A	P	A	P	P	A	A
1030240	COG2932K	gbs1221		Cellular processes	Transcription	Phage transcriptional repressor	0,738	P	P	P	P	P	P	P	P	1,263	P	P	P	P	P	P	P	P
1030239	-	gbs1222		Mobile and extrachromosomal elements		Phage protein	1,124	P	P	P	P	P	P	P	P	1,805	P	A	P	P	P	P	P	P
1030238	COG4823V	gbs1223		Hypothetical		Hypothetical protein	1,266	P	A	P	P	P	A	P	P	0,912	P	A	A	A	P	P	P	M
1030246	COG0582L	gbs1224	int.3	Mobile and extrachromosomal elements		DNA integration recombination inversion protein	0,727	P	A	P	P	M	P	A	P	0,438	P	M	P	A	P	P	A	P
1030244	COG0539J	gbs1225		Cellular processes	Translation, ribosomal structure and biogenesis	SSU ribosomal protein S1P	1,260	P	P	P	P	P	P	P	P	0,508	P	P	P	M	P	P	P	P
1030250	-	gbs1226		Hypothetical		Hypothetical cytosolic protein	1,970	P	P	P	P	P	P	P	P	1,662	P	A	M	A	A	A	P	A
1030248	COG0115EH	gbs1227	bcaT	Metabolism and transport	Amino acids, peptides, aminosugars and amines	Branched-chain amino acid aminotransferase (EC 2.6.1.42)	1,036	P	P	P	P	P	P	P	P	1,436	P	M	P	P	P	P	P	P
1030251	COG0188L	gbs1228	parC	Cellular processes	DNA replication, recombination and repair	Topoisomerase IV subunit A (EC 5.99.1.-)	2,595	P	A	P	A	A	P	M	A	1,351	P	A	A	A	A	A	A	A
1030247	COG0187L	gbs1229	parE	Cellular processes	DNA replication, recombination and repair	Topoisomerase IV subunit B (EC 5.99.1.-)	2,328	P	M	A	A	P	A	A	A	0,514	P	A	A	A	A	A	A	M
1030254	COG0344S	gbs1230		Hypothetical		Hypothetical membrane spanning protein	1,073	P	P	P	P	P	P	P	P	1,175	P	P	P	P	P	P	P	P
1030253	COG0692L	gbs1231	ung	Cellular processes	DNA replication, recombination and repair	Uracil-DNA glycosylase (EC 3.2.2.-)	1,696	P	P	P	P	P	P	P	P	0,890	P	P	P	P	P	P	P	P
1030255	-	gbs1232		Hypothetical		Hypothetical protein	1,941	P	P	P	P	P	P	P	P	0,961	P	P	P	P	P	P	P	P
1030252	1083M, COG2	gbs1233	neuA	General function predicted only		Acylneuraminate cytidylyltransferase (EC 2.7.7.43)	3,131	P	A	M	A	A	P	A	A	1,584	P	A	P	A	A	P	A	P
1030259	COG0110R	gbs1234	neuD	Cell Envelope	Biosynthesis and degradation of surface polysaccharides and lipopo	Sialic acid biosynthesis protein NeuD	2,814	P	P	P	P	P	P	P	P	1,359	P	P	P	P	P	P	P	P
1030260	COG0381M	gbs1235	neuC	Cell Envelope	Biosynthesis and degradation of surface polysaccharides and lipopo	UDP-N-acetylglucosamine 2-epimerase (N-acetylmannosamine-forming) (EC 5.1.3	3,511	P	A	P	A	P	P	P	P	1,521	P	A	P	P	P	P	P	P
1030261	COG2089M	gbs1236	neuB	Cell Envelope	Biosynthesis and degradation of surface polysaccharides and lipopo	N-acetylneuraminate synthase (EC 2.5.1.56)	1,231	P	P	P	P	P	P	P	P	1,054	P	P	P	P	P	P	P	P
1030258	COG2244R	gbs1237	cpsM	Cell Envelope	Biosynthesis and degradation of surface polysaccharides and lipopo	Capsular polysaccharide protein CpsK	5,682	P	A	A	A	M	A	A	A	1,692	P	A	P	A	M	P	A	A
1030256	-	gbs1237.1	cpsL	Cell Envelope	Biosynthesis and degradation of surface polysaccharides and lipopo	beta-D-Galp alpha-2,3-sialyltransferase (EC 2.4.99.-)	4,020	P	A	A	A	P	A	A	A	1,619	P	A	P	A	P	P	A	A
1030262	COG0463M	gbs1238	CpsIaJ	Cell Envelope	Biosynthesis and degradation of surface polysaccharides and lipopo	beta-D-GlcNAc beta-1,4-galactosyltransferase (EC 2.4.1.-)	4,919	P	A	P	A	P	P	A	A	1,671	P	A	P	A	P	P	A	A
1030263	COG0463M	gbs1239	cpsJ	Cell Envelope	Biosynthesis and degradation of surface polysaccharides and lipopo	beta-D-Galp beta-1,3-N-acetylglucosaminyltransferase (EC 2.4.1.-)	4,724	P	P	P	A	P	P	P	P	1,448	P	P	P	P	P	P	P	P
1030265	-	gbs1240	cpsI	General function predicted only		Secreted polysaccharide polymerase	5,582	P	M	A	P	P	P	A	P	1,235	P	P	P	P	P	P	P	P
1030267	-	gbs1241	cpsG	Cell Envelope	Biosynthesis and degradation of surface polysaccharides and lipopo	beta-D-Glcp beta-1,4-galactosyltransferase (EC 2.4.1.-)	3,344	P	A	A	A	A	A	A	A	1,237	P	A	A	A	A	P	M	P
1030269	COG0707M	gbs1242	cpsF	Metabolism and transport	Carbohydrates, organic alcohols, and acids	Beta-1,4-galactosyltransferase accessory protein	3,259	P	P	P	A	P	P	A	P	1,226	P	A	P	P	P	P	P	P
1030266	COG2148M	gbs1243	cpsE	Cell Envelope	Biosynthesis and degradation of surface polysaccharides and lipopo	Undecaprenyl-phosphate beta-glucosephosphotransferase (EC 2.7.8.-)	2,373	P	P	P	A	P	P	P	P	0,840	P	P	P	P	P	P	P	P
1030264	COG0489D	gbs1244	cpsD	Cell Envelope	Biosynthesis and degradation of surface polysaccharides and lipopo	Tyrosine-protein kinase (capsular polysaccharide biosynthesis)	1,842	P	P	P	P	P	P	P	P	1,133	P	P	P	P	P	P	P	P
1030272	COG3944M	gbs1245	cpsC	Cellular processes	Cell division	Chain length regulator (capsular polysaccharide biosynthesis)	2,311	P	P	P	P	P	P	P	P	1,104	P	A	P	P	P	P	P	P
1030270	COG4464GM	gbs1246	cpsB	Cell Envelope	Biosynthesis and degradation of surface polysaccharides and lipopo	Phosphotyrosine-protein phosphatase (capsular polysaccharide biosynthesis) (EC	3,753	P	P	P	P	P	P	P	P	1,387	P	P	P	A	P	P	P	P
1030271	COG1316K	gbs1247	cpsA	Cellular processes	Transcription	Transcriptional activator CpsA	2,402	P	P	P	P	P	P	P	P	1,056	P	P	P	P	P	P	P	P
1030274	COG0583K	gbs1248	cpsY	Cellular processes	Transcription	Transcriptional regulators, LysR family	1,604	P	P	P	P	P	M	P	M	0,797	P	A	P	M	A	M	A	A
1030275	-	gbs1250	cpsX	Cellular processes	Signal transduction	Histidine protein kinase	1,439	P	P	P	P	P	P	P	P	1,071	P	P	P	P	P	P	P	P
1030276	COG0813F	gbs1251	deoD2	Metabolism and transport	Purines, pyrimidines, nucleosides, and nucleotides	Purine nucleoside phosphorylase (EC 2.4.2.1)	1,483	P	P	P	P	P	P	P	P	1,264	P	P	P	P	P	P	P	P
1030273	COG0038P	gbs1252		Metabolism and transport	Inorganic ion transport and metabolism	Chloride channel protein	1,939	P	P	P	P	P	P	P	P	1,219	P	P	P	P	P	P	P	P
1030279	COG0005F	gbs1253	punA	Metabolism and transport	Purines, pyrimidines, nucleosides, and nucleotides	Purine nucleoside phosphorylase (EC 2.4.2.1)	1,571	P	P	P	P	P	P	P	P	1,293	P	P	P	P	P	P	P	P
1030278	COG1393P	gbs1254	arsC	Metabolism and transport	Inorganic ion transport and metabolism	Arsenate reductase (EC 1.20.4.1)	1,373	P	P	P	P	P	P	P	P	1,138	P	P	P	P	P	P	P	P
1030280	COG1015G	gbs1255		Metabolism and transport	Carbohydrates, organic alcohols, and acids	Phosphopentomutase (EC 5.4.2.7)	1,298	P	P	P	P	P	P	P	P	1,154	P	P	P	P	P	P	P	P
1030277	COG0120G	gbs1256	rpiA	Metabolism and transport	Carbohydrates, organic alcohols, and acids	Ribose 5-phosphate isomerase (EC 5.3.1.6)	1,732	P	P	P	P	P	P	P	P	1,111	P	P	P	P	P	P	P	P
1030282	COG5549O	gbs1257		Cellular processes	Posttranslational modification, protein turnover, chaperones	Endopeptidase, M10 family (EC 3.4.24.-)	2,526	P	P	P	P	P	P	P	P	1,155	P	M	P	P	P	P	P	P
1030281	COG0627R	gbs1258	estA	General function predicted only		Acetyl esterase (EC 3.1.1.-)	2,077	P	P	P	P	P	P	P	P	1,434	P	A	P	A	P	P	P	P
1030283	COG0595R	gbs1259		General function predicted only		Zn-dependent hydrolase (EC 3.-.-.-)	1,822	P	P	P	A	P	P	P	P	0,920	P	M	P	A	P	P	P	P
1030287	COG1101R	gbs1260		Transport and binding proteins	Unknown substrate	ABC transporter ATP-binding protein	0,914	P	P	M	A	P	P	A	A	0,531	P	A	A	A	A	A	A	A
1030288	COG4120R	gbs1261		Transport and binding proteins	Unknown substrate	ABC transporter permease protein	1,588	P	A	A	A	A	A	A	A	1,014	M	A	A	A	A	A	A	A
1030290	COG2984R	gbs1262		Transport and binding proteins	Unknown substrate	ABC transporter substrate-binding protein	0,843	P	P	P	P	P	P	P	P	0,412	P	A	P	P	M	P	A	P

1030285	COG1293K	gbs1263	fbp	Cell Envelope	Other	Fibronectin-binding protein Fibrinogen-binding protein	1,432	P	P	P	P	P	P	P	P	1,747	P	A	A	A	A	A	A	A
1030284	COG3527Q	gbs1264		Metabolism and transport	Secondary metabolites	Alpha-acetolactate decarboxylase (EC 4.1.1.5)	1,584	P	P	P	P	P	P	P	P	1,543	P	P	P	P	P	P	P	P
1030291	COG0028EH	gbs1265		Metabolism and transport	Amino acids, peptides, aminosugars and amines	Acetolactate synthase (EC 2.2.1.6)	0,949	P	P	P	P	P	P	P	P	0,869	P	P	P	P	P	P	P	P
1030293	-	gbs1266		Hypothetical		Tetratricopeptide repeat family protein	2,186	P	A	P	A	P	A	P	A	1,463	P	A	A	A	A	P	A	A
1030289	COG0628R	gbs1267		Hypothetical		Hypothetical membrane spanning protein	1,848	P	M	P	P	P	P	P	P	0,985	P	A	P	A	P	P	P	P
1030295	COG0494LR	gbs1268		Cellular processess	DNA replication, recombination and repair	Phosphohydrolase (MutT nudix family protein)	1,329	P	P	P	A	M	P	A	A	0,809	P	A	A	A	A	M	A	A
1030296	COG0494LR	gbs1269		General function predicted only		7,8-dihydro-8-oxoguanine-triphosphatase (EC 3.6.1.-)	1,162	P	P	P	P	P	P	P	P	1,002	P	A	P	A	P	P	M	P
1030294	-	gbs1270	hylB/A	General function predicted only		Hyaluronate lyase precursor (EC 4.2.2.1)	1,027	P	A	P	A	A	A	P	A	0,903	P	A	A	A	A	A	P	A
1030292	COG1088M	gbs1271	cpsFQ	Metabolism and transport	Carbohydrates, organic alcohols, and acids	dTDP-glucose 4,6-dehydratase (EC 4.2.1.46)	1,120	P	P	P	A	P	P	P	P	1,363	P	A	P	M	A	P	P	A
1030299	COG1898M	gbs1272	rmlC	Metabolism and transport	Carbohydrates, organic alcohols, and acids	dTDP-4-dehydrorhamnose 3,5-epimerase (EC 5.1.3.13)	1,200	P	P	P	P	P	P	P	P	1,302	P	P	P	P	P	P	P	P
1030298	COG1209M	gbs1273	rmlA	Metabolism and transport	Carbohydrates, organic alcohols, and acids	Glucose-1-phosphate thymidylyltransferase (EC 2.7.7.24)	0,877	P	P	P	P	P	P	P	P	1,222	P	P	P	M	P	P	P	P
1030300	COG0665E	gbs1274		Metabolism and transport	Amino acids, peptides, aminosugars and amines	Glycine D-amino acid oxidases family	2,082	P	A	A	A	A	A	A	A	1,306	P	A	A	A	A	A	A	A
1030297	COG0327S	gbs1275		General function predicted only		NIF3-related protein	1,173	P	M	P	A	P	P	A	M	1,058	P	A	A	A	A	P	A	A
1030303	COG2384R	gbs1276		Hypothetical		Hypothetical cytosolic protein	2,067	P	M	P	P	P	P	P	P	1,030	P	A	P	A	A	P	A	A
1030302	COG3935L	gbs1277	dnaD	Cellular processess	DNA replication, recombination and repair	DNA replication protein dnaD	5,373	P	A	A	A	A	A	A	A	1,168	P	A	A	A	A	A	A	A
1030304	COG0503F	gbs1278	apt	Metabolism and transport	Purines, pyrimidines, nucleosides, and nucleotides	Adenine phosphoribosyltransferase (EC 2.4.2.7)	1,680	P	P	P	P	P	P	P	P	0,696	P	P	P	P	P	P	P	P
1030301	-	gbs1279		Cell Envelope	Other	M protein	0,956	P	P	P	P	P	P	P	P	1,913	P	A	P	P	P	P	P	P
1030307	COG0608L	gbs1280	recJ	Cellular processess	DNA replication, recombination and repair	Single-stranded-DNA-specific exonuclease recJ (EC 3.1.-.-)	1,900	P	A	A	A	A	A	P	A	0,838	P	A	M	A	A	M	A	A
1030308	COG0300R	gbs1281		Metabolism and transport	Central intermediary metabolism	Short chain dehydrogenase	1,615	P	P	P	P	P	P	P	P	0,910	P	M	P	P	P	P	P	P
1030306	COG1234R	gbs1282	elaC	Cellular processess	Transcription	Ribonuclease Z (EC 3.1.26.11)	1,452	P	P	P	P	P	P	P	P	0,976	P	P	P	P	P	P	P	P
1030305	-	gbs1283		Metabolism and transport	Carbohydrates, organic alcohols, and acids	galactose 1-phosphate uridylyltransferase	2,484	P	P	P	P	P	P	P	P	1,427	P	P	P	P	P	P	P	A
1030311	COG2262R	gbs1284	hflX	General function predicted only		GTP-binding protein hflX	1,088	P	P	P	P	P	P	P	P	0,817	P	P	P	P	P	P	A	A
1030309	COG0324J	gbs1285	miaA	Cellular processess	Translation, ribosomal structure and biogenesis	tRNA delta(2)-isopentenylpyrophosphate transferase (EC 2.5.1.8)	0,511	P	P	P	P	P	P	P	P	0,772	P	A	P	P	P	A	A	P
1030310	-	gbs1286		Hypothetical		Hypothetical protein	0,415	P	P	P	P	P	P	P	P	0,544	P	P	P	P	P	P	P	P
1030314	COG1275P	gbs1287	exfA	Hypothetical		Hypothetical membrane-spanning protein	0,963	P	A	A	A	A	A	A	A	0,784	P	A	P	A	P	A	A	A
1030315	COG1523G	gbs1288		Metabolism and transport	Carbohydrates, organic alcohols, and acids	LPXTG Pullulanase (EC 3.2.1.41)	0,335	A	A	A	A	A	P	A	A	1,747	P	P	A	A	A	P	P	A
1030313	COG1387ER	gbs1289		Metabolism and transport	Amino acids, peptides, aminosugars and amines	Histidinol-phosphatase (EC 3.1.3.15)	2,666	P	A	P	A	P	P	A	A	4,167	P	A	A	A	A	A	A	A
1030312	COG1573L	gbs1290		Cellular processess	DNA replication, recombination and repair	Uracil DNA glycosylase superfamily protein	2,828	P	P	P	A	P	P	M	P	3,926	P	A	M	A	A	A	A	A
1030317	COG0624E	gbs1291		Metabolism and transport	Amino acids, peptides, aminosugars and amines	Xaa-His dipeptidase (EC 3.4.13.3)	0,889	P	P	P	P	P	P	P	P	1,409	P	P	P	P	P	P	P	P
1030316	COG0778C	gbs1292		Metabolism and transport	Energy production and conversion	NAD(P)H-dependent quinone reductase (EC 1.-.-.-)	1,131	P	P	P	P	P	P	P	P	1,916	P	P	P	P	P	P	P	P
1030319	0584C, COG4	gbs1293		Metabolism and transport	Energy production and conversion	glycerophosphoryl diester phosphodiesterase	1,584	P	P	P	M	M	A	A	P	0,534	P	A	P	P	A	P	A	A
1030322	COG0322L	gbs1294	uvrC	Cellular processess	DNA replication, recombination and repair	Excinuclease ABC subunit C	2,123	P	A	A	A	A	A	A	A	1,111	P	A	A	A	A	A	A	A
1030320	COG1636S	gbs1295		Hypothetical		Hypothetical cytosolic protein	1,906	M	A	A	A	A	A	A	A	1,663	A	A	A	A	A	A	A	A
1030321	COG0534V	gbs1296		Cellular processess	Toxin production and resistance	Na+ driven multidrug efflux pump	1,789	P	A	A	A	A	A	A	A	1,135	A	A	A	A	A	A	A	A
1030318	COG2764S	gbs1297		Metabolism and transport	Energy production and conversion	PhnB protein	1,090	P	P	P	P	P	P	P	P	0,866	P	P	P	P	P	P	P	P
1030327	-	gbs1298		Hypothetical		hypothetical protein	2,300	P	P	P	P	P	P	P	P	0,890	P	P	P	P	P	P	P	P
1030325	-	gbs1299		Mobile and extrachromosomal elements		PUTATIVE HOST CELL SURFACE-EXPOSED LIPOPROTEIN	2,618	P	A	M	A	P	P	M	A	1,645	P	A	A	A	A	A	A	A
1030323	COG2801L	gbs1301		Mobile and extrachromosomal elements		Transposase	0,273	A	A	A	A	A	A	A	A	0,162	A	A	A	A	A	A	A	A
1030324	-	gbs1302		Mobile and extrachromosomal elements		Transposase	1,035	A	A	A	A	A	A	A	A	0,126	A	A	A	A	A	A	A	A
1030329	-	gbs1306		Mobile and extrachromosomal elements		Streptococcal histidine triad protein	4,656	A	A	A	A	A	A	A	A	3,449	A	A	A	A	A	A	A	A
1030333	COG0803P	gbs1307	lmb	Metabolism and transport	Inorganic ion transport and metabolism	Laminin-binding surface protein	0,871	P	A	A	A	A	A	A	A	1,312	P	A	A	A	A	M	A	A
1030336	COG1404O	gbs1308	scpB	Cellular processess	Posttranslational modification, protein turnover, chaperones	LPXTN C5A peptidase precursor (EC 3.4.21.-)	1,928	P	M	P	M	P	P	P	P	1,628	P	A	P	M	M	P	A	A
1030332	COG2176L	gbs1312		Hypothetical		Hypothetical protein	4,233	P	A	A	A	A	A	A	A	1,294	A	A	A	A	A	A	A	A
1030342	-	gbs1313		Hypothetical		Hypothetical protein	2,835	P	P	A	P	A	P	P	M	0,689	P	A	P	A	A	P	P	A
1030339	COG0582L	gbs1314		Mobile and extrachromosomal elements		DNA integration recombination inversion protein	1,160	P	P	P	P	P	P	P	P	2,731	P	P	P	P	P	P	P	P
1030338	-	gbs1315		Hypothetical		Hypothetical protein	1,091	P	P	P	P	P	P	P	P	1,900	P	P	P	P	P	P	P	P
1030351	-	gbs1316		Mobile and extrachromosomal elements		Replication protein	0,346	M	A	A	A	P	M	A	A	0,361	A	A	A	A	A	A	A	A
1030348	-	gbs1318		Hypothetical		Hypothetical protein	0,152	A	A	A	A	A	A	A	A	0,141	A	A	A	A	A	A	A	A
1030343	COG1674D	gbs1320		Cellular processess	Cell division	FtsK SpoIIIE family	0,455	A	A	A	A	A	A	A	A	0,104	A	A	A	A	A	A	A	A
1030357	-	gbs1321		Hypothetical		Hypothetical protein	0,435	P	M	A	P	A	A	A	A	0,113	A	A	A	A	A	A	A	A
1030354	-	gbs1322		Hypothetical		Hypothetical protein	1,371	P	A	A	A	A	A	A	A	1,904	P	A	A	A	A	A	A	A
1030361	-	gbs1323		Hypothetical		Hypothetical protein	1,624	M	A	A	A	A	A	A	A	1,165	P	A	A	A	A	A	A	A
1030353	-	gbs1324	hsdM	Mobile and extrachromosomal elements		TYPE II RESTRICTION-MODIFICATION SYSTEM MODIFICATION SUBUNIT	0,375	A	A	A	A	A	A	A	A	0,220	A	A	A	A	A	A	A	A
1030364	COG0582L	gbs1325		Mobile and extrachromosomal elements		DNA integration recombination inversion protein	1,225	P	P	P	P	P	P	P	P	1,977	P	P	P	P	P	P	P	P
1030363	-	gbs1326		Hypothetical		Hypothetical protein	0,753	P	P	P	P	P	P	P	P	1,251	P	A	P	A	P	P	A	P
1030360	COG1396K	gbs1327		Cellular processess	Transcription	Transcriptional regulator, Cro CI family	0,173	A	A	A	A	A	A	A	A	0,347	A	A	A	A	A	A	A	A
1030368	COG2017G	gbs1328		Metabolism and transport	Carbohydrates, organic alcohols, and acids	Aldose 1-epimerase family protein	0,445	P	A	A	A	P	A	A	P	0,567	P	A	M	A	A	A	A	P
1030367	-	gbs1329	lacG	Metabolism and transport	Carbohydrates, organic alcohols, and acids	6-phospho-beta-galactosidase (EC 3.2.1.85)	0,642	P	A	A	A	A	A	A	A	2,211	P	A	P	A	A	M	P	P
1030369	COG1455G	gbs1330	lacE	Metabolism and transport	Carbohydrates, organic alcohols, and acids	PTS system, lactose-specific IIBC component (EC 2.7.1.69)	0,368	P	P	P	M	M	P	P	M	2,344	P	A	P	P	A	P	P	P
1030366	-	gbs1331	lacF	Metabolism and transport	Carbohydrates, organic alcohols, and acids	PTS system, lactose-specific IIA component (EC 2.7.1.69)	0,290	A	A	P	A	A	A	M	A	2,756	P	M	P	A	A	A	P	A
1030373	COG3711K	gbs1332		Cellular processess	Transcription	Transcription antiterminator	0,349	P	A	A	A	A	A	P	A	3,539	P	A	M	P	A	P	P	A
1030371	COG3684G	gbs1333	lacD.2	Metabolism and transport	Carbohydrates, organic alcohols, and acids	Tagatose-bisphosphate aldolase (EC 4.1.2.40)	0,400	P	P	P	P	P	P	P	P	0,443	P	M	P	P	P	P	P	A
1030372	COG1105G	gbs1334	lacC.2	Metabolism and transport	Carbohydrates, organic alcohols, and acids	Tagatose-6-phosphate kinase (EC 2.7.1.144)	0,365	A	A	A	A	A	A	A	A	1,022	A	A	A	A	A	A	A	A
1030370	COG0698G	gbs1335	lacB.1	Metabolism and transport	Carbohydrates, organic alcohols, and acids	Galactose-6-phosphate isomerase lacB subunit (EC 5.3.1.26)	0,547	A	A	A	A	A	A	A	A	0,773	P	A	A	A	A	A	A	A
1030376	COG0698G	gbs1336	lacA.2	Metabolism and transport	Carbohydrates, organic alcohols, and acids	Galactose-6-phosphate isomerase lacA subunit (EC 5.3.1.26)	0,495	P	A	A	A	P	P	M	A	0,960	P	A	A	A	A	A	A	A
1030375	COG1349KG	gbs1337		Cellular processess	Transcription	Lactose phosphotransferase system repressor	2,038	P	P	P	P	P	P	P	P	1,512	P	A	P	A	M	P	P	P
1030378	COG3843U	gbs1338	srtI	General function predicted only		Relaxase	0,244	A	A	A	A	A	A	A	A	0,633	A	A	A	A	A	A	A	A
1030374	-	gbs1339		Mobile and extrachromosomal elements		Mobilisation protein	0,586	A	A	A	A	A	A	A	A	0,040	A	A	A	A	A	A	A	A
1030381	-	gbs1340		Hypothetical		Hypothetical protein	0,284	P	M	A	P	M	P	M	A	0,289	P	M	A	M	A	P	M	A
1030379	COG0210L	gbs1341		Cellular processess	DNA replication, recombination and repair	DNA helicase II (EC 3.6.1.-)	0,902	P	P	P	P	P	P	P	P	1,057	P	P	P	P	P	P	P	P
1030380	COG3593L	gbs1342		Hypothetical		Hypothetical protein	0,707	P	P	P	P	P	P	P	P	1,085	P	A	P	P	P	P	P	P
1030377	-	gbs1343		Cellular processess	Toxin production and resistance	Hypothetical protein	0,369	P	P	P	P	P	P	P	P	0,957	P	P	P	P	P	P	P	P
1030384	COG1396K	gbs1344		Cellular processess	Transcription	Transcriptional regulator, Cro CI family	0,238	P	P	P	P	P	P	P	P	0,715	P	P	P	P	P	P	P	P
1030383	-	gbs1345		Hypothetical		Hypothetical protein	0,544	A	A	M	A	A	A	A	A	0,185	A	A	A	A	A	A	A	A
1030382	-	gbs1346		Hypothetical		Hypothetical protein	0,987	A	A	A	A	A	A	A	A	0,229	A	A	A	A	A	A	A	A
1030388	-	gbs1347		Hypothetical		Hypothetical protein	0,435	A	A	A	A	A	A	A	A	0,702	A	A	A	A	A	A	A	A
1030387	-	gbs1348		General function predicted only		LtrC-like protein	0,521	P	A	A	A	A	A	A	A	0,693	P	A	A	A	A	P	A	A
1030386	-	gbs1349		Hypothetical		Hypothetical protein	0,972	P	A	P	A	A	A	A	A	0,646	P	A	A	A	A	A	A	A
1030391	-	gbs1350		Hypothetical		Hypothetical protein	0,821	P	P	P	A	P	A	P	P	0,736	P	A	P	A	A	A	A	A
1030390	-	gbs1351		Hypothetical		Hypothetical protein	0,900	A	A	A	A	A	A	A	A	1,475	A	A	A	A	A	A	A	A
1030392	)553KL, COG4	gbs1352		General function predicted only		Superfamily II DNA and RNA helicase (SNF2 family)	0,448	A	A	A	A	A	A	A	A	0,344	A	A	A	A	A	A	A	A
1030389	-	gbs1353		General function predicted only		Superfamily II DNA and RNA helicase (SNF2 family)	0,290	A	A	A	A	A	A	A	A	0,191	A	A	A	A	A	A	A	A
1030397	-	gbs1354		Hypothetical		Hypothetical protein	1,524	A	A	A	A	A	A	A	A	0,267	A	M	A	A	A	A	A	A
1030395	-	gbs1355		Hypothetical		Hypothetical protein	0,283	A	A	A	A	A	A	A	A	0,383	A	A	A	A	A	A	A	A
1030396	-	gbs1356		Cellular processess	Toxin production and resistance	LPXTG Cell surface protein	0,508	A	A	A	A	A	A	A	A	0,292	A	A	A	A	A	A	A	A
1030393	COG5340K	gbs1357		Mobile and extrachromosomal elements		Abortive infection protein AbiEi	1,928	P	P	P	P	P	P	P	P	1,145	P	P	P	P	P	P	P	P
1030401	COG2253S	gbs1358		Mobile and extrachromosomal elements		Abortive infection protein AbiEii	1,377	P	P	P	P	P	P	P	P	1,004	P	A	P	P	P	P	P	P
1030400	COG3942R	gbs1359		Hypothetical		Hypothetical membrane associated protein	0,318	A	A	A	A	A	A	A	A	0,107	A	A	A	A	A	A	A	A
1030398	COG3451U	gbs1360		Hypothetical		TRSE PROTEIN	0,160	A	A	A	A	A	A	A	A	0,250	A	A	A	A	A	A	A	A
1030399	-	gbs1361		Hypothetical		Hypothetical protein	0,424	A	A	A	A	A	A	A	A	0,191	A	A	A	A	A	A	A	A
1030405	-	gbs1362		Hypothetical		Hypothetical protein	0,287	A	A	A	A	A	A	A	A	0,149	A	A	A	A	A	A	A	A
1030403	-	gbs1363		Hypothetical		Hypothetical protein	0,182	A	A	A	P	A	A	A	A	0,130	A	A	A	A	A	P	A	A
1030406	COG3505U	gbs1364		Mobile and extrachromosomal elements		TraG TraD family	0,386	A	A	A	A	A	A	A	A	0,158	A	A	A	A	A	A	A	A
1030402	-	gbs1365		Hypothetical		Hypothetical protein	0,199	A	A	A	A	A	A	A	A	0,150	A	A	A	A	A	A	A	A
1030411	-	gbs1366		Cellular processess	Posttranslational modification, protein turnover, chaperones	CAAX amino terminal protease family	0,162	A	A	A	A	A	A	A	A	0,084	A	A	A	A	A	A	A	A

1030407	-	gbs1367	clpL	Hypothetical		Hypothetical protein	1,062	A	A	A	A	A	A	A	A	0,121	A	A	A	A	A	A	A	A
1030409	COG1393P	gbs1368		Metabolism and transport	Inorganic ion transport and metabolism	Arsenate reductase (EC 1.20.4.1)	0,081	A	A	A	A	A	A	A	A	0,015	A	A	A	A	A	A	A	A
1030408	-	gbs1369		Hypothetical		Hypothetical protein	0,500	A	A	A	A	A	M	A	A	0,066	A	A	A	A	A	A	A	A
1030414	COG0270L	gbs1370		Cellular processess	DNA replication, recombination and repair	DNA-cytosine methyltransferase (EC 2.1.1.37)	0,306	A	A	A	A	A	A	A	A	0,329	A	A	A	A	A	A	A	A
1030413	-	gbs1371		Cellular processess	DNA replication, recombination and repair	DNA-cytosine methyltransferase (EC 2.1.1.37)	0,354	A	A	A	A	A	A	A	A	0,099	A	A	A	A	A	A	A	A
1030415	-	gbs1372		Hypothetical		IFN-response binding factor 1	0,418	A	A	A	A	M	A	A	A	0,199	A	A	A	A	A	A	A	A
1030412	-	gbs1373		Hypothetical		Hypothetical protein	0,164	A	A	A	A	A	A	A	A	0,125	A	A	A	A	A	A	A	A
1030417	COG0222J	gbs1374	rplL	Cellular processess	Translation, ribosomal structure and biogenesis	LSU ribosomal protein L12P (L7 L12)	0,636	P	P	P	P	P	P	P	P	0,752	P	P	P	P	P	P	P	P
1030420	COG0244J	gbs1375	rplJ	Cellular processess	Translation, ribosomal structure and biogenesis	LSU ribosomal protein L10P	0,692	P	P	P	P	P	P	P	P	0,911	P	P	P	P	P	P	P	P
1030418	COG0542O	gbs1376	clpL	Cellular processess	Posttranslational modification, protein turnover, chaperones	ATP-dependent endopeptidase clp ATP-binding subunit clpL	1,637	P	M	P	M	P	A	A	A	1,170	P	A	P	P	P	P	P	P
1030421	COG2040E	gbs1377	mmuM	Metabolism and transport	Amino acids, peptides, aminosugars and amines	Homocysteine S-methyltransferase (EC 2.1.1.10)	2,073	P	A	A	A	A	A	A	A	1,767	P	A	A	A	A	M	A	A
1030419	COG0833E	gbs1378		Metabolism and transport	Amino acids, peptides, aminosugars and amines	S-methylmethionine permease	1,351	P	A	A	A	P	P	M	A	1,450	P	A	A	A	A	A	A	A
1030425	-	gbs1379		Hypothetical		Hypothetical protein	0,938	P	M	P	A	A	P	P	P	0,508	P	A	P	A	P	P	A	P
1030422	-	gbs1380		Hypothetical		Hypothetical protein	0,781	P	M	P	P	P	P	P	P	0,419	P	P	M	M	P	P	P	A
1030423	COG1309K	gbs1381		Cellular processess	Transcription	Transcriptional regulator, TetR family	1,555	P	A	A	A	P	A	M	A	0,518	P	A	A	A	A	A	A	A
1030428	COG0218R	gbs1382		General function predicted only		GTP-binding protein YihA	3,117	P	P	P	P	P	P	P	P	1,350	P	P	P	A	P	P	P	P
1030429	COG1219O	gbs1383	clpX	Cellular processess	Posttranslational modification, protein turnover, chaperones	ATP-dependent endopeptidase clp ATP-binding subunit clpX	2,497	P	P	P	A	P	P	M	P	0,817	P	M	P	A	A	P	A	P
1030427	COG0262H	gbs1384	dfrA	Metabolism and transport	Cofactors, prosthetic groups, and carriers	Dihydrofolate reductase (EC 1.5.1.3)	2,563	P	P	P	P	P	P	P	P	1,228	P	P	P	A	P	P	P	P
1030432	-	gbs1385	ThyA	Metabolism and transport	Purines, pyrimidines, nucleosides, and nucleotides	Thymidylate synthase (EC 2.1.1.45)	2,202	P	P	P	P	P	P	P	P	1,072	P	P	P	P	P	P	P	P
1030426	COG3425I	gbs1386	mvaS.1	Metabolism and transport	Fatty acid and phospholipids	Hydroxymethylglutaryl-CoA synthase (EC 2.3.3.10)	2,743	P	A	A	A	A	A	A	A	1,231	P	A	A	A	A	A	A	A
1030434	COG1257I	gbs1387		Metabolism and transport	Fatty acid and phospholipids	3-hydroxy-3-methylglutaryl-coenzyme A reductase (EC 1.1.1.34) 3-hydroxy-3-met	3,049	P	M	A	A	P	P	M	A	1,969	P	A	A	A	A	A	A	A
1030433	-	gbs1388		Hypothetical		Hypothetical cytosolic protein	1,754	P	P	P	P	P	P	P	P	3,007	P	A	P	A	P	P	P	P
1030436	COG1272R	gbs1389	hemIII	General function predicted only		Conserved membrane protein (hemolysin III homolog)	0,733	P	P	P	P	P	P	P	P	1,776	P	P	P	P	P	P	P	P
1030431	COG1597IR	gbs1390		Hypothetical		Hypothetical protein	1,474	P	P	P	P	P	P	P	P	1,992	P	A	P	A	P	P	P	P
1030437	COG0435O	gbs1391		Metabolism and transport	Central intermediary metabolism	Glutathione S-transferase family protein	0,636	P	P	P	P	P	P	P	P	0,695	P	P	P	P	P	P	P	P
1030435	-	gbs1392		Hypothetical		Hypothetical protein	3,299	P	A	P	A	P	P	P	P	1,302	P	A	P	A	A	P	P	A
1030443	COG1304C	gbs1393		Metabolism and transport	Energy production and conversion	Isopentenyl-diphosphate delta-isomerase (EC 5.3.3.2)	2,375	P	P	P	M	P	P	P	P	1,272	P	A	P	A	P	P	P	P
1030440	COG1577I	gbs1394	mvaK2	Metabolism and transport	Fatty acid and phospholipids	Phosphomevalonate kinase (EC 2.7.4.2)	4,631	P	P	P	A	P	P	A	M	2,091	P	A	P	A	A	P	A	A
1030441	COG3407I	gbs1395	mvaD	Metabolism and transport	Fatty acid and phospholipids	Diphosphomevalonate decarboxylase (EC 4.1.1.33)	3,023	P	A	M	A	A	A	A	A	1,395	P	A	A	A	A	A	A	A
1030439	COG1577I	gbs1396	mvaK1	Metabolism and transport	Fatty acid and phospholipids	Mevalonate kinase (EC 2.7.1.36)	2,298	P	A	P	A	P	M	A	A	1,638	P	A	M	A	A	M	A	A
1030438	COG0642T	gbs1397		Cellular processess	Signal transduction	Two component system histidine kinase (EC 2.7.3.-)	4,640	P	P	P	P	P	P	A	P	1,070	P	A	P	A	P	P	P	A
1030446	COG0745TK	gbs1398		Cellular processess	Signal transduction	Two-component response regulator	3,323	P	P	P	M	P	P	P	P	1,122	P	A	M	A	P	M	A	A
1030447	COG2357S	gbs1399		Metabolism and transport	Purines, pyrimidines, nucleosides, and nucleotides	GTP pyrophosphokinase (EC 2.7.6.5)	5,283	P	P	P	M	P	P	P	P	1,646	P	A	P	P	A	M	A	A
1030445	COG1132V	gbs1400		Cellular processess	Toxin production and resistance	Multidrug protein lipid ABC transporter family, ATP-binding and permease protein	2,000	P	A	A	A	A	A	A	A	0,751	A	A	A	A	A	A	A	A
1030448	COG1132V	gbs1401		Cellular processess	Toxin production and resistance	Multidrug resistance ABC transporter ATP-binding and permease protein	1,571	P	A	P	A	A	P	A	A	0,651	P	A	P	A	A	M	A	A
1030444	COG1846K	gbs1402		Cellular processess	Transcription	Transcriptional regulator, MarR family	1,541	P	A	A	A	A	M	A	A	0,294	P	A	A	A	A	A	A	A
1030452	COG0737F	gbs1403		Metabolism and transport	Purines, pyrimidines, nucleosides, and nucleotides	LPXTN 5 -nucleotidase (EC 3.1.3.5)	0,998	P	P	P	P	P	P	P	P	1,301	P	P	P	P	P	P	P	P
1030451	-	gbs1404	fms	Cellular processess	Posttranslational modification, protein turnover, chaperones	Peptide deformylase (EC 3.5.1.88)	1,435	P	P	P	P	P	P	P	P	1,259	P	P	P	P	P	P	P	P
1030449	COG0334E	gbs1405		Metabolism and transport	Amino acids, peptides, aminosugars and amines	NADP-specific glutamate dehydrogenase (EC 1.4.1.4)	1,019	P	P	P	P	P	P	P	P	0,557	P	P	P	P	P	P	P	P
1030456	COG3247S	gbs1406		Hypothetical		Hypothetical membrane spanning protein	1,549	P	P	P	P	P	P	P	P	1,018	P	A	P	M	P	P	P	P
1030453	COG1132V	gbs1407		Cellular processess	Toxin production and resistance	Multidrug protein lipid ABC transporter family, ATP-binding and permease protein	1,772	P	A	P	A	A	A	A	A	1,557	P	A	A	A	A	A	M	A
1030454	COG1132V	gbs1408		Cellular processess	Toxin production and resistance	Multidrug resistance ABC transporter ATP-binding and permease protein	1,595	P	A	P	P	P	P	A	A	1,141	P	A	P	A	A	P	A	A
1030461	-	gbs1409		Metabolism and transport	Central intermediary metabolism	Acetyltransferase yjgM (EC 2.3.1.-)	2,493	P	A	P	A	P	P	A	M	1,406	P	A	P	A	P	P	M	P
1030463	COG0488R	gbs1410		Transport and binding proteins	Unknown substrate	ABC transporter ATP-binding protein uup	1,898	P	P	M	A	P	P	P	P	1,381	P	A	P	P	P	P	P	M
1030458	COG0617J	gbs1411	papS	Cellular processess	Translation, ribosomal structure and biogenesis	tRNA nucleotidyltransferase (EC 2.7.7.25)	4,497	P	A	A	A	A	A	A	A	1,959	P	A	A	A	A	A	A	A
1030459	COG1307S	gbs1412		Metabolism and transport	Fatty acid and phospholipids	Fatty acid-binding protein, DegV family	2,521	P	P	P	P	P	P	P	P	1,381	P	P	P	P	P	P	P	P
1030462	-	gbs1413		Hypothetical		Hypothetical cytosolic protein	2,625	P	P	P	P	P	P	P	P	1,274	P	P	P	P	P	P	P	P
1030457	-	gbs1414		Hypothetical		Hypothetical protein	2,481	P	P	P	P	P	P	P	P	0,969	P	M	P	P	A	P	P	A
1030468	COG4331S	gbs1415		General function predicted only		CPRD14 PROTEIN	2,919	P	P	P	P	P	P	P	P	2,076	P	A	P	A	P	P	A	P
1030467	l299G, COG17	gbs1416	fruA	Metabolism and transport	Carbohydrates, organic alcohols, and acids	PTS system, fructose-specific IIABC component (EC 2.7.1.69)	0,813	P	A	P	M	P	P	A	P	4,684	P	A	A	A	A	A	A	A
1030466	COG1105G	gbs1417	fruB	Metabolism and transport	Carbohydrates, organic alcohols, and acids	1-phosphofructokinase (EC 2.7.1.56)	0,787	P	A	A	A	A	A	A	P	4,687	P	A	A	A	A	A	A	A
1030465	COG1349KG	gbs1418	fruR	Cellular processess	Transcription	Fructose repressor	1,333	P	A	A	A	A	A	A	M	7,504	P	A	A	A	A	P	A	A
1030472	COG2348V	gbs1419		Cellular processess	Toxin production and resistance	Factor essential for expression of methicillin resistance	0,670	P	P	P	P	P	P	P	P	0,545	P	P	P	P	P	P	P	P
1030471	-	gbs1420		General function predicted only		LPXTG Choline-binding protein	0,234	P	P	P	P	P	P	P	P	0,483	P	P	P	P	P	P	P	P
1030469	COG1893H	gbs1421	apbA	Metabolism and transport	Cofactors, prosthetic groups, and carriers	Ketopantoate reductase PanE ApbA family protein	1,491	P	P	P	A	P	P	P	M	0,441	P	A	P	A	P	P	A	M
1030475	COG3641R	gbs1422		Hypothetical		Hypothetical membrane spanning protein	2,491	P	A	P	A	A	A	A	A	1,369	P	A	A	A	A	A	A	A
1030473	COG0492O	gbs1423	trxB	Metabolism and transport	Energy production and conversion	Thioredoxin reductase (EC 1.8.1.9)	2,376	P	A	A	A	A	A	A	A	1,018	A	A	A	A	A	A	A	A
1030474	-	gbs1424	trmD	Cellular processess	Translation, ribosomal structure and biogenesis	tRNA (Guanine-N(1)-)-methyltransferase (EC 2.1.1.31)	2,166	P	A	A	A	A	A	A	A	0,789	A	A	A	A	A	A	A	A
1030477	COG0806J	gbs1425	rimM	Cellular processess	Translation, ribosomal structure and biogenesis	16S rRNA processing protein rimM	1,123	P	P	P	P	P	P	P	P	0,340	P	P	P	P	P	P	P	P
1030481	-	gbs1426		Cellular processess	Transcription	Transcriptional regulator	1,797	P	P	P	P	P	P	P	P	1,033	P	A	P	P	M	P	P	P
1030480	COG1837R	gbs1427		General function predicted only		RNA binding protein	1,687	P	P	P	P	P	P	P	P	0,946	P	P	P	A	P	P	P	P
1030482	-	gbs1428	rpsP	Cellular processess	Translation, ribosomal structure and biogenesis	SSU ribosomal protein S16P	1,907	P	P	P	P	P	P	P	P	1,001	P	P	P	P	P	P	P	P
1030476	COG0577V	gbs1429		Transport and binding proteins	Unknown substrate	ABC transporter permease protein	1,596	P	A	A	A	A	A	A	A	2,698	P	A	A	A	A	A	A	A
1030484	COG1136V	gbs1430		Transport and binding proteins	Unknown substrate	ABC transporter ATP-binding protein	1,419	P	A	A	A	P	A	M	A	1,273	P	A	A	A	A	A	A	A
1030486	COG0845M	gbs1431		Cell Envelope	Other	HlyD family secretion protein	1,240	P	P	P	P	P	P	P	P	0,945	P	A	P	A	P	P	A	A
1030483	COG0458EF	gbs1432		Metabolism and transport	Purines, pyrimidines, nucleosides, and nucleotides	Carbamoyl-phosphate synthase large chain (EC 6.3.5.5)	2,166	P	P	P	A	P	M	P	P	1,600	P	A	P	M	P	M	A	M
1030490	COG0505EF	gbs1433		Metabolism and transport	Purines, pyrimidines, nucleosides, and nucleotides	Carbamoyl-phosphate synthase small chain (EC 6.3.5.5)	1,549	P	P	P	P	P	P	P	P	1,114	P	A	P	P	P	P	P	P
1030487	COG2065F	gbs1434	pyrR	Metabolism and transport	Purines, pyrimidines, nucleosides, and nucleotides	Uracil phosphoribosyltransferase (EC 2.4.2.9) Pyrimidine operon regulatory prote	1,174	P	P	P	P	P	P	P	P	0,714	P	M	P	P	P	P	P	P
1030488	COG0564J	gbs1435		Cellular processess	Translation, ribosomal structure and biogenesis	Ribosomal large subunit pseudouridine synthase D (EC 4.2.1.70)	1,042	P	P	P	P	P	P	P	P	0,977	P	A	P	A	P	P	A	A
1030494	COG0597MU	gbs1436	lsp	Cellular processess	Posttranslational modification, protein turnover, chaperones	Lipoprotein signal peptidase (EC 3.4.23.36)	1,865	P	P	P	P	P	P	P	P	2,063	P	A	P	A	P	P	P	A
1030495	COG0583K	gbs1437		Cellular processess	Transcription	Transcriptional regulators, LysR family	1,732	P	P	P	P	P	P	P	P	1,837	P	A	P	A	A	A	A	A
1030493	-	gbs1438	rpmA	Cellular processess	Translation, ribosomal structure and biogenesis	LSU ribosomal protein L27P	0,927	P	P	P	P	P	P	P	P	0,835	P	P	P	P	P	P	P	P
1030499	COG2868J	gbs1439		General function predicted only		hypothetical ribosome-associated protein	0,952	P	P	P	P	P	P	P	P	0,858	P	P	P	P	P	P	P	P
1030492	-	gbs1440	rpl21	Cellular processess	Translation, ribosomal structure and biogenesis	LSU ribosomal protein L21P	0,922	P	P	P	P	P	P	P	P	0,841	P	P	P	P	P	P	P	P
1030504	COG2843M	gbs1441	capA	Cell Envelope	Biosynthesis and degradation of surface polysaccharides and lipopo	Capsule biosynthesis protein capA	0,768	P	P	P	P	P	P	P	P	1,392	P	P	P	P	A	P	P	A
1030505	COG0301H	gbs1442	thiI	Metabolism and transport	Cofactors, prosthetic groups, and carriers	Thiamine biosynthesis protein thiI	1,447	P	A	P	A	P	P	P	P	0,558	P	P	P	M	M	P	M	M
1030502	COG1104E	gbs1443	nifS1	Metabolism and transport	Amino acids, peptides, aminosugars and amines	Cysteine desulfurase (EC 2.8.1.7) Selenocysteine lyase (EC 4.4.1.16)	6,620	P	A	A	A	A	A	A	A	1,431	P	A	A	A	A	A	A	A
1030501	-	gbs1444		Hypothetical		Hypothetical protein	2,698	P	P	P	P	P	P	P	P	0,714	P	A	P	A	P	P	A	P
1030500	COG1249C	gbs1445	gor	Metabolism and transport	Energy production and conversion	Glutathione reductase (EC 1.8.1.7)	0,988	P	P	P	M	P	P	M	P	0,790	P	A	A	A	A	P	A	A
1030506	COG3679S	gbs1446		Hypothetical		Hypothetical cytosolic protein	1,517	P	P	P	P	P	P	P	P	1,325	P	A	P	P	P	P	P	M
1030507	-	gbs1447	aroF	Metabolism and transport	Amino acids, peptides, aminosugars and amines	Chorismate synthase (EC 4.2.3.5)	4,213	P	A	A	A	A	A	A	A	1,971	P	A	A	A	A	A	A	A
1030510	COG0337E	gbs1448	aroB	Metabolism and transport	Amino acids, peptides, aminosugars and amines	3-dehydroquinate synthase (EC 4.2.3.4)	9,799	P	A	A	A	A	A	A	A	3,153	P	A	A	A	A	A	A	A
1030512	-	gbs1449	aroD	Metabolism and transport	Amino acids, peptides, aminosugars and amines	3-dehydroquinate dehydratase (EC 4.2.1.10)	3,459	P	P	P	P	P	P	P	P	1,399	P	P	P	P	P	P	P	P
1030508	COG1092R	gbs1450		Metabolism and transport	Central intermediary metabolism	Methyltransferase (EC 2.1.1.-)	3,777	P	P	P	P	P	P	P	P	1,265	P	P	P	P	P	P	P	P
1030514	COG1368M	gbs1451		Metabolism and transport	Central intermediary metabolism	Sulfatase family protein	1,686	P	A	A	A	A	A	A	A	0,557	M	A	A	A	A	A	A	A
1030516	-	gbs1452	rplT	Cellular processess	Translation, ribosomal structure and biogenesis	LSU ribosomal protein L20P	1,011	P	P	P	P	P	P	P	P	1,114	P	P	P	A	P	P	P	P
1030515	COG0291J	gbs1453	rpmI	Cellular processess	Translation, ribosomal structure and biogenesis	LSU ribosomal protein L35P	0,774	P	P	P	P	P	P	P	P	1,002	P	A	A	A	A	M	A	A
1030513	COG0290J	gbs1454	infC	Cellular processess	Translation, ribosomal structure and biogenesis	Bacterial Protein Translation Initiation Factor 3 (IF-3)	0,987	P	P	P	P	P	P	P	P	1,000	P	P	P	P	P	P	P	P
1030511	COG0283F	gbs1455	cmk	Metabolism and transport	Purines, pyrimidines, nucleosides, and nucleotides	Cytidylate kinase (EC 2.7.4.14)	1,640	P	P	P	P	P	P	P	P	1,590	P	M	P	P	P	P	P	P
1030522	-	gbs1456		Hypothetical		Hypothetical membrane associated protein	1,129	P	P	P	P	P	P	P	P	1,221	P	P	P	P	P	P	P	P
1030519	COG1141C	gbs1457		Metabolism and transport	Energy production and conversion	Ferredoxin	1,440	P	P	P	P	P	P	P	P	0,785	P	P	P	P	P	P	P	A
1030518	-	gbs1458	ebsA	General function predicted only		Pore forming protein ebsA	0,821	P	P	P	P	P	P	P	P	1,326	P	A	P	P	P	P	P	P
1030517	COG2195E	gbs1459	pepT	Metabolism and transport	Amino acids, peptides, aminosugars and amines	Tripeptidase T (EC 3.4.11.4)	0,870	P	P	P	P	P	P	P	P	1,103	P	P	P	P	P	P	P	P
1030526	COG2244R	gbs1460		Cell Envelope	Biosynthesis and degradation of surface polysaccharides and lipopo	Export protein for polysaccharides and teichoic acids	1,609	P	A	P	A	P	A	M	A	0,724	P	A	A	M	A	P	A	A
1030524	COG0769M	gbs1461	murE	Cell Envelope	Biosynthesis and degradation of murein sacculus and peptidoglycan	UDP-N-acetylmuramoyl-L-alanyl-D-glutamate--lysine ligase (EC 6.3.2.7)	4,050	P	A	A	A	P	P	P	A	1,482	P	A	A	A	A	P	P	A

1030523	COG1120PH	gbs1462	fhuA	Metabolism and transport	Inorganic ion transport and metabolism	Ferrichrome transport ATP-binding protein fhuC	6,917	P	A	A	A	P	M	A	A	1,275	P	A	P	A	A	P	A	A
1030532	COG0614P	gbs1463	fhuD	Metabolism and transport	Inorganic ion transport and metabolism	Ferrichrome-binding protein	2,223	P	P	P	P	P	P	P	P	0,828	P	P	P	P	P	P	P	P
1030528	COG0609P	gbs1464	fhuB	Metabolism and transport	Inorganic ion transport and metabolism	Ferrichrome transport system permease protein fhuB	1,562	P	A	P	M	P	A	A	A	0,621	P	A	P	A	A	P	A	A
1030533	COG0609P	gbs1465	fhuG	Metabolism and transport	Inorganic ion transport and metabolism	Ferrichrome transport system permease protein fhuG	10,270	P	A	A	A	A	A	A	A	2,397	P	A	A	A	A	A	A	A
1030525	-	gbs1466		Hypothetical		Hypothetical cytosolic protein	2,104	P	P	P	P	P	P	P	P	1,274	P	A	P	P	P	P	P	P
1030534	COG1227C	gbs1467	ppaC	Metabolism and transport	Energy production and conversion	Inorganic pyrophosphatase (EC 3.6.1.1)	0,883	P	P	P	P	P	P	P	P	0,913	P	P	P	P	P	P	P	P
1030531	COG1180O	gbs1468	pflC	Metabolism and transport	Energy production and conversion	Pyruvate formate-lyase activating enzyme (EC 1.97.1.4)	0,975	P	P	P	P	P	P	P	P	1,554	P	P	P	P	P	P	P	P
1030539	COG1253R	gbs1469	hlyX	General function predicted only		Magnesium and cobalt efflux protein corC	2,062	P	P	P	P	P	P	P	P	1,071	P	P	P	P	P	P	P	P
1030537	-	gbs1470		Metabolism and transport	Central intermediary metabolism	SAM-dependent methyltransferase (EC 2.1.-.-)	1,680	P	P	P	P	P	P	P	P	1,118	P	A	P	A	P	P	P	M
1030543	COG1242R	gbs1471		General function predicted only		Radical SAM superfamily protein	1,989	P	A	P	P	P	A	P	A	2,066	P	A	P	A	A	A	M	A
1030536	COG0671I	gbs1472		Metabolism and transport	Fatty acid and phospholipids	Membrane-associated phospholipid phosphatase	0,665	P	P	P	P	P	P	P	P	1,730	P	P	P	P	P	P	P	P
1030535	COG3601S	gbs1473		Transport and binding proteins	Unknown substrate	Riboflavin transporter	0,559	P	P	P	P	P	P	P	P	1,041	P	P	P	P	P	P	P	P
1030548	COG4932M	gbs1474	pilC	hypothetical		LPXTG Hypothetical protein	1,896	P	A	P	P	P	P	P	P	2,547	P	A	P	A	A	P	A	M
1030545	COG3764M	gbs1475		Cellular processess	Posttranslational modification, protein turnover, chaperones	Sortase	3,932	P	A	P	A	A	P	A	A	3,413	P	A	P	A	A	A	A	A
1030542	COG3764M	gbs1476		Cellular processess	Posttranslational modification, protein turnover, chaperones	Sortase	2,719	P	P	P	P	P	P	P	P	1,784	P	P	P	A	P	P	M	P
1030552	-	gbs1477	pilB	Hypothetical		IPXTG Cell wall surface anchor family protein	0,639	P	P	P	P	P	P	P	P	4,661	P	A	P	A	A	P	P	A
1030551	COG4932M	gbs1478	pilA	Cell Envelope	Other	IPXTG Collagen adhesion protein	3,320	P	A	P	A	A	A	A	A	6,161	P	A	A	A	A	A	A	A
1030555	-	gbs1479	rogB	Cellular processess	Transcription	Transcriptional regulator RogB	2,564	P	P	P	A	P	M	P	P	2,312	P	A	A	A	A	A	A	A
1030549	COG0438M	gbs1480		Cell Envelope	Biosynthesis and degradation of surface polysaccharides and lipopo	N-acetylglucosaminyl-phosphatidylinositol biosynthetic protein	2,621	P	A	P	A	P	A	P	A	2,220	P	A	A	A	A	P	A	A
1030556	COG1216R	gbs1481		General function predicted only		dTDP-rhamnosyl transferase rfbF (EC 2.-.-.-)	4,220	P	A	P	M	P	P	A	M	2,589	P	A	P	A	P	P	P	P
1030557	COG2244R	gbs1482		General function predicted only		Oligosaccharide translocase (flippase)	3,438	P	A	A	A	A	A	A	A	1,687	P	P	A	A	A	M	A	A
1030553	-	gbs1483		Hypothetical		Hypothetical protein	3,286	P	P	P	P	P	P	A	P	1,281	P	P	P	A	P	P	P	P
1030563	COG0463M	gbs1484		Metabolism and transport	Central intermediary metabolism	Glycosyltransferase involved in cell wall biogenesis (EC 2.4.-.-)	2,584	P	P	P	P	P	P	P	P	1,566	P	P	P	P	P	P	P	P
1030560	COG0463M	gbs1485		Metabolism and transport	Central intermediary metabolism	Glycosyltransferase involved in cell wall biogenesis (EC 2.4.-.-)	2,474	P	P	P	P	P	P	P	P	1,187	P	P	P	P	P	P	P	P
1030566	COG0451MG	gbs1486		Hypothetical		UDP-D-glucuronate carboxy-lyase (EC 4.1.1.35)	2,314	P	P	P	P	P	P	P	P	1,313	P	P	P	P	P	P	P	P
1030559	COG1211I	gbs1487		Metabolism and transport	Fatty acid and phospholipids	2-C-methyl-D-erythritol 4-phosphate cytidylyltransferase (EC 2.7.7.60)	1,656	P	P	P	A	P	P	P	P	1,262	P	A	P	A	P	P	A	P
1030567	COG3475M	gbs1488		Cell Envelope	Biosynthesis and degradation of surface polysaccharides and lipopo	Lipooligosaccharide cholinephosphotransferase (EC 2.7.8.-)	1,866	P	P	P	P	P	P	P	P	0,748	P	P	P	P	P	P	P	P
1030565	-	gbs1489		Hypothetical		Hypothetical protein	4,095	P	A	A	A	A	A	A	A	1,862	P	A	A	A	A	A	A	A
1030564	COG2456S	gbs1490		Hypothetical		Hypothetical membrane spanning protein	4,094	P	P	P	P	P	P	P	P	1,682	P	P	P	P	P	P	P	P
1030570	COG0463M	gbs1491		Metabolism and transport	Central intermediary metabolism	Glycosyltransferase involved in cell wall biogenesis (EC 2.4.-.-)	2,703	P	A	P	A	A	P	A	A	0,967	P	A	A	A	A	M	M	A
1030568	COG0463M	gbs1492	rgpBc	Cell Envelope	Biosynthesis and degradation of murein sacculus and peptidoglycan	alpha-L-Rha alpha-1,3-L-rhamnosyltransferase (EC 2.4.1.-)	2,834	P	A	A	A	A	A	A	A	1,800	P	A	A	A	A	A	A	A
1030571	COG0438M	gbs1493	rgpAc	Cell Envelope	Biosynthesis and degradation of murein sacculus and peptidoglycan	alpha-D-GlcNAc alpha-1,2-L-rhamnosyltransferase (EC 2.4.1.-)	3,018	P	A	P	A	A	A	A	A	1,258	P	A	A	A	A	A	A	A
1030575	COG1091M	gbs1494	rmlD	Metabolism and transport	Carbohydrates, organic alcohols, and acids	dTDP-4-dehydrorhamnose reductase (EC 1.1.1.133)	1,175	P	P	P	A	P	P	P	P	1,085	P	A	A	A	A	A	A	A
1030577	-	gbs1495		Hypothetical		Hypothetical cytosolic protein	1,577	P	P	P	P	P	P	P	P	0,943	P	P	P	M	M	P	P	A
1030574	COG0568K	gbs1496	rpoD	Cellular processess	Transcription	RNA polymerase sigma factor rpoD	2,372	P	P	P	P	P	P	P	P	1,227	P	A	P	A	P	P	P	A
1030576	COG0358L	gbs1497	dnaG	Cellular processess	DNA replication, recombination and repair	DNA primase (EC 2.7.7.-)	3,439	P	A	A	A	A	M	A	A	1,070	P	A	P	A	P	P	A	A
1030572	-	gbs1498	mscL	Transport and binding proteins	Unknown substrate	Large-conductance mechanosensitive channel	0,439	P	P	P	P	P	P	P	P	0,548	P	P	P	P	P	P	P	P
1030581	COG0828J	gbs1499	rpsU	Cellular processess	Translation, ribosomal structure and biogenesis	SSU ribosomal protein S21P	0,568	P	P	P	P	P	P	P	P	0,608	P	P	P	P	P	P	P	P
1030579	COG0727R	gbs1500		Hypothetical		Hypothetical protein	1,128	P	A	P	A	P	A	A	A	0,690	A	A	A	A	A	A	A	A
1030580	COG0834ET	gbs1501		Metabolism and transport	Amino acids, peptides, aminosugars and amines	Arginine-binding protein	0,674	P	P	P	M	P	P	M	P	0,325	P	A	P	A	A	M	P	A
1030578	-	gbs1502		Metabolism and transport	Amino acids, peptides, aminosugars and amines	Ammonium transporter	0,455	P	P	P	P	P	P	M	P	0,191	P	A	P	M	A	A	A	P
1030584	COG0492O	gbs1503		Cellular processess	Posttranslational modification, protein turnover, chaperones	Oxidoreductase (EC 1.1.1.-)	1,149	A	A	A	A	A	A	A	A	0,400	A	A	A	A	A	A	A	A
1030583	COG1054R	gbs1504		Metabolism and transport	Central intermediary metabolism	Rhodanese-related sulfurtransferases	1,231	P	P	P	A	P	P	P	P	0,815	P	P	P	A	M	P	A	A
1030582	-	gbs1505		Hypothetical		Hypothetical protein	0,771	P	P	P	P	P	P	P	P	0,608	P	A	P	A	P	P	P	P
1030586	COG2271G	gbs1506		Metabolism and transport	Carbohydrates, organic alcohols, and acids	Transporter	0,969	A	A	A	A	A	A	A	A	1,795	P	A	A	A	A	A	A	A
1030592	COG0058G	gbs1507	glgP	Metabolism and transport	Carbohydrates, organic alcohols, and acids	Maltodextrin phosphorylase (EC 2.4.1.1)	0,636	P	P	P	P	P	P	P	P	3,406	P	P	P	P	P	P	P	P
1030595	-	gbs1508	malM	Metabolism and transport	Carbohydrates, organic alcohols, and acids	4-alpha-glucanotransferase (EC 2.4.1.25)	0,353	P	P	P	P	P	P	P	P	2,730	P	A	P	M	A	P	P	P
1030593	COG1609K	gbs1509	malR	Cellular processess	Transcription	Transcriptional regulator, LacI family	2,693	P	P	P	A	P	P	P	P	1,434	P	A	A	A	A	P	A	A
1030597	COG2182G	gbs1510	malE	Metabolism and transport	Carbohydrates, organic alcohols, and acids	Maltose maltodextrin-binding protein	0,242	P	P	P	P	P	P	P	P	0,967	P	P	P	P	P	P	P	P
1030591	COG1175G	gbs1511	malF	Metabolism and transport	Carbohydrates, organic alcohols, and acids	Maltodextrin transport system permease protein malC	0,482	P	M	P	P	P	P	P	P	1,757	P	P	P	P	P	P	P	P
1030599	COG3833G	gbs1512	malG	Metabolism and transport	Carbohydrates, organic alcohols, and acids	Maltose transport system permease protein malG	0,663	P	P	P	P	P	P	P	P	2,179	P	P	P	P	P	P	P	P
1030600	COG3104E	gbs1513		Metabolism and transport	Amino acids, peptides, aminosugars and amines	Di- tripeptide transporter	2,751	P	M	P	P	M	P	A	A	1,047	P	A	P	A	A	P	A	A
1030598	COG0494LR	gbs1514		Cellular processess	DNA replication, recombination and repair	Phosphohydrolase (MutT nudix family protein)	4,039	P	A	A	A	A	A	A	A	2,023	P	A	A	A	A	A	A	A
1030603	-	gbs1515		Hypothetical		Hypothetical protein	4,143	P	A	A	A	A	A	A	A	2,247	P	A	A	A	A	M	A	P
1030602	-	gbs1516		Hypothetical		surface protein Pls	1,840	P	M	P	P	P	P	A	P	1,212	P	A	P	A	P	P	A	P
1030607	COG0438M	gbs1517		Metabolism and transport	Central intermediary metabolism	Probable poly(Glycerol-phosphate) alpha-glucosyltransferase (EC 2.4.1.52)	2,859	P	A	A	A	A	A	A	A	2,594	P	A	A	A	A	A	A	A
1030610	COG0653U	gbs1518		Cellular processess	Posttranslational modification, protein turnover, chaperones	Protein translocase subunit secA	2,282	P	A	A	A	A	A	A	A	0,944	P	A	P	A	M	A	A	A
1030609	-	gbs1519		Hypothetical		Hypothetical protein	3,148	P	A	A	A	A	A	A	A	1,751	P	A	P	A	A	P	A	A
1030608	-	gbs1520		Hypothetical		Hypothetical protein	2,016	P	P	A	A	P	M	P	A	1,048	P	M	P	M	P	P	A	P
1030605	-	gbs1521		Hypothetical		Hypothetical protein	19,176	P	A	A	A	A	A	A	A	2,181	P	A	A	A	A	A	A	A
1030604	COG0201U	gbs1522		Cellular processess	Posttranslational modification, protein turnover, chaperones	Protein translocase subunit secY	2,476	P	M	P	A	M	P	A	A	1,676	P	A	P	P	P	P	A	P
1030612	-	gbs1523		Hypothetical		Hypothetical protein	1,952	P	A	P	P	M	P	A	A	1,119	P	P	P	P	P	P	M	P
1030613	COG0463M	gbs1524		Metabolism and transport	Central intermediary metabolism	Glycosyltransferase (EC 2.4.1.-)	2,747	P	A	A	A	A	A	A	A	1,840	P	A	P	A	A	A	A	A
1030614	COG1442M	gbs1525		Metabolism and transport	Central intermediary metabolism	Glycosyl transferase, family 8	5,160	P	A	A	A	A	A	A	A	1,701	P	A	A	A	A	A	A	A
1030611	COG1442M	gbs1526		Metabolism and transport	Central intermediary metabolism	Glycosyl transferase, family 8	6,786	M	A	A	A	A	A	A	A	2,074	P	A	A	A	A	A	A	A
1030617	COG1442M	gbs1527		Metabolism and transport	Central intermediary metabolism	Glycosyl transferase, family 8	1,499	P	M	P	A	A	A	A	A	0,732	P	A	M	A	A	A	A	A
1030616	-	gbs1528		Metabolism and transport	Carbohydrates, organic alcohols, and acids	Putative galactofuranosyltransferase (EC 2.4.1.-)	1,998	P	A	A	A	A	A	A	A	2,950	P	A	A	A	A	A	A	A
1030619	-	gbs1529		Cell Envelope	Other	LPXTG Fibronectin-binding protein	1,279	P	A	A	A	P	A	A	A	0,341	P	A	A	A	M	P	P	A
1030615	-	gbs1530	rofA	Cellular processess	Transcription	Transcriptional regulator RogB	2,722	P	A	P	A	P	P	P	A	1,062	P	A	P	A	A	P	A	A
1030621	COG0556L	gbs1531	UvrB	Cellular processess	DNA replication, recombination and repair	Excinuclease ABC subunit B	1,213	P	A	P	A	P	P	A	A	0,868	P	A	A	A	A	P	A	A
1030620	-	gbs1532		Cellular processess	Posttranslational modification, protein turnover, chaperones	CAAX amino terminal protease family	2,636	P	P	P	P	P	P	P	P	1,049	P	A	P	A	M	P	P	M
1030618	0765E, COG08	gbs1533	glnP	Metabolism and transport	Amino acids, peptides, aminosugars and amines	Glutamine-binding protein Glutamine transport system permease protein glnP	13,329	P	A	A	A	A	A	A	A	8,781	M	A	A	A	A	A	A	A
1030623	COG1126E	gbs1534	glnQ	Metabolism and transport	Amino acids, peptides, aminosugars and amines	Glutamine transport ATP-binding protein glnQ	7,859	P	P	P	P	P	P	P	P	5,053	P	A	P	A	A	P	A	A
1030622	-	gbs1535		Hypothetical		Hypothetical protein	4,036	P	P	P	P	P	P	P	P	1,920	P	M	P	P	P	P	P	P
1030625	-	gbs1536		Hypothetical		Hypothetical cytosolic protein	0,979	P	P	P	P	P	P	P	P	1,166	P	A	A	A	P	P	M	M
1030627	COG0536R	gbs1537	obg	General function predicted only		GTP-binding protein CgtA (probably involved in DNA repair)	1,568	P	P	P	P	P	P	P	P	1,217	P	P	P	P	P	P	P	P
1030626	COG2309E	gbs1538		Metabolism and transport	Amino acids, peptides, aminosugars and amines	Aminopeptidase S (EC 3.4.11.-)	0,760	P	P	P	P	P	P	P	P	0,930	P	A	P	M	M	P	P	A
1030624	-	gbs1539		Hypothetical		LPXTG Hypothetical protein	2,007	P	P	P	P	P	P	P	P	0,744	P	P	P	P	P	P	P	P
1030630	-	gbs1540		Metabolism and transport	Carbohydrates, organic alcohols, and acids	LPXTG Amidase family protein	1,056	P	P	P	P	P	P	P	P	0,914	P	P	P	P	P	P	P	P
1030632	COG1187J	gbs1541	rsuA	Cellular processess	Translation, ribosomal structure and biogenesis	Ribosomal small subunit pseudouridine synthase A (EC 4.2.1.70)	0,654	P	P	P	P	P	P	P	P	0,531	P	A	P	A	P	P	A	M
1030628	COG0656R	gbs1542		General function predicted only		Aldo keto reductase family	0,285	P	P	P	P	P	P	P	P	0,567	P	P	P	P	P	P	P	P
1030633	COG0778C	gbs1543		Metabolism and transport	Energy production and conversion	NAD(P)H-dependent quinone reductase (EC 1.-.-.-)	0,688	P	P	P	P	P	P	P	P	0,715	P	A	P	P	P	P	A	P
1030634	COG0346E	gbs1544	gloA	Metabolism and transport	Amino acids, peptides, aminosugars and amines	Lactoylglutathione lyase (EC 4.4.1.5)	0,593	P	P	P	P	P	P	P	P	0,658	P	P	P	P	P	P	P	P
1030631	COG0463M	gbs1545		Metabolism and transport	Central intermediary metabolism	Bactoprenol glucosyl transferase (EC 2.4.1.-)	1,618	P	A	A	A	A	A	A	A	2,785	P	A	A	A	A	A	A	A
1030636	COG1113E	gbs1546		Metabolism and transport	Amino acids, peptides, aminosugars and amines	Amino acid permease	1,785	P	P	P	P	P	P	P	P	0,543	P	P	P	P	P	P	P	P
1030639	COG0691O	gbs1547		Cellular processess	Posttranslational modification, protein turnover, chaperones	SsrA-binding protein	3,226	P	M	P	A	M	P	A	A	1,866	P	A	A	A	A	A	A	A
1030635	COG0557K	gbs1548		Cellular processess	DNA replication, recombination and repair	Exoribonuclease II (EC 3.1.13.1)	1,451	P	P	P	P	P	P	P	P	0,660	P	A	P	P	P	P	A	P
1030640	COG1314U	gbs1549	secG	Cellular processess	Posttranslational modification, protein turnover, chaperones	Protein translocase subunit secG	2,790	P	P	P	P	P	P	P	P	1,254	P	P	P	P	P	P	P	P
1030641	-	gbs1550		Cellular processess	Toxin production and resistance	Multidrug resistance efflux pump	2,150	P	P	P	P	M	P	A	A	0,861	P	A	A	A	A	M	A	A
1030637	-	gbs1551		Hypothetical		Hypothetical protein	1,106	P	A	A	A	A	A	A	A	0,749	P	A	A	A	A	A	A	A
1030638	COG1131V	gbs1552		Transport and binding proteins	Unknown substrate	ABC transporter ATP-binding protein	1,055	P	A	A	A	M	A	P	A	1,501	A	A	A	A	A	A	A	A
1030643	COG0237H	gbs1553	coaE	Metabolism and transport	Cofactors, prosthetic groups, and carriers	Dephospho-CoA kinase (EC 2.7.1.24)	2,029	P	A	P	A	A	A	A	A	0,606	P	A	A	A	A	A	A	A
1030644	COG0266L	gbs1554	fpg	Cellular processess	DNA replication, recombination and repair	Formamidopyrimidine-DNA glycosylase (EC 3.2.2.23)	1,607	P	A	A	A	P	A	A	P	1,009	P	A	P	A	M	A	A	A
1030646	-	gbs1555	mutR	Cellular processess	Transcription	Transcriptional regulator	2,456	P	A	A	A	A	A	A	A	1,149	P	A	A	A	A	M	P	A
1030642	COG5279D	gbs1556		Cell Envelope	Biosynthesis and degradation of murein sacculus and peptidoglycan	S-layer homology domain Putative murein endopeptidase	0,413	P	P	P	P	P	P	P	P	1,333	P	P	P	P	P	P	P	P

1030650	-	gbs1558		Cellular processess	Posttranslational modification, protein turnover, chaperones	CAAX amino terminal protease family	2,155	P	A	A	A	P	A	A	A	0,421	P	A	A	A	A	A	A	A
1030648	-	gbs1559		Hypothetical		Hypothetical protein	1,645	P	P	P	P	P	P	P	P	1,317	P	A	P	A	P	P	P	A
1030645	COG1159R	gbs1560	era	General function predicted only		GTP-binding protein era	1,627	P	P	P	P	P	P	A	P	1,201	P	A	P	A	A	A	A	A
1030652	-	gbs1561	dgk	Metabolism and transport	Fatty acid and phospholipids	Diacylglycerol kinase (EC 2.7.1.107)	1,852	P	M	P	P	P	P	P	P	1,669	P	P	P	P	P	P	P	A
1030653	COG0319R	gbs1562		Hypothetical		Hypothetical metal-binding protein	1,291	P	P	P	P	P	P	P	P	1,369	P	P	P	P	P	P	P	P
1030651	COG3315Q	gbs1563		Metabolism and transport	Secondary metabolites	Polyketide synthase O-methyltransferase (EC 2.1.1.-)	1,235	P	A	P	A	A	P	A	P	2,658	P	A	A	A	A	A	A	A
1030655	COG0494LR	gbs1564		Cellular processess	DNA replication, recombination and repair	Phosphohydrolase (MutT nudix family protein)	6,964	P	A	A	A	A	A	A	A	2,300	P	A	A	A	A	A	A	A
1030649	-	gbs1565		Hypothetical		Hypothetical protein	3,218	P	A	A	A	A	A	A	A	1,903	A	A	A	A	A	A	A	A
1030656	COG1702T	gbs1566	phoH	Cellular processess	Signal transduction	PhoH protein	2,472	P	A	A	A	A	M	A	M	0,780	P	A	A	A	A	A	P	A
1030654	COG4716S	gbs1567		General function predicted only		Myosin-crossreactive antigen	1,816	P	A	P	A	P	M	A	A	1,672	P	A	M	A	A	A	A	A
1030661	-	gbs1568		Hypothetical		Hypothetical cytosolic protein	1,669	P	P	P	P	P	P	P	P	2,211	P	P	P	A	P	P	P	P
1030660	-	gbs1569	msrA.2	Cellular processess	Posttranslational modification, protein turnover, chaperones	Peptide methionine sulfoxide reductase msrA (EC 1.8.4.6)	1,283	P	P	P	P	P	P	P	P	1,045	P	A	P	A	P	P	A	M
1030658	COG2996S	gbs1570		General function predicted only		S1 RNA binding domain	1,569	P	P	P	P	P	P	P	P	1,221	P	P	P	P	P	P	P	P
1030674	-	gbs1571	frr	Cellular processess	Translation, ribosomal structure and biogenesis	Ribosome Recycling Factor (RRF)	1,149	P	P	P	P	P	P	P	P	1,409	P	P	P	P	P	P	P	P
1031936	COG0528F	gbs1572	pyrH	Metabolism and transport	Purines, pyrimidines, nucleosides, and nucleotides	Uridylate kinase (EC 2.7.4.-)	1,736	P	P	P	P	P	P	P	P	1,629	P	P	P	P	P	P	P	P
1031802	COG1124EP	gbs1573		Metabolism and transport	Amino acids, peptides, aminosugars and amines	Nickel transport ATP-binding protein nikE	2,541	P	P	P	P	A	A	A	A	1,624	P	M	P	A	A	P	A	P
1030662	COG0444EP	gbs1574		Metabolism and transport	Amino acids, peptides, aminosugars and amines	Nickel transport ATP-binding protein nikD	3,474	P	A	A	A	A	A	M	A	2,022	P	A	P	A	A	P	A	A
1031803	COG1173EP	gbs1575		Metabolism and transport	Amino acids, peptides, aminosugars and amines	Nickel transport system permease protein nikC	17,601	P	A	A	A	A	A	A	A	3,760	P	A	A	A	A	A	A	A
1031800	COG0601EP	gbs1576		Metabolism and transport	Amino acids, peptides, aminosugars and amines	Nickel transport system permease protein nikB	6,485	P	A	A	A	A	A	A	A	2,254	P	A	A	A	A	A	A	A
1031804	COG0747E	gbs1577		Metabolism and transport	Amino acids, peptides, aminosugars and amines	Nickel-binding protein	3,748	P	A	A	P	A	A	A	A	1,082	P	A	P	A	A	P	A	A
1031398	-	578 (gbsp	rplA	Cellular processess	Translation, ribosomal structure and biogenesis	LSU ribosomal protein L1P	1,819	P	P	P	P	P	P	P	P	0,649	P	P	P	P	P	P	P	P
1031801	-	579 (gbsp	rplK	Cellular processess	Translation, ribosomal structure and biogenesis	LSU ribosomal protein L11P	2,501	P	P	P	P	P	P	P	P	0,564	P	P	P	P	P	P	P	P
1031806	-	gbs1580		Hypothetical		Hypothetical protein	1,493	P	A	P	A	A	A	A	A	0,875	P	A	A	A	A	A	A	A
1031805	COG1473R	gbs1581		General function predicted only		Carboxypeptidase, M20(D) family	1,527	P	A	A	A	A	A	A	A	1,089	A	A	A	A	A	A	A	A
1031811	COG0583K	gbs1582		Cellular processess	Transcription	Transcriptional regulators, LysR family	2,448	P	A	A	P	P	A	P	A	0,690	P	A	A	A	A	A	A	A
1031808	-	gbs1583		Hypothetical		Integral membrane protein	1,745	P	P	P	P	P	P	P	P	0,850	P	A	P	P	P	P	P	P
1031809	115EH, COG0	gbs1584		Metabolism and transport	Amino acids, peptides, aminosugars and amines	Para-aminobenzoate synthetase component I (EC 6.3.5.8) 4-amino-4-deoxychori	1,113	P	A	A	A	A	A	P	M	1,248	P	A	A	A	A	A	A	A
1031807	COG1674D	gbs1585	ftsK	Cellular processess	Cell division	Cell division protein ftsK	2,440	P	P	P	P	P	P	P	P	1,033	P	A	P	M	P	P	A	P
1031813	COG0652O	gbs1586		Cellular processess	Posttranslational modification, protein turnover, chaperones	Peptidyl-prolyl cis-trans isomerase (EC 5.2.1.8)	1,491	P	P	P	P	P	P	P	P	0,962	P	P	P	P	A	P	P	P
1031812	COG1108P	gbs1587	mtsC	Metabolism and transport	Inorganic ion transport and metabolism	Manganese transport system membrane protein	6,407	P	A	P	P	P	P	P	P	1,673	P	P	P	P	P	P	P	P
1031810	COG1121P	gbs1588	mtsB	Metabolism and transport	Inorganic ion transport and metabolism	Manganese transport system ATP-binding protein	3,498	P	A	P	P	P	P	P	P	1,782	P	M	P	M	P	P	P	P
1031817	COG0803P	gbs1589	mtsA	Metabolism and transport	Inorganic ion transport and metabolism	Manganese-binding protein	0,914	P	P	P	P	P	P	P	P	1,523	P	P	P	P	P	P	P	P
1031815	COG1321K	gbs1590	scaR	Cellular processess	Transcription	Iron-dependent repressor	0,760	P	P	P	P	P	P	P	P	1,003	P	P	P	P	P	P	P	P
1031814	COG0775F	gbs1591	pfs	Metabolism and transport	Purines, pyrimidines, nucleosides, and nucleotides	5 -methylthioadenosine nucleosidase (EC 3.2.2.16) S-adenosylhomocysteine nuc	1,096	P	A	P	A	P	A	P	A	1,861	P	A	M	A	A	A	A	A
1031818	-	gbs1592		Hypothetical		Hypothetical membrane associated protein	1,291	P	P	P	P	P	P	P	P	1,784	P	A	P	A	P	P	M	A
1031816	COG0494LR	gbs1593		Metabolism and transport	Purines, pyrimidines, nucleosides, and nucleotides	ADP-ribose pyrophosphatase (EC 3.6.1.13)	0,977	P	P	P	P	P	P	P	P	2,141	P	A	P	P	M	P	M	A
1031822	COG1207M	gbs1594	gcaD	Metabolism and transport	Amino acids, peptides, aminosugars and amines	Glucosamine-1-phosphate acetyltransferase (EC 2.3.1.-) UDP-N-acetylglucosami	0,877	P	P	P	P	P	P	A	P	1,125	P	A	M	A	A	M	M	A
1031821	COG2764S	gbs1595		Metabolism and transport	Energy production and conversion	PhnB protein	0,447	P	P	P	P	P	P	P	P	0,538	P	P	P	P	P	P	A	P
1031819	COG0346E	gbs1596		Metabolism and transport	Amino acids, peptides, aminosugars and amines	Lactoylglutathione lyase (EC 4.4.1.5)	0,615	P	P	P	P	P	P	P	P	0,820	P	A	P	M	A	A	A	A
1031820	COG0673R	gbs1597		Metabolism and transport	Central intermediary metabolism	NAD-dependent oxidoreductase	1,307	P	P	P	P	P	P	P	P	1,147	P	A	P	P	P	P	P	P
1031823	-	gbs1598		Hypothetical		Hypothetical protein	1,526	P	P	P	P	P	P	P	P	2,455	P	A	P	P	A	P	A	P
1031823	-	gbs1598		Hypothetical		Hypothetical protein	1,244	P	P	P	P	P	P	P	P	1,411	P	A	P	M	M	P	A	A
1031837	-	gbs1599		Hypothetical		Hypothetical protein	1,235	P	P	P	P	P	P	P	P	1,470	P	A	P	A	A	P	A	A
1031834	COG1028IQR	gbs1600	fabG	Metabolism and transport	Fatty acid and phospholipids	Short chain dehydrogenase	1,145	P	A	P	A	P	P	A	A	1,528	P	A	A	A	A	A	P	A
1031833	-	gbs1601		Hypothetical		Hypothetical protein	0,646	P	P	P	P	P	P	P	P	1,195	P	P	P	M	M	P	P	A
1031830	-	gbs1602		General function predicted only		DNA-binding protein	1,429	P	P	P	P	P	P	P	P	1,157	P	A	P	A	A	P	A	A
1031824	-	gbs1603		Hypothetical		Hypothetical protein	1,137	P	P	P	P	P	P	P	P	1,156	P	A	P	P	A	P	A	A
1031831	COG1215M	gbs1605		Metabolism and transport	Central intermediary metabolism	Glycosyltransferase (EC 2.4.1.-)	0,906	P	A	P	P	P	P	P	A	1,970	P	A	M	A	A	M	P	A
1031839	-	gbs1606		Hypothetical		Hypothetical protein	3,745	P	A	A	A	A	A	A	A	3,069	P	A	A	A	A	A	A	A
1031836	-	gbs1607		Hypothetical		Hypothetical protein	1,228	P	A	A	A	A	A	A	A	1,426	P	A	A	A	A	A	A	A
1031835	COG1285S	gbs1608		Hypothetical		Hypothetical protein	1,023	P	A	A	A	A	A	A	A	0,481	P	A	A	A	A	A	A	A
1031844	-	gbs1609		Hypothetical		Hypothetical protein	1,690	P	A	A	A	A	A	A	A	1,141	P	A	A	A	A	A	A	A
1031843	COG1114E	gbs1610		Metabolism and transport	Amino acids, peptides, aminosugars and amines	Branched-chain amino acid transport system carrier protein	1,355	P	P	P	M	P	P	P	P	0,934	P	A	A	A	A	P	A	A
1031842	0073R, COG0	gbs1611	metG	Cellular processess	Translation, ribosomal structure and biogenesis	Methionyl-tRNA synthetase (EC 6.1.1.10) Protein secretion chaperonin CsaA	1,405	P	P	P	P	P	P	P	P	1,349	P	A	P	A	P	P	M	P
1031845	COG3615P	gbs1612		Metabolism and transport	Inorganic ion transport and metabolism	Tellurite resistance protein tehB	0,754	P	P	P	P	P	P	P	P	0,954	P	A	P	P	P	P	P	P
1031841	-	gbs1613		Hypothetical		Hypothetical membrane spanning protein	2,306	P	A	A	A	A	A	A	A	0,839	P	A	A	A	A	A	P	A
1031846	COG1455G	gbs1614		Metabolism and transport	Carbohydrates, organic alcohols, and acids	PTS system, cellobiose-specific IIC component	1,603	P	A	A	A	P	M	P	A	2,044	P	A	A	A	A	A	A	A
1031840	COG4814R	gbs1615		General function predicted only		putative acyltransferases and hydrolases with the alpha beta hydrolase fold	2,394	P	P	P	P	P	P	P	P	2,052	P	A	P	P	P	P	P	P
1031848	COG0708L	gbs1616	exoA	Cellular processess	DNA replication, recombination and repair	Exodeoxyribonuclease III (EC 3.1.11.2)	1,902	P	P	P	P	P	P	P	P	1,305	P	A	P	P	P	P	P	P
1031847	COG1393P	gbs1617		Metabolism and transport	Inorganic ion transport and metabolism	Arsenate reductase family protein	0,739	P	P	P	P	P	P	P	P	0,980	P	A	P	A	P	P	P	A
1031851	COG0350L	gbs1618		Metabolism and transport	Energy production and conversion	O6-methylguanine-DNA methyltransferase (EC 2.1.1.63)	1,439	P	P	P	P	P	P	P	P	0,836	P	A	A	A	A	P	P	A
1031849	COG0111HE	gbs1619		Metabolism and transport	Carbohydrates, organic alcohols, and acids	D-3-phosphoglycerate dehydrogenase (EC 1.1.1.95)	3,249	P	P	P	P	P	P	P	P	1,102	P	A	M	A	M	P	A	A
1031850	-	gbs1620		Metabolism and transport	Central intermediary metabolism	Acetyltransferase (EC 2.3.1.-)	3,494	P	P	P	P	P	P	P	P	1,739	P	A	M	A	A	A	A	A
1031852	COG1932HE	gbs1621	serC	Metabolism and transport	Amino acids, peptides, aminosugars and amines	Phosphoserine aminotransferase (EC 2.6.1.52)	1,942	P	P	P	P	P	P	P	P	0,458	P	P	P	A	P	P	P	P
1031856	COG3142P	gbs1622	cutC	Metabolism and transport	Inorganic ion transport and metabolism	Copper homeostasis protein cutC	1,017	P	P	P	P	P	P	P	P	0,605	P	P	P	P	P	P	A	M
1031854	COG0313R	gbs1623		General function predicted only		Tetrapyrrole (Corrin Porphyrin) methylase family protein	1,981	P	A	P	A	A	A	A	P	1,775	P	A	A	A	A	A	A	A
1031853	COG4467S	gbs1624		Hypothetical		Initiation-control protein	1,720	P	P	P	P	P	P	P	P	1,787	P	A	P	P	P	P	P	P
1031855	COG0470L	gbs1625	dnaX	Cellular processess	DNA replication, recombination and repair	DNA polymerase III, delta subunit (EC 2.7.7.7)	1,549	P	P	P	P	P	P	P	P	1,373	P	P	P	P	P	P	P	P
1031858	COG0125F	gbs1626	tmk	Metabolism and transport	Purines, pyrimidines, nucleosides, and nucleotides	Thymidylate kinase (EC 2.7.4.9)	0,947	P	P	P	P	P	P	P	P	1,165	P	A	P	P	P	P	P	A
1031863	COG0517R	gbs1627		Metabolism and transport	Energy production and conversion	Acetoin utilization acuB protein	0,504	P	P	P	P	P	P	P	P	1,871	P	A	P	A	A	P	A	P
1031860	COG0410E	gbs1628		Metabolism and transport	Amino acids, peptides, aminosugars and amines	Branched-chain amino acid transport ATP-binding protein livF	0,526	P	M	P	A	M	P	A	P	2,779	P	A	A	A	A	A	A	A
1031861	COG0411E	gbs1629		Metabolism and transport	Amino acids, peptides, aminosugars and amines	Branched-chain amino acid transport ATP-binding protein livG	0,637	P	P	P	P	P	P	P	P	2,323	P	A	P	A	A	P	M	P
1031864	COG4177E	gbs1630		Metabolism and transport	Amino acids, peptides, aminosugars and amines	Branched-chain amino acid transport system permease protein livM	0,588	P	P	P	P	P	P	P	P	1,874	P	A	P	A	A	P	P	P
1031862	COG0559E	gbs1631		Metabolism and transport	Amino acids, peptides, aminosugars and amines	Branched-chain amino acid transport system permease protein livH	0,646	P	P	P	P	P	P	P	P	1,014	P	P	P	M	P	P	P	P
1031859	COG0683E	gbs1632		Metabolism and transport	Amino acids, peptides, aminosugars and amines	Leucine-, isoleucine-, valine-, threonine-, and alanine-binding protein	0,731	P	P	P	P	P	P	P	P	0,897	P	A	P	M	P	P	P	P
1031857	-	gbs1633		Hypothetical		Hypothetical cytosolic protein	2,871	P	P	P	P	P	P	P	P	1,338	P	A	P	A	P	P	P	P
1031870	COG0740OU	gbs1634	clpP	Cellular processess	Posttranslational modification, protein turnover, chaperones	ATP-dependent endopeptidase clp proteolytic subunit clpP (EC 3.4.21.92)	0,966	P	P	P	P	P	P	P	P	0,689	P	P	P	P	P	P	P	P
1031867	COG0035F	gbs1635	upp	Metabolism and transport	Purines, pyrimidines, nucleosides, and nucleotides	Uracil phosphoribosyltransferase (EC 2.4.2.9)	0,807	P	P	P	P	P	P	P	P	0,936	P	P	P	P	P	P	P	P
1031865	COG1168E	gbs1636		Metabolism and transport	Amino acids, peptides, aminosugars and amines	Cystathionine beta-lyase (EC 4.4.1.8)	1,156	P	M	P	A	P	P	P	P	0,655	P	A	A	A	A	P	A	A
1031866	COG0219J	gbs1637		Cellular processess	Translation, ribosomal structure and biogenesis	23S rRNA methyltransferase (EC 2.1.1.-)	2,604	P	A	A	A	A	P	A	P	1,268	A	A	A	A	A	A	A	A
1031868	COG0531E	gbs1638		Metabolism and transport	Amino acids, peptides, aminosugars and amines	Amino acid permease	2,458	P	M	A	A	P	P	M	P	0,785	P	A	A	A	A	P	P	A
1031872	COG0569P	gbs1639		Metabolism and transport	Inorganic ion transport and metabolism	Trk system potassium uptake protein trkA	2,072	P	A	A	A	P	P	M	A	0,634	P	M	P	A	A	A	A	A
1031871	COG0168P	gbs1640		Metabolism and transport	Inorganic ion transport and metabolism	Trk system potassium uptake protein trkH	4,138	P	P	P	A	P	P	P	A	1,526	P	A	A	A	A	A	P	A
1031874	-	gbs1641		Hypothetical		Hypothetical cytosolic protein	2,220	P	P	P	P	P	P	P	P	1,401	P	P	P	P	P	P	P	P
1031876	COG1187J	gbs1642		Cellular processess	Translation, ribosomal structure and biogenesis	Ribosomal large subunit pseudouridine synthase B (EC 4.2.1.70)	3,019	P	A	P	P	A	A	A	A	1,137	P	A	P	A	P	P	A	A
1031869	-	gbs1643	scpB	Cellular processess	Cell division	Segregation and condensation protein ScpB	2,408	P	A	A	A	A	P	A	A	1,111	P	A	P	A	A	P	A	M
1031877	COG1354S	gbs1644	scpA	Cellular processess	Cell division	Segregation and condensation protein ScpA	6,185	P	A	P	A	A	M	P	A	2,146	P	A	P	A	P	P	M	P
1031878	-	gbs1645		Mobile and extrachromosomal elements		DNA integration recombination inversion protein	4,129	P	P	P	A	P	P	M	P	1,209	P	P	P	P	P	P	P	P
1031875	COG0517R	gbs1646		General function predicted only		CBS domain containing protein	3,822	P	A	M	A	P	A	A	A	1,682	P	A	P	A	P	P	P	A
1031882	COG0622R	gbs1647		General function predicted only		putative phosphoesterase	3,313	P	P	P	P	P	P	P	P	1,632	P	P	P	P	P	P	P	P
1031879	COG0127F	gbs1648		Metabolism and transport	Purines, pyrimidines, nucleosides, and nucleotides	Xanthosine triphosphate pyrophosphatase (EC 3.6.1.-)	2,223	P	P	P	P	P	P	P	P	1,406	P	P	P	P	P	P	P	P
1031880	COG0796M	gbs1649	glr	Cell Envelope	Biosynthesis and degradation of murein sacculus and peptidoglycan	Glutamate racemase (EC 5.1.1.3)	3,279	P	P	P	A	P	P	M	P	1,527	P	A	P	A	A	P	A	A
1031873	COG3763S	gbs1650		Hypothetical		Hypothetical exported protein	2,314	P	P	P	P	P	P	P	P	1,283	P	P	P	P	P	P	P	P
1031883	-	gbs1651		Hypothetical		Hypothetical protein	2,288	P	P	P	P	P	P	P	P	0,422	P	A	P	A	A	A	A	A
1031884	-	gbs1652		Cellular processess	Transcription	Transcriptional regulator, biotin repressor family	2,469	P	P	P	P	P	P	P	P	2,336	P	A	P	A	A	M	P	A

1031881	COG0670R	gbs1653		Hypothetical		Integral membrane protein	1,157	P	P	P	P	P	P	P	P	1,170	P	P	P	P	P	P	P	P
1031889	COG1418R	gbs1654		Metabolism and transport	Central intermediary metabolism	Hydrolase (HAD superfamily)	1,091	P	P	P	P	P	P	P	P	1,051	P	P	P	P	P	P	P	P
1031886	COG0566J	gbs1655		Cellular processess	Translation, ribosomal structure and biogenesis	23S rRNA methyltransferase (EC 2.1.1.-)	1,992	P	P	P	M	P	P	P	P	1,717	P	M	P	P	P	P	P	P
1031887	COG1254C	gbs1656		Metabolism and transport	Energy production and conversion	Acylphosphatase (EC 3.6.1.7)	0,744	P	P	P	P	P	P	P	P	1,016	P	A	A	A	P	A	A	A
1031885	COG0706U	gbs1657		Cell Envelope	Other	60 kDa inner membrane protein YIDC	1,372	P	P	P	P	P	P	P	P	0,574	P	P	P	P	P	P	P	P
1031891	COG0765E	gbs1658		Metabolism and transport	Amino acids, peptides, aminosugars and amines	Probable amino-acid ABC transporter permease protein yckA	1,035	P	P	P	P	P	P	P	P	1,339	P	P	P	P	P	P	P	P
1030732	COG0834ET	gbs1659		Metabolism and transport	Amino acids, peptides, aminosugars and amines	Cystine-binding protein	0,890	P	P	P	P	P	P	P	P	0,993	P	P	P	P	P	P	P	P
1029816	-	gbs1660	amiC	Metabolism and transport	Amino acids, peptides, aminosugars and amines	Amidase family protein	1,406	P	A	P	A	P	A	A	A	1,473	P	A	A	A	A	A	A	A
1031179	COG0782K	gbs1661	greA	Cellular processess	Transcription	Transcription elongation factor greA	2,221	P	P	P	P	P	P	P	P	0,745	P	A	P	A	A	P	A	A
1031229	-	gbs1662		Hypothetical		Hypothetical membrane associated protein	2,075	P	P	P	P	P	P	P	P	0,843	P	P	P	A	P	P	P	P
1031232	-	gbs1663		Metabolism and transport	Central intermediary metabolism	Acetyltransferase, GNAT family	4,019	P	A	A	A	A	A	A	A	2,018	A	A	A	A	A	A	A	A
1030666	COG0773M	gbs1664	murC	Cell Envelope	Biosynthesis and degradation of murein sacculus and peptidoglycan	UDP-N-acetylmuramate--alanine ligase (EC 6.3.2.8)	2,326	P	P	P	P	P	P	P	P	0,707	P	P	P	P	P	P	P	P
1030737	-	gbs1665		Hypothetical		Hypothetical cytosolic protein	2,498	P	P	P	P	P	P	P	P	0,892	P	P	P	P	P	P	P	P
1031231	COG0553KL	gbs1666	snf	Hypothetical		SWF SNF family helicase	2,750	P	A	M	A	A	P	M	A	3,354	P	A	A	A	A	A	A	A
1031116	COG1160R	gbs1667	pgdA	General function predicted only		GTP-binding protein	1,780	P	P	P	A	P	P	P	P	1,216	P	A	P	M	P	P	P	P
1030812	COG1484L	gbs1668	dnaI	Cellular processess	DNA replication, recombination and repair	Primosomal protein dnaI	1,451	P	A	A	A	P	A	A	A	1,024	P	A	A	A	A	A	A	A
1030665	COG3611L	gbs1669	dnaB	Cellular processess	DNA replication, recombination and repair	Replicative DNA helicase (EC 3.6.1.-)	2,358	P	P	P	P	P	P	P	P	1,327	P	A	P	P	P	P	P	P
1030833	-	gbs1670		Cellular processess	Transcription	Putative regulatory protein	1,659	P	P	P	P	P	P	P	P	1,035	P	P	P	P	P	P	P	P
1030601	COG0642T	gbs1671	covS	Cellular processess	Signal transduction	Transmembrane histidine kinase CsrS	1,548	P	A	P	A	P	A	P	P	0,852	P	A	P	A	A	P	P	P
1031230	COG0745TK	gbs1672	covR	Cellular processess	Signal transduction	Response regulator CsrR	1,452	P	P	P	P	P	P	P	P	0,996	P	P	P	P	P	P	P	P
1031227	COG1399R	gbs1673		Hypothetical		Hypothetical cytosolic protein	1,635	P	P	P	P	P	P	P	P	0,795	P	P	P	P	P	P	P	P
1030606	COG0501O	gbs1674		Cellular processess	Posttranslational modification, protein turnover, chaperones	Endopeptidase htpX (EC 3.4.24.-)	0,621	P	P	P	P	P	P	P	P	1,841	P	A	P	P	P	P	P	P
1030706	-	gbs1675	lemA	General function predicted only		LemA protein	0,342	P	P	P	P	P	P	P	P	0,684	P	P	P	P	P	P	P	P
1030734	COG0357M	gbs1676	gidB	Metabolism and transport	Central intermediary metabolism	Methyltransferase gidB (EC 2.1.-.-)	3,170	P	P	P	M	P	P	P	P	1,201	P	A	P	A	A	P	P	P
1030714	COG0168P	gbs1677		Metabolism and transport	Inorganic ion transport and metabolism	Potassium uptake protein ktrB	1,972	P	M	P	A	P	A	A	A	1,323	P	A	M	A	A	A	A	A
1031228	COG0569P	gbs1678		Metabolism and transport	Inorganic ion transport and metabolism	Potassium uptake protein ktrA	1,317	P	P	P	M	P	P	P	P	0,997	P	A	P	A	A	P	A	A
1030689	COG0619P	gbs1679		Metabolism and transport	Inorganic ion transport and metabolism	Cobalt transport protein cbiQ	2,853	P	P	P	A	P	P	M	A	1,707	P	A	A	A	A	P	M	A
1030735	COG1122P	gbs1680		Metabolism and transport	Inorganic ion transport and metabolism	Cobalt transport ATP-binding protein cbiO	6,705	P	A	A	A	A	A	A	A	3,167	P	A	A	A	A	A	P	A
1030541	COG4720S	gbs1681		Hypothetical		Hypothetical protein	2,074	P	A	P	A	P	P	A	A	1,236	P	A	A	A	A	P	P	A
1031530	COG3633E	gbs1682		Metabolism and transport	Amino acids, peptides, aminosugars and amines	Serine threonine sodium symporter	4,728	P	A	P	A	P	M	A	A	1,682	P	A	A	A	A	A	A	A
1030673	COG1114E	gbs1683	braB	Metabolism and transport	Amino acids, peptides, aminosugars and amines	Branched-chain amino acid transport system carrier protein	1,951	P	P	A	A	P	P	P	P	0,715	P	A	P	A	A	P	M	P
1031574	COG1063ER	gbs1684		Metabolism and transport	Carbohydrates, organic alcohols, and acids	Zn-dependent alcohol dehydrogenases and related dehydrogenases	0,134	P	P	P	P	P	P	P	P	1,375	P	P	P	P	P	P	P	P
1031563	COG2011P	gbs1685		Transport and binding proteins	Unknown substrate	ABC transporter permease protein	1,598	P	P	P	P	P	P	P	P	2,253	P	A	A	A	P	P	A	A
1031562	COG1135P	gbs1686		Transport and binding proteins	Unknown substrate	ABC transporter ATP-binding protein	2,182	P	M	P	A	P	P	A	P	2,054	P	A	A	A	A	A	A	A
1031570	COG0624E	gbs1687		Metabolism and transport	Amino acids, peptides, aminosugars and amines	Acetylornithine deacetylase Succinyl-diaminopimelate desuccinylase and related d	3,033	P	A	A	A	A	A	A	A	5,056	P	A	A	A	A	A	A	A
1031051	COG1464P	gbs1688		Transport and binding proteins	Unknown substrate	ABC transporter substrate-binding protein	2,297	P	P	P	P	P	P	P	P	3,171	P	A	P	P	P	P	P	P
1030774	COG0834ET	gbs1689		Transport and binding proteins	Unknown substrate	ABC transporter substrate-binding protein	0,658	P	P	P	A	P	P	P	M	0,379	M	A	A	A	A	P	A	P
1031549	COG2071R	gbs1690		General function predicted only		Glutamine amidotransferase, class I	1,183	P	M	P	A	P	A	A	A	0,436	A	A	A	A	A	A	A	A
1031325	-	gbs1691		Hypothetical		Hypothetical cytosolic protein	1,322	P	P	P	P	P	P	P	P	0,991	P	P	P	P	P	P	P	P
1031277	COG2376G	gbs1692		Metabolism and transport	Carbohydrates, organic alcohols, and acids	Dihydroxyacetone kinase (EC 2.7.1.29)	0,855	P	A	A	A	A	A	A	A	1,314	P	A	A	A	A	A	A	A
1031198	COG1309K	gbs1693		Cellular processess	Transcription	Transcriptional regulator, TetR family	1,162	P	A	A	P	P	A	A	P	2,834	P	A	A	A	A	A	A	A
1030356	COG2376G	gbs1694		Metabolism and transport	Carbohydrates, organic alcohols, and acids	Dihydroxyacetone kinase (EC 2.7.1.29)	0,838	A	A	A	A	A	A	A	A	0,774	P	A	A	A	A	A	A	A
1030362	COG2376G	gbs1695		Metabolism and transport	Carbohydrates, organic alcohols, and acids	Dihydroxyacetone kinase (EC 2.7.1.29)	0,846	P	A	P	P	P	P	A	M	0,939	P	P	P	P	P	P	P	P
1030340	COG3412S	gbs1696		Metabolism and transport	Fatty acid and phospholipids	Dihydroxyacetone kinase phosphotransfer protein	2,664	P	A	A	A	A	A	A	A	1,245	P	A	A	A	A	A	A	A
1030843	-	gbs1697		Metabolism and transport	Carbohydrates, organic alcohols, and acids	Glycerol uptake facilitator protein	0,907	P	A	M	P	P	A	A	A	0,564	P	A	P	P	P	P	P	P
1029835	COG0662G	gbs1698		Hypothetical		Hypothetical cytosolic protein	0,417	P	P	P	P	P	P	P	P	0,782	P	P	P	P	P	P	P	P
1030365	COG0789K	gbs1699		Cellular processess	Transcription	Transcriptional activator tipA	2,297	P	A	A	A	A	A	A	P	1,572	P	A	A	A	A	A	A	A
1031422	COG1323R	gbs1700		Hypothetical		Hypothetical cytosolic protein	2,455	P	A	A	A	A	A	A	A	0,982	A	A	A	A	A	A	A	A
1030792	-	gbs1701		Hypothetical		Hypothetical protein	3,523	P	A	P	P	A	P	A	A	1,670	P	A	P	A	A	P	A	A
1031378	COG0500QR	gbs1702		Metabolism and transport	Secondary metabolites	Methyltransferase (EC 2.1.1.-)	3,754	P	A	A	A	A	A	A	A	2,161	A	A	A	A	A	A	A	A
1031376	-	gbs1703		Hypothetical		iojap protein family	2,220	P	P	P	P	P	P	P	P	0,955	P	M	P	P	P	P	P	P
1030344	COG1335Q	gbs1704		Metabolism and transport	Secondary metabolites	Isochorismatase family protein	2,584	P	A	P	M	P	P	P	P	1,030	P	P	P	M	P	P	P	P
1030352	COG1713H	gbs1705		Metabolism and transport	Cofactors, prosthetic groups, and carriers	Hydrolase (HAD superfamily)	3,206	P	M	A	A	A	A	A	A	1,321	P	A	M	A	A	M	A	A
1031292	COG1057H	gbs1706	nadD	Metabolism and transport	Cofactors, prosthetic groups, and carriers	Nicotinate-nucleotide adenylyltransferase (EC 2.7.7.18)	3,017	P	P	P	P	P	P	P	P	1,437	P	P	P	P	P	P	P	P
1030078	-	gbs1707		Hypothetical		hypothetical RNA binding protein	3,710	P	P	A	M	A	A	A	A	1,910	P	A	P	A	A	P	A	M
1031389	COG1161R	gbs1708		General function predicted only		GTP-binding protein	2,046	P	P	P	P	P	P	P	P	0,806	P	P	P	P	P	P	P	P
1031668	COG2179R	gbs1709		General function predicted only		Putative lipase	5,137	P	A	A	A	A	A	A	A	2,121	P	A	A	A	A	A	A	A
1031355	COG0697GER	gbs1710		Transport and binding proteins	Unknown substrate	Transporter, drug metabolite exporter family	1,719	P	A	A	A	A	A	A	A	0,739	M	A	A	A	A	A	A	A
1030455	COG0064J	gbs1711	gatB	Cellular processess	Translation, ribosomal structure and biogenesis	Aspartyl glutamyl-tRNA(Asn Gln) amidotransferase subunit B (EC 6.3.5.-)	1,409	P	P	P	M	P	P	P	P	0,970	P	A	P	P	P	P	P	P
1030183	-	gbs1712	gatA	Cellular processess	Translation, ribosomal structure and biogenesis	Aspartyl glutamyl-tRNA(Asn Gln) amidotransferase subunit A (EC 6.3.5.-)	3,447	P	P	P	A	P	P	P	P	1,729	P	A	P	A	M	P	P	P
1029862	COG0721J	gbs1713	gatC	Cellular processess	Translation, ribosomal structure and biogenesis	Aspartyl glutamyl-tRNA(Asn Gln) amidotransferase subunit C (EC 6.3.5.-)	2,880	P	P	P	A	P	P	P	P	1,426	P	M	P	M	P	P	P	P
1030268	COG0574G	gbs1714		Metabolism and transport	Carbohydrates, organic alcohols, and acids	Pyruvate,phosphate dikinase (EC 2.7.9.1)	1,419	M	A	A	A	A	A	A	A	0,109	A	A	P	A	P	P	A	A
1031461	-	gbs1715		General function predicted only		ATP GTP-binding protein	1,409	P	A	A	A	A	A	A	A	0,084	A	M	P	A	P	P	A	A
1031400	COG0517R	gbs1716		General function predicted only		CBS domain containing protein	1,315	P	P	P	P	P	A	A	A	0,126	P	P	P	P	P	P	P	P
1030863	COG1250I	gbs1717		Metabolism and transport	Fatty acid and phospholipids	3-hydroxybutyryl-CoA dehydrogenase (EC 1.1.1.157)	0,822	P	P	P	P	P	P	P	P	0,300	P	P	P	P	P	P	P	P
1030063	COG1335Q	gbs1718		Metabolism and transport	Secondary metabolites	Pyrazinamidase (EC 3.5.1.-) Nicotinamidase (EC 3.5.1.19)	2,768	P	P	P	M	P	P	P	P	1,974	P	A	P	A	A	P	A	A
1031455	-	gbs1719	codY	Cellular processess	Transcription	Transcription pleiotropic repressor codY	2,811	P	A	P	A	A	A	A	A	1,122	P	A	M	A	A	A	A	A
1030746	COG0436E	gbs1720		Metabolism and transport	Amino acids, peptides, aminosugars and amines	Aspartate aminotransferase (EC 2.6.1.1)	1,696	P	P	P	P	P	P	P	P	0,987	P	P	P	P	P	P	P	P
1030629	COG0589T	gbs1721		Cellular processess	Posttranslational modification, protein turnover, chaperones	Universal stress protein family	0,171	P	P	P	P	P	P	P	P	0,341	P	P	P	P	P	P	P	P
1031541	‹0561R, COG4	gbs1722		Metabolism and transport	Central intermediary metabolism	Hydrolase (HAD superfamily)	1,756	P	A	A	A	A	A	A	A	0,476	P	A	A	A	A	A	A	A
1030529	-	gbs1723	asnB	Metabolism and transport	Amino acids, peptides, aminosugars and amines	L-asparaginase (EC 3.5.1.1)	1,506	P	A	M	A	A	A	A	A	1,569	P	A	A	A	A	A	A	A
1030023	COG0169E	gbs1724	aroE	Metabolism and transport	Amino acids, peptides, aminosugars and amines	Shikimate 5-dehydrogenase (EC 1.1.1.25)	3,427	P	A	P	A	P	A	M	M	1,558	P	A	A	A	A	A	A	A
1030846	COG4989R	gbs1725		Metabolism and transport	Central intermediary metabolism	Oxidoreductase (EC 1.1.1.-)	2,498	P	P	P	P	P	P	P	P	1,191	P	A	M	A	A	A	A	M
1030858	COG1200LK	gbs1726	recG	Cellular processess	DNA replication, recombination and repair	ATP-dependent DNA helicase recG (EC 3.6.1.-)	1,674	P	P	P	M	P	P	P	P	0,908	P	A	P	M	A	P	A	A
1030170	COG3942R	gbs1727	isp2	General function predicted only		Immunogenic secreted protein	1,959	P	P	P	P	P	P	P	P	0,909	P	P	P	P	P	P	P	P
1029940	COG0787M	gbs1728	alr	Cell Envelope	Biosynthesis and degradation of murein sacculus and peptidoglycan	Alanine racemase (EC 5.1.1.1)	2,482	P	A	A	A	A	A	A	A	2,983	P	A	A	A	A	A	A	A
1030345	COG0736I	gbs1729	acpS	Metabolism and transport	Fatty acid and phospholipids	Holo-[acyl-carrier protein] synthase (EC 2.7.8.7)	2,219	P	A	A	A	A	A	A	A	1,823	P	A	A	A	A	A	A	A
1031536	COG0722E	gbs1730		Metabolism and transport	Amino acids, peptides, aminosugars and amines	3-deoxy-7-phosphoheptulonate synthase (EC 2.5.1.54)	2,804	P	A	A	A	A	A	A	A	1,643	P	A	A	A	A	A	A	A
1030346	COG0653U	gbs1731	secA	Cellular processess	Posttranslational modification, protein turnover, chaperones	Protein translocase subunit secA	1,616	P	P	P	P	P	P	P	P	1,297	P	P	P	P	P	P	P	P
1030410	COG1482G	gbs1732	pmi	Metabolism and transport	Carbohydrates, organic alcohols, and acids	Mannose-6-phosphate isomerase (EC 5.3.1.8)	1,397	P	A	P	A	P	P	P	A	1,224	P	A	P	A	A	A	A	A
1030394	COG1940KG	gbs1733	scrK	Metabolism and transport	Carbohydrates, organic alcohols, and acids	Fructokinase (EC 2.7.1.4)	0,888	A	A	A	A	A	A	A	A	0,676	A	A	A	A	A	A	A	A
1030249	COG1263G	gbs1734	scrA	Metabolism and transport	Carbohydrates, organic alcohols, and acids	PTS system, sucrose-specific IIABC component (EC 2.7.1.69)	1,256	P	A	A	A	A	A	A	A	2,070	P	A	A	A	A	A	A	A
1030678	-	gbs1735	scrB	Metabolism and transport	Carbohydrates, organic alcohols, and acids	Sucrose-6-phosphate hydrolase (EC 3.2.1.26)	2,506	P	A	A	A	A	A	A	A	2,142	P	M	A	A	A	A	A	A
1030460	COG1609K	gbs1736	scrR	Cellular processess	Transcription	Sucrose operon repressor	1,960	P	A	A	A	A	A	A	A	1,929	P	A	A	A	A	A	A	A
1030497	COG0781K	gbs1737	nusB	Cellular processess	Transcription	N utilization substance protein B	1,242	P	P	P	P	P	P	P	P	1,527	P	P	P	P	P	P	P	P
1030404	-	gbs1738		Cellular processess	Posttranslational modification, protein turnover, chaperones	General stress protein, Gls24 family	0,872	P	P	P	P	P	P	P	P	1,424	P	P	P	P	P	P	P	P
1030682	COG0231J	gbs1739	efp	Cellular processess	Translation, ribosomal structure and biogenesis	Protein Translation Elongation Factor P (EF-P)	0,987	P	P	P	P	P	P	P	P	1,158	P	P	P	P	P	P	P	P
1030540	COG1132V	gbs1740		Transport and binding proteins	Unknown substrate	ABC transporter ATP-binding and permease protein	4,239	A	A	A	A	A	A	A	A	1,202	P	A	A	A	A	A	A	A
1030681	COG1132V	gbs1741		Transport and binding proteins	Unknown substrate	ABC transporter, ATP-binding/permease protein	1,576	P	A	A	M	P	P	P	P	0,874	P	A	P	P	P	P	P	P
1030676	COG1122P	gbs1742		Metabolism and transport	Inorganic ion transport and metabolism	Cobalt transport ATP-binding protein cbiO	5,395	P	A	A	A	A	A	A	A	1,250	P	A	P	P	M	A	P	A
1030686	COG0619P	gbs1743		Metabolism and transport	Inorganic ion transport and metabolism	Cobalt permease and related transporters	4,019	P	A	A	A	A	A	A	A	1,301	P	A	P	M	P	M	P	P
1030679	-	gbs1744		Hypothetical		membrane-bound protein	7,498	P	A	A	A	A	A	A	A	1,603	P	A	P	A	A	P	A	P
1030680	-	gbs1745		Hypothetical		Hypothetical protein	3,774	P	A	A	A	A	A	A	A	1,235	P	A	P	M	P	P	A	A
1030677	-	gbs1746		Hypothetical		Hypothetical protein	6,524	P	P	P	A	P	P	P	P	1,500	P	P	P	P	P	P	P	P
1030684	COG1021Q	gbs1748		Metabolism and transport	Secondary metabolites	2,3-dihydroxybenzoate-AMP ligase (EC 2.7.7.58)	2,614	P	A	A	P	M	P	A	A	1,135	P	P	P	P	P	P	P	P

1030683	COG1321K	gbs1749		Cellular processess	Transcription	Iron-dependent repressor	3,031	P	A	P	A	A	P	A	P	3,089	P	A	A	A	A	A	A	A
1030685	COG2131F	gbs1750	comEB	Metabolism and transport	Purines, pyrimidines, nucleosides, and nucleotides	ComE operon protein 2	1,911	P	M	P	A	P	P	P	A	3,418	P	A	A	A	A	A	A	A
1030715	COG0006E	gbs1751		Metabolism and transport	Amino acids, peptides, aminosugars and amines	Xaa-Pro dipeptidase (EC 3.4.13.9)	1,331	P	P	P	P	P	P	P	P	1,992	P	A	P	A	A	M	A	A
1030708	-	gbs1752		Hypothetical		Hypothetical protein	1,603	P	A	A	A	A	A	A	A	1,044	P	A	A	A	A	A	A	A
1030687	-	gbs1753		Cellular processess	Toxin production and resistance	Multidrug resistance protein B	1,505	P	A	A	A	A	A	A	A	0,583	P	A	A	A	A	A	A	A
1030705	COG0178L	gbs1754	uvrA	Cellular processess	DNA replication, recombination and repair	Excinuclease ABC subunit A	1,540	P	A	A	A	A	A	A	A	1,045	P	A	A	A	A	A	A	A
1030719	-	gbs1755		Hypothetical		Hypothetical membrane spanning protein	1,712	P	P	P	A	M	P	A	A	0,568	M	A	P	A	A	P	A	A
1030723	COG0598P	gbs1756	corA	Metabolism and transport	Inorganic ion transport and metabolism	Magnesium and cobalt transport protein corA	2,008	P	P	P	M	P	P	P	P	0,568	P	A	P	A	A	P	A	A
1030704	-	gbs1757	rpsR	Cellular processess	Translation, ribosomal structure and biogenesis	SSU ribosomal protein S18P	0,715	P	P	P	P	P	P	P	P	0,378	P	P	P	P	P	P	P	P
1030713	COG0629L	gbs1758	ssb3	Cellular processess	DNA replication, recombination and repair	Single strand binding protein	1,012	P	P	P	P	P	P	P	P	0,424	P	P	P	P	P	P	P	P
1030730	COG0360J	gbs1759	rpsF	Cellular processess	Translation, ribosomal structure and biogenesis	SSU ribosomal protein S6P	0,797	P	P	P	P	P	P	P	P	0,396	P	P	P	P	P	P	P	P
1030720	COG1194L	gbs1760	mutY	Cellular processess	DNA replication, recombination and repair	A G-specific adenine DNA glycosylase (EC 3.2.2.-)	1,505	P	P	P	A	P	P	P	P	0,922	P	A	P	A	A	P	M	A
1030726	COG1396K	gbs1761		Cellular processess	Transcription	Transcriptional regulator	0,795	P	M	P	P	P	P	P	P	0,841	P	A	A	A	A	A	A	A
1030744	COG0526OC	gbs1762	trx	Metabolism and transport	Energy production and conversion	Thioredoxin	1,273	P	P	P	P	P	P	P	P	1,033	P	P	P	P	P	P	P	P
1031017	-	gbs1763		Metabolism and transport	Fatty acid and phospholipids	Phosphatidylglycerophosphatase B (EC 3.1.3.27)	2,334	P	P	P	P	P	P	P	P	1,332	P	P	P	P	P	P	P	P
1030736	COG1193L	gbs1764	mutS2	Cellular processess	DNA replication, recombination and repair	DNA mismatch repair protein mutS	2,578	P	A	A	A	A	A	A	A	1,849	P	A	A	A	A	A	A	A
1031828	-	gbs1765		General function predicted only		CvpA family membrane protein	1,542	P	P	P	P	P	P	P	P	1,035	P	A	P	P	P	P	P	A
1030758	-	gbs1766		Hypothetical		Hypothetical cytosolic protein	2,718	P	P	P	P	P	P	P	P	2,041	P	A	P	P	P	P	P	P
1031367	COG1039L	gbs1767		Cellular processess	Transcription	Ribonuclease HIII (EC 3.1.26.4)	5,060	P	A	P	A	P	P	P	P	1,381	P	A	P	A	P	P	P	A
1030794	COG0681U	gbs1768	spi	Cellular processess	Posttranslational modification, protein turnover, chaperones	Signal peptidase I (EC 3.4.21.89)	1,330	P	P	P	P	P	P	P	P	0,712	P	A	P	A	P	P	P	P
1030802	COG0507L	gbs1769	recD	Cellular processess	DNA replication, recombination and repair	Exodeoxyribonuclease V alpha chain (EC 3.1.11.5)	0,910	P	A	A	A	A	A	A	A	0,622	A	A	A	A	A	A	A	A
1030764	-	gbs1770		Hypothetical		Hypothetical protein	0,505	P	P	P	P	P	P	P	P	1,297	P	P	P	P	P	P	P	P
1031309	COG0389L	gbs1771	dinP	Cellular processess	DNA replication, recombination and repair	DNA polymerase IV (EC 2.7.7.7)	1,125	P	P	P	P	P	P	P	P	0,760	P	A	P	P	P	P	M	P
1030768	COG1882C	gbs1772	pfl	Metabolism and transport	Energy production and conversion	Formate acetyltransferase (EC 2.3.1.54)	0,368	P	P	P	P	P	P	P	P	0,533	P	P	P	P	P	P	P	P
1030831	-	gbs1773		General function predicted only		FMN-binding protein	0,590	P	P	P	P	P	P	P	P	0,871	P	P	P	P	P	P	P	P
1030769	COG1680V	gbs1774		Cellular processess	Toxin production and resistance	Beta-lactamase family protein	2,309	P	P	P	A	P	P	A	P	0,631	P	A	A	A	A	M	A	A
1030770	-	gbs1775		Hypothetical		Hypothetical cytosolic protein	1,464	P	P	P	M	P	P	P	P	0,525	P	A	P	A	P	P	A	A
1030773	-	gbs1776		Hypothetical		Hypothetical membrane spanning protein	2,502	P	P	P	A	P	P	P	P	0,523	P	A	P	A	A	P	P	A
1030776	-	gbs1777	glpF.2	Metabolism and transport	Carbohydrates, organic alcohols, and acids	Aquaporin Glycerol uptake facilitator protein	1,812	P	P	P	P	P	P	P	P	0,884	P	P	P	P	P	P	P	A
1030777	COG0589T	gbs1778		Cellular processess	Posttranslational modification, protein turnover, chaperones	Universal stress protein family	2,426	A	A	A	A	A	A	A	A	0,512	A	A	A	A	A	A	A	A
1031468	-	gbs1779	norA	Transport and binding proteins	Unknown substrate	Transporter, MFS superfamily	0,907	P	A	A	A	A	M	A	A	0,734	A	A	A	A	A	A	A	A
1030778	COG0664T	gbs1780	srv	Cellular processess	Transcription	Transcription regulator, crp family	1,266	P	P	P	P	P	P	P	P	1,716	P	A	P	A	M	P	A	P
1030789	COG2936R	gbs1781	pepXP	Metabolism and transport	Amino acids, peptides, aminosugars and amines	Xaa-Pro dipeptidyl-peptidase (EC 3.4.14.11)	1,434	P	P	P	P	P	P	P	P	1,015	P	A	P	P	P	P	P	P
1031410	-	gbs1782		Hypothetical		Hypothetical protein	3,450	P	P	P	A	P	P	P	P	2,986	P	A	P	A	P	P	A	P
1030790	COG0142H	gbs1783		Metabolism and transport	Cofactors, prosthetic groups, and carriers	Farnesyl pyrophosphate synthetase (EC 2.5.1.1) Geranyltranstransferase (EC 2.5	2,004	P	P	P	P	P	P	P	P	1,154	P	A	P	A	M	P	P	M
1030803	COG4987CO	gbs1784		Metabolism and transport	Energy production and conversion	Transport ATP-binding protein cydC	13,095	A	A	A	A	A	A	A	A	2,010	A	A	A	A	A	A	A	A
1030795	COG4988CO	gbs1785		Metabolism and transport	Energy production and conversion	Transport ATP-binding protein cydD	2,182	P	A	A	A	A	A	A	A	1,770	P	A	A	A	A	A	A	A
1030801	COG1294C	gbs1786		Metabolism and transport	Energy production and conversion	Cytochrome d ubiquinol oxidase subunit II (EC 1.10.3.-)	1,912	P	P	P	P	P	P	P	P	1,220	P	P	P	P	P	P	P	P
1030701	COG1271C	gbs1787		Metabolism and transport	Energy production and conversion	Cytochrome d ubiquinol oxidase subunit I (EC 1.10.3.-)	1,837	P	P	P	P	P	P	P	P	1,011	P	P	P	P	P	P	P	P
1030807	COG1252C	gbs1788		Metabolism and transport	Energy production and conversion	NADH dehydrogenase family	1,446	P	A	P	A	P	P	A	P	0,838	P	A	P	A	A	P	P	P
1030761	COG1575H	gbs1789		Metabolism and transport	Cofactors, prosthetic groups, and carriers	1,4-dihydroxy-2-naphthoate polyprenyltransferase (EC 2.5.1.-)	1,823	P	A	A	A	A	A	A	A	0,972	P	A	M	A	A	A	A	A
1030847	-	gbs1790		Hypothetical		Hypothetical protein	0,637	P	P	P	P	P	P	P	P	0,782	P	P	P	P	P	P	P	P
1030848	COG0718S	gbs1791		Hypothetical		Hypothetical cytosolic protein	1,815	P	P	P	P	P	P	P	P	0,865	P	A	P	P	P	P	P	P
1030850	COG2230M	gbs1792		Cell Envelope	Biosynthesis and degradation of surface polysaccharides and lipopo	Cyclopropane-fatty-acyl-phospholipid synthase (EC 2.1.1.79)	1,467	P	P	P	P	P	P	P	P	0,448	P	A	A	A	A	A	A	A
1030851	COG0789K	gbs1793		Cellular processess	Transcription	Transcriptional regulator, MerR family	0,681	P	P	P	P	P	P	P	P	0,929	P	A	P	P	A	P	P	P
1030859	COG0847L	gbs1794	dnaQ	Cellular processess	DNA replication, recombination and repair	DNA polymerase III, epsilon chain (EC 2.7.7.7)	1,583	P	P	P	P	P	P	P	P	1,451	P	A	A	A	A	A	A	A
1030852	-	gbs1795		Hypothetical		Hypothetical cytosolic protein	2,967	P	P	P	P	P	P	P	P	1,207	P	P	P	P	P	P	P	P
1030856	COG2081R	gbs1796		General function predicted only		NAD(FAD)-utilizing dehydrogenases	1,132	P	A	P	A	A	P	A	A	0,710	M	A	A	A	A	A	A	A
1030860	COG1085C	gbs1797		Metabolism and transport	Energy production and conversion	Galactose-1-phosphate uridylyltransferase (EC 2.7.7.10)	1,121	P	P	P	P	P	P	P	M	0,677	P	A	P	P	A	P	P	A
1030855	COG0199J	gbs1798	rpsN2	Cellular processess	Translation, ribosomal structure and biogenesis	SSU ribosomal protein S14P	0,444	P	P	P	P	P	P	P	P	1,668	P	P	P	P	P	P	P	P
1030854	COG2008E	gbs1799		Metabolism and transport	Amino acids, peptides, aminosugars and amines	Low-specificity L-threonine aldolase (EC 4.1.2.5)	1,301	P	P	P	P	P	P	P	P	0,982	P	A	P	A	P	P	M	A
1030853	COG0533O	gbs1800		Cellular processess	Posttranslational modification, protein turnover, chaperones	O-sialoglycoprotein endopeptidase (EC 3.4.24.57)	1,999	P	A	M	A	A	A	A	A	1,027	P	A	A	A	A	A	P	A
1030862	COG0456R	gbs1801		Cellular processess	Translation, ribosomal structure and biogenesis	Ribosomal-protein-S18-alanine acetyltransferase (EC 2.3.1.128)	2,932	P	P	P	P	P	P	P	P	0,939	P	A	P	A	M	P	P	A
1030865	COG1214O	gbs1802		Cellular processess	Posttranslational modification, protein turnover, chaperones	Non-proteolytic protein, peptidase family M22	2,595	P	A	M	A	A	A	A	A	1,250	P	A	A	A	A	A	P	A
1030868	-	gbs1803		General function predicted only		Putative transcriptional regulator	2,689	P	P	P	P	P	P	P	P	1,539	P	P	P	P	P	P	P	P
1030867	COG0595R	gbs1804		General function predicted only		Zn-dependent hydrolase (EC 3.-.-.-)	2,285	P	P	P	P	P	P	P	P	1,702	P	A	P	A	P	P	P	A
1030861	COG3942R	gbs1805		General function predicted only		Secreted protein	2,208	P	A	A	A	A	P	A	A	0,828	A	A	A	A	A	A	A	A
1030872	-	gbs1806	glnA	Metabolism and transport	Amino acids, peptides, aminosugars and amines	Glutamine synthetase (EC 6.3.1.2)	6,862	P	M	P	P	P	P	P	P	2,133	P	A	A	A	A	P	P	A
1030864	COG0789K	gbs1807		Cellular processess	Transcription	Transcriptional regulator, MerR family	2,655	P	P	P	P	P	P	P	P	0,972	P	P	P	P	P	P	P	P
1030866	COG4129S	gbs1808		Hypothetical		Hypothetical protein	1,258	P	M	P	P	P	P	P	P	0,790	P	A	P	P	A	P	P	P
1030870	-	gbs1809	pgk	Metabolism and transport	Carbohydrates, organic alcohols, and acids	Phosphoglycerate kinase (EC 2.7.2.3)	0,522	P	P	P	P	P	P	P	P	0,644	P	P	P	P	P	P	P	P
1030873	COG2503R	gbs1810	lppC	Metabolism and transport	Cofactors, prosthetic groups, and carriers	Acid phosphatase (EC 3.1.3.2)	0,476	P	P	P	P	P	P	P	P	0,748	P	P	P	P	P	P	P	P
1030869	COG0057G	gbs1811	gapC/plr	Metabolism and transport	Carbohydrates, organic alcohols, and acids	Glyceraldehyde 3-phosphate dehydrogenase (EC 1.2.1.12) GAPDH	0,371	P	P	P	P	P	P	P	P	0,418	P	P	P	P	P	P	P	P
1030871	COG0480J	gbs1812	fus	Cellular processess	Translation, ribosomal structure and biogenesis	Protein Translation Elongation Factor G (EF-G)	0,951	P	P	P	P	P	P	P	P	0,896	P	P	P	P	P	P	P	P
1030883	COG0049J	gbs1813	rpsG	Cellular processess	Translation, ribosomal structure and biogenesis	SSU ribosomal protein S7P	1,715	P	P	P	P	P	P	P	P	0,723	P	P	P	P	P	P	P	P
1030884	-	gbs1814	rpsL	Cellular processess	Translation, ribosomal structure and biogenesis	SSU ribosomal protein S12P	1,545	P	P	P	P	P	P	P	P	0,665	P	P	P	P	P	P	P	P
1030890	COG0503F	gbs1815	purR	Metabolism and transport	Purines, pyrimidines, nucleosides, and nucleotides	Pur operon repressor	0,592	P	P	P	P	P	P	P	P	1,059	P	P	P	M	P	P	P	P
1030876	COG3481R	gbs1816	cbf	General function predicted only		CMP-binding factor	1,727	P	P	P	P	P	P	P	P	1,621	P	P	P	P	P	P	P	P
1030896	COG1322S	gbs1817		General function predicted only		RmuC family protein	3,379	P	A	M	A	P	P	A	P	2,173	P	A	P	A	P	P	A	A
1030891	COG1564H	gbs1818		Metabolism and transport	Cofactors, prosthetic groups, and carriers	Thiamin pyrophosphokinase (EC 2.7.6.2)	2,971	P	A	P	A	P	P	M	A	1,513	P	A	P	A	P	P	P	A
1030894	COG0036G	gbs1819	rpe	Metabolism and transport	Carbohydrates, organic alcohols, and acids	Ribulose-phosphate 3-epimerase (EC 5.1.3.1)	1,686	P	P	P	P	P	P	P	P	1,364	P	A	P	P	P	P	P	P
1030900	COG1162R	gbs1820		General function predicted only		GTPase (EC 3.6.1.-)	3,276	P	M	P	A	P	M	M	P	2,236	P	A	P	A	A	A	M	A
1030895	-	gbs1821		Cellular processess	Translation, ribosomal structure and biogenesis	23S rRNA m(1)G 745 methyltransferase (EC 2.1.1.51)	3,912	P	A	A	A	M	P	A	A	1,167	P	A	M	A	A	P	A	A
1030893	COG0030J	gbs1822	ksgA	Cellular processess	Translation, ribosomal structure and biogenesis	Dimethyladenosine transferase (EC 2.1.1.-)	2,103	P	A	A	A	A	M	A	P	1,111	P	A	A	A	A	A	A	A
1030892	COG0537FGR	gbs1823		General function predicted only		HIT family hydrolase	2,102	P	P	P	P	P	P	P	P	1,078	P	A	P	P	P	P	P	P
1030897	COG1658L	gbs1824		Cellular processess	Transcription	Ribonuclease M5 (EC 3.1.26.8)	1,092	P	A	M	A	P	A	M	A	0,905	P	A	A	A	A	P	A	A
1030898	COG0084L	gbs1825	tatD	Cellular processess	DNA replication, recombination and repair	DNase, TatD family (EC 3.1.-.-)	2,061	P	A	A	A	P	M	P	A	1,108	P	A	A	A	A	A	A	A
1030899	-	gbs1826		Hypothetical		Hypothetical protein	2,223	P	P	P	P	P	P	P	P	1,518	P	A	P	A	P	P	A	P
1030909	-	gbs1827		Hypothetical		Hypothetical protein	2,366	P	P	P	P	P	P	P	P	1,961	P	M	P	A	P	P	P	A
1030903	-	gbs1828		Hypothetical		Hypothetical cytosolic protein	1,866	P	P	P	P	P	P	P	P	1,705	P	A	P	A	A	M	A	A
1030902	-	gbs1829		Hypothetical		Hypothetical secreted protein	1,357	P	P	P	P	P	P	P	P	1,581	P	A	P	A	P	P	A	P
1030904	COG3966M	gbs1830	dltD	General function predicted only		Protein dltD precursor	3,241	P	P	P	P	P	P	P	P	1,984	P	A	A	A	A	P	A	M
1030910	COG0236IQ	gbs1831		Metabolism and transport	Fatty acid and phospholipids	D-alanyl carrier protein	2,230	P	P	P	P	P	P	P	P	2,276	P	A	P	A	P	P	A	P
1030913	COG1696M	gbs1832		General function predicted only		Protein dltB	3,011	P	P	P	P	P	P	P	P	1,972	P	A	P	M	P	P	P	A
1030911	COG1020Q	gbs1833	dltA	Metabolism and transport	Secondary metabolites	D-alanine-activating enzyme (EC 6.3.2.-)	3,248	P	P	P	P	P	P	P	P	1,774	P	A	P	A	A	P	M	A
1030917	COG0642T	gbs1834		Cellular processess	Signal transduction	Sensory transduction protein kinase (EC 2.7.3.-)	3,388	P	A	A	A	A	A	A	A	2,319	P	A	A	A	A	A	A	A
1030914	COG0745TK	gbs1835		Cellular processess	Transcription	Transcriptional regulatory protein	3,158	P	P	P	P	P	P	P	P	1,839	P	A	P	A	A	P	M	P
1030915	-	gbs1836	rpmH	Cellular processess	Translation, ribosomal structure and biogenesis	LSU ribosomal protein L34P	0,807	P	P	P	P	P	P	P	P	0,755	P	A	P	A	M	P	P	P
1030912	COG3314S	gbs1837		Hypothetical		Hypothetical protein	2,620	P	A	A	A	A	A	A	A	0,887	P	A	A	A	A	A	A	A
1030922	32113E, COG4	gbs1838	opuABC	Metabolism and transport	Amino acids, peptides, aminosugars and amines	Glycine betaine-binding protein Glycine betaine transport system permease prote	0,880	P	P	P	P	P	P	P	P	0,873	P	A	P	A	P	P	A	P
1030926	COG4175E	gbs1839	opuAA	Metabolism and transport	Amino acids, peptides, aminosugars and amines	Glycine betaine transport ATP-binding protein	0,827	P	P	P	A	P	P	P	P	0,738	P	A	A	A	P	P	P	A
1030923	COG3957G	gbs1840		Metabolism and transport	Carbohydrates, organic alcohols, and acids	Xylulose-5-phosphate (EC 4.1.2.9) Fructose-6-phosphate phosphoketolase (EC 4	0,270	A	A	A	A	A	A	A	A	0,395	P	P	P	P	A	P	P	P
1030916	COG2220R	gbs1841		Cellular processess	Toxin production and resistance	Metal-dependent hydrolase (EC 3.-.-.-)	0,180	P	P	P	P	P	P	P	P	0,615	P	P	P	P	P	P	P	P
1030928	COG3711K	gbs1842		Cellular processess	Transcription	Transcription antiterminator, BglG family	0,835	P	P	P	P	P	P	P	P	0,789	P	P	P	P	P	P	P	P
1030929	-	gbs1843		General function predicted only		protein with aldose 1-epimerase motif involved in 2-ketogluconate utilization	0,480	A	A	A	A	A	A	A	A	0,096	A	M	A	A	P	A	A	A

1030924	COG1070G	gbs1844		Metabolism and transport	Carbohydrates, organic alcohols, and acids	L-xylulokinase (EC 2.7.1.53)	1,190	A	A	A	A	A	A	A	A	0,404	A	A	A	A	A	A	A	A
1030919	COG2159R	gbs1845		Metabolism and transport	Purines, pyrimidines, nucleosides, and nucleotides	Amidohydrolase	0,481	A	A	A	A	A	A	A	A	1,652	A	A	A	A	A	A	A	A
1030921	COG3775G	gbs1846		Metabolism and transport	Carbohydrates, organic alcohols, and acids	PTS system, galactitol-specific IIC component (EC 2.7.1.69)	0,741	A	A	A	A	A	A	A	A	1,552	A	A	A	A	A	A	A	A
1030932	COG1052CHR	gbs1847		Metabolism and transport	Central intermediary metabolism	Glyoxylate reductase (NADP+) (EC 1.1.1.79) Glyoxylate reductase (NAD+) (EC 1	0,640	P	A	P	A	P	A	A	A	0,715	P	A	A	A	A	A	A	A
1030931	-	gbs1848		Hypothetical		Hypothetical protein	0,625	A	A	A	A	A	A	A	A	0,503	A	A	A	A	A	A	A	A
1030925	COG1609K	gbs1849		Cellular processess	Transcription	Kdg operon repressor	1,718	P	A	A	A	A	A	A	A	1,912	P	A	A	A	A	A	A	A
1030936	-	gbs1850		Metabolism and transport	Energy production and conversion	Transaldolase (EC 2.2.1.2)	0,745	P	A	M	A	A	A	A	P	0,772	P	P	P	P	P	P	P	A
1030939	COG0235G	gbs1851	araD	Metabolism and transport	Carbohydrates, organic alcohols, and acids	L-ribulose-5-phosphate 4-epimerase (EC 5.1.3.4)	0,696	P	A	P	A	A	A	A	A	0,770	P	P	P	P	A	P	P	A
1030935	COG3623G	gbs1852		Metabolism and transport	Carbohydrates, organic alcohols, and acids	L-xylulose 5-phosphate 3-epimerase (EC 5.3.1.-)	0,561	P	P	P	P	P	P	P	P	0,863	P	P	P	P	P	P	P	P
1030930	COG0269G	gbs1853		Metabolism and transport	Carbohydrates, organic alcohols, and acids	3-keto-L-gulonate-6-phosphate decarboxylase (EC 4.1.1.-)	0,580	P	M	M	M	P	P	M	P	0,794	P	P	P	P	P	P	P	P
1030937	COG1762GT	gbs1854		Metabolism and transport	Carbohydrates, organic alcohols, and acids	PTS system, 3-keto-L-gulonate specific IIA component (EC 2.7.1.69)	0,595	P	A	A	A	A	A	A	P	1,100	P	P	P	P	P	P	P	P
1030933	COG3414G	gbs1855		Metabolism and transport	Carbohydrates, organic alcohols, and acids	PTS system, 3-keto-L-gulonate specific IIB component (EC 2.7.1.69)	0,273	P	M	P	P	P	P	P	P	1,000	P	P	P	P	P	P	P	P
1030941	-	gbs1856	ulaA	Metabolism and transport	Carbohydrates, organic alcohols, and acids	PTS system, 3-keto-L-gulonate specific IIC component (EC 2.7.1.69)	0,192	P	P	P	P	P	P	P	P	0,969	P	P	P	P	P	P	P	P
1030938	-	gbs1857		Hypothetical		Hypothetical membrane associated protein	1,115	P	P	P	P	P	P	P	M	1,291	P	A	P	A	A	P	P	A
1030943	-	gbs1858		Hypothetical		Hypothetical protein	1,892	P	A	A	A	A	M	A	A	0,600	P	A	A	A	A	A	A	A
1030945	-	gbs1859	purA	Metabolism and transport	Purines, pyrimidines, nucleosides, and nucleotides	Adenylosuccinate synthetase (EC 6.3.4.4)	0,655	P	P	P	P	P	P	P	P	0,406	P	P	A	P	A	P	P	P
1030942	COG1299G	gbs1860	sloR	Cellular processess	Transcription	Transcriptional regulator pfoR	1,501	P	A	A	A	A	A	A	A	1,682	P	A	P	A	A	A	A	A
1030940	COG2964S	gbs1861		General function predicted only		Putative DNA-binding protein	0,847	P	P	P	P	P	P	P	P	0,748	P	P	P	P	P	P	P	P
1030947	:1181M, COG2	gbs1862		Metabolism and transport	Cofactors, prosthetic groups, and carriers	Amino acid ligase family protein (putative polyamide biosynthesis enzyme)	1,195	P	A	A	A	A	A	A	A	1,142	A	A	A	A	A	A	A	A
1030950	-	gbs1863		Hypothetical		Hypothetical membrane associated protein	1,744	P	P	P	M	P	P	A	P	3,271	P	A	A	A	A	P	A	A
1030944	COG3853P	gbs1864		Metabolism and transport	Inorganic ion transport and metabolism	Tellurite resistance protein	1,109	P	M	P	M	P	P	A	P	1,307	P	A	A	P	M	A	A	A
1030949	-	gbs1865	hslO	Cellular processess	Posttranslational modification, protein turnover, chaperones	33 kDa chaperonin	5,227	P	A	A	A	P	P	P	P	1,301	P	A	A	A	A	M	A	A
1030948	COG0042J	gbs1866		Cellular processess	Translation, ribosomal structure and biogenesis	tRNA-dihydrouridine synthase	1,702	P	A	A	A	A	A	A	A	0,632	A	A	A	A	A	A	A	A
1030951	COG1428F	gbs1867		Metabolism and transport	Purines, pyrimidines, nucleosides, and nucleotides	Deoxyadenosine kinase (EC 2.7.1.76) Deoxyguanosine kinase (EC 2.7.1.113)	2,435	P	A	P	A	A	A	A	A	0,444	P	P	A	A	A	A	M	A
1030946	COG1247M	gbs1868	clpC	Metabolism and transport	Fatty acid and phospholipids	Phosphinothricin N-acetyltransferase (EC 2.3.1.-)	3,998	P	A	P	A	A	P	A	A	0,558	P	A	A	A	A	P	A	M
1030952	COG0542O	gbs1869	clpC	Cellular processess	Transcription	Negative regulator of genetic competence clpC mecB	1,753	P	P	P	P	P	P	P	P	0,998	P	P	P	P	P	P	P	P
1030958	-	gbs1870	ctsR	Cellular processess	Transcription	Transcriptional regulator ctsR	3,310	P	P	P	P	P	P	P	P	1,282	P	P	P	P	P	P	P	P
1030955	-	gbs1871		Cellular processess	Toxin production and resistance	Small multidrug export protein	1,518	P	P	P	P	P	P	P	P	1,056	P	A	A	A	A	M	A	A
1030953	-	gbs1872	tsf	Cellular processess	Translation, ribosomal structure and biogenesis	Protein Translation Elongation Factor Ts (EF-Ts)	1,427	P	P	P	P	P	P	P	P	0,839	P	P	P	A	P	P	P	P
1030963	-	gbs1873	rpsB	Cellular processess	Translation, ribosomal structure and biogenesis	SSU ribosomal protein S2P	1,747	P	P	P	P	P	P	P	P	0,823	P	P	P	P	P	P	P	P
1030961	COG0450O	gbs1874	ahpC	Metabolism and transport	Energy production and conversion	Peroxiredoxin (EC 1.11.1.15)	0,693	P	P	P	P	P	P	P	P	1,025	P	P	P	P	P	P	P	P
1030959	COG3634O	gbs1875	ahpF	Metabolism and transport	Energy production and conversion	Peroxiredoxin reductase (NAD(P)H) (EC 1.6.4.-) NADH oxidase H2O2-forming (E	0,879	P	P	P	P	P	P	P	P	1,134	P	P	P	P	P	P	P	P
1030954	COG0025P	gbs1876		Metabolism and transport	Inorganic ion transport and metabolism	Na+ H+ antiporter nhaP	2,552	P	A	A	A	A	A	A	A	1,009	P	A	A	A	A	A	A	A
1030965	-	gbs1877		Hypothetical		Hypothetical protein	2,880	P	M	M	A	A	A	A	A	1,653	P	A	A	A	A	A	A	A
1030964	COG1619V	gbs1878	mccF	Cell Envelope	Biosynthesis and degradation of murein sacculus and peptidoglycan	Muramoyltetrapeptide carboxypeptidase (EC 3.4.17.13)	0,510	P	P	P	P	P	P	P	P	0,712	P	P	P	P	P	P	P	P
1030971	COG3590O	gbs1879	pepO	Cellular processess	Posttranslational modification, protein turnover, chaperones	Oligoendopeptidase O (EC 3.4.24.-)	1,106	P	P	P	P	P	P	P	P	1,303	P	A	P	A	P	P	P	P
1030970	COG0673R	gbs1880		Metabolism and transport	Central intermediary metabolism	NAD-dependent oxidoreductase	0,967	P	M	P	A	P	P	A	P	0,586	A	A	A	A	A	A	A	A
1030967	-	gbs1881		Transport and binding proteins	Unknown substrate	Transporter, MFS superfamily	1,009	P	A	A	A	A	A	A	A	1,183	P	A	A	A	A	P	P	A
1030962	COG0664T	gbs1882		Cellular processess	Signal transduction	Catabolite gene activator	2,252	P	M	P	A	P	P	P	P	0,810	P	A	A	A	A	A	A	A
1030973	-	gbs1883	def	Cellular processess	Posttranslational modification, protein turnover, chaperones	Peptide deformylase (EC 3.5.1.88)	1,653	P	P	P	P	P	P	P	P	1,326	P	P	P	A	P	P	P	P
1030969	COG1609K	gbs1884	regR	Cellular processess	Transcription	Transcriptional regulator, LacI family	1,254	P	A	P	A	P	A	A	P	1,346	P	A	P	A	P	A	A	A
1030968	-	gbs1885		Hypothetical		Oligohyaluronate lyase (EC 4.2.2.-)	0,497	A	A	A	A	A	A	A	A	0,415	A	A	A	A	A	A	A	A
1030976	-	gbs1886	agaD	Metabolism and transport	Carbohydrates, organic alcohols, and acids	PTS system, N-acetylgalactosamine-specific IID component (EC 2.7.1.69)	0,447	P	A	M	A	P	A	A	A	0,935	P	A	A	M	A	P	A	A
1030974	-	gbs1887		Metabolism and transport	Carbohydrates, organic alcohols, and acids	PTS system, N-acetylgalactosamine-specific IIC component (EC 2.7.1.69)	0,252	A	A	A	A	A	A	A	A	0,582	M	A	A	A	A	A	M	A
1030980	-	gbs1888	agaV	Metabolism and transport	Carbohydrates, organic alcohols, and acids	PTS system, N-acetylgalactosamine-specific IIB component (EC 2.7.1.69)	1,176	A	A	A	A	A	A	A	A	0,922	A	A	A	A	A	A	A	A
1030972	COG4225R	gbs1889		General function predicted only		Unsaturated glucuronyl hydrolase (EC 3.2.1.-)	0,441	A	A	A	A	A	A	A	A	0,613	A	A	A	A	A	A	A	A
1030988	COG2893G	gbs1890		Metabolism and transport	Carbohydrates, organic alcohols, and acids	PTS system, N-acetylgalactosamine-specific IIA component (EC 2.7.1.69)	0,564	P	A	A	A	A	A	A	A	0,421	P	A	A	A	A	M	A	A
1030993	COG1028IQR	gbs1891	idnO	Metabolism and transport	Fatty acid and phospholipids	2-deoxy-D-gluconate 3-dehydrogenase (EC 1.1.1.125)	0,473	A	A	A	A	A	A	A	A	0,387	A	A	A	A	A	A	A	A
1030995	COG0698G	gbs1892		Metabolism and transport	Carbohydrates, organic alcohols, and acids	Galactose-6-phosphate isomerase LacB subunit (EC 5.3.1.26)	0,827	A	A	A	A	A	A	A	A	0,706	P	A	A	A	A	P	A	A
1030975	COG0524G	gbs1893		Metabolism and transport	Carbohydrates, organic alcohols, and acids	2-dehydro-3-deoxygluconokinase (EC 2.7.1.45)	0,421	A	A	A	A	A	A	A	A	0,769	A	A	A	A	A	A	A	A
1031009	COG0800G	gbs1894	kgdA	Metabolism and transport	Carbohydrates, organic alcohols, and acids	4-Hydroxy-2-oxoglutarate aldolase (EC 4.1.3.16) 2-dehydro-3-deoxyphosphogluc	0,730	A	A	A	A	A	A	A	A	0,491	A	A	A	A	A	A	A	A
1031001	-	gbs1895		Hypothetical		Hypothetical protein	0,987	P	P	P	P	P	P	P	P	5,928	P	A	A	A	A	P	P	P
1031005	COG3560R	gbs1896		General function predicted only		Nitroreductase family protein	0,359	P	P	P	P	P	P	P	P	0,511	P	A	P	P	A	P	P	P
1031010	COG1846K	gbs1897		Cellular processess	Transcription	Transcriptional regulator, MarR family	1,928	P	A	A	A	A	M	A	A	1,123	P	A	A	A	A	A	A	M
1031006	COG2176L	gbs1898	polC	Cellular processess	DNA replication, recombination and repair	DNA polymerase III alpha subunit (EC 2.7.7.7)	1,273	P	A	P	P	P	P	P	A	1,354	P	A	P	A	A	P	A	P
1031003	COG1705NU	gbs1899	mur1.2	Cell Envelope	Biosynthesis and degradation of murein sacculus and peptidoglycan	N-acetylmuramidase (EC 3.2.1.17)	1,972	P	P	P	P	P	P	P	A	0,428	P	A	A	A	A	P	A	A
1031002	COG0442J	gbs1900	proS	Cellular processess	Translation, ribosomal structure and biogenesis	Prolyl-tRNA synthetase (EC 6.1.1.15)	1,486	P	P	P	A	P	P	P	A	0,853	P	A	P	A	A	P	A	A
1031007	COG0750M	gbs1901		Cellular processess	Posttranslational modification, protein turnover, chaperones	Membrane endopeptidase, M50 family	1,789	P	A	P	A	P	P	A	A	1,065	P	A	M	A	A	P	A	A
1031014	COG0575I	gbs1902	cdsA	Metabolism and transport	Fatty acid and phospholipids	Phosphatidate cytidylyltransferase (EC 2.7.7.41)	2,505	P	P	P	A	P	P	A	A	0,998	P	M	P	A	A	P	A	A
1031008	COG0020I	gbs1903	uppS	Metabolism and transport	Fatty acid and phospholipids	Undecaprenyl pyrophosphate synthetase (EC 2.5.1.31)	2,161	P	P	P	P	P	P	P	A	1,227	P	A	P	A	P	P	A	M
1031015	COG1862U	gbs1904	yajC	Cellular processess	Posttranslational modification, protein turnover, chaperones	Protein translocase subunit YajC	0,685	P	P	P	P	P	P	P	P	0,847	P	P	P	P	P	P	P	P
1031019	-	gbs1905		Metabolism and transport	Energy production and conversion	Thioredoxin	1,265	P	P	P	P	P	P	P	P	1,339	P	A	P	P	P	P	P	P
1031021	COG0281C	gbs1906		Metabolism and transport	Energy production and conversion	NAD-dependent malic enzyme (EC 1.1.1.39)	0,693	P	A	A	P	A	A	A	A	1,017	P	A	A	P	A	A	A	A
1031011	-	gbs1907	malP	Metabolism and transport	Carbohydrates, organic alcohols, and acids	Malate-sodium symport	1,176	P	A	A	A	A	A	A	A	3,242	P	A	A	P	A	A	A	A
1031022	COG3290T	gbs1908	dpiB	Cellular processess	Signal transduction	Sensor kinase dpiB (EC 2.7.3.-)	1,363	P	A	A	M	P	A	P	A	1,607	P	A	A	A	A	A	A	A
1031023	COG4565KT	gbs1909	dpiA	Cellular processess	Transcription	Transcriptional regulatory protein	1,120	P	A	A	A	A	A	A	A	1,172	P	A	A	A	A	A	A	A
1031020	COG1087M	gbs1910		Metabolism and transport	Energy production and conversion	UDP-glucose 4-epimerase (EC 5.1.3.2)	0,810	P	P	P	P	P	P	P	P	1,448	P	M	P	P	P	P	P	P
1031027	-	gbs1911	dexB	Metabolism and transport	Carbohydrates, organic alcohols, and acids	Glucan 1,6-alpha-glucosidase (EC 3.2.1.70)	0,477	P	P	P	P	P	P	P	P	1,856	P	A	P	P	A	P	P	P
1031029	COG3839G	gbs1912	msmK	Metabolism and transport	Carbohydrates, organic alcohols, and acids	Multiple sugar transport ATP-binding protein msmK	0,882	P	P	P	P	P	P	P	P	1,702	P	P	P	P	P	P	P	P
1031025	COG2508TQ	gbs1913	lrp	Cellular processess	Signal transduction	Leucine rich protein	1,543	P	A	A	A	M	A	A	A	1,443	P	A	A	A	A	A	A	A
1031024	COG2017G	gbs1914		Metabolism and transport	Carbohydrates, organic alcohols, and acids	Aldose 1-epimerase family protein	1,298	P	A	A	A	P	A	A	A	6,943	P	A	A	A	A	A	A	A
1031035	COG3684G	gbs1915		Metabolism and transport	Carbohydrates, organic alcohols, and acids	Tagatose-bisphosphate aldolase (EC 4.1.2.40)	0,324	A	P	A	M	M	A	A	A	3,800	P	A	P	A	A	A	A	A
1031028	COG1105G	gbs1916		Metabolism and transport	Carbohydrates, organic alcohols, and acids	Tagatose-6-phosphate kinase (EC 2.7.1.144)	2,112	A	A	A	A	A	A	A	A	18,228	P	A	A	A	A	A	A	A
1031032	COG0698G	gbs1917		Metabolism and transport	Carbohydrates, organic alcohols, and acids	Galactose-6-phosphate isomerase lacB subunit (EC 5.3.1.26)	0,575	A	P	A	A	A	A	P	A	7,097	P	A	A	A	M	P	M	A
1031048	COG0698G	gbs1918	lacA.1	Metabolism and transport	Carbohydrates, organic alcohols, and acids	Galactose-6-phosphate isomerase lacA subunit (EC 5.3.1.26)	0,938	A	A	M	A	A	A	A	A	42,462	P	A	A	A	A	A	A	A
1031043	COG4409G	gbs1919		Metabolism and transport	Carbohydrates, organic alcohols, and acids	Sialidase A precursor (EC 3.2.1.18)	2,239	P	A	A	A	A	A	A	A	3,296	P	A	A	A	A	A	A	A
1031030	COG3775G	gbs1920		Metabolism and transport	Carbohydrates, organic alcohols, and acids	PTS system, galactose-specific IIC component (EC 2.7.1.69)	0,499	P	A	A	A	A	A	P	A	8,580	P	M	M	A	M	P	P	A
1031054	COG3414G	gbs1921		Metabolism and transport	Carbohydrates, organic alcohols, and acids	PTS system, galactose-specific IIB component (EC 2.7.1.69)	0,470	P	A	A	A	A	A	A	A	23,989	P	A	A	A	A	A	P	A
1031044	COG1762GT	gbs1922		Metabolism and transport	Carbohydrates, organic alcohols, and acids	PTS system, galactose-specific IIA component (EC 2.7.1.69)	0,417	P	A	P	A	P	P	P	P	14,315	P	A	P	A	A	M	P	A
1031037	COG1349KG	gbs1923	lacR.1	Cellular processess	Transcription	Lactose phosphotransferase system repressor	0,460	P	P	P	P	P	P	P	P	1,604	P	A	P	P	P	M	P	A
1031057	-	gbs1925		Mobile and extrachromosomal elements		Streptococcal histidine triad protein	1,145	P	A	A	A	A	A	A	A	0,667	P	A	A	A	A	A	A	A
1031045	COG0803P	gbs1926		Metabolism and transport	Inorganic ion transport and metabolism	Laminin-binding surface protein	1,234	P	A	A	A	A	A	A	A	0,714	P	A	A	A	A	A	A	A
1031062	-	gbs1927		Cellular processess	Translation, ribosomal structure and biogenesis	D-tyrosyl-tRNA(Tyr) deacylase (EC 3.1.-.-)	1,500	P	P	P	P	P	P	P	P	0,740	P	A	P	A	A	P	A	A
1031064	COG0317TK	gbs1928	relA	Cellular processess	Signal transduction	GTP pyrophosphokinase (EC 2.7.6.5) Guanosine-3 ,5 -bis(Diphosphate) 3 -pyrop	1,408	P	A	P	A	M	A	A	A	0,872	P	A	A	A	A	A	A	A
1031058	COG0737F	gbs1929		Metabolism and transport	Purines, pyrimidines, nucleosides, and nucleotides	LPXTG 2 ,3 -cyclic-nucleotide 2 -phosphodiesterase (EC 3.1.4.16) 3 -nucleotidas	0,709	P	P	P	P	P	P	P	P	1,211	P	P	P	P	P	P	P	P
1031061	COG1780F	gbs1930		Metabolism and transport	Purines, pyrimidines, nucleosides, and nucleotides	NrdI protein	1,028	P	P	P	P	P	P	P	P	1,274	P	A	P	A	P	P	A	M
1031076	COG1363G	gbs1931		Metabolism and transport	Carbohydrates, organic alcohols, and acids	Deblocking aminopeptidase (EC 3.4.11.-)	1,410	P	P	P	P	P	P	P	P	1,500	P	A	P	M	A	P	A	A
1031063	-	gbs1932		General function predicted only		Putative kinase	1,554	P	P	P	P	P	P	P	P	1,590	P	A	P	P	M	P	A	A
1031060	COG1840P	gbs1933		Metabolism and transport	Inorganic ion transport and metabolism	Iron(III)-binding protein	1,289	P	A	A	A	A	A	A	A	2,764	P	A	P	A	M	P	P	P
1031078	COG4753T	gbs1934		Cellular processess	Signal transduction	Two-component response regulator yesN	9,991	P	A	A	A	A	A	A	A	3,162	P	A	P	A	A	P	A	P
1031079	COG2972T	gbs1935		Cellular processess	Signal transduction	Two-component sensor kinase yesM (EC 2.7.3.-)	4,091	P	A	A	A	A	A	A	A	4,113	P	A	A	A	A	A	M	A
1031074	-	gbs1936	ptsD	Metabolism and transport	Carbohydrates, organic alcohols, and acids	PTS system, mannose fructose family IID component	1,578	P	A	M	P	P	P	A	P	2,206	P	P	P	P	P	P	P	P
1031065	-	gbs1937	ptsC	Metabolism and transport	Carbohydrates, organic alcohols, and acids	PTS system, mannose fructose family IIC component	3,098	P	A	A	A	A	A	A	A	2,569	P	P	P	P	P	P	P	P
1031081	-	gbs1938	ptsB	Metabolism and transport	Carbohydrates, organic alcohols, and acids	PTS system, mannose fructose family IIB component	2,182	P	P	P	P	P	P	A	P	2,507	P	P	P	P	P	P	P	P
1031080	COG2893G	gbs1939		Metabolism and transport	Carbohydrates, organic alcohols, and acids	PTS system, mannose fructose family IIA component	1,711	P	A	P	A	P	A	A	M	1,560	P	P	P	P	P	P	P	P

1031082	-	gbs1940		Hypothetical		Hypothetical membrane spanning protein	12,117	P	A	A	A	A	A	A	A	5,233	P	A	A	A	A	A	A	A
1031075	-	gbs1941		Transport and binding proteins	Unknown substrate	Transporter	2,035	P	P	P	M	P	P	A	P	2,366	P	A	P	A	A	P	P	P
1031084	COG1131V	gbs1942		Transport and binding proteins	Unknown substrate	ABC transporter ATP-binding protein	0,995	P	P	P	A	P	P	A	A	3,578	P	A	P	A	A	A	A	A
1031085	COG2972T	gbs1943	fasB	hypothetical		Hypothetical protein	2,541	P	P	P	P	P	P	A	P	3,267	P	A	A	A	A	A	A	A
1031083	COG3279KT	gbs1944	fasA	Cellular processess	Transcription	Response regulator FasA	2,505	P	P	P	P	P	P	P	P	2,550	P	A	P	M	A	P	P	P
1031089	COG3568R	gbs1945		General function predicted only		Endonuclease Exonuclease phosphatase family protein	0,699	P	A	A	A	A	P	A	A	3,292	P	A	P	A	M	P	P	M
1031087	COG1263G	gbs1946		Metabolism and transport	Carbohydrates, organic alcohols, and acids	PTS system, glucose-specific IIABC component (EC 2.7.1.69)	0,356	P	A	P	P	P	P	P	P	3,805	P	P	P	P	P	P	P	P
1031091	COG0642T	gbs1947		Cellular processess	Signal transduction	Phosphate regulon sensor protein phoR (EC 2.7.3.-)	1,557	P	P	P	A	P	P	P	A	1,432	P	A	P	A	A	A	A	A
1031090	COG0745TK	gbs1948		Cellular processess	Signal transduction	Alkaline phosphatase synthesis two-component response regulator phoP	1,145	P	A	P	A	P	P	A	P	1,214	P	A	A	A	M	A	A	A
1031086	COG0704P	gbs1949		Metabolism and transport	Inorganic ion transport and metabolism	Phosphate transport system protein phoU	3,457	P	A	A	A	A	A	A	A	1,597	A	A	A	A	A	A	A	A
1031103	COG1117P	gbs1950		Metabolism and transport	Inorganic ion transport and metabolism	Phosphate transport ATP-binding protein pstB	2,722	A	A	A	A	A	A	A	A	0,505	A	A	A	A	A	A	A	A
1031094	COG0581P	gbs1951		Metabolism and transport	Inorganic ion transport and metabolism	Phosphate transport system permease protein pstA	2,673	A	A	A	A	A	A	A	A	0,401	A	A	A	A	A	A	A	A
1031092	COG0573P	gbs1952		Metabolism and transport	Inorganic ion transport and metabolism	Phosphate transport system permease protein pstC	1,169	A	A	A	A	A	A	A	A	0,165	A	A	A	A	A	A	A	A
1031095	COG0226P	gbs1953		Metabolism and transport	Inorganic ion transport and metabolism	probable hemolysin precursor	1,778	P	A	A	A	A	A	A	A	0,967	A	A	A	A	A	A	A	A
1031108	-	gbs1954		Hypothetical		Hypothetical protein	1,835	P	A	P	A	P	P	A	A	1,043	P	A	P	A	A	P	A	P
1031100	COG1385S	gbs1955		Hypothetical		Hypothetical cytosolic protein	3,141	P	A	A	A	A	A	A	A	3,669	A	A	A	A	A	A	A	A
1031096	COG2264J	gbs1956		Cellular processess	Translation, ribosomal structure and biogenesis	Ribosomal protein L11 methyltransferase (EC 2.1.1.-)	0,699	P	A	P	P	P	P	P	P	0,616	P	A	P	A	P	P	M	A
1031099	COG4815S	gbs1957		Hypothetical		Hypothetical cytosolic protein	1,235	P	P	P	P	P	P	P	P	0,899	P	A	P	P	P	P	P	P
1031111	COG0789K	gbs1958		Cellular processess	Transcription	Transcriptional activator tipA	0,544	P	P	P	P	P	P	P	P	0,623	P	A	P	A	P	P	P	M
1031101	-	gbs1959		Metabolism and transport	Central intermediary metabolism	Acetyltransferase (EC 2.3.1.-)	0,661	P	M	P	P	M	P	A	P	1,182	P	A	A	A	A	M	A	P
1031110	COG0494LR	gbs1960		Cellular processess	DNA replication, recombination and repair	Phosphohydrolase (MutT nudix family protein)	0,651	P	P	P	P	P	P	P	P	0,898	P	A	M	A	A	M	A	A
1031106	-	gbs1961		Hypothetical		Hypothetical protein	1,009	P	P	P	P	P	P	P	P	1,626	P	P	P	P	P	P	P	P
1031114	-	gbs1962		Hypothetical		Hypothetical protein	0,940	P	A	P	A	M	A	A	A	1,155	P	A	A	A	A	A	A	A
1031107	-	gbs1963		Metabolism and transport	Central intermediary metabolism	Acetyltransferase, GNAT family	1,111	P	A	M	A	A	A	A	A	0,744	A	A	A	A	A	A	A	A
1031118	COG2256L	gbs1964		Cellular processess	DNA replication, recombination and repair	ATPase, AAA family	1,997	P	P	P	A	A	A	A	A	2,564	P	A	A	A	A	A	A	A
1031109	-	gbs1965		Hypothetical		Hypothetical protein	1,134	P	A	A	A	M	A	A	A	2,344	P	A	A	A	A	A	A	A
1031119	-	gbs1966		Hypothetical		Hypothetical protein	0,895	P	P	P	P	P	P	A	P	2,003	P	A	A	A	M	P	A	A
1031129	-	gbs1967		Hypothetical		Hypothetical cytosolic protein	0,516	P	P	P	P	P	P	P	P	1,198	P	A	P	P	P	P	A	P
1031122	-	gbs1968		Hypothetical		Hypothetical protein	0,415	P	P	P	P	P	P	P	P	1,935	P	A	P	A	P	P	A	P
1031130	-	gbs1969		Mobile and extrachromosomal elements		DNA integration recombination inversion protein	0,458	P	A	A	A	M	P	A	A	0,384	P	A	A	A	A	A	A	A
1031133	-	gbs1970		Hypothetical		Hypothetical protein	0,242	A	A	A	A	A	A	A	A	0,476	A	A	A	A	A	A	A	A
1031135	-	gbs1971		Hypothetical		Hypothetical protein	0,971	A	A	A	A	A	A	A	A	0,505	A	A	A	A	A	A	A	A
1031136	COG1396K	gbs1972		Cellular processess	Transcription	Transcriptional regulator, Cro CI family	0,981	P	P	P	P	P	P	P	P	0,617	P	A	P	A	P	P	A	P
1031128	-	gbs1973		Hypothetical		Hypothetical protein	1,622	P	P	P	P	P	P	M	P	1,349	P	P	P	A	P	P	P	P
1031142	-	gbs1974		Hypothetical		Hypothetical protein	1,085	P	A	A	A	A	A	A	A	1,819	P	A	A	A	A	A	A	A
1031140	COG2378K	gbs1975		Cellular processess	Transcription	Transcriptional regulator, DeoR family	1,092	P	P	P	P	P	P	M	P	1,928	P	A	P	A	A	P	A	A
1031143	-	gbs1976		Hypothetical		Hypothetical protein	0,323	P	P	P	P	P	P	P	P	1,804	P	A	A	A	A	A	A	A
1031137	-	gbs1977		Hypothetical		Hypothetical protein	0,903	P	P	P	P	P	P	P	P	0,546	P	A	A	P	A	P	P	A
1031131	-	gbs1978		Hypothetical		Hypothetical protein	0,750	P	P	P	A	P	P	A	P	0,862	P	A	P	A	A	A	A	A
1031134	COG4842S	gbs1979		Cellular processess	Posttranslational modification, protein turnover, chaperones	a small heat shock protein of Clostridium acetobutylicum homolog lmo0056	0,102	A	A	A	A	A	A	A	A	1,036	A	A	A	A	A	A	A	A
1031144	-	gbs1980		Hypothetical		Hypothetical secreted protein	0,919	P	A	P	P	P	P	P	P	1,290	P	A	A	A	A	A	A	A
1031178	-	gbs1981		Hypothetical		Hypothetical protein	0,567	P	P	P	P	P	P	P	P	1,958	P	A	P	M	P	A	P	P
1031222	COG3077L	gbs1982		Cellular processess	DNA replication, recombination and repair	DNA-damage-inducible protein J	1,028	A	A	A	A	A	A	A	A	0,080	A	A	A	A	A	A	A	A
1031139	-	gbs1983		Hypothetical		Hypothetical protein	1,830	P	A	A	A	M	A	A	A	1,757	P	A	A	A	A	A	A	A
1031221	-	gbs1984		Hypothetical		Hypothetical protein	1,626	P	M	P	P	P	P	A	P	1,745	P	A	P	P	P	P	A	A
1031226	-	gbs1985		Hypothetical		Hypothetical membrane spanning protein	2,224	P	P	P	P	P	P	M	P	2,178	P	A	P	A	A	P	A	A
1031224	0488R, COG1	gbs1986		Transport and binding proteins	Unknown substrate	ABC transporter ATP-binding protein	2,121	P	P	P	P	P	P	P	P	1,719	P	A	P	P	P	P	P	P
1031252	-	gbs1987		Cellular processess	Toxin production and resistance	Streptomycin adenylyltransferase (Aminoglycoside 6- adenylyltransferase) (Aminogl	1,551	P	A	P	A	P	A	A	A	2,193	P	A	A	A	A	A	A	A
1031273	-	gbs1988		Hypothetical		Hypothetical protein	1,328	P	A	A	A	A	M	A	A	0,488	P	A	A	A	A	A	A	A
1031271	-	gbs1989		Hypothetical		Hypothetical protein	1,250	P	A	A	A	P	P	P	A	0,590	P	A	A	A	A	P	A	A
1031272	COG1695K	gbs1990		Cellular processess	Transcription	Transcriptional regulator, PadR family	1,760	P	A	A	A	A	A	A	A	0,377	P	A	A	A	A	A	A	A
1031274	-	gbs1991		Metabolism and transport	Central intermediary metabolism	Acetyltransferase, GNAT family	0,829	M	A	A	A	A	A	A	A	0,345	A	A	A	A	A	A	A	A
1031282	-	gbs1992		Transport and binding proteins	Unknown substrate	ABC transporter permease protein	0,688	A	A	A	A	A	A	A	A	0,680	A	A	A	A	A	A	A	A
1031275	COG1131V	gbs1993		Transport and binding proteins	Unknown substrate	ABC transporter ATP-binding protein	0,479	P	A	A	A	A	M	M	A	0,444	P	M	A	A	A	A	A	A
1031279	COG1396K	gbs1994		Cellular processess	Transcription	Transcriptional regulator, Cro CI family	0,492	P	A	A	A	A	A	A	A	0,856	A	A	A	A	A	A	A	A
1031276	-	gbs1995		Metabolism and transport	Fatty acid and phospholipids	Phosphatidylglycerophosphatase B homolog	6,916	P	A	A	A	P	A	A	A	0,351	P	A	A	A	A	A	A	A
1031286	COG1694R	gbs1996		Hypothetical		Hypothetical protein	0,665	P	P	P	A	P	P	P	A	0,472	A	A	A	A	A	A	A	A
1031281	COG1611R	gbs1997		General function predicted only		Lysine decarboxylase family	0,656	P	P	P	P	P	P	P	P	0,383	P	P	P	P	P	P	P	P
1031284	COG1266R	gbs1998		Cellular processess	Posttranslational modification, protein turnover, chaperones	CAAX amino terminal protease family	1,393	P	M	P	A	M	P	P	P	1,579	P	A	A	A	A	P	A	A
1031280	COG0607P	gbs1999		Metabolism and transport	Inorganic ion transport and metabolism	Rhodanese-related sulfurtransferases	0,337	P	P	P	P	P	P	P	P	1,079	P	P	P	P	P	P	P	P
1031278	-	gbs2000	cfb	Cellular processess	Transcription	CAMP factor	0,378	P	P	P	P	P	P	P	P	1,101	P	P	P	P	P	P	P	P
1031283	COG0563F	gbs2001	flaR	Metabolism and transport	Purines, pyrimidines, nucleosides, and nucleotides	DNA topology modulation protein flar-related protein	1,046	A	A	A	A	A	A	A	A	0,496	A	A	A	A	A	A	A	A
1031285	COG0371C	gbs2002		Metabolism and transport	Energy production and conversion	Glycerol dehydrogenase (EC 1.1.1.6)	0,585	P	P	P	P	P	P	P	P	0,992	P	P	P	M	P	P	P	P
1031288	COG4420S	gbs2003		Hypothetical		Hypothetical membrane spanning protein	0,944	P	A	P	A	A	M	A	P	1,649	P	A	A	A	A	A	A	A
1031287	0646E, COG0	gbs2004		Metabolism and transport	Amino acids, peptides, aminosugars and amines	5-methyltetrahydrofolate--homocysteine methyltransferase (EC 2.1.1.13) homocys	12,847	P	A	A	A	A	A	A	A	17,965	P	A	A	A	A	A	A	A
1031289	COG0620E	gbs2005		Metabolism and transport	Amino acids, peptides, aminosugars and amines	5-methyltetrahydropteroyltriglutamate--homocysteine methyltransferase (EC 2.1.1.	6,044	P	A	P	A	A	A	A	A	8,225	P	A	A	A	A	A	A	A
1031291	COG4392S	gbs2006		Metabolism and transport	Amino acids, peptides, aminosugars and amines	Branched-chain amino acid transport protein azlD	2,904	P	A	A	A	M	P	M	A	2,238	P	A	A	A	A	P	A	A
1031296	COG1296E	gbs2007		Metabolism and transport	Amino acids, peptides, aminosugars and amines	Branched-chain amino acid transport protein azlC	2,314	P	P	P	P	P	P	P	P	1,020	P	A	A	A	A	A	A	P
1031304	COG1404O	gbs2008		Cellular processess	Posttranslational modification, protein turnover, chaperones	LPXTG Endopeptidase lactocepin (EC 3.4.21.96)	0,337	P	P	P	P	P	P	P	P	1,298	P	P	P	P	P	P	P	P
1031388	COG0745TK	gbs2009		Cellular processess	Transcription	Transcriptional regulatory protein	1,109	P	M	P	M	P	M	A	P	1,001	P	A	A	A	A	A	A	A
1031308	COG0642T	gbs2010		Cellular processess	Signal transduction	Two component system histidine kinase (EC 2.7.3.-)	0,694	P	A	M	A	A	A	A	A	0,964	P	A	A	A	A	A	A	A
1031392	-	gbs2011		Hypothetical		Hypothetical protein	0,517	P	P	P	P	P	P	P	P	1,048	P	A	P	A	M	M	A	A
1031290	COG0495J	gbs2012	leuS	Cellular processess	Translation, ribosomal structure and biogenesis	Leucyl-tRNA synthetase (EC 6.1.1.4)	1,451	P	P	P	P	P	P	P	P	1,130	P	P	P	A	P	P	P	P
1031390	-	gbs2013		Transport and binding proteins	Unknown substrate	Transporter, MFS superfamily	0,911	P	P	P	P	P	P	P	P	0,291	P	P	P	P	P	P	P	P
1031391	COG4667R	gbs2014		Cellular processess	Posttranslational modification, protein turnover, chaperones	Phospholipase (EC 3.1.-.-)	1,194	P	P	P	P	P	P	P	P	1,629	P	A	A	A	A	A	A	A
1031395	COG1442M	gbs2015		Metabolism and transport	Central intermediary metabolism	Glycosyl transferase, family 8	2,727	P	P	P	A	P	P	A	M	1,891	P	A	A	A	A	P	A	A
1031328	COG1442M	gbs2016		Metabolism and transport	Central intermediary metabolism	Glycosyl transferase, family 8	1,969	P	A	P	P	P	P	P	P	1,652	P	A	P	A	P	P	A	A
1031405	COG0250K	gbs2017	nusG	Cellular processess	Transcription	Transcription antitermination protein nusG	0,839	P	P	P	P	P	P	P	P	1,001	P	A	A	A	A	P	A	A
1031394	-	gbs2018	bibA	Hypothetical		LPXTG Hypothetical protein	0,688	P	P	P	P	P	P	P	P	7,522	P	P	P	P	P	P	P	P
1031393	COG0690U	gbs2019	secE	Cellular processess	Posttranslational modification, protein turnover, chaperones	Protein translocase subunit secE	2,312	P	P	P	P	P	P	P	P	0,943	P	P	P	P	P	P	P	P
1031409	COG0744M	gbs2020	pbp2A	Cell Envelope	Biosynthesis and degradation of murein sacculus and peptidoglycan	Multimodular transpeptidase-transglycosylase PBP 2A	1,365	P	P	P	P	P	P	P	P	0,810	P	A	P	M	P	P	A	P
1031404	COG0564J	gbs2021		Cellular processess	Translation, ribosomal structure and biogenesis	Ribosomal large subunit pseudouridine synthase D (EC 4.2.1.70)	2,242	P	A	A	A	A	P	P	M	1,031	P	A	M	A	A	A	A	A
1031397	COG4640S	gbs2022		Hypothetical		Hypothetical protein	1,877	P	P	P	P	P	P	P	P	0,796	P	A	A	A	P	P	P	P
1031407	COG0274F	gbs2024	deoC	Metabolism and transport	Purines, pyrimidines, nucleosides, and nucleotides	Deoxyribose-phosphate aldolase (EC 4.1.2.4)	1,051	P	P	P	P	P	P	P	P	1,251	P	P	P	P	P	P	P	P
1031408	-	gbs2025	nupC	Metabolism and transport	Purines, pyrimidines, nucleosides, and nucleotides	Nucleoside permease nupC	0,877	P	P	P	P	P	P	P	P	0,948	P	P	P	P	P	P	P	P
1031412	COG2820F	gbs2026	udp	Metabolism and transport	Purines, pyrimidines, nucleosides, and nucleotides	Uridine phosphorylase (EC 2.4.2.3)	0,763	P	P	P	P	P	P	P	P	0,973	P	P	P	P	P	P	P	P
1031415	COG2188K	gbs2027	crgR	Cellular processess	Transcription	Transcriptional regulator, GntR family	1,546	P	A	P	P	P	P	P	M	1,273	P	A	P	A	P	A	A	A
1031414	-	gbs2028		Hypothetical		Hypothetical protein	0,755	P	P	P	P	P	P	P	P	1,298	P	A	P	A	A	P	A	A
1031419	COG0459O	gbs2029	groEL	Cellular processess	Posttranslational modification, protein turnover, chaperones	60 kDa chaperonin GROEL	1,774	P	P	P	P	P	P	P	P	1,452	P	A	M	A	A	P	A	A
1031421	COG0234O	gbs2030	groES	Cellular processess	Posttranslational modification, protein turnover, chaperones	10 kDa chaperonin GROES	2,117	P	P	P	P	P	P	P	P	0,897	P	P	P	M	P	P	P	A
1031427	COG1101R	gbs2031		Transport and binding proteins	Unknown substrate	ABC transporter ATP-binding protein	0,935	P	A	A	A	A	A	A	A	1,894	A	A	A	A	A	A	A	A
1031423	COG4120R	gbs2032		Transport and binding proteins	Unknown substrate	ABC transporter permease protein	0,855	M	A	A	A	A	A	A	A	1,879	A	A	A	A	A	A	A	A
1031435	COG2984R	gbs2033		Transport and binding proteins	Unknown substrate	ABC transporter substrate-binding protein	1,617	A	A	A	A	A	A	A	A	1,726	A	A	A	A	A	A	A	A
1031459	COG0561R	gbs2034		Metabolism and transport	Central intermediary metabolism	Hydrolase (HAD superfamily)	0,991	P	P	P	P	P	P	P	P	1,670	P	P	P	A	P	P	P	A
1031451	COG2514R	gbs2035		Metabolism and transport	Energy production and conversion	Glyoxalase family protein	0,529	A	A	P	P	A	A	A	A	1,062	A	A	A	A	A	A	A	A

1031452	COG3022S	gbs2036	yaaA	Hypothetical		Hypothetical cytosolic protein	1,600	P	A	P	P	A	P	A	A	1,046	P	A	A	A	A	M	A	A
1031438	COG0602O	gbs2037	nrdG	Cellular processess	Posttranslational modification, protein turnover, chaperones	Anaerobic ribonucleoside-triphosphate reductase activating protein	2,601	P	A	P	P	P	M	A	A	1,398	P	A	P	A	A	M	A	A
1031463	COG3981R	gbs2038		Metabolism and transport	Central intermediary metabolism	Acetyltransferase (EC 2.3.1.-)	3,991	P	A	M	A	P	P	A	A	3,601	P	A	A	A	A	A	A	A
1031457	COG0673R	gbs2039		Metabolism and transport	Central intermediary metabolism	NAD-dependent oxidoreductase	2,480	P	A	P	A	P	M	A	M	2,195	P	A	A	A	A	A	A	A
1031458	-	gbs2040		Hypothetical		Hypothetical protein	1,747	P	P	P	P	P	P	P	P	1,903	P	P	P	P	P	P	P	P
1031480	COG1328F	gbs2041	nrdD	Metabolism and transport	Purines, pyrimidines, nucleosides, and nucleotides	Anaerobic ribonucleoside-triphosphate reductase (EC 1.17.4.2)	2,046	P	P	P	P	P	P	P	P	0,703	P	P	P	P	P	P	P	P
1031460	-	gbs2042		Hypothetical		Hypothetical membrane spanning protein	3,976	P	A	P	P	P	P	P	P	2,491	P	A	P	A	A	P	A	P
1031467	COG3906S	gbs2043		Hypothetical		Hypothetical cytosolic protein	0,625	P	P	P	P	P	P	P	P	1,474	P	P	P	P	P	P	P	P
1031487	-	gbs2044		Cellular processess	DNA replication, recombination and repair	Endonuclease involved in recombination	0,558	P	P	P	P	P	P	P	P	1,330	P	P	P	P	P	P	P	P
1031478	-	gbs2045		Hypothetical		Hypothetical cytosolic protein	0,434	P	P	P	P	P	P	P	P	1,002	P	P	P	P	P	P	P	P
1031465	COG1393P	gbs2046	spxA	Metabolism and transport	Inorganic ion transport and metabolism	Arsenate reductase family protein	0,734	P	P	P	P	P	P	P	P	0,679	P	P	P	P	P	P	P	P
1031471	COG0468L	gbs2047	recA	Cellular processess	DNA replication, recombination and repair	RecA protein	1,456	P	A	P	A	P	M	P	P	1,251	P	A	P	A	A	A	M	A
1031601	1058R, COG1	gbs2048	cinA	General function predicted only		Colligrin	1,380	P	A	A	M	M	M	A	P	0,476	P	A	A	A	A	A	A	A
1031543	-	gbs2049	tag	Cellular processess	DNA replication, recombination and repair	DNA-3-methyladenine glycosylase (EC 3.2.2.20)	2,487	P	M	P	A	P	P	A	P	1,557	P	A	P	A	P	P	A	A
1031545	COG0632L	gbs2050	ruvA	Cellular processess	DNA replication, recombination and repair	Holliday junction DNA helicase ruvA	5,742	P	A	A	A	A	A	A	A	3,162	P	A	A	A	A	A	A	A
1031501	-	gbs2051	lmrP	Cellular processess	Toxin production and resistance	Multidrug resistance protein imrP	5,579	P	A	P	A	A	P	A	A	3,410	P	A	P	A	A	P	A	A
1031648	COG0323L	gbs2052	mutL	Cellular processess	DNA replication, recombination and repair	DNA mismatch repair protein mutL	2,239	P	A	P	A	A	A	A	M	1,548	P	A	A	A	A	A	A	A
1031494	-	gbs2053	csp	Cellular processess	Posttranslational modification, protein turnover, chaperones	Cold shock protein	1,290	P	P	P	P	P	P	P	P	0,751	P	P	P	P	P	P	P	P
1031681	COG0249L	gbs2054	mutS	Cellular processess	DNA replication, recombination and repair	DNA mismatch repair protein mutS	0,816	P	A	A	A	A	A	A	A	0,710	P	A	A	A	A	A	A	A
1031544	COG1438K	gbs2055	argR2	Cellular processess	Transcription	Arginine repressor, argR	1,336	P	P	P	P	P	P	P	P	1,281	P	A	P	A	A	P	A	A
1031682	COG0018J	gbs2056	argS	Cellular processess	Translation, ribosomal structure and biogenesis	Arginyl-tRNA synthetase (EC 6.1.1.19)	1,159	P	P	P	P	P	P	P	P	1,125	P	P	P	P	P	P	P	P
1031685	-	gbs2057	uviB			Bacteriocin uviB	1,674	P	P	P	P	P	P	P	P	2,530	P	A	P	A	A	P	A	A
1031680	COG1284S	gbs2058		Hypothetical		Hypothetical membrane spanning protein	1,496	P	P	P	P	P	P	A	P	0,569	P	M	P	M	P	P	A	P
1030780	COG1284S	gbs2059		Hypothetical		Hypothetical membrane spanning protein	2,959	P	P	P	P	P	P	P	P	0,967	P	P	P	A	P	P	P	P
1031827	COG0173J	gbs2060	aspS	Cellular processess	Translation, ribosomal structure and biogenesis	Aspartyl-tRNA synthetase (EC 6.1.1.12)	2,238	P	P	P	M	P	P	P	P	0,928	P	A	M	A	P	M	P	M
1031683	COG0124J	gbs2061	hisS	Cellular processess	Translation, ribosomal structure and biogenesis	Histidyl-tRNA synthetase (EC 6.1.1.21)	2,134	P	M	P	A	P	P	P	A	1,301	P	A	A	A	A	A	A	A
1031684	COG0333J	gbs2062	rpmF	Cellular processess	Translation, ribosomal structure and biogenesis	LSU ribosomal protein L32P	0,853	P	P	P	P	P	P	P	P	0,683	P	P	P	P	P	P	P	P
1030811	COG0267J	gbs2063	rpmG	Cellular processess	Translation, ribosomal structure and biogenesis	LSU ribosomal protein L33P	0,590	P	P	P	P	P	P	P	P	0,638	P	P	P	P	P	P	P	P
1030716	-	gbs2064	cadD	Cellular processess	Toxin production and resistance	Cadmium resistance protein	1,456	P	A	A	A	A	A	M	A	1,357	P	A	A	A	A	A	A	A
1030808	COG0640K	gbs2065	cadC	Cellular processess	Toxin production and resistance	Cadmium efflux system accessory protein	1,158	P	A	M	A	P	P	A	A	1,398	P	A	A	A	A	A	A	A
1030814	-	gbs2066		Hypothetical		Hypothetical protein	1,156	P	M	P	P	P	P	P	P	2,120	P	A	A	A	M	A	A	A
1030813	-	gbs2067		Hypothetical		Hypothetical protein	0,567	P	A	M	A	A	A	A	A	0,364	A	A	A	A	A	A	A	A
1030815	-	gbs2068		Hypothetical		Hypothetical protein	0,665	P	P	P	A	P	A	P	P	0,596	P	A	M	P	M	P	P	A
1030830	COG1674D	gbs2069		Cellular processess	Cell division	FtsK SpoIIIE family	1,037	P	A	A	A	A	A	A	A	0,840	P	A	A	A	A	A	A	A
1031433	-	gbs2070		Hypothetical		Hypothetical protein	1,152	P	A	A	A	A	A	A	A	3,029	P	A	A	A	A	A	A	A
1030817	-	gbs2071		Mobile and extrachromosomal elements		Replication protein	1,141	A	A	A	A	A	A	A	A	0,326	A	A	A	A	A	A	A	A
1030783	-	gbs2072		Mobile and extrachromosomal elements		Phage protein	3,419	P	A	A	A	A	A	A	A	1,351	A	A	A	A	A	A	A	A
1030747	COG0582L	gbs2073		Mobile and extrachromosomal elements		DNA integration recombination inversion protein	2,301	P	P	P	P	P	P	P	P	1,311	P	P	P	P	P	P	P	P
1030842	-	gbs2074		Hypothetical		Hypothetical cytosolic protein	5,410	P	A	P	A	P	P	A	P	2,221	P	A	P	M	M	P	A	A
1030781	-	gbs2076		Hypothetical		Hypothetical protein	1,525	P	M	P	P	P	P	P	P	1,307	P	A	P	P	A	M	A	A
1030820	-	gbs2077		Hypothetical		Hypothetical protein	3,107	P	A	A	P	P	P	P	A	0,785	P	A	A	A	A	A	A	A
1031055	COG3212S	gbs2080		Hypothetical		Hypothetical protein	1,059	P	A	A	A	A	A	A	A	0,126	A	A	A	A	A	A	A	A
1030819	COG0745TK	gbs2081		Cellular processess	Transcription	Transcriptional regulatory protein	1,012	P	P	P	P	P	P	P	P	1,162	P	A	P	M	M	P	P	A
1030759	COG0642T	gbs2082		Cellular processess	Signal transduction	Two-component sensor kinase czcS (EC 2.7.3.-)	1,329	P	P	P	P	P	P	M	P	2,094	P	A	A	A	A	P	A	A
1030703	COG1288S	gbs2083		Metabolism and transport	Amino acids, peptides, aminosugars and amines	Arginine ornithine antiporter	0,308	A	A	A	A	A	A	A	A	6,813	P	A	A	A	A	A	A	A
1030750	COG0549E	gbs2084		Metabolism and transport	Amino acids, peptides, aminosugars and amines	Carbamate kinase (EC 2.7.2.2)	1,249	P	A	A	A	A	A	A	A	3,280	P	A	A	P	A	A	A	A
1031013	COG0078E	gbs2085		Metabolism and transport	Amino acids, peptides, aminosugars and amines	Ornithine carbamoyltransferase (EC 2.1.3.3)	0,753	P	A	P	A	P	P	P	P	1,972	P	A	P	P	A	P	M	P
1031066	COG0642T	gbs2086		Cellular processess	Signal transduction	Sporulation kinase A (EC 2.7.3.-)	1,675	P	A	A	A	A	A	A	A	1,760	A	A	A	A	A	A	A	A
1030729	COG4753T	gbs2087		Cellular processess	Signal transduction	Two-component response regulator	1,391	P	A	A	A	A	A	A	A	1,617	P	A	A	A	A	M	A	A
1031098	COG1125E	gbs2088	proV	Metabolism and transport	Amino acids, peptides, aminosugars and amines	Glycine betaine transport ATP-binding protein	1,815	P	P	P	P	P	P	P	P	1,524	P	A	P	P	P	P	A	P
1030749	1174E, COG17	gbs2089		Metabolism and transport	Amino acids, peptides, aminosugars and amines	Glycine betaine transport system permease protein Glycine betaine-binding prote	1,474	P	P	P	P	P	P	P	P	1,048	P	P	P	P	P	P	P	P
1031102	0392S, COG2	gbs2090		General function predicted only		Lysylcardiolipin synthase (EC 2.3.2.-) Lysyltransferase (EC 2.3.2.3)	1,595	P	P	P	P	P	P	P	P	1,521	P	A	P	P	P	P	A	P
1031088	COG0596R	gbs2091		General function predicted only		Non-heme chloroperoxidase (EC 1.11.1.10)	2,405	P	P	P	P	P	P	M	P	1,566	P	A	P	P	A	M	A	A
1030688	COG3759S	gbs2092		Hypothetical		Hypothetical membrane spanning protein	1,475	A	A	A	A	A	A	A	A	0,162	A	A	A	A	A	A	A	A
1030562	COG1511S	gbs2093		Mobile and extrachromosomal elements		Phage infection protein	3,144	A	A	A	A	A	A	A	A	0,130	A	A	A	A	A	A	A	A
1030957	COG1309K	gbs2094		Cellular processess	Transcription	Transcriptional regulator, TetR family	1,436	P	A	A	A	A	A	A	A	1,060	P	A	A	A	A	A	A	A
1030235	-	gbs2095		Hypothetical		Hypothetical protein	3,872	P	A	A	A	A	A	A	A	0,565	A	A	A	A	A	A	A	A
1029947	COG0522J	gbs2096	rpsD	Cellular processess	Translation, ribosomal structure and biogenesis	SSU ribosomal protein S4P	1,888	P	P	P	P	P	P	P	P	0,477	P	P	P	P	P	P	P	P
1031112	-	gbs2097		Hypothetical		Hypothetical protein	1,943	P	P	P	P	P	P	P	P	1,504	P	P	P	P	P	P	P	P
1031046	COG0305L	gbs2098	holB	Cellular processess	DNA replication, recombination and repair	Replicative DNA helicase (EC 3.6.1.-)	2,110	P	P	P	P	P	P	P	P	1,304	P	A	P	A	P	P	P	P
1030821	COG0359J	gbs2099	rplI	Cellular processess	Translation, ribosomal structure and biogenesis	LSU ribosomal protein L9P	1,659	P	P	P	P	P	P	P	P	1,182	P	P	P	P	P	P	P	P
1030960	COG3887T	gbs2100		Metabolism and transport	Central intermediary metabolism	Phosphoesterase, DHH family protein	2,188	P	P	P	P	P	P	P	P	1,292	P	P	P	P	P	P	P	P
1030341	COG0445D	gbs2101	gidA	General function predicted only		Putative tRNA (5-carboxymethylaminomethyl-2-thiouridylate) synthase subunit Gid	1,420	P	A	M	A	M	A	A	A	1,287	P	A	A	A	A	A	A	A
1031036	COG2095U	gbs2102		Cell Envelope	Other	MarC family integral membrane protein	2,776	P	A	A	A	A	A	A	A	0,827	P	A	A	A	A	A	A	A
1030810	COG0482J	gbs2103	trmU	Cellular processess	Translation, ribosomal structure and biogenesis	tRNA (5-methylaminomethyl-2-thiouridylate)-methyltransferase (EC 2.1.1.61)	2,183	P	A	A	A	A	A	A	A	0,767	P	A	A	A	A	A	A	A
1031056	COG1760E	gbs2104	sdhB	Metabolism and transport	Amino acids, peptides, aminosugars and amines	L-serine dehydratase beta subunit (EC 4.3.1.17)	3,552	P	A	A	A	A	A	A	A	2,469	P	A	A	A	A	A	A	A
1030711	COG1760E	gbs2105	sdhA	Metabolism and transport	Amino acids, peptides, aminosugars and amines	L-serine dehydratase alpha subunit (EC 4.3.1.17)	1,080	P	A	P	A	M	A	A	A	1,023	P	A	M	A	A	A	A	A
1031113	-	gbs2106		General function predicted only		Transglycosylase SLT domain family protein	2,358	P	A	A	A	A	A	A	A	1,070	M	A	A	A	A	A	A	A
1031059	-	gbs2107		Cell Envelope	Biosynthesis and degradation of surface polysaccharides and lipopo	peptidoglycan binding protein (LysM domain)	1,340	P	A	P	A	P	P	P	P	0,904	P	A	A	A	A	A	A	A
1030956	COG0619P	gbs2108	cbiQ	Metabolism and transport	Inorganic ion transport and metabolism	Hyaluronan ABC exporter permease protein	1,432	P	P	P	P	P	P	P	P	0,778	P	A	P	A	P	P	A	A
1030712	COG1122P	gbs2109	cbiO2	Metabolism and transport	Inorganic ion transport and metabolism	Hyaluronan ABC exporter ATP-binding protein	2,362	P	A	A	P	P	A	P	P	0,840	P	A	P	A	A	A	P	A
1030739	COG1122P	gbs2110	cbiO1	Metabolism and transport	Inorganic ion transport and metabolism	Hyaluronan ABC exporter ATP-binding protein	1,860	P	M	P	A	P	P	P	P	1,179	P	A	A	A	A	A	M	A
1030710	COG0558I	gbs2111	pgsA	Metabolism and transport	Fatty acid and phospholipids	CDP-diacylglycerol--glycerol-3-phosphate 3-phosphatidyltransferase (EC 2.7.8.5)	1,649	P	P	P	P	P	P	P	P	0,986	P	P	P	M	P	P	P	P
1030721	COG0612R	gbs2112		Cellular processess	Posttranslational modification, protein turnover, chaperones	Peptidase, M16 family	2,763	P	A	P	A	P	P	P	M	1,346	P	P	P	A	A	P	A	P
1030765	COG0612R	gbs2113		General function predicted only		Non-proteolytic protein, peptidase family M16	3,767	P	A	A	A	A	A	A	A	0,968	P	A	A	A	A	A	A	A
1030822	COG2501S	gbs2114		Hypothetical		Hypothetical cytosolic protein	2,435	P	P	P	P	P	P	P	P	0,974	P	A	P	A	P	P	A	A
1030733	COG1195L	gbs2115	recF	Cellular processess	DNA replication, recombination and repair	DNA replication and repair protein recF	1,935	P	P	P	P	P	P	P	P	0,816	P	P	P	P	P	P	P	P
1031093	COG4975G	gbs2116		Metabolism and transport	Carbohydrates, organic alcohols, and acids	Glucose uptake family protein	2,948	P	P	P	P	P	P	P	P	1,467	P	A	P	M	P	P	P	P
1031097	-	gbs2117		Cellular processess	Transcription	Transcriptional regulator, Cro CI family	8,472	P	A	P	A	A	P	P	A	2,233	P	A	A	A	A	A	A	A
1030752	G0516F, COG0	gbs2118	guaB	Metabolism and transport	Purines, pyrimidines, nucleosides, and nucleotides	Inosine-5 -monophosphate dehydrogenase (EC 1.1.1.205)	3,519	P	P	P	P	P	P	P	P	3,147	P	M	P	A	P	P	P	A
1030751	COG1438K	gbs2119	ahrC.2	Cellular processess	Transcription	Arginine repressor, argR	1,153	P	M	P	A	P	P	P	M	0,844	P	A	A	A	A	P	P	A
1031069	COG0664T	gbs2120		Cellular processess	Transcription	Transcriptional regulatory protein	1,679	P	P	P	P	P	P	P	P	0,960	P	A	P	P	P	P	P	P
1030823	COG3382S	gbs2121		Hypothetical		Hypothetical protein	1,515	P	A	A	A	A	A	A	A	1,211	P	A	A	A	A	A	A	A
1031077	COG2235E	gbs2122	arcA	Metabolism and transport	Amino acids, peptides, aminosugars and amines	Arginine deiminase (EC 3.5.3.6)	0,067	P	P	P	P	P	P	P	P	1,048	P	P	P	P	P	P	P	P
1031073	-	gbs2123		Metabolism and transport	Central intermediary metabolism	Acetyltransferase (EC 2.3.1.-)	0,056	P	P	P	P	P	P	P	P	0,872	P	P	P	P	P	P	P	P
1031070	COG0078E	gbs2124	arcB	Metabolism and transport	Amino acids, peptides, aminosugars and amines	Ornithine carbamoyltransferase (EC 2.1.3.3)	0,139	P	P	P	P	P	P	P	P	0,629	P	P	P	P	P	P	P	P
1030825	COG0531E	gbs2125		Metabolism and transport	Amino acids, peptides, aminosugars and amines	Arginine ornithine antiporter	0,200	P	P	P	M	P	P	P	P	0,752	P	P	P	P	P	P	P	P
1030767	COG0549E	gbs2126	arcC	Metabolism and transport	Amino acids, peptides, aminosugars and amines	Carbamate kinase (EC 2.7.2.2)	0,419	P	P	M	P	P	P	P	P	0,652	P	P	P	P	P	P	P	P
1030760	COG0180J	gbs2127	trpS	Cellular processess	Translation, ribosomal structure and biogenesis	Tryptophanyl-tRNA synthetase (EC 6.1.1.2)	2,194	P	P	P	P	P	P	P	P	1,498	P	A	P	P	P	P	P	A
1030806	-	gbs2128		Hypothetical		Hypothetical membrane spanning protein	1,201	P	A	A	A	A	P	A	A	0,506	P	A	M	A	A	A	A	A
1030763	COG1284S	gbs2129		Hypothetical		Hypothetical membrane spanning protein	2,845	P	A	P	A	P	P	P	P	1,015	P	A	P	A	A	P	P	P
1031072	COG0488R	gbs2130		Transport and binding proteins	Unknown substrate	ABC transporter ATP-binding protein	1,493	P	P	P	P	P	P	P	P	0,906	P	A	P	P	P	P	A	P
1031067	-	gbs2131		Transport and binding proteins	Unknown substrate	ABC transporter, permease protein	1,713	A	A	A	A	A	A	A	A	0,912	M	A	A	A	A	A	A	A
1031071	COG1576S	gbs2132		Hypothetical		Hypothetical cytosolic protein	1,270	P	P	P	P	P	P	P	P	1,506	P	A	P	P	P	P	P	P
1030829	COG0265O	gbs2133	htrA	Cellular processess	Posttranslational modification, protein turnover, chaperones	Endopeptidase degP (EC 3.4.21.-)	1,943	P	P	P	P	P	P	P	P	1,827	P	P	P	A	P	P	P	P

1031012	COG1475K	gbs2134	parB	Cellular processess	Transcription	Chromosome partitioning protein parB	1,618	P	P	P	M	P	P	P	P	1,242	P	A	P	A	A	A	A	A
